# Benzene construction *via* Pd-catalyzed cyclization of 2,7-alkadiynylic carbonates in the presence of alkynes†
†Electronic supplementary information (ESI) available: Experimental section, characterization of all the compounds, and copies of ^1^H NMR and ^13^C NMR spectra. CCDC 1849157, 1849158 and 1855724. For ESI and crystallographic data in CIF or other electronic format see DOI: 10.1039/c8sc04681f


**DOI:** 10.1039/c8sc04681f

**Published:** 2018-12-19

**Authors:** Yuchen Zhang, Wangteng Wu, Chunling Fu, Xin Huang, Shengming Ma

**Affiliations:** a Laboratory of Molecular Recognition and Synthesis , Department of Chemistry , Zhejiang University , Hangzhou 310027 , Zhejiang , People's Republic of China . Email: xinhuangzju@zju.edu.cn ; Email: masm@sioc.ac.cn

## Abstract

A palladium-catalyzed highly regio- and chemo-selective cyclization of 2,7-alkadiynylic carbonates with functionalized alkynes to construct 1,3-dihydroisobenzofuran and isoindoline derivatives under mild conditions has been developed. Functional groups such as alcohol, sulfonamide, and indoles could be well tolerated. After careful mechanistic studies, a mechanism involving oxidative addition and regioselectivity-defined double alkyne insertions has been proposed.

## Abstract 2

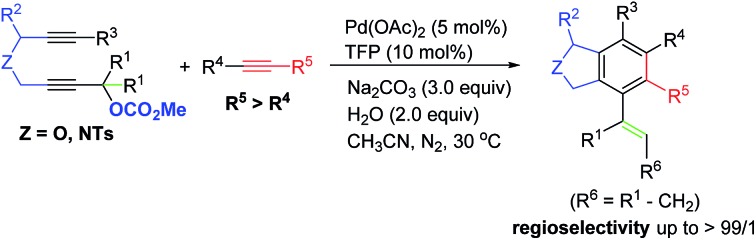
A palladium-catalyzed highly regio- and chemo-selective cyclization to construct 1,3-dihydroisobenzofuran and isoindoline derivatives under mild conditions has been developed.

## Introduction

**Fig. 1:**
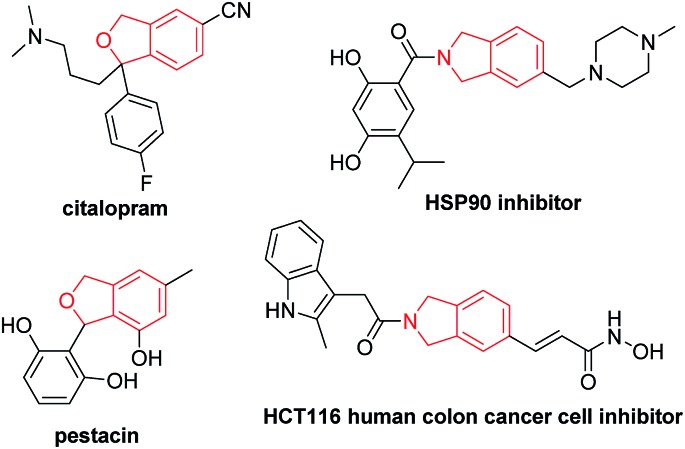
Some typical natural or bioactive phthalans and isoindolines compounds.

Benzocyclopentane derivatives, especially those containing oxygen and nitrogen heterocycles, exist widely in natural products and biologically active molecules:^1^ the phthalan (1,3-dihydroisobenzofuran) and isoindoline skeletons are representative examples (Fig. 1),^2^ which are also important building blocks in organic synthesis.^3^ Common approaches for the construction of phthalan and isoindoline structures include: (1) [2 + 2 + 2] cycloaddition reactions of 1,6-diynes with alkynes;^4^–^11^ (2) tetradehydro-Diels–Alder reaction of eneynes and alkynes or hexadehydro-Diels–Alder reaction of 1,3,8-triynes and electrophiles;^12^ (3) domino reaction consisting of Heck couplings and consecutive 6π-electrocyclizations or Sonogashira couplings and sequenced Garratt–Braverman cyclization of alkenyl halides and 1,6-diynes.^13^

**Scheme 1:**
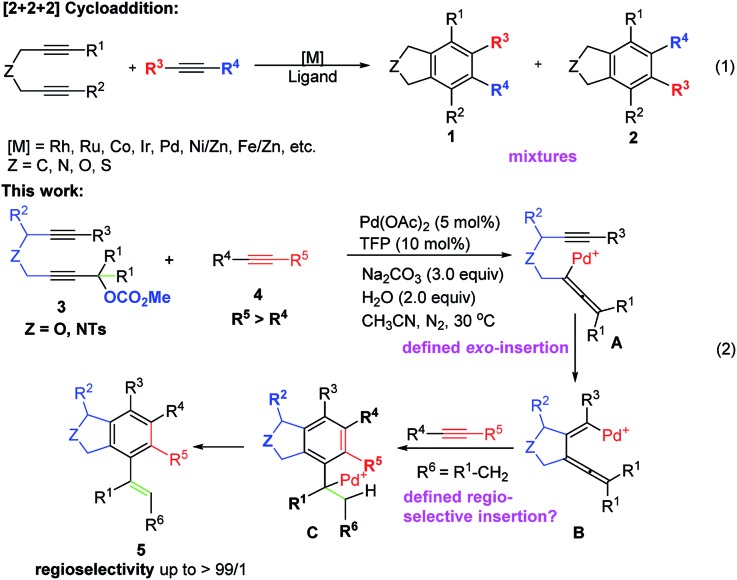
The transition metal-catalyzed [2 + 2 + 2] cyclotrimerization and cyclization of 2,7-alkadiynyl carbonate **3** in the presence of functionalized alkynes **4**.

Among all these methods, transition metal-catalyzed [2 + 2 + 2] cyclization of 1,6-diynes and alkynes is the most straightforward one.^4^–^11^ However, there is an issue of regioselectivity when non-symmetric 1,6-diynes and non-symmetric alkynes were applied (Scheme 1, eqn (1)).^4^–^11^ Based on our previous explorations in the tandem reactions between 2,7-alkadiynyl carbonates **3** and various allenes to construct fused tricycles,^14^ we envisioned a new approach to benzocyclopentanes by applying Pd-catalyzed tandem reaction of 2,7-alkadiynyl carbonates **3** with functionalized terminal or non-terminal alkynes **4**, in which the selectivity issue may be addressed by starting the cyclization from the oxidative addition of the propargylic carbonate unit to afford allenylpalladium intermediate **A**. Then the defined exo-insertion of the intramolecular alkyne and the subsequent regio-selective insertion of the intermolecular alkyne would produce benzylpalladium intermediate **C**, which underwent β-H elimination to give the final alkenyl benzene product (Scheme 1, eqn (2)). Here we wish to report the realization of such a concept.

## Results and discussion

Initially we conducted the reaction of 2,7-alkadiynylic carbonate **3a** (0.3 mmol) and 4-methyl-*N*-(prop-2-yn-1-yl)benzenesulfonamide **4a** (1.2 equiv.) under the catalysis of Pd(OAc)_2_ (5 mol%) and TFP (10 mol%) in the presence of K_2_CO_3_ (3.0 equiv.) and H_2_O (2.0 equiv.) at 70 °C in CH_3_CN. Interestingly, a pair of regioisomers **5aa** and **6aa** were observed in 81% and 3% yields, respectively, demonstrating a high regioselectivity. In addition, 2% yield of the cyclization product **7aa** was obtained (entry 1, Table 1). Encouraged by this exciting observation, the influence of the critical reaction parameters was investigated. Firstly, considering of our previous works,14b,^15^ an appropriate amount of water may increase the solubility of K_2_CO_3_ in CH_3_CN, the effect of water was tested: the reaction failed to give better results when more water or no water were added (entries 2 and 3, Table 1). After screening a series of mono-phosphine ligand such as PPh_3_, LB-Phos·HBF_4_,^16^ and Gorlos-Phos·HBF_4_,^17^ it was found that TFP was still the best (entries 4–6, Table 1). The effect of base was also investigated: the reactions using NaOH or NEt_3_ as the base produced **5aa** in lower yields with a poorer selectivity (entries 7 and 8, Table 1). Na_2_CO_3_ was slightly better than K_2_CO_3_, resulting in 82% yield of **5aa** (entry 9, Table 1).

**Table 1:** The effect of water, ligand, and base^*a*^

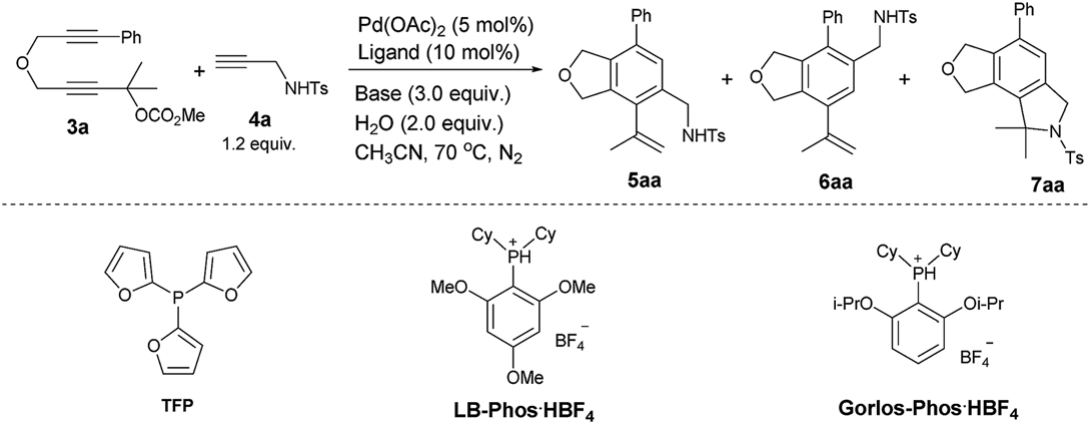					
Entry	Ligand	Base	Time (h)	Yield of **5aa**/**6aa**/**7aa**^*b*^ (%)	Recovery of **3a**^*b*^ (%)
1	TFP	K_2_CO_3_	2	81/3/2	0
2^*c*^	TFP	K_2_CO_3_	2	76/3/3	0
3^*d*^	TFP	K_2_CO_3_	2	70/4/3	0
4	PPh_3_	K_2_CO_3_	26	37/5/5	14
5	LB-Phos·HBF_4_	K_2_CO_3_	24	19/3/2	43
6	Gorlos-Phos·HBF_4_	K_2_CO_3_	24	31/4/2	27
7	TFP	NaOH	2	64/5/4	0
8	TFP	Et_3_N	2	77/4/2	0
**9**	**TFP**	**Na**_**2**_ **CO**_**3**_	**2**	**82/3/2**	**0**

^*a*^Reaction condition: **3a** (0.3 mmol), **4a** (1.2 equiv.), Pd(OAc)_2_ (5 mol%), ligand (10 mol%), base (3.0 equiv.), and H_2_O (2.0 equiv.) in CH_3_CN (3.0 mL) at 70 °C unless otherwise noted.

^*b*^Determined by the ^1^H NMR analysis of the crude product using mesitylene as the internal standard.

^*c*^H_2_O (4.0 equiv.) were added.

^*d*^No H_2_O was added.

Further solvent screening showed that the reactions in dioxane, DMSO, DMF, or DCE all delivered poorer results than those in CH_3_CN (entries 1–4, Table 2). It is worth mentioning that the efficiency, yield, and selectivity could be kept at the same level when the reaction was conducted at a lower temperature of 30 °C (entries 5–7, Table 2). The reaction at 10 °C is sluggish (entry 8, Table 2). Based on these studies, the optimal mild conditions have been defined as follows: Pd(OAc)_2_ (5 mol%), TFP (10 mol%), Na_2_CO_3_ (3.0 equiv.), and H_2_O (2.0 equiv.) in CH_3_CN at 30 °C.

**Table 2:** The effect of solvent and temperature^*a*^

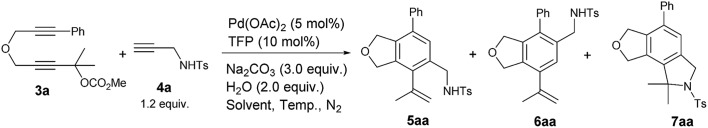					
Entry	Solvent	Temp. (°C)	Time (h)	Yield of **5aa**/**6aa**/**7aa**^*b*^ (%)	Recovery of **3a**^*b*^ (%)
1	Dioxane	70	2	72/6/4	0
2	DMSO	70	26	48/3/3	25
3	DMF	70	2	76/3/5	0
4	DCE	70	6	49/7/3	0
5	CH_3_CN	80	2	80/3/3	0
6	CH_3_CN	60	2	80/3/3	0
**7**	**CH**_**3**_ **CN**	**30**	**2**	**82/3/2**	**0**
8	CH_3_CN	10	26	68/2/4	3

^*a*^Reaction condition: **3a** (0.3 mmol), **4a** (1.2 equiv.), Pd(OAc)_2_ (5 mol%), TFP (10 mol%), Na_2_CO_3_ (3.0 equiv.), and H_2_O (2.0 equiv.) in solvent (3.0 mL).

^*b*^Determined by the ^1^H NMR analysis of the crude product using mesitylene as the internal standard.

**Fig. 2:**
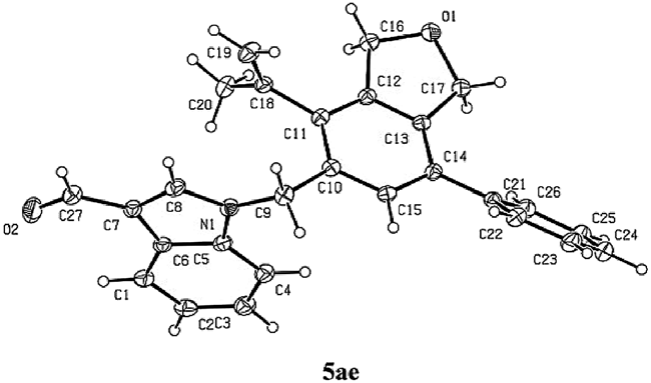
ORTEP representation of **5ae**.

The scope of the terminal alkynes was examined by using methyl (2-methyl-5-((3-phenylprop-2-yn-1-yl)oxy)pent-3-yn-2-yl) carbonate (**3a**) as the model substrate on a 1 mmol scale (Table 3). The reaction of **3a** with propargyl tosylamide **4a** afforded **5aa**/**6aa** in 78% yield with a selectivity of 97/3. When propargyl alcohol **4b** was used, **5ab**/**6ab** was obtained in 72% yield with the same selectivity. In light of the fact that the indole skeletons are very common in natural products and biologically active molecules,^18^ it is interesting to note that the reactions with a series of *N*-propargyl indole derivatives (**4c–4f**) also worked, affording the phthalan derivatives bearing an indole ring **5ac**/**6ac**–**5af**/**6af** in 66–69% yields with a ratio of 95/5 ∼> 99/1. Synthetically useful groups such as methyl, formyl, bromo could be introduced at different positions in the indole ring. The structure of product was unambiguously established by the X-ray single crystal diffraction analysis of **5ae** (Fig. 2).^19^

**Table 3:** Scope of terminal alkynes **4**.^*a*^ ^,^^*b*^ ^,^^*c*^

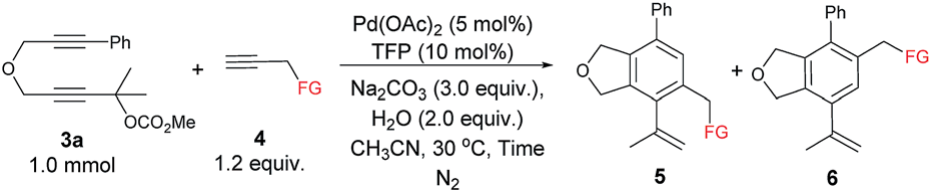
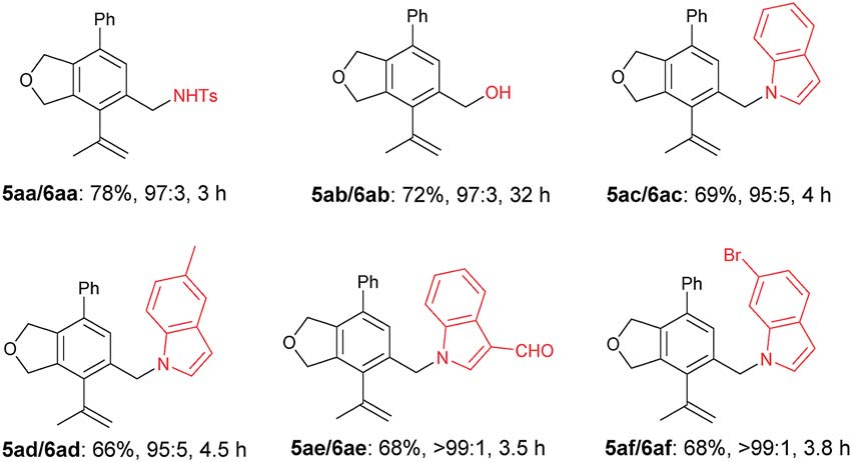

^*a*^Reaction conditions: **3a** (1.0 mmol), **4** (1.2 equiv.), Pd(OAc)_2_ (5 mol%), TFP (10 mol%), Na_2_CO_3_ (3.0 equiv.), and H_2_O (2.0 equiv.) in CH_3_CN (10 mL) at 30 °C.

^*b*^Combined isolated yield of **5** and **6**.

^*c*^The ratio of **5** and **6** was determined by ^1^H NMR analysis of the isolated product.

Next, the reactivity of various oxygen- or nitrogen-tethered 2,7-alkadiynylic carbonates was examined with different functionalized terminal alkynes (Table 4). In addition to being methyl groups, the two R^1^ groups could be a five- or six-membered ring. The corresponding products **5ba**/**6ba** and **5ja**/**6ja** were isolated in 79% yield with a selectivity of 98/2 and 53% yield with a selectivity of 90/10, respectively. The substrates with the Ar group bearing either electron-rich or electron-deficient groups at the 8-position could also be applied, producing the expected products **5cb**/**6cb**–**5fg**/**6fg** in 59%–85% yields with the ratio of 93/7–97/3. This method could be extended to 8-(3′-thienyl), 8-^*n*^Bu, 8-TMS and 6-propyl substituted 2,7-alkadiynylic carbonates to afford **5ga**/**6ga**–**5ha**/**6ha** in moderate to good yields with the selectivity of 96/4 to >99/1. Nitrogen-tethered 2,7-alkadiynylic carbonate **3i** also worked and the isoindoline derivatives **5ia**/**6ia** were obtained in 67% yield with a selectivity of 98/2.

**Table 4:** The reaction of 2,7-alkadiynylic carbonates **3** with functionalized terminal alkynes **4**^*a*^ ^,^^*b*^ ^,^^*c*^

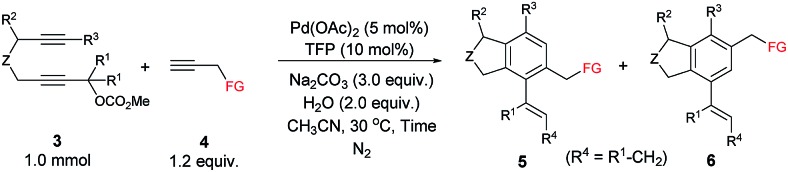
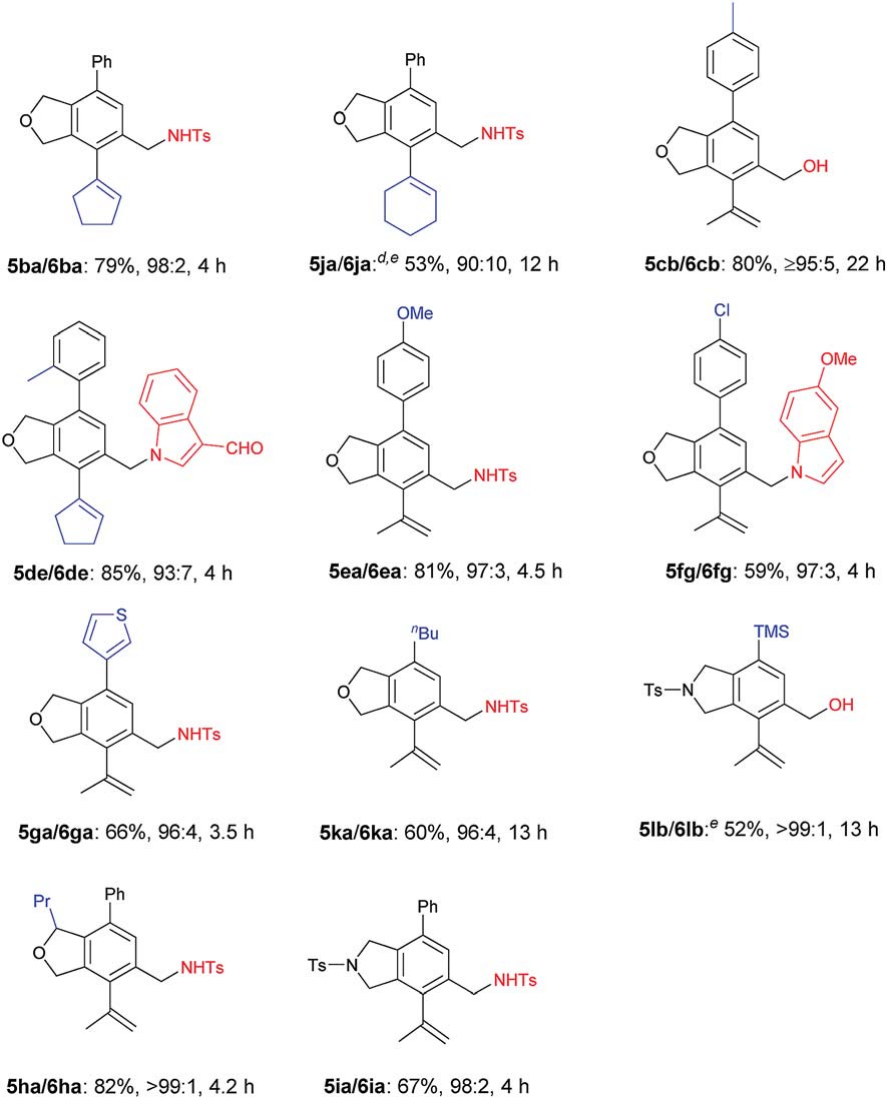

^*a*^Reaction conditions: **3** (1.0 mmol), **4** (1.2 equiv.), Pd(OAc)_2_ (5 mol%), TFP (10 mol%), Na_2_CO_3_ (3.0 equiv.), H_2_O (2.0 equiv.) in CH_3_CN (10 mL) at 30 °C.

^*b*^Combined yield of **5** and **6**.

^*c*^The ratio of **5** and **6** was determined by ^1^H NMR analysis of the isolated product.

^*d*^The reaction was conducted at 50 °C and 11% of **3j** was recovered.

^*e*^0.5 mmol scale.

The reaction of **3a** with **4a** could be easily conducted on a gram-scale synthesis, resulting in the isolation of 1.42 g (85%) of **5aa**/**6aa** with a selectivity of 97/3 together with a 1% yield of **7aa** (eqn (3)).
3

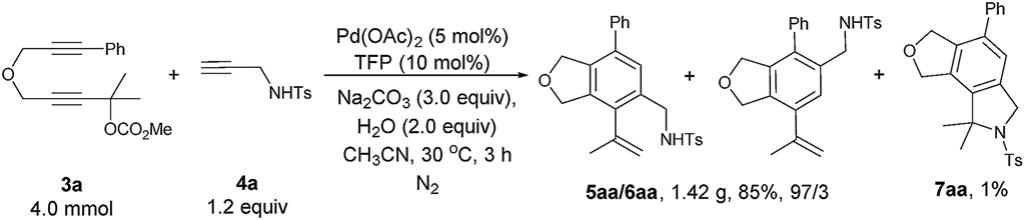




**Scheme 2:**
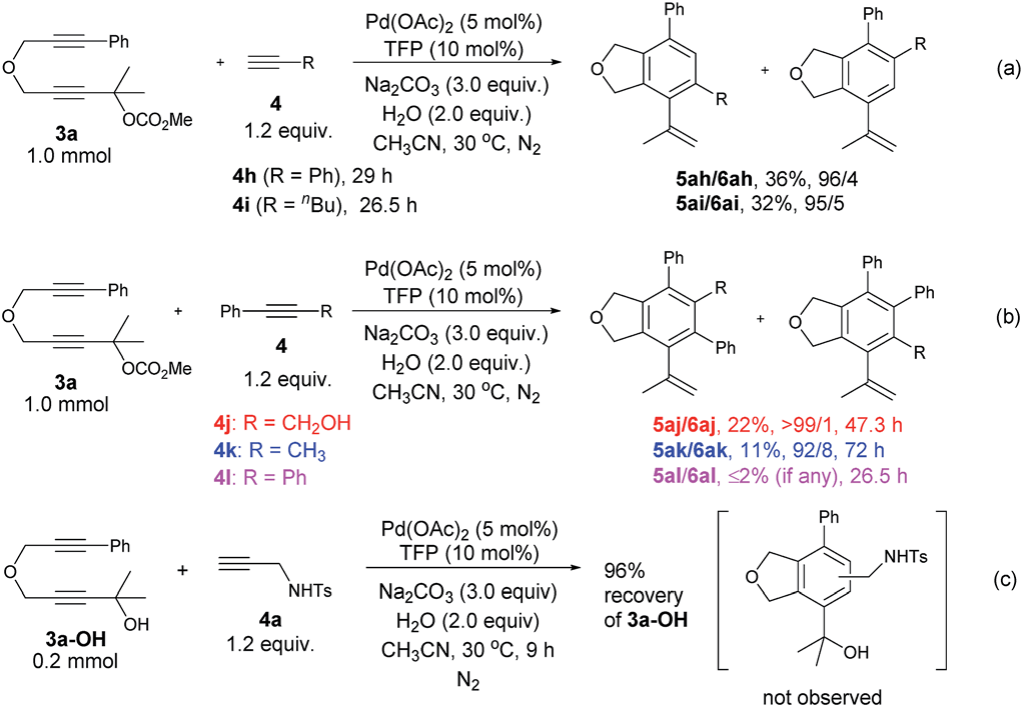
Control experiments.

**Fig. 3:**
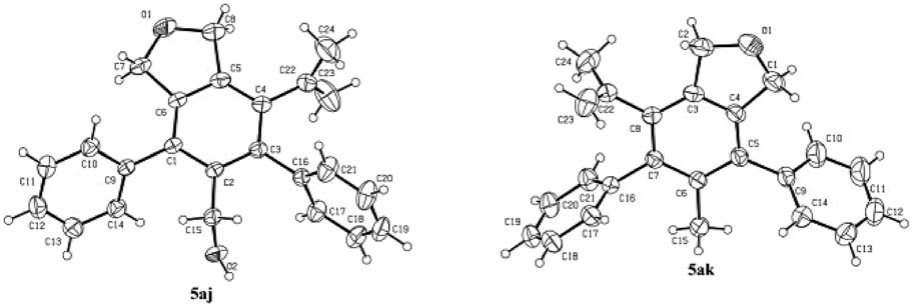
ORTEP representations of **5aj** and **5ak**.

Some control experiments were conducted to obtain further information concerning the regioselectivity (Scheme 2). When phenyl or *n*-butyl substituted terminal alkyne **4h** or **4i** was used, the corresponding bicyclic product could still be formed with a decent regioselectivity, albeit in a much lower yield (Scheme 2a). Furthermore, the reaction of **3a** with internal propargylic alcohol **4j** could give 22% yield of the product **5aj** exclusively (Scheme 2b). X-ray single crystal diffraction analysis of **5aj** showed that the regioselectivity was reversed-the CH_2_OH group was at the *ortho*-position of the phenyl group from **3a** (Fig. 3).^20^ The reaction of **3a** with 1-phenylpropyne **4k** also gave **5ak**/**6ak** in 11% yield with the same regioselectivity as **4j**, indicating that the hydroxy group should have nothing to do with the regioselectivity (Scheme 2b). The structure of **5ak** was also confirmed by X-ray single crystal diffraction analysis (Fig. 3).^21^ However, the reaction did not work with 1,2-diphenylethyne **4l** (Scheme 2b). Thus, the regioselectivity obviously depends on the size of two groups of the alkyne: the larger group of the alkyne was more likely to stay away from the phenyl group originating from **3a**. The failure of the reaction of **3a-OH** with **4a** under the standard conditions indicated that the reaction may not proceed the [2 + 2 + 2] cycloaddition of the diyne unit in **3a** with the C–C triple bond in **4a** (Scheme 2c).

**Scheme 3:**
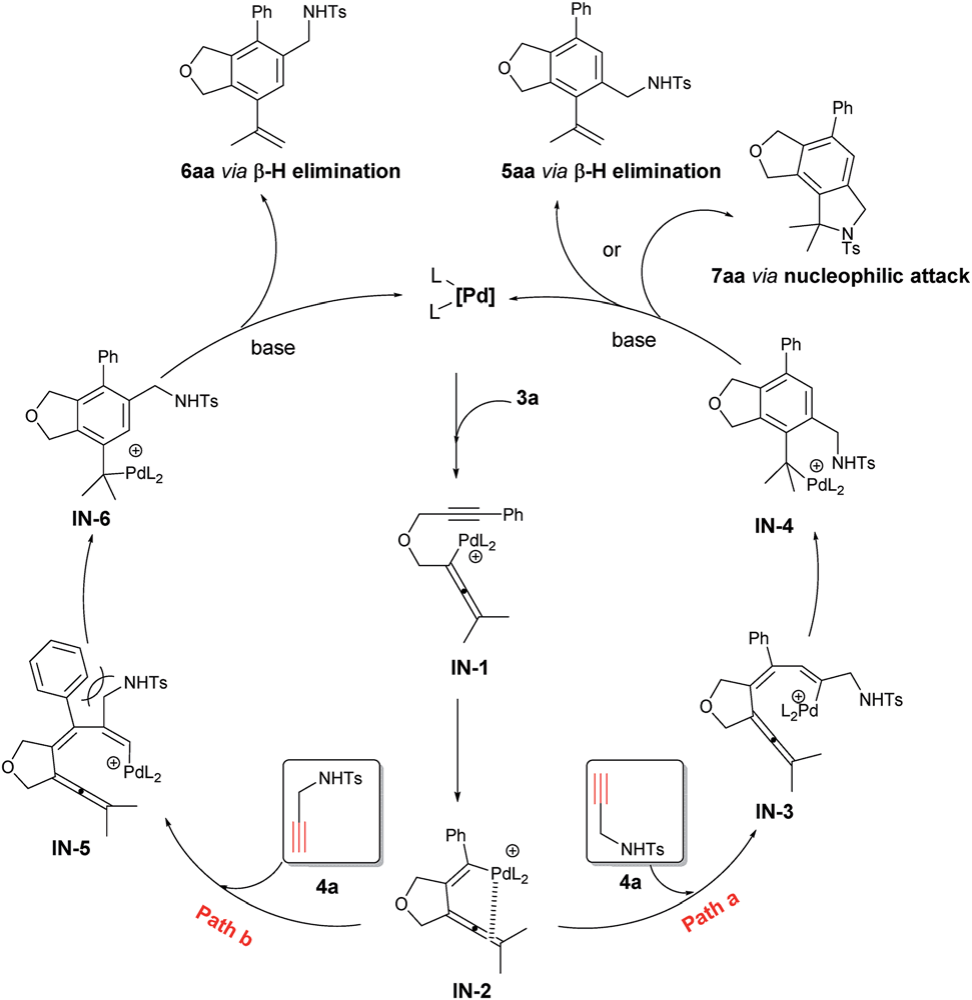
The proposed mechanistic pathways.

Based on these experimental results, a possible mechanism is shown in Scheme 3 by taking the reaction of **3a** and **4a** as an example: oxidative addition of **3a** with the catalytically active species Pd(0) would give the allenylpalladium intermediate **IN-1**,^22^ which undergoes intramolecular exo-mode insertion of the C–C triple bond to generate the alkenylpalladium species **IN-2**.^23^ The species **IN-2** would undergo intermolecular carbopalladation of the C≡C bond of **4a** to generate a new alkenylpalladium intermediate **IN-3** (path a) or **IN-5** (path b). Then intramolecular carbopalladation of allene would form a new benzylpalladium intermediate **IN-4** or **IN-6**. In the presence of a base, the key intermediate **IN-4** could undergo the intramolecular nucleophilic attack or β-H elimination forming tricyclic product **7aa** or the phthalan derivative **5aa** and regenerating the catalytically active Pd(0). As a comparison, the key intermediate **IN-6** could only go through the β-H elimination to deliver the phthalan derivative isomer **6aa**. Obviously, there is a strong steric interaction between the phenyl group and –CH_2_NHTs moiety in **IN-5**, which make the reaction more likely to go through path a.

**Scheme 4:**
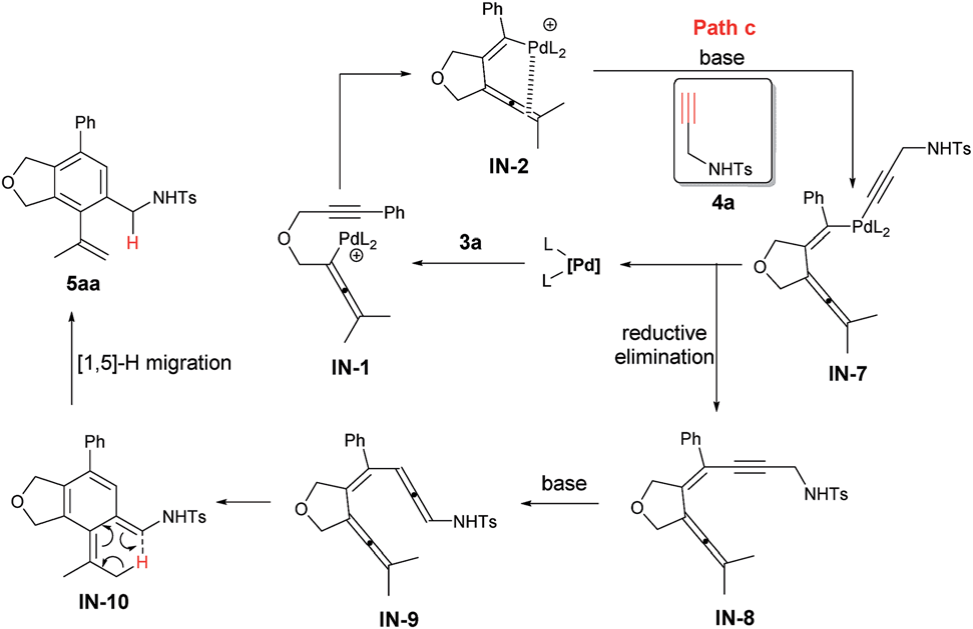
An alternative mechanism involving a Sonogashira coupling of **IN-2**.

An alternative mechanism involving a Sonogashira coupling (path c) of **IN-2** has been presented in Scheme 4. The resulted intermediate **IN-8** could go through the Garratt–Braverman cyclization to deliver **5aa**.^24^

There should be a possibility of [1,5]-H migration process in path c. Thus, the reaction of **3a-d_6_** and **4a** was conducted and a mixture of **5aa-d_5_** and **6aa-d_5_** with a ratio 98/2 with no deuteration at the α-position of the NHTs group was afforded in 85% yield (eqn (4)), indicating that there is no H-migration process, thus, path c is not viable for the formation of **5aa**.
4

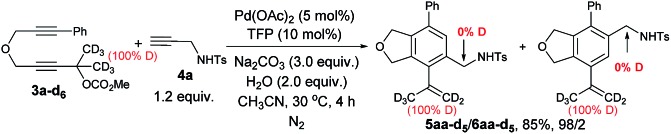




**Scheme 5:**
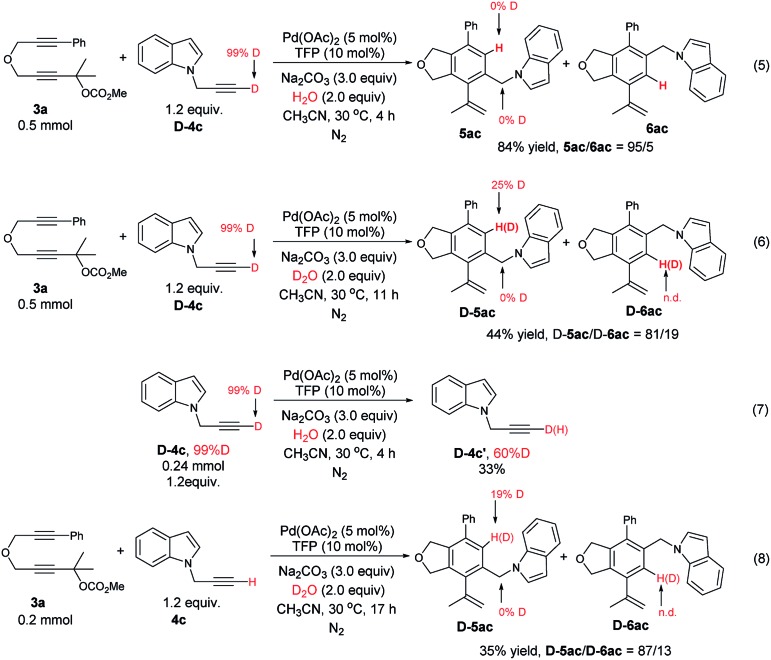
Deuterium labeling experiments: investigating of the D–H exchange of the terminal alkyne (n.d. = not able to be determined by ^1^H NMR analysis).

We also performed some deuterium labelling experiments for the investigation of the D–H exchange of the terminal alkyne (Scheme 5). To our surprise, the reaction of **3a** with **D-4c** under the standard conditions afforded **5ac**/**6ac** in 84% yield with a selectivity of 95/5 without any deuterium incorporation (eqn (5)). We conjectured that the deuterium atom may be easily exchanged with the hydrogen atom under the aqueous environment. Thus, H_2_O (2.0 equiv.) was replaced with D_2_O (2.0 equiv.), which led to the formation of 25% deuterium incorporation in **D-5ac** (eqn (6)). Interestingly, the regioselectivity dropped from 95 : 5 to 81 : 19 (compare eqn (5) with eqn (6)), which might be explained by the steric effect of D *vs.* H at 30 °C.^25^ Of course, further attention is obviously needed. The D–H exchange was also proven by the reaction of **D-4c** with H_2_O or the reaction of **4c** with **3a** in the presence of D_2_O (eqn (7) and (8)). These experimental facts further support the mechanism shown in Scheme 3.

**Scheme 6:**
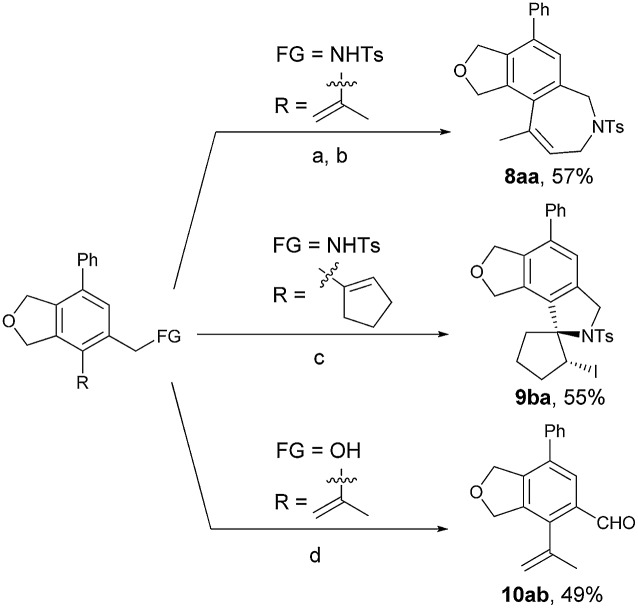
Synthetic applications. Reaction condition: (a) allylbromide (2.0 equiv.), K_2_CO_3_ (4.0 equiv.), CH_3_CN, refluxed (85 °C), 4 h; (b) Grubbs' II catalyst (10 mol%), toluene, 80 °C, 26 h; (c) NIS (1.5 equiv.), CH_3_CN/H_2_O = 15/1, rt, 27 h. (d) Fe(NO_3_)_3_·9H_2_O (10 mol%), TEMPO (10 mol%), NaCl (5 mol%), CH_2_Cl_2_, rt, 17.5 h.

In order to show the potential of the products, some synthetic applications have been conducted (Scheme 6). The bicyclic product **5aa** could be transferred to tricyclic isobenzofuro[5,4-*c*]azepine derivative **8aa** in 57% yield after an allylation-RCM process.^26^ The tetracyclic product **9ba** containing a spirocycle skeleton can be easily obtained through an electrophilic cyclization with NIS (1.5 equiv.).^27^ The Fe(NO_3_)_3_·9H_2_O–TEMPO–NaCl–catalyzed oxidation of **5ab** proceeded smoothly to give the aryl aldehyde **10ab** in 49% yield.^28^

## Conclusions

In summary, we have developed a highly regio- and chemo-selective annulation of 2,7-alkadiynylic carbonates in the presence of functionalized alkynes to construct 1,3-dihydroisobenzofuran and isoindoline derivatives under mild conditions. Functional groups such as sulfonamide, alcohol, and indoles could be kept untouched, which provides a chance for many further transformations to more complicated polycycles. Further studies in this area are being pursued in our laboratory.

## Conflicts of interest

There are no conflicts to declare.

## Supplementary Material

Supplementary information

Crystal structure data
