# Supplementary material for: Benzene construction via Pd-catalyzed cyclization of 2,7-alkadiynylic carbonates in the presence of alkynes
Source: Chem Sci. 2018 Dec 19;10(7):2228–35. doi: 10.1039/c8sc04681f (PMC6385558; doi:10.1039/c8sc04681f)
Supplement: Supplementary file 1 [file SC-010-C8SC04681F-s001.pdf]

Electronic Supplementary Material (ESI) for Chemical Science.
This journal is © The Royal Society of Chemistry 2019

Benzene Construction via Pd-catalyzed Cyclization of 2,7-Alkadiynylic Carbonates in the Presence of Alkynes

Yuchen Zhang, Wangteng Wu, Chunling Fu, Xin Huang* and Shengming Ma*

Laboratory of Molecular Recognition and Synthesis, Department of Chemistry,Zhejiang University, Hangzhou 310027, Zhejiang, People's Republic of China.

Fax: (+86)21-64167510

E-mail: xinhuangzju@zju.edu.cn; masm@sioc.ac.cn

Supporting Information

General information	S2
Synthesis of new starting materials	S3-S6
Synthesis of products **5aa/6aa~5la/6la**.	S7-S29
Control experiments	S30-S38
Deuterium labelling experiments	S39-S43
Synthetic applications	S44-S48
References	S49
Copies of the ^{1}H NMR and ^{13}C NMR spectra	S50-S140

General Information

^{1}H NMR and ^{13}C NMR spectra were recorded in $CDCl_3$ using a Bruker AM 300 MHz NMR spectrometer (^{1}H at 300 MHz, ^{13}C at 75 MHz). IR spectra were recorded with a Perkin–Elmer 983G instrument. Elemental analyses were measured with a Carlo-Erba EA1110 elementary analysis instrument. Mass spectrometry was performed with an HP 5989A system. High-resolution mass spectrometry was determined with a Finnigan MAT 8430 or Bruker APEXIII instrument. $Pd(OAc)_2$ was purchased from *Adamas*. TFP was purchased from *J&K*. Na_2CO_3 was bought from *Hongguang Chemical Reagent Co., Ltd*. CH_3CN was refluxed over CaH_2 and distilled right before use. Unless otherwise indicated, chemicals and solvents were purchased from commercial suppliers. All the temperatures are referred to the oil baths used. 2,7-Alkadiynylic carbonates were prepared according to our previous literatures.[1,2]

Synthesis of starting material 3j.[1] (wwt-2-35)

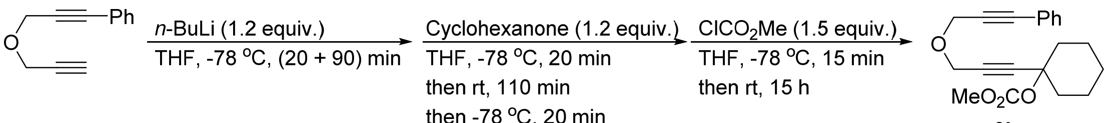

3j
43 %

To a solution of 3-(propargyloxy)phenylpropyne (5086.4 mg, 30 mmol) in THF (120 mL) was added dropwise *n*-BuLi (2.5 M in hexane, 14.4 mL, 36 mmol) at -78 °C within 20 minutes. After lithiation for 90 minutes at -78 °C, cyclohexanone (3.7 mL, d = 0.95 g/mL, 3515.0 mg, 36 mmol) was added dropwise with an addition funnel at -78 °C within 20 minutes. The cooling bath was removed and the reaction mixture was warmed up to room temperature and stirred for 110 minutes. Then the mixture was cooled down to -78 °C again for 20 minutes. Methyl chloroformate (3.5 mL, d = 1.22 g/mL, 4270.0 mg, 45 mmol) was added dropwise at -78 °C within 15 minutes. The cooling bath was removed and the reaction mixture was warmed up to room temperature and stirred for 15 h. After the reaction was complete as monitored by TLC (eluent: petroleum ether/ethyl acetate = 20/1), it was quenched with a saturated aqueous solution of NH_4Cl (50 mL). The resulting mixture was extracted with ethyl acetate (30 mL × 3) and the combined organic phase was dried over anhydrous Na_2SO_4. After filtration and evaporation of the solvent, the crude residue was purified by chromatography on silica gel (eluent: petroleum ether/ethyl acetate = 50/1 (3570 mL) ot 10/1 (1100 mL)) to afford **3j** (4165.2 mg, 43%) as a liquid: ^{1}H NMR (300 MHz, $CDCl_3$) δ 7.50-7.39 (m, 2 H, ArH), 7.35-7.27 (m, 3 H, ArH), 4.49 (s, 2 H, OCH_2), 4.40 (s, 2 H, OCH_2), 3.76 (s, 3 H, OCH_3), 2.24-2.10 (m, 2 H, CH_2), 1.95-1.80 (m, 2 H, CH_2), 1.75-1.46 (m, 5 H, CH_2 × 2 and one proton of CH_2), 1.41-1.23 (m, 1 H,

one proton of CH_2); ^{13}C NMR (75 MHz, $CDCl_3$) δ 153.3, 131.8, 128.5, 128.3, 122.5, 86.6, 86.3, 84.3, 81.9, 77.6, 57.1, 56.8, 54.3, 36.8, 24.9, 22.6; IR (neat) ν (cm^{-1}) 2938, 2859, 2236, 2197, 1752, 1490, 1441, 1349, 1274, 1247, 1217, 1181, 1130, 1082, 1016; MS (EI): *m/z* (%) 326 (M^+, 1.79), 115 (100); HRMS calcd. for $C_{20}H_{22}O_4$ [M^+]: 326.1518; Found: 326.1521.

Synthesis of starting material 3l.[1] (zyc-3-26)

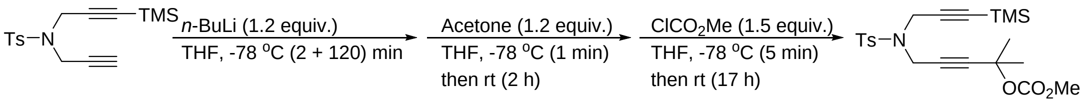

3l
25 %

To a solution of *N*-(prop-2-ynyl)-*N*-(3-(trimethylsilyl)prop-2-ynyl)-*p*-tolylsulfonamide (1596.2 mg, 5 mmol) in THF (12 mL) was added dropwise *n*-BuLi (2.5 M in hexane, 2.4 mL, 6 mmol) at -78 °C within 2 minutes. After lithiation for 120 minutes at -78 °C, acetone (0.45 mL, d = 0.788 g/mL, 354.6 mg, 6.1 mmol) was added dropwise with an addition funnel at -78 °C within 1 minutes. The cooling bath was removed and the reaction mixture was warmed up to room temperature and stirred for 2 hours. Then methyl chloroformate (0.58 mL, d = 1.22 g/mL, 707.6 mg, 7.5 mmol) was added dropwise at -78 °C within 5 minutes. The cooling bath was removed and the reaction mixture was warmed up to room temperature and stirred for 17 h. After the reaction was complete as monitored by TLC (eluent: petroleum ether/ethyl acetate = 20/1), it was quenched with a saturated aqueous solution of NH_4Cl (10 mL). The resulting mixture was extracted with ethyl acetate (10 mL × 3) and the combined organic phase was dried over anhydrous Na_2SO_4. After filtration and evaporation of

the solvent, the crude residue was purified by chromatography on silica gel (eluent: petroleum ether/ethyl acetate/diethyl ether = 20/1/1) to afford **3l** (560.2 mg, purity = 96%, 25%) as a liquid: ^{1}H NMR (300 MHz, $CDCl_3$) δ 7.70 (d, *J* = 8.4 Hz, 2 H, ArH), 7.30 (d, *J* = 8.1 Hz, 2 H, ArH), 4.18 (s, 4 H, OCH_2 × 2), 3.73 (s, 3 H, OCH_3), 2.42 (s, 3 H, CH_3), 1.53 (s, 6 H, CH_3 × 2), 0.05 (s, 9 H, CH_3 × 3); ^{13}C NMR (75 MHz, $CDCl_3$) δ 153.2, 143.6, 135.0, 129.4, 127.7, 97.4, 90.7, 86.0, 77.1, 73.5, 54.1, 37.0, 36.3, 28.3, 21.3, -0.6; IR (neat) ν (cm^{-1}) 2990, 2958, 2900, 2180, 1755, 1598, 1495, 1441, 1354, 1278, 1194, 1165, 1138, 1096, 1045, 1003; MS (EI): *m/z* (%) 435 (M^+, 3.89), 73 (100); HRMS calcd. for $C_{21}H_{29}NO_5SSi$ [M^+]: 435.1536; Found: 435.1539.

Synthesis of starting material 4f.[3] (zyc-1-124)

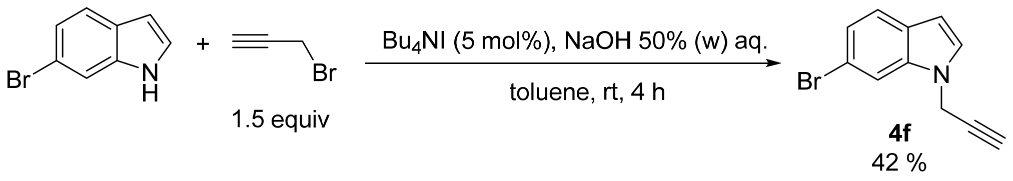

To a dry flask were added 6-bromoindole (1000.8 mg, 5 mmol)/toluene (4 mL), propargyl bromide (0.6 mL, d = 1.52 g/mL, 912.0 mg, 7.7 mmol)/toluene (6 mL), Bu_4NI (92.9 mg, 0.25 mmol), and an aqueous solution of NaOH (2492.4 mg in 2.5 mL of H_2O) sequentially. The reaction was complete after being stirred at room temperature for 4 hours as monitored by TLC. The resulting mixture was transferred to a separation funnel. Water (5 mL) and ethyl acetate (10 mL) were then added. After the separation of the organic phase, the aqueous phase was extracted with ethyl acetate (10 mL × 2). The combined organic phase was washed with brine (10 mL) and dried over anhydrous Na_2SO_4. After filtration and evaporation of the solvent, the

residue was purified by column chromatography on silica gel (eluent: petroleum ether (60-90 °C)/ethyl acetate = 150/1) to afford impure **4f** (985.6 mg), which was further purified by recrystallization (*n*-hexane/DCM) to afford pure **4f** (493.9 mg, 42%) as a solid: m.p. 51.8-52.7 °C (*n*-hexane/DCM); ^{1}H NMR (300 MHz, $CDCl_3$) δ 7.54 (t, J = 0.9 Hz, 1 H, ArH), 7.46 (dd, J_1 = 8.3 Hz, J_2 = 0.5 Hz, 1 H, ArH), 7.22 (dd, J_1 = 8.4 Hz, J_2 = 1.5 Hz, 1 H, ArH), 7.14 (d, J = 3.3 Hz, 1 H, ArH), 6.48 (dd, J_1 = 3.3 Hz, J_2 = 0.9 Hz, 1 H, ArH), 4.77 (d, J = 2.4 Hz, 2 H, NCH_2), 2.40 (t, J = 2.6 Hz, 1 H, ≡CH); ^{13}C NMR (75 MHz, $CDCl_3$) δ 136.5, 127.9, 127.7, 123.1, 122.3, 115.5, 112.4, 102.3, 77.1, 73.9, 35.8; IR (KBr) ν (cm^{-1}) 3270, 3123, 3102, 2932, 2117, 1869, 1724, 1700, 1695, 1685, 1601, 1560, 1504, 1462, 1425, 1418, 1391, 1343, 1334, 1317, 1308, 1247, 1191, 1100, 1057, 1042; MS (EI): *m/z* (%) 235 ($(M(^{81}Br)^+$, 32.16), 233 ($M(^{79}Br)^+$, 31.09), 154 (100); Anal. Calcd. for $C_{11}H_8BrN$ (%): C 56.44, H 3.44, N 5.98, Found: C 56.28, H 3.47, N 5.98.

Synthesis of products 5aa/6aa~5ia/6ia.

1. Synthesis of *N*-((7-phenyl-4-(propen-2-yl)-1,3-dihydroisobenzofuran-5-yl)methyl)-4-methylbenzenesulfonamide (**5aa**). (wwt-1-193)

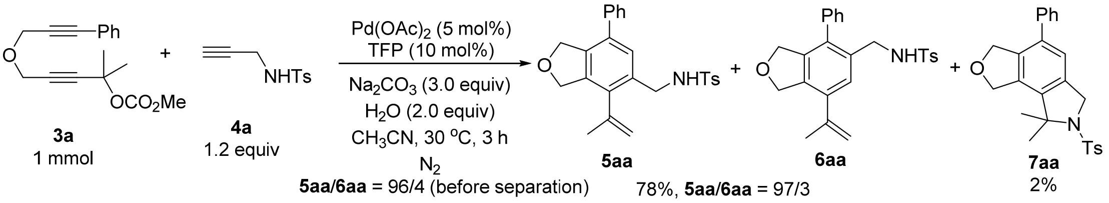

Typical Procedure I: To a flame-dried Schlenk tube containing Na_2CO_3 (318.6 mg, 3.0 mmol) were added $Pd(OAc)_2$ (11.1 mg, 0.05 mmol), TFP (23.3 mg, 0.1 mmol), **4a** (250.9 mg, 1.2 mmol)/CH_3CN (2.0 mL), **3a** (286.7 mg, 1.0 mmol)/CH_3CN (6.0 mL), and H_2O (35.6 mg, 2.0 mmol)/CH_3CN (2.0 mL) sequentially under nitrogen atmosphere. The reaction was complete after being stirred at 30 °C for 3 hours as monitored by TLC. The resulting mixture was filtrated through a short column of silica gel and eluted with ethyl acetate (30 mL × 3). After evaporation, the crude residue (**5aa**/**6aa** = 96/4 as determined by ^{1}H NMR analysis of the crude product using mesitylene (46 μL) as the internal standard) was purified by chromatography on silica gel (eluent: petroleum ether (60-90 °C)/ethyl acetate = 5/1 (360 mL) to petroleum ether (60-90 °C)/ethyl acetate/DCM = 4/1/1 (600 mL)) to afford **7aa** (6.7 mg, 2%) and **5aa**/**6aa** (326.7 mg, 78%, **5aa**/**6aa** = 97/3 as determined by ^{1}H NMR analysis of the isolated product).

5aa/**6aa** (97/3): solid; ^{1}H NMR (300 MHz, $CDCl_3$) δ 7.73 (d, *J* = 8.1 Hz, 2 H, ArH), 7.48-7.30 (m, 3 H, ArH), 7.30-7.20 (m, 4 H, ArH), 7.15 (s, 1 H, ArH), 5.26-5.09 (m, 3 H, one proton of =CH_2 and OCH_2), 5.08-4.87 (m, 3 H, OCH_2 and NH), 4.85-4.77 (m, 1 H, one proton of =CH_2), 4.15 (d, *J* = 6.0 Hz, 2 H, NCH_2), 2.39

(s, 3 H, CH_3), 1.89 (s, 3 H, CH_3); ^{13}C NMR (75 MHz, $CDCl_3$) δ 143.4, 141.9, 139.3, 138.3, 136.8, 136.7, 136.0, 134.7, 132.9, 129.6, 128.6, 128.5, 127.6, 127.5, 127.1, 116.6, 73.7, 72.9, 44.3, 23.4, 21.4; IR (KBr) ν (cm^{-1}) 3277, 3061, 2916, 2856, 1598, 1477, 1446, 1327, 1290, 1159, 1093, 1058; MS (EI): *m/z* (%) 419 (M^+, 0.09), 264 ($(M\text{-}Ts)^+$, 100); Anal. Calcd. for $C_{25}H_{25}NO_3S$ (%): C 71.57, H 6.01, N 3.34; Found: C 71.53, H 6.07, N 3.17. The following signals are discernible for **6aa**: ^{1}H NMR (300 MHz, $CDCl_3$) δ 4.77-4.72 (m, 1 H, OCH_2), 3.98 (d, J = 6.3 Hz, 2 H, NCH_2), 2.06 (s, 3 H, CH_3).

This mixture may further be purified by recrystallization to afford the pure product **5aa**: m.p. 152.4-154.5 °C (*n*-hexane/DCM).

7aa: liquid; ^{1}H NMR (300 MHz, $CDCl_3$) δ 7.83 (d, J = 8.4 Hz, 2 H, ArH), 7.49-7.27 (m, 7 H, ArH), 7.10 (s, 1 H, ArH), 5.19 (s, 2 H, OCH_2), 5.11 (s, 2 H, OCH_2), 4.68 (s, 2 H, NCH_2), 2.41 (s, 3 H, CH_3), 1.78 (s, 6 H, $CH_3 \times 2$); ^{13}C NMR (75 MHz, $CDCl_3$) δ 143.2, 139.6, 139.2, 138.1, 137.9, 135.8, 133.8, 133.0, 129.5, 128.7, 127.8, 127.7, 127.4, 121.8, 72.8, 71.2, 70.6, 53.1, 27.1, 21.5; IR (neat) ν (cm^{-1}) 2971, 2923, 2854, 1763, 1598, 1495, 1467, 1409, 1339, 1273, 1162, 1149, 1135, 1093, 1058, 1005; MS (EI): *m/z* (%) 419 (M^+, 0.1), 404 ($(M\text{-}CH_3)^+$, 100); HRMS calcd. for $C_{24}H_{22}NO_3S$ [$(M\text{-}CH_3)^+$]: 404.1320; Found: 404.1321.

Gram-scale synthesis of 5aa. (zyc-1-166)

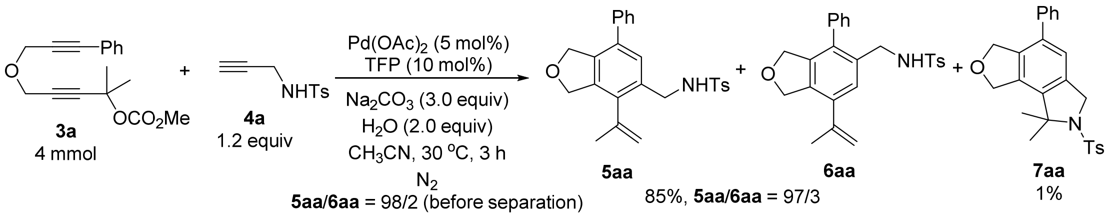

Following **Typical Procedure I**, the reaction of **3a** (1144.6 mg, 4.0 mmol), **4a** (1004.7 mg, 4.8 mmol), $Pd(OAc)_2$ (44.5 mg, 0.2 mmol), TFP (93.4 mg, 0.4 mmol), Na_2CO_3 (1272.2 mg, 12.0 mmol), and H_2O (144.4 mg, 8.0 mmol) in CH_3CN (40 mL) afforded **7aa** (20.5 mg, 1%) and **5aa**/**6aa** (1417.3 mg, 85%, **5aa**/**6aa** = 97/3 as determined by ^{1}H NMR analysis of the isolated product) (eluent: petroleum ether (60-90 ^{o}C)/ethyl acetate/DCM = 10/1/1 (2040 mL) to 5/1/1 (210 mL)) (**5aa**/**6aa** = 98/2 as determined by ^{1}H NMR analysis of the crude product using mesitylene (184 μL) as the internal standard).

5aa/**6aa** (97/3): ^{1}H NMR (300 MHz, $CDCl_3$) δ 7.72 (d, J = 8.1 Hz, 2 H, ArH), 7.44-7.18 (m, 7 H, ArH), 7.15 (s, 1 H, ArH), 5.25-5.16 (m, 1 H, one proton of =CH_2), 5.26-5.06 (m, 3 H, one proton of NH and OCH_2), 5.05-4.95 (m, 2 H, OCH_2), 4.84-4.76 (m, 1 H, one proton of =CH_2), 4.15 (d, J = 6.0 Hz, 2 H, NCH_2), 2.37 (s, 3 H, CH_3), 1.89 (s, 3 H, CH_3); ^{13}C NMR (75 MHz, $CDCl_3$) δ 143.4, 141.9, 139.3, 138.2, 136.9, 136.6, 136.0, 134.7, 132.9, 129.6, 128.6, 128.4, 127.6, 127.5, 127.1, 116.5, 73.6, 72.9, 44.2, 23.4, 21.4. The following signals are discernible for **6aa**: ^{1}H NMR (300 MHz, $CDCl_3$) δ 4.75-4.72 (m, 1 H, one proton of =CH_2), 3.97 (d, J = 6.0 Hz, 2 H, NCH_2), 2.04 (s, 3 H, CH_3).

7aa: liquid; ^{1}H NMR (300 MHz, $CDCl_3$) δ 7.83 (d, J = 8.1 Hz, 2 H, ArH), 7.47-7.25 (m, 7 H, ArH), 7.10 (s, 1 H, ArH), 5.19 (s, 2 H, OCH_2), 5.11 (s, 2 H, OCH_2), 4.68 (s, 2 H, NCH_2), 2.41 (s, 3 H, CH_3), 1.78 (s, 6 H, CH_3 × 2); ^{13}C NMR (75 MHz, $CDCl_3$) δ 143.2, 139.6, 139.2, 138.0, 137.8, 135.8, 133.8, 132.9, 129.5, 128.7, 127.8, 127.7, 127.3, 121.8, 72.8, 71.1, 70.6, 53.1, 27.1, 21.5.

The following compounds **5ab**/**6ab** ~ **5ia**/**6ia** were prepared according to **Typical Procedure I**.

2. Synthesis of (7-phenyl-4-(propen-2-yl)-1,3-dihydroisobenzofuran-5-yl)methanol (**5ab**). (wwt-1-189, wwt-2-90)

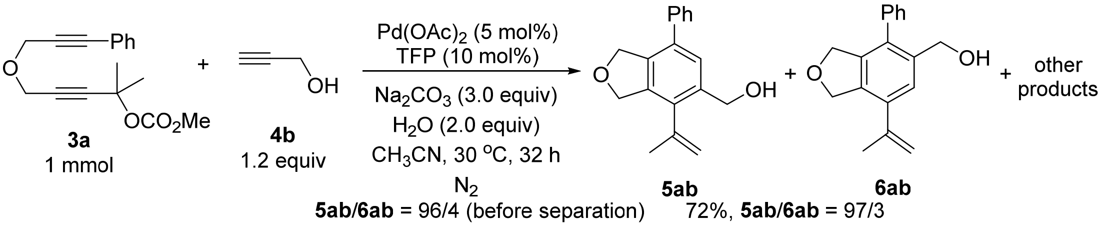

The reaction of Na_2CO_3 (316.4 mg, 3.0 mmol), $Pd(OAc)_2$ (11.3 mg, 0.05 mmol), TFP (23.1 mg, 0.1 mmol), **4b** (67.3 mg, 1.2 mmol), **3a** (285.4 mg, 1.0 mmol), and H_2O (36.2 mg, 2.0 mmol) in CH_3CN (10 mL) afforded **5ab**/**6ab** (190.1 mg, 72%, **5ab**/**6ab** = 97/3 as determined by ^{1}H NMR analysis of the isolated product) (eluent: petroleum ether (60-90 ^{o}C)/ethyl acetate = 10/1 (330 mL) to 5/1 (480 mL)) (**5ab**/**6ab** = 96/4 as determined by ^{1}H NMR analysis of the crude product using mesitylene (46 μL) as the internal standard).

5ab/**6ab** (97/3): liquid; ^{1}H NMR (300 MHz, $CDCl_3$) δ 7.48-7.28 (m, 6 H, ArH), 5.29-5.23 (m, 1 H, one proton of =CH_2), 5.20-5.14 (m, 2 H, OCH_2), 5.09-5.02 (m, 2 H, OCH_2), 4.91-4.85 (m, 1 H, one proton of =CH_2), 4.68 (s, 2 H, OCH_2), 2.34 (br s, 1 H, OH), 2.00 (t, J = 1.2 Hz, 3 H, CH_3); ^{13}C NMR (75 MHz, $CDCl_3$) δ 142.4, 139.7, 137.9, 137.6, 136.2, 135.5, 134.6, 128.6, 127.7, 127.6, 127.4, 116.0, 73.7, 72.9, 62.4, 23.6; IR (neat) ν (cm^{-1}) 3416, 3076, 3029, 2938, 2911, 2855, 1640, 1601, 1474, 1446, 1370, 1329, 1287, 1195, 1136, 1057; GC-MS (GC condition: injector: 280 ^{o}C; column:

DB-5, temperature: 60 °C (2 min), 20 °C/min to 280 °C, 280 °C (30 min)) (EI) *m/z* (%) for **5ab**: T_R 5.3 min: 266 (M^+, 100); for **6ab**: T_R 5.2 min: 266 (M^+, 100); HRMS calcd. for $C_{18}H_{18}O_2$ [M^+]: 266.1307; Found: 266.1306. The following signals are discernible for **6ab**: ^{1}H NMR (300 MHz, $CDCl_3$) δ 5.23-5.20 (m, 1 H, one proton of $=CH_2$), 5.00-4.97 (m, 2 H, OCH_2), 4.84-4.80 (m, 2 H, OCH_2), 4.48 (s, 2 H, CH_2), 2.14-2.10 (m, 3 H, CH_3).

3. Synthesis of 1-((7-phenyl-4-(propen-2-yl)-1,3-dihydroisobenzofuran-5-yl)methyl)-1*H*-indole (**5ac**). (wwt-2-123, zyc-1-68)

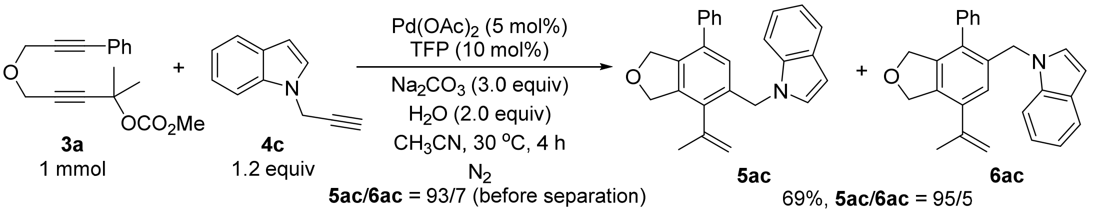

The reaction of Na_2CO_3 (317.6 mg, 3 mmol), $Pd(OAc)_2$ (11.3 mg, 0.05 mmol), TFP (23.1 mg, 0.1 mmol), **4c** (185.4 mg, 1.2 mmol), **3a** (285.4 mg, 1.0 mmol), and H_2O (36.2 mg, 2 mmol) in CH_3CN (10 mL) afforded **5ac**/**6ac** (252.9 mg, 69%, **5ac**/**6ac** = 95/5 as determined by ^{1}H NMR analysis of the isolated product) (eluent: petroleum ether (60-90 °C)/ethyl acetate = 40/1) (**5ac**/**6ac** = 93/7 as determined by ^{1}H NMR analysis of the crude product using mesitylene (46 μL) as the internal standard).

5ac/**6ac** (95/5): solid; ^{1}H NMR (300 MHz, $CDCl_3$) δ 7.66-7.57 (m, 1 H, ArH), 7.47-7.00 (m, 9 H, ArH), 6.93 (s, 1 H, ArH), 6.52-6.46 (m, 1 H, ArH), 5.34-5.26 (m, 3 H, one proton of $=CH_2$ and NCH_2), 5.21-5.15 (m, 2 H, OCH_2), 5.11-5.05 (m, 2 H, OCH_2), 4.99-4.93 (m, 1 H, one proton of $=CH_2$), 1.88 (s, 3 H, CH_3); ^{13}C NMR (75

MHz, $CDCl_3$) δ 142.1, 139.4, 138.4, 136.6, 136.3, 135.8, 134.7, 133.9, 128.6, 128.0, 127.6, 127.4, 127.3, 121.5, 120.9, 119.4, 116.3, 109.5, 101.7, 73.7, 72.9, 47.2, 23.1; GC-MS (GC condition: injector: 280 °C; column: DB-5, temperature: 60 °C (2 min), 20 °C/min to 280 °C, 280 °C (30 min)) (EI) *m/z* (%) for **5ac**: T_R 6.8 min: 365 (M^+, 71.0), 350 (100); for **6ac**: T_R 7.5 min: 365 (M^+, 31.1), 350 (100); HRMS calcd. for $C_{26}H_{23}NO$ [M^+]: 365.1780, Found: 365.1784. The following signals are discernible for **6ac**: ^1H NMR (300 MHz, $CDCl_3$) δ 6.46-6.42 (m, 1 H, ArH), 5.46-5.38 (m, 3 H, one proton of =CH_2 and NCH_2), 5.13 (s, 2 H, OCH_2).

This mixture may further be purified by recrystallization to afford the pure product **5ac**: m.p. 121.5-123.3 °C (*n*-hexane/DCM).

4. Synthesis of 5-methyl-1-((7-phenyl-4-(propen-2-yl)-1,3-dihydroisobenzofuran-5-yl)methyl)-1*H*-indole (**5ad**). (zyc-1-102)

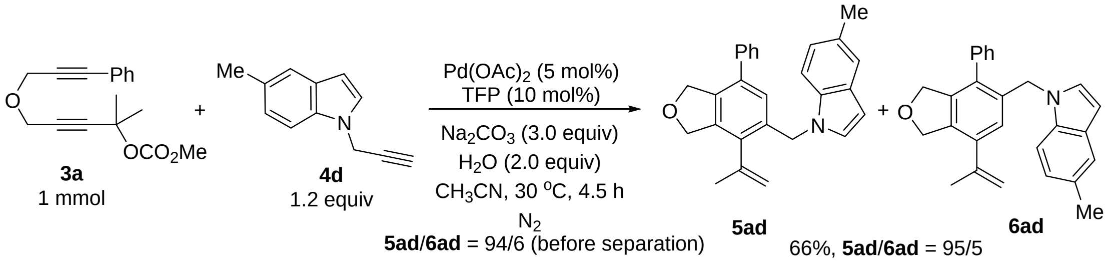

The reaction of Na_2CO_3 (317.9 mg, 3.0 mmol), $Pd(OAc)_2$ (11.8 mg, 0.05 mmol), TFP (24.1 mg, 0.1 mmol), **4d** (203.5 mg, 1.2 mmol), **3a** (285.7 mg, 1.0 mmol), and H_2O (37.5 mg, 2.1 mmol) in CH_3CN (10 mL) afforded **5ad**/**6ad** (249.9 mg, 66%, **5ad**/**6ad** = 95/5 as determined by ^1H NMR analysis of the isolated product) (eluent: petroleum ether (60-90 °C)/ethyl acetate = 30/1) (**5ad**/**6ad** = 94/6 as determined by ^1H NMR analysis of the crude product using mesitylene (46 μL) as the internal standard).

5ad/6ad (95/5): solid; ^{1}H NMR (300 MHz, $CDCl_3$) δ 7.46-7.10 (m, 7 H, ArH), 7.07-6.88 (m, 3 H, ArH), 6.42 (d, *J* = 3.3 Hz, 1 H, ArH), 5.37-5.24 (m, 3 H, one proton of $=CH_2$ and NCH_2), 5.22-5.16 (m, 2 H, OCH_2), 5.12-5.05 (m, 2 H, OCH_2), 5.01-4.93 (m, 1 H, one proton of $=CH_2$), 2.42 (s, 3 H, CH_3), 1.89 (s, 3 H, CH_3); ^{13}C NMR (75 MHz, $CDCl_3$) δ 142.2, 139.5, 138.4, 136.6, 135.8, 134.8, 134.1, 128.9, 128.6, 128.1, 127.7, 127.45, 127.37, 123.2, 120.6, 116.3, 109.2, 101.1, 73.8, 73.0, 47.3, 23.2, 21.3; IR (KBr) ν (cm^{-1}) 3073, 3028, 2965, 2909, 2832, 1508, 1485, 1474, 1437, 1390, 1371, 1345, 1333, 1301, 1256, 1227, 1180, 1132, 1059, 1029; MS (EI): *m/z* (%) 379 (M^+, 77.0), 364 ($(M-CH_3)^+$, 100.0); HRMS calcd. for $C_{27}H_{25}NO$ [M^+]: 379.1936, Found: 379.1937. The following signals are discernible for **6ad**: ^{1}H NMR (300 MHz, $CDCl_3$) δ 6.36 (s, 1 H, ArH), 5.44-5.41 (m, 3 H, NCH_2 and one proton of $=CH_2$), 2.45 (s, 3 H, CH_3).

This mixture may further be purified by recrystallization to afford the pure product **5ad**: m.p. 119.6-122.8 °C (DCM).

5. Synthesis of 1-((7-phenyl-4-(propen-2-yl)-1,3-dihydroisobenzofuran-5-yl)methyl)-1*H*-indole-3-carbaldehyde (**5ae**). (zyc-1-79)

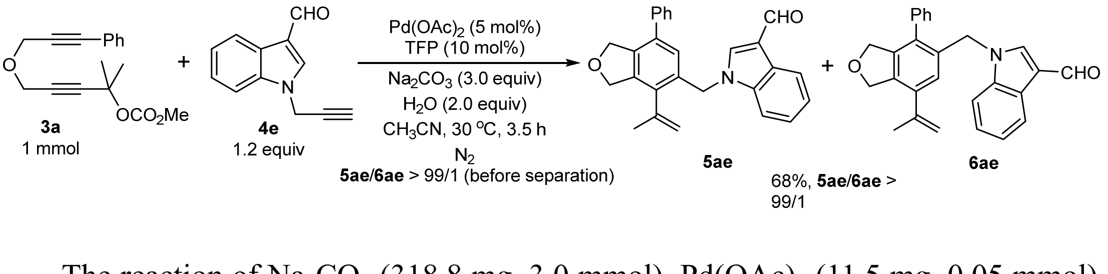

The reaction of Na_2CO_3 (318.8 mg, 3.0 mmol), $Pd(OAc)_2$ (11.5 mg, 0.05 mmol), TFP (24.2 mg, 0.1 mmol), **4e** (220.2 mg, 1.2 mmol), **3a** (285.9 mg, 1.0 mmol), and

H_2O (37.0 mg, 2.0 mmol) in CH_3CN (10 mL) afforded **5ae** (268.9 mg, 68%, **5ae**/**6ae** > 99/1 as determined by ^1H NMR analysis of the isolated product) (eluent: petroleum ether (60-90 ^oC)/ethyl acetate = 10/1 (880 mL) to 5/1 (1000 mL)) (**5ae**/**6ae** > 99/1 as determined by ^1H NMR analysis of the crude product using mesitylene (46 μL) as the internal standard).

5ae: solid; m.p. 158.8-160.4 ^oC (*n*-hexane/DCM); ^1H NMR (300 MHz, $CDCl_3$) δ 9.97 (s, 1 H, CHO), 8.35-8.26 (m, 1 H, ArH), 7.64 (s, 1 H, ArH), 7.45-7.22 (m, 8 H, ArH), 7.07 (s, 1 H, ArH), 5.38 (s, 2 H, NCH_2), 5.34-5.28 (m, 1 H, one proton of $=CH_2$), 5.25-5.19 (m, 2 H, OCH_2), 5.13-5.06 (m, 2 H, OCH_2), 4.98-4.91 (m, 1 H, one proton of $=CH_2$), 1.87 (s, 3 H, CH_3); ^{13}C NMR (75 MHz, $CDCl_3$) δ 184.5, 141.7, 139.00, 138.96, 138.3, 137.7, 137.4, 136.2, 135.1, 131.6, 128.7, 127.9, 127.7, 127.6, 125.3, 124.0, 123.0, 122.0, 118.4, 116.7, 110.2, 73.7, 72.9, 48.0, 23.1; IR (KBr) ν (cm^{-1}) 3111, 3080, 3059, 3019, 2962, 2938, 2911, 2836, 1659, 1615, 1577, 1531, 1466, 1435, 1406, 1388, 1352, 1259, 1157, 1136, 1057, 1046, 1013; MS (EI): *m/z* (%) 393 (M^+, 100.0); HRMS calcd. for $C_{27}H_{23}NO_2$ [M^+]: 393.1729, Found: 393.1728.

6. Synthesis of 6-bromo-1-((7-phenyl-4-(propen-2-yl)-1,3-dihydroisobenzofuran-5-yl) methyl)-1*H*-indole (**5af**). (zyc-1-130)

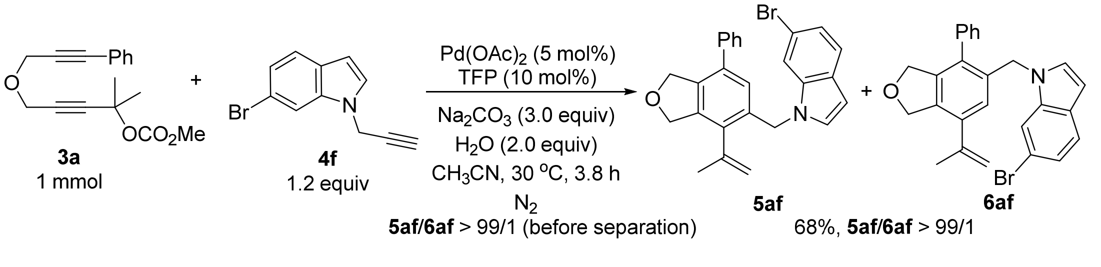

The reaction of Na_2CO_3 (317.5 mg, 3.0 mmol), $Pd(OAc)_2$ (11.6 mg, 0.05 mmol),

TFP (23.9 mg, 0.1 mmol), **4f** (281.7 mg, 1.2 mmol), **3a** (286.0 mg, 1.0 mmol), and H_2O (37.2 mg, 2.0 mmol) in CH_3CN (10 mL) afforded **5af** (304.3 mg, 68%, **5af**/**6af** > 99/1 as determined by ^1H NMR analysis of the isolated product) (The crude product was purified by column chromatography on silica gel (petroleum ether (60-90 ^oC)/ethyl acetate = 40/1) to afford impure **5af** (365.9 mg), which was further purified with chromatography (petroleum ether (60-90 ^oC)/DCM = 1/1)) (**5af**/**6af** > 99/1 as determined by ^1H NMR analysis of the crude product using mesitylene (46 μL) as the internal standard).

5af: solid; m.p. 120.6-122.7 ^oC (DCM); ^1H NMR (300 MHz, $CDCl_3$) δ 7.55-7.43 (m, 2 H, ArH), 7.42-7.22 (m, 5 H, ArH), 7.19 (dd, J_1 = 8.4 Hz, J_2 = 1.8 Hz, 1 H, ArH), 7.03 (d, J = 3.3 Hz, 1 H, ArH), 6.91 (s, 1 H, ArH), 6.48 (d, J = 3.3 Hz, 1 H, ArH), 5.36-5.31 (m, 1 H, one proton of $=CH_2$), 5.28 (s, 2 H, NCH_2), 5.24-5.17 (m, 2 H, OCH_2), 5.14-5.06 (m, 2 H, OCH_2), 5.02-4.95 (m, 1 H, one proton of $=CH_2$), 1.88 (s, 3 H, CH_3); ^{13}C NMR (75 MHz, $CDCl_3$) δ 142.0, 139.4, 138.6, 137.1, 136.9, 135.8, 134.9, 133.4, 128.7, 128.6, 127.7, 127.5, 127.4, 127.3, 122.8, 122.1, 116.5, 115.2, 112.6, 102.0, 73.7, 73.0, 47.3, 23.2; IR (KBr) ν (cm^{-1}) 3072, 3025, 2964, 2938, 2898, 2854, 1637, 1602, 1561, 1504, 1461, 1439, 1391, 1366, 1332, 1319, 1241, 1205, 1187, 1130, 1094, 1057, 1043, 1031; MS (EI): *m/z* (%) 443 ($(M(^{81}Br)^+$, 79.0), 445 ($M(^{79}Br)^+$, 80.5), 428 (100); Anal. Calcd. for $C_{26}H_{22}BrNO$ (%): C 70.28, H 4.99, N 3.15; Found: C 69.98, H 5.12, N 2.95.

7. Synthesis of N-((4-(cyclopentenyl)-7-phenyl-1,3-dihydroisobenzofuran-5-yl)meth-

yl)-4-methylbenzenesulfonamide (**5ba**). (wwt-2-130)

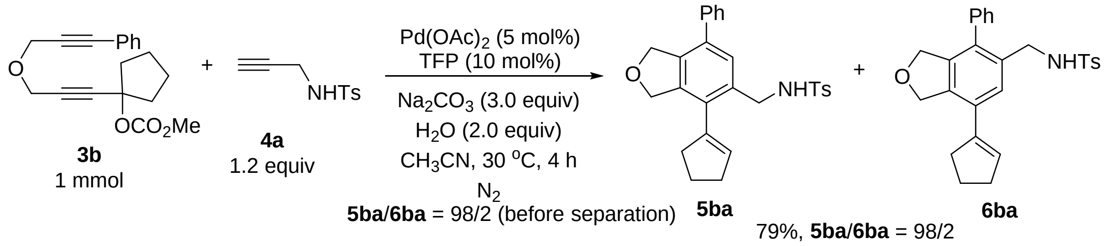

The reaction of Na_2CO_3 (318.2 mg, 3.0 mmol), $Pd(OAc)_2$ (11.3 mg, 0.05 mmol), TFP (23.4 mg, 0.1 mmol), **4a** (251.1 mg, 1.2 mmol), **3b** (311.7 mg, 1.0 mmol), and H_2O (36.0 mg, 2.0 mmol) in CH_3CN (10 mL) afforded **5ba**/**6ba** (351.0 mg, 79%, **5ba**/**6ba** = 98/2 as determined by ^{1}H NMR analysis of the isolated product) (The crude product was purified by column chromatography on silica gel to afford a part of pure **5ba**/**6ba** (petroleum ether (60-90 °C)/ethyl acetate = 8/1 (450 mL) to 6/1 (490 mL)) and impure **5ba**/**6ba** (determined by TLC). The impure part was further purified by column chromatography on silica gel (petroleum ether (60-90 °C)/ethyl acetate = 8/1) to afford another part of pure **5ba**/**6ba**) (**5ba**/**6ba** = 98/2 as determined by ^{1}H NMR analysis of the crude product using mesitylene (46 μL) as the internal standard).

5ba/**6ba** (98/2): solid; ^{1}H NMR (300 MHz, $CDCl_3$) δ 7.71 (d, *J* = 8.4 Hz, 2 H, ArH), 7.42-7.27 (m, 3 H, ArH), 7.27-7.17 (m, 4 H, ArH), 7.15 (s, 1 H, ArH), 5.60-5.50 (m, 1 H, =CH), 5.29 (br s, 1 H, NH), 5.15-5.02 (m, 2 H, OCH_2), 5.02-4.85 (m, 2 H, OCH_2), 4.10 (d, *J* = 6.0 Hz, 2 H, NCH_2), 2.50-2.28 (m, 7 H, $CH_2 \times 2$ and CH_3), 2.00-1.70 (m, 2 H, CH_2); ^{13}C NMR (75 MHz, $CDCl_3$) δ 143.1, 139.5, 139.1, 138.7, 136.6, 136.0, 134.2, 133.6, 131.5, 131.0, 129.3, 128.4, 127.4, 127.2, 126.8, 73.3, 73.0, 44.4, 36.2, 32.9, 23.6, 21.2; IR (neat) ν (cm^{-1}) 3274, 3059, 3027, 2949, 2920, 2847, 1599, 1495, 1474, 1446, 1328, 1266, 1161, 1094, 1055; MS (EI): *m/z* (%)

290 ((M-Ts)$^+$, 100); Anal. Calcd. for $C_{27}H_{27}NO_3S$ (%): C 72.78, H 6.11, N 3.14; Found: C 72.58, H 6.31, N 3.06. The following signals are discernible for **6ba**: ^{1}H NMR (300 MHz, $CDCl_3$) δ 7.52 (d, *J* = 8.4 Hz, 2 H, ArH), 4.75-4.70 (m, 2 H, OCH_2), 3.95 (d, *J* = 6.3 Hz, 2 H, CH_2).

This mixture may further be purified by recystallization to afford the pure product **5ba**: m.p. 123.4-125.3 °C (*n*-hexane/DCM).

8. Synthesis of *N*-((4-(cyclohexenyl)-7-phenyl-1,3-dihydroisobenzofuran-5-yl)methyl)-4-methylbenzenesulfonamide (**5ja**). (zyc-3-38)

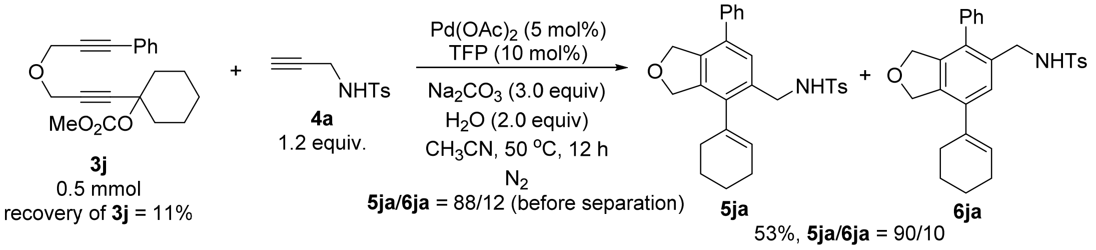

The reaction of Na_2CO_3 (159.0 mg, 1.5 mmol), $Pd(OAc)_2$ (5.6 mg, 0.025 mmol), TFP (11.6 mg, 0.05 mmol), **4a** (125.7 mg, 0.6 mmol), **3j** (163.4 mg, 0.5 mmol), and H_2O (18.1 mg, 1.0 mmol) in CH_3CN (5 mL) afforded **5ja**/**6ja** (121.1 mg, 53%, **5ja**/**6ja** = 90/10 as determined by ^{1}H NMR analysis of the isolated product) (eluent: petroleum ether (60-90 °C)/ethyl acetate = 10/1 (1100 mL) to 5/1 (480 mL)) (**5ja**/**6ja** = 88/12 as determined by ^{1}H NMR analysis of the crude product using mesitylene (23 μL) as the internal standard, recovery of **3j** = 11%).

5ja/**6ja** (90/10): solid; m.p. 152.2-153.8 °C (*n*-hexane/DCM); ^{1}H NMR (300 MHz, $CDCl_3$) δ 7.74 (d, *J* = 8.1 Hz, 2 H, ArH), 7.44-7.29 (m, 3 H, ArH), 7.26 (d, *J* = 8.1 Hz, 4 H, ArH), 7.14 (s, 1 H, ArH), 5.57-5.44 (m, 1 H, =CH), 5.19-5.16 (m, 2 H,

OCH_2), 5.03-4.93 (m, 2 H, OCH_2), 4.88 (t, *J* = 6.0 Hz, 1 H, NH), 4.22-4.01 (m, 2 H, NCH_2), 2.39 (s, 3 H, CH_3), 2.13-1.91 (m, 4 H, $CH_2 \times 2$), 1.70-1.50 (m, 4 H, $CH_2 \times 2$); ^{13}C NMR (75 MHz, $CDCl_3$) δ 143.4, 139.4, 138.9, 136.8, 136.7, 136.6, 135.1, 134.4, 133.3, 129.6, 128.6, 128.5, 127.6, 127.5, 127.4, 127.1, 73.7, 72.9, 44.4, 29.3, 25.1, 22.6, 21.7, 21.4; IR (neat) ν (cm^{-1}) 3277, 2927, 2856, 1599, 1475, 1447, 1436, 1328, 1162, 1094, 1054; MS (EI): *m/z* (%) 459 (M^+, 0.69), 304 ($(M-Ts)^+$, 100); HRMS calcd. for $C_{28}H_{29}NO_3S$ [M^+]: 459.1868; Found: 459.1862. The following signals are discernible for **6ja**: ^1H NMR (300 MHz, $CDCl_3$) δ 7.53 (d, *J* = 8.4 Hz, 2 H, ArH), 7.17 (d, *J* = 8.4 Hz, 2 H, ArH), 5.74-5.66 (m, 1 H, =CH), 4.75-4.70 (m, 2 H, OCH_2), 4.66 (t, *J* = 6.0 Hz, 1 H, NH), 4.97 (d, *J* = 6.3 Hz, 2 H, NCH_2), 2.15-2.25 (m, 4 H, $CH_2 \times 2$), 1.82-1.77 (m, 4 H, $CH_2 \times 2$).

9. Synthesis of (4-(propen-2-yl)-7-(p-tolyl)-1,3-dihydroisobenzofuran-5-yl)methanol (**5cb**). (cfsy-zy-138, wwt-2-23)

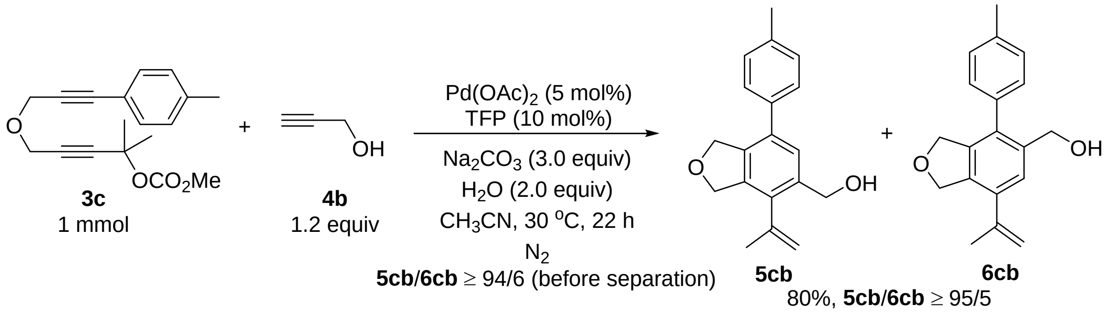

The reaction of Na_2CO_3 (317.5 mg, 3.0 mmol), $Pd(OAc)_2$ (11.3 mg, 0.05 mmol), TFP (23.4 mg, 0.1 mmol), **4b** (66.8 mg, 1.2 mmol), **3c** (300.7 mg, 1.0 mmol), and H_2O (36.0 μL, d = 1.0 g/mL, 2.0 mmol) in CH_3CN (10 mL) afforded **5cb** (224.5 mg, 80%, **5cb**/**6cb** ≥ 95/5 as determined by ^1H NMR analysis of the isolated product)

(eluent: petroleum ether (60-90 °C)/ethyl acetate = 20/1 (525 mL) to 5/1 (480 mL)) (**5cb**/**6cb** ≥ 94/6 as determined by ^{1}H NMR analysis of the crude product using mesitylene (46 μL) as the internal standard).

5cb: liquid; ^{1}H NMR (400 MHz, $CDCl_3$) δ 7.42 (s, 1 H, ArH), 7.27 (d, *J* = 8.0 Hz, 2 H, ArH), 7.21 (d, *J* = 8.0 Hz, 2 H, ArH), 5.29-5.23 (m, 1 H, one proton of $=CH_2$), 5.21-5.14 (m, 2 H, OCH_2), 5.08-5.01 (m, 2 H, OCH_2), 4.91-4.85 (m, 1 H, one proton of $=CH_2$), 4.68 (s, 2 H, OCH_2), 2.38 (s, 3 H, CH_3), 2.23 (br s, 1 H, OH), 2.01 (s, 3 H, CH_3); ^{13}C NMR (100 MHz, $CDCl_3$) δ 142.5, 137.9, 137.5, 137.2, 136.8, 136.2, 135.3, 134.6, 129.3, 127.6, 115.9, 73.8, 72.9, 62.5, 23.7, 21.1; IR (neat) ν (cm^{-1}) 3412, 3076, 3024, 2915, 2858, 1640, 1518, 1477, 1444, 1370, 1330, 1286, 1136, 1057; MS (EI): *m/z* (%) 280 (M^+, 100); HRMS calcd. for $C_{19}H_{20}O_2$ [M^+]: 280.1463; Found: 280.1465. The following signals are discernible for **6cb**: ^{1}H NMR (400 MHz, $CDCl_3$) δ 7.10 (d, *J* = 8.0 Hz, 2 H, ArH), 5.01-4.98 (m, 1 H, one proton of $=CH_2$), 4.85-4.82 (m, 2 H, OCH_2), 4.49 (s, 2 H, OCH_2), 2.13 (s, 3 H, CH_3).

10. Synthesis of 1-((4-(cyclopentenyl)-7-(o-tolyl)-1,3-dihydroisobenzofuran-5-yl) methyl)-1*H*-indole-3-carbaldehyde (**5de**). (zyc-1-94)

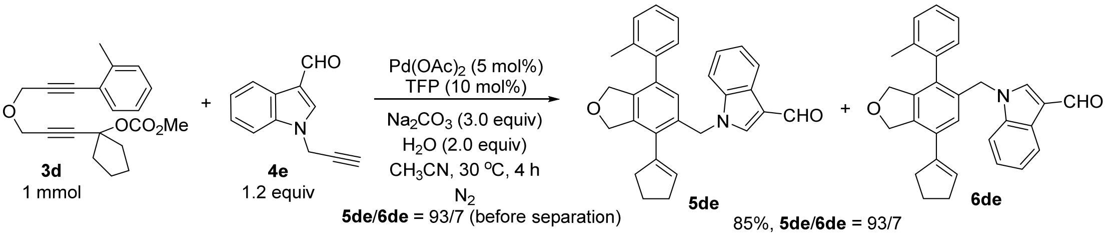

The reaction of **3d** (324.4 mg, 1.0 mmol), **4e** (219.8 mg, 1.2 mmol), $Pd(OAc)_2$ (11.1 mg, 0.05 mmol), TFP (23.7 mg, 0.1 mmol), Na_2CO_3 (317.8 mg, 3.0 mmol), and

H_2O (37.1 mg, 2.1 mmol) in CH_3CN (10 mL) afforded **5de**/**6de** (366.7 mg, 85%, **5de**/**6de** = 93/7 as determined by ^1H NMR analysis of the isolated product) (eluent: petroleum ether (60-90 °C)/ethyl acetate = 10/1 (550 mL) to 5/1 (720 mL)) (**5de**/**6de** = 93/7 as determined by ^1H NMR analysis of the crude product using mesitylene (46 μL) as the internal standard).

5de/**6de** (93/7): solid; ^1H NMR (300 MHz, $CDCl_3$) δ 9.93 (s, 1 H, CHO), 8.36-8.15 (m, 1 H, ArH), 7.59 (s, 1 H, ArH), 7.40-6.95 (m, 7 H, ArH), 6.84 (s, 1 H, ArH), 5.72-5.58 (m, 1 H, =CH), 5.33 (s, 2 H, NCH_2), 5.14-5.01 (m, 2 H, OCH_2), 4.97-4.85 (m, 2 H, OCH_2), 2.52-2.25 (m, 4 H, $CH_2 \times 2$), 2.00 (s, 3 H, CH_3), 1.96-1.79 (m, 2 H, CH_2); ^{13}C NMR (75 MHz, $CDCl_3$) δ 184.5, 139.6, 139.1, 138.6, 138.3, 137.3, 135.2, 134.7, 131.9, 131.7, 131.2, 130.3, 128.8, 128.6, 127.8, 125.7, 125.3, 123.9, 122.9, 122.0, 118.3, 110.2, 73.5, 73.4, 48.5, 36.1, 33.2, 23.6, 19.8; IR (KBr) ν (cm^{-1}) 3045, 2951, 2925, 2846, 1655, 1617, 1577, 1560, 1530, 1466, 1401, 1387, 1362, 1332, 1164, 1134, 1081, 1051, 1040; MS (EI): *m/z* (%) 433 (M^+, 100.0); Anal. Calcd. for $C_{30}H_{27}NO_2$ (%): C 83.11, H 6.28, N 3.23; Found: C 82.74, H 6.43, N 2.99. The following signals are discernible for **6de**: ^1H NMR (300 MHz, $CDCl_3$) δ 10.37 (s, 1 H, CHO), 8.64-8.55 (m, 1 H, ArH), 2.72-2.51 (m, 4 H, $CH_2 \times 2$).

This mixture may further be purified by recrystallization to afford the pure product **5de**: m.p. 84.0-87.0 °C (DCM).

11. Synthesis of *N*-((7-(4-methoxyphenyl)-4-(propen-2-yl)-1,3-dihydroisobenzofuran-5-yl)methyl)-4-methylbenzenesulfonamide (**5ea**). (zyc-1-101)

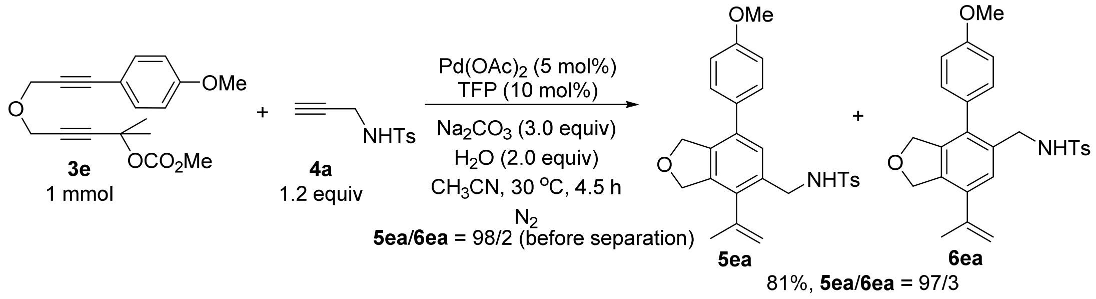

The reaction of **3e** (315.7 mg, 1.0 mmol), **4a** (252.2 mg, 1.2 mmol), $Pd(OAc)_2$ (11.4 mg, 0.05 mmol), TFP (23.9 mg, 0.1 mmol), Na_2CO_3 (318.4 mg, 3.0 mmol), and H_2O (37.0 mg, 2.0 mmol) in CH_3CN (10 mL) afforded **5ea**/**6ea** (364.0 mg, 81%, **5ea**/**6ea** = 97/3 as determined by ^{1}H NMR analysis of the isolated product) (eluent: petroleum ether (60-90 ^{o}C)/ethyl acetate = 5/1 (360 mL) to 3/1 (600 mL)) (**5ea**/**6ea** = 98/2 as determined by ^{1}H NMR analysis of the crude product using mesitylene (46 μL) as the internal standard).

5ea/**6ea** (97/3): solid; ^{1}H NMR (300 MHz, $CDCl_3$) δ 7.73 (d, J = 8.4 Hz, 2 H, ArH), 7.24 (d, J = 7.8 Hz, 2 H, ArH), 7.19 (d, J = 9.0 Hz, 2 H, ArH), 7.14 (s, 1 H, ArH), 6.91 (d, J = 9.0 Hz, 2 H, ArH), 5.24-5.14 (m, 2 H, one proton of $=CH_2$ and NH), 5.14-5.09 (m, 2 H, OCH_2), 5.01-4.94 (m, 2 H, OCH_2), 4.83-4.75 (m, 1 H, one proton of $=CH_2$), 4.13 (d, J = 5.7 Hz, 2 H, NCH_2), 3.83 (s, 3 H, CH_3), 2.38 (s, 3 H, CH_3), 1.87 (s, 3 H, CH_3); ^{13}C NMR (75 MHz, $CDCl_3$) δ 159.1, 143.3, 142.0, 138.1, 136.9, 136.3, 135.5, 134.3, 132.8, 131.7, 129.5, 128.7, 128.1, 127.1, 116.5, 114.0, 73.7, 72.8, 55.2, 44.2, 23.4, 21.4; IR (KBr) ν (cm^{-1}) 3265, 3005, 2963, 2927, 2895, 2834, 1654, 1643, 1610, 1519, 1482, 1450, 1438, 1412, 1348, 1325, 1290, 1251, 1211, 1179, 1159, 1117, 1093, 1054, 1036; MS (EI): m/z (%) 449 (M^+, 0.2), 294 ($(M-Ts)^+$, 100.0); Anal. Calcd. for $C_{26}H_{27}NO_4S$ (%): C 69.46, H 6.05, N 3.12; Found: C 69.46, H 6.14, N 2.86.

The following signals are discernible for **6ea**: ^1H NMR (300 MHz, $CDCl_3$) δ 7.54 (d, J = 8.1 Hz, 2 H, ArH), 4.93-4.91 (m, 2 H, OCH_2), 3.98 (d, J = 6.0 Hz, 2 H, NCH_2), 2.04 (s, 3 H, CH_3).

This mixture may further be purified by recrystallization to afford the pure product **5ea**: m.p. 152.4-154.1 °C (*n*-hexane/DCM).

12. Synthesis of 1-((7-(4-chlorophenyl)-4-(propen-2-yl)-1,3-dihydroisobenzofuran -5-yl)methyl)-5-methoxy-1*H*-indole (**5fg**). (zyc-1-87)

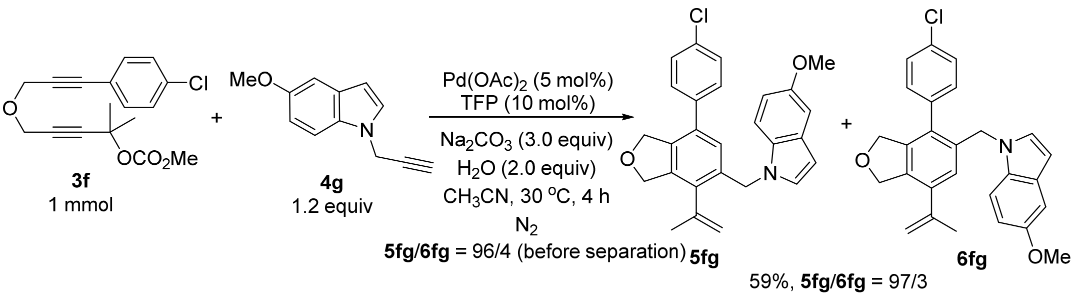

The reaction of Na_2CO_3 (318.2 mg, 3.0 mmol), $Pd(OAc)_2$ (11.5 mg, 0.05 mmol), TFP (24.8 mg, 0.1 mmol), **4g** (223.7 mg, 1.2 mmol), **3f** (320.8 mg, 1.0 mmol), and H_2O (36.8 mg, 2.0 mmol) in CH_3CN (10 mL) afforded **5fg**/**6fg** (252.3 mg, 59%, **5fg**/**6fg** = 97/3 as determined by ^1H NMR analysis of the isolated product) (The crude product was purified by column chromatography on silica gel (petroleum ether (60-90 °C)/ethyl acetate = 20/1) to afford impure **5fg**/**6fg** (281.1 mg), which was further purified with chromatography (petroleum ether (60-90 °C)/ethyl acetate = 40/1 (1000 mL) to 20/1 (700 mL)) (**5fg**/**6fg** = 96/4 as determined by ^1H NMR analysis of the crude product using mesitylene (46 μL) as the internal standard).

5fg/**6fg** (97/3): solid; ^1H NMR (300 MHz, $CDCl_3$) δ 7.31 (d, J = 8.4 Hz, 2 H,

ArH), 7.18-7.10 (m, 3 H, ArH), 7.08 (d, J = 2.4 Hz, 1 H, ArH), 7.04 (d, J = 3.0 Hz, 1 H, ArH), 6.87-6.77 (m, 2 H, ArH), 6.44 (dd, J_1 = 3.3 Hz, J_2 = 0.6 Hz, 1 H, ArH), 5.36-5.31 (m, 1 H, one proton of $=CH_2$), 5.29 (s, 2 H, NCH_2), 5.18-5.12 (m, 2 H, OCH_2), 5.12-5.06 (m, 2 H, OCH_2), 5.01-4.93 (m, 1 H, one proton of $=CH_2$), 3.83 (s, 3 H, OCH_3), 1.91 (s, 3 H, CH_3); ^{13}C NMR (75 MHz, $CDCl_3$) δ 154.0, 142.0, 138.6, 137.9, 136.6, 136.0, 134.3, 133.58, 133.56, 131.6, 129.0, 128.9, 128.8, 128.6, 127.0, 116.4, 112.0, 110.3, 102.5, 101.3, 73.6, 73.0, 55.7, 47.4, 23.1; IR (KBr) ν (cm^{-1}) 3101, 2986, 2964, 2903, 2830, 1619, 1577, 1487, 1449, 1385, 1366, 1342, 1295, 1255, 1239, 1187, 1150, 1131, 1092, 1054, 1026, 1014; MS (EI): *m/z* (%) 431 ($M(^{37}Cl)^+$, 25.2), 429 ($M(^{35}Cl)^+$, 69.1), 414 (100.0); HRMS calcd. for $C_{27}H_{24}{}^{35}ClNO_2$ [M^+]: 429.1496, Found: 429.1493. The following signals are discernible for **6fg**: ^1H NMR (300 MHz, $CDCl_3$) δ 6.38 (dd, J_1 = 3.2 Hz, J_2 = 0.8 Hz, 1 H, ArH), 5.22-5.18 (m, 2 H, OCH_2), 5.06-5.04 (m, 2 H, OCH_2), 4.93-4.91 (m, 1 H, one proton of $=CH_2$), 3.86 (s, 3 H, OCH_3), 1.97 (s, 3 H, CH_3).

This mixture may further be purified by recrystallization to afford the pure product **5fg**: m.p. 146.2-147.7 °C (*n*-hexane/DCM).

13. Synthesis of *N*-((4-(propen-2-yl)-7-(thiophen-3-yl)-1,3-dihydroisobenzofuran-5-yl)methyl)-4-methylbenzenesulfonamide (**5ga**). (zyc-1-131)

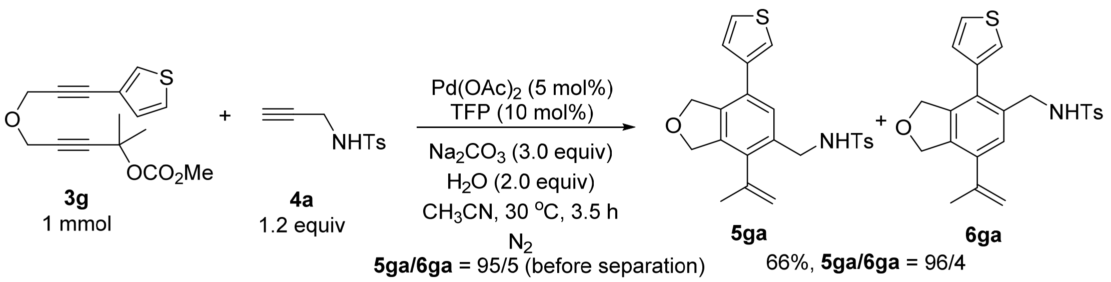

The reaction of Na_2CO_3 (318.2 mg, 3.0 mmol), $Pd(OAc)_2$ (11.4 mg, 0.05 mmol), TFP (24.1 mg, 0.1 mmol), **4a** (251.5 mg, 1.2 mmol), **3g** (292.1 mg, 1.0 mmol), and H_2O (37.0 mg, 2.0 mmol) in CH_3CN (10 mL) afforded **5ga**/**6ga** (281.8 mg, 66%, **5ga**/**6ga** = 96/4 as determined by ^1H NMR analysis of the isolated product) (The crude product was purified by column chromatography on silica gel (petroleum ether (60-90 °C)/ethyl acetate = 5/1 (500 mL) to 3/1 (300 mL)) to afford impure **5ga**/**6ga** (356.6 mg), which was further purified by recrystallization (*n*-hexane/DCM)) (**5ga**/**6ga** = 95/5 as determined by ^1H NMR analysis of the crude product using mesitylene (46 μL) as the internal standard).

5ga/6ga (96/4): solid; m.p. 146.2-148.3 °C (*n*-hexane/DCM); ^1H NMR (300 MHz, $CDCl_3$) δ 7.73 (d, *J* = 8.4 Hz, 2 H, ArH), 7.35 (t, *J* = 3.9 Hz, 1 H, ArH), 7.26 (d, *J* = 3.6 Hz, 2 H, ArH), 7.23 (s, 1 H, ArH), 7.13 (d, *J* = 4.2 Hz, 2 H, ArH), 5.29 (t, *J* = 5.9 Hz, 1 H, NH), 5.22-5.10 (m, 3 H, one proton of $=CH_2$ and OCH_2), 5.03-4.93 (m, 2 H, OCH_2), 4.82-4.73 (m, 1 H, one proton of $=CH_2$), 4.12 (d, *J* = 5.7 Hz, 2 H, CH_2), 2.39 (s, 3 H, CH_3), 1.86 (s, 3 H, CH_3); ^{13}C NMR (75 MHz, $CDCl_3$) δ 143.4, 141.8, 139.7, 138.2, 136.7, 135.9, 135.7, 132.8, 129.6, 129.0, 127.5, 127.0, 126.7, 126.1, 121.9, 116.5, 74.1, 72.9, 44.1, 23.4, 21.4; IR (KBr) ν (cm^{-1}) 3315, 3193, 2963, 2942, 2918, 2854, 1642, 1598, 1495, 1438, 1370, 1325, 1213, 1187, 1162, 1124, 1096, 1057, 1027; MS (EI): *m/z* (%) 425 (M^+, 0.07), 270 ($(M-Ts)^+$, 100.0); Anal. Calcd. for

$C_{23}H_{23}NO_3S_2$ (%): C 64.92, H 5.45, N 3.29, Found: C 64.57, H 5.49, N 3.21. The following signals are discernible for **6ga**: ^{1}H NMR (300 MHz, $CDCl_3$) δ 7.59 (d, *J* = 8.1 Hz, 2 H, ArH), 7.05-7.00 (m, 1 H, ArH), 6.88-6.83 (m, 1 H, ArH), 4.93-4.90 (m, 2 H, OCH_2), 4.02 (d, *J* = 5.7 Hz, 2 H, NCH_2), 2.03 (s, 3 H, CH_3).

14. Synthesis of *N*-((7-butyl-4-(propen-2-yl)-1,3-dihydroisobenzofuran-5-yl)methyl)-4-methylbenzenesulfonamide (**5ka**). (zyc-3-32)

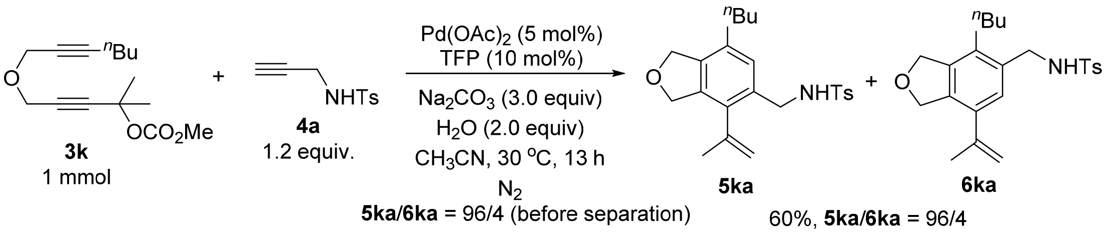

The reaction of Na_2CO_3 (318.0 mg, 3.0 mmol), $Pd(OAc)_2$ (11.2 mg, 0.05 mmol), TFP (23.3 mg, 0.1 mmol), **4a** (251.3 mg, 1.2 mmol), **3k** (266.2 mg, 1.0 mmol), and H_2O (36.1 mg, 2.0 mmol) in CH_3CN (10 mL) afforded **5ka**/**6ka** (238.8 mg, 60%, **5ka**/**6ka** = 96/4 as determined by ^{1}H NMR analysis of the isolated product) (eluent: petroleum ether (60-90 °C)/ethyl acetate = 10/1) (**5ka**/**6ka** = 96/4 as determined by ^{1}H NMR analysis of the crude product using mesitylene (46 μL) as the internal standard).

5ka/**6ka** (96/4): liquid; ^{1}H NMR (300 MHz, $CDCl_3$) δ 7.73 (d, *J* = 8.4 Hz, 2 H, ArH), 7.27 (d, *J* = 7.8 Hz, 2 H, ArH), 6.92 (s, 1 H, ArH), 5.37 (t, *J* = 5.3 Hz, 1 H, NH), 5.19-5.08 (m, 1 H, one proton of =CH_2), 5.08-4.96 (m, 2 H, OCH_2), 4.96-4.86 (m, 2 H, OCH_2), 4.75-4.65 (m, 1 H, one proton of =CH_2), 4.06 (d, *J* = 6.0 Hz, 2 H, NCH_2), 2.42 (s, 3 H, CH_3), 2.37 (t, *J* = 7.8 Hz, 2 H, CH_2), 1.82 (s, 3 H, CH_3), 1.55-1.38 (m, 2 H, CH_2), 1.38-1.21 (m, 2 H, CH_2), 0.90 (t, *J* = 7.2 Hz, 3 H, CH_3); ^{13}C NMR (75 MHz,

$CDCl_3$) δ 143.1, 142.0, 137.0, 136.81, 136.76, 134.9, 134.3, 132.2, 129.4, 128.2, 127.0, 116.1, 73.0, 72.8, 44.0, 32.6, 31.9, 23.4, 22.4, 21.3, 13.7; IR (neat) ν (cm^{-1}) 3277, 2956, 2930, 2859, 1639, 1598, 1444, 1329, 1161, 1094, 1056; MS (EI): *m/z* (%) 399 (M^+, 0.04), 244 ($(M-Ts)^+$, 100); HRMS calcd. for $C_{23}H_{29}NO_3S$ [M^+]: 399.1868; Found: 399.1864. The following signals are discernible for **6ka**: ^1H NMR (300 MHz, $CDCl_3$) δ 7.00 (s, 1 H, ArH), 4.85-4.80 (m, 1 H, one proton of $=CH_2$), 1.98 (s, 3 H, CH_3).

15. Synthesis of (4-(propen-2-yl)-2-tosyl-7-(trimethylsilyl)isoindolin-5-yl)methanol (**5lb**). (zyc-3-33)

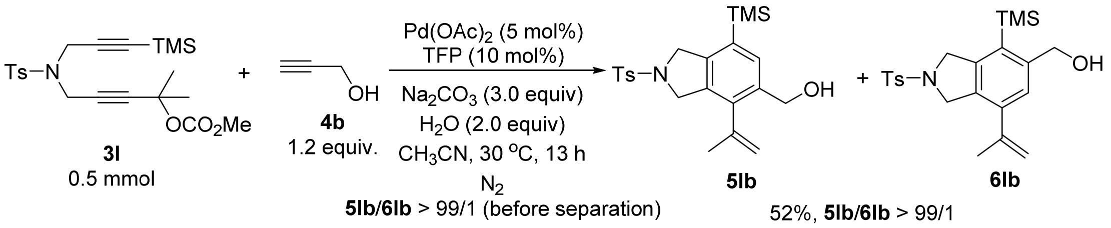

The reaction of Na_2CO_3 (158.9 mg, 1.5 mmol), $Pd(OAc)_2$ (5.5 mg, 0.025 mmol), TFP (11.6 mg, 0.05 mmol), **4b** (33.7 mg, 0.6 mmol), **3l** (227.1 mg, 0.5 mmol), and H_2O (18.1 mg, 1.0 mmol) in CH_3CN (5 mL) afforded **5lb**/**6lb** (108.7 mg, 52%, **5lb**/**6lb** > 99/1 as determined by ^1H NMR analysis of the isolated product) (eluent: petroleum ether (60-90 °C)/ethyl acetate = 10/1 (1000 mL) to 5/1 (600 mL)) (**5lb**/**6lb** > 99/1 as determined by ^1H NMR analysis of the crude product using mesitylene (23 μL) as the internal standard).

5lb: solid; m.p. 112.9-114.2 °C (*n*-hexane/DCM); ^1H NMR (300 MHz, $CDCl_3$) δ 7.75 (d, *J* = 8.1 Hz, 2 H, ArH), 7.44 (s, 1 H, ArH), 7.32 (d, *J* = 7.8 Hz, 2 H, ArH),

5.31-5.21 (m, 1 H, one proton of $=CH_2$), 4.83-4.76 (m, 1 H, one proton of $=CH_2$), 4.71-4.63 (m, 2 H, NCH_2), 4.63-4.54 (m, 2 H, NCH_2), 4.49 (s, 2 H, OCH_2), 2.40 (s, 3 H, CH_3), 1.96 (s, 4 H, OH and CH_3), 0.27 (s, 9 H, $CH_3 \times 3$); ^{13}C NMR (75 MHz, $CDCl_3$) δ 143.7, 142.1, 140.6, 138.7, 136.0, 133.7, 133.3, 133.1, 129.8, 127.4, 116.1, 62.5, 54.4, 52.4, 23.6, 21.4, -1.0; IR (neat) ν (cm^{-1}) 3527, 2954, 2908, 2856, 1637, 1597, 1383, 1347, 1251, 1163, 1098, 1066; MS (EI): *m/z* (%) 415 (M^+, 18.08), 91 (100); HRMS calcd. for $C_{22}H_{29}NO_3SSi$ [M^+]: 415.1637; Found: 415.1635.

16. Synthesis of *N*-((7-phenyl-4-(propen-2-yl)-1-propyl-1,3-dihydroisobenzofuran-5-yl)methyl)-4-methylbenzenesulfonamide (**5ha**). (zyc-1-71, wwt-2-132)

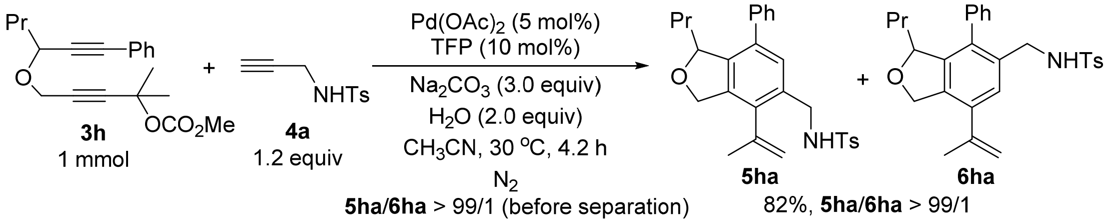

The reaction of **3h** (329.1 mg, 1.0 mmol), **4a** (251.5 mg, 1.2 mmol), $Pd(OAc)_2$ (11.2 mg, 0.05 mmol), TFP (23.8 mg, 0.1 mmol), Na_2CO_3 (318.7 mg, 3.0 mmol), and H_2O (35.8 mg, 2.0 mmol) in CH_3CN (10 mL) afforded **5ha** (378.7 mg, 82%, **5ha**/**6ha** > 99/1 as determined by ^{1}H NMR analysis of the isolated product) (eluent: petroleum ether (60-90 °C)/ethyl acetate = 10/1 (500 mL) to 5/1 (500 mL)) (**5ha**/**6ha** > 99/1 as determined by ^{1}H NMR analysis of the crude product using mesitylene (46 μL) as the internal standard).

5ha: solid; m.p. 127.4-130.2 °C (*n*-hexane/DCM); ^{1}H NMR (300 MHz, $CDCl_3$) δ 7.73 (d, *J* = 8.1 Hz, 2 H, ArH), 7.44-7.33 (m, 3 H, ArH), 7.28-7.20 (m, 4 H, ArH),

7.02 (s, 1 H, ArH), 5.66-5.56 (m, 1 H, OCH), 5.23-5.16 (m, 1 H, one proton of =CH_2), 5.04-4.92 (m, 2 H, OCH_2), 4.86-4.78 (m, 1 H, one proton of =CH_2), 4.75 (t, J = 6.0 Hz, 1 H, NH), 4.15 (d, J = 6.3 Hz, 2 H, NCH_2), 2.39 (s, 3 H, CH_3), 1.91 (s, 3 H, CH_3), 1.33-1.02 (m, 4 H, CH_2 × 2), 0.67 (t, J = 7.1 Hz, 3 H, CH_3); ^{13}C NMR (75 MHz, $CDCl_3$) δ 143.3, 141.9, 139.4, 139.2, 138.2, 136.8, 135.9, 135.4, 132.6, 129.5, 129.3, 128.4, 128.0, 127.4, 127.1, 116.5, 84.0, 71.6, 44.2, 35.9, 23.4, 21.4, 18.1, 13.6; IR (neat) ν (cm^{-1}) 3280, 3061, 3030, 2957, 2929, 2871, 1641, 1599, 1495, 1446, 1329, 1289, 1160, 1094, 1072; MS (EI): *m/z* (%): 461 (M^+, 0.07), 418 ($(M-C_3H_7)^+$, 20.6), 306 (100); Anal. Calcd. for $C_{28}H_{31}NO_3S$ (%): C 72.85, H 6.77, N 3.03; Found: C 72.49, H 6.64, N 2.62.

17. Synthesis of *N*-((7-phenyl-4-(propen-2-yl)-2-(p-tosyl)isoindolin-5-yl)methyl)-4-methylbenzenesulfonamide (**5ia**). (wwt-2-31, zyc-1-143)

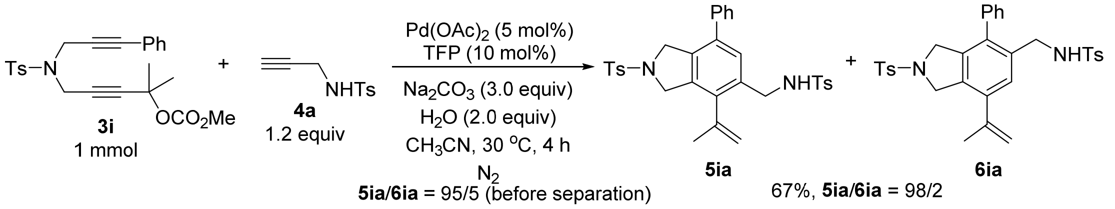

The reaction of Na_2CO_3 (318.6 mg, 3.0 mmol), $Pd(OAc)_2$ (11.4 mg, 0.05 mmol), TFP (23.1 mg, 0.1 mmol), **4a** (251.2 mg, 1.2 mmol), **3i** (440.8 mg, 1.0 mmol), and H_2O (36.2 mg, 2.0 mmol) in CH_3CN (10 mL) afforded **5ia/6ia** (382.8 mg, 67%, **5ia**/**6ia** = 98/2 as determined by ^1H NMR analysis of the isolated product) (eluent: petroleum ether (60-90 °C)/ethyl acetate = 7/1 to 5/1 to petroleum ether (60-90 °C)/ethyl acetate/DCM = 6/1/1) (**5ia**/**6ia** = 95/5 as determined by ^1H NMR analysis of

the crude product using mesitylene (46 μL) as the internal standard).

5ia/6ia (98/2): solid; ^{1}H NMR (300 MHz, $CDCl_3$) δ 7.77-7.64 (m, 4 H, ArH), 7.46-7.16 (m, 9 H, ArH), 7.08 (s, 1 H, ArH), 5.26-5.18 (m, 1 H, one proton of $=CH_2$), 4.79-4.74 (m, 1 H, one proton of $=CH_2$), 4.70 (t, J = 5.7 Hz, 1 H, NH), 4.64-4.57 (m, 2 H, NCH_2), 4.52-4.45 (m, 2 H, NCH_2), 4.08 (d, J = 6.0 Hz, 2 H, NCH_2), 2.40 (s, 3 H, CH_3), 2.38 (s, 3 H, CH_3), 1.87 (s, 3 H, CH_3); ^{13}C NMR (75 MHz, $CDCl_3$) δ 143.7, 143.5, 141.4, 138.8, 137.2, 136.7, 136.1, 135.0, 133.6, 133.41, 133.37, 129.8, 129.6, 129.0, 128.6, 127.8, 127.7, 127.5, 127.0, 117.0, 53.6, 52.9, 44.1, 23.4, 21.4; IR (neat) ν (cm^{-1}) 3301, 2968, 2923, 2847, 1597, 1493, 1477, 1426, 1344, 1329, 1157, 1095, 1067; MS (EI): *m/z* (%) 572 (M^+, 2.87), 417 (100); Anal. Calcd. for $C_{32}H_{32}N_2O_4S_2$ (%): C 67.11, H 5.63, N 4.89; Found: C 66.97, H 5.86, N 4.81. The following signals are discernible for **6ia**: ^{1}H NMR (300 MHz, $CDCl_3$) δ 4.24-4.21 (m, 2 H, NCH_2), 3.89 (d, J = 6.3 Hz, 2 H, CH_2), 1.75 (s, 3 H, CH_3).

This mixture may further be purified by recrystallization to afford the pure product **5ia**: m.p. 160.7-163.0 ^{o}C (*n*-hexane/DCM).

Control experiments

1. Synthesis of 5,7-diphenyl-4-(propen-2-yl)-1,3-dihydroisobenzofuran (**5ah**). (zyc-1-113)

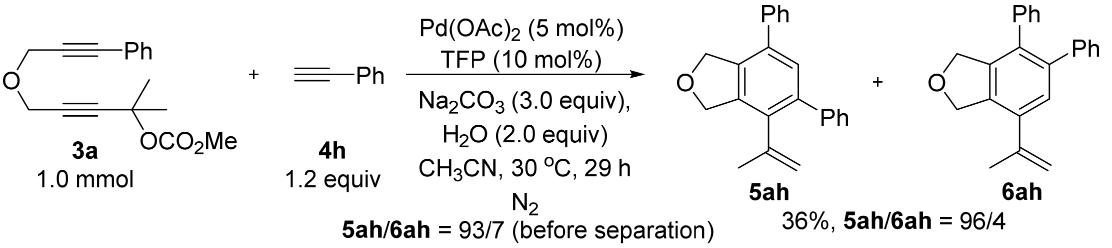

Following **Typical Procedure I**, the reaction of **3a** (286.1 mg, 1.0 mmol), **4h** (122.8 mg, 1.2 mmol), $Pd(OAc)_2$ (11.1 mg, 0.05 mmol), TFP (24.0 mg, 0.1 mmol), Na_2CO_3 (317.4 mg, 3.0 mmol), and H_2O (36.1 mg, 2.0 mmol) in CH_3CN (10 mL) afforded **5ah**/**6ah** (119.7 mg, purity = 93%, 36%, **5ah**/**6ah** = 96/4 as determined by ^1H NMR analysis of the isolated product) (The crude product was purified by column chromatography on silica gel (petroleum ether (60-90 °C)/ethyl acetate = 60/1) to afford impure **5ah**/**6ah** (determined by TLC). The impure **5ah**/**6ah** were purified by column chromatography on silica gel (petroleum ether (60-90 °C)/ethyl acetate = 100/1) to afford pure **5ah**/**6ah** and impure **5ah**/**6ah** (determined by TLC). The impure part was further purified by chromatography on silica gel (petroleum ether/ethyl acetate = 100/1) to afford another part of pure **5ah**/**6ah**.) (**5ah**/**6ah** = 93/7 as determined by ^1H NMR analysis of the crude product using mesitylene (46 μL) as the internal standard).

5ah/**6ah** (96/4): solid; ^1H NMR (300 MHz, $CDCl_3$) δ 7.55-7.27 (m, 11 H, ArH), 5.34-5.26 (m, 2 H, OCH_2), 5.25-5.19 (m, 1 H, one proton of $=CH_2$), 5.19-5.14 (m, 2 H, OCH_2), 5.07-5.02 (m, 1 H, one proton of $=CH_2$), 1.61 (s, 3 H, CH_3); ^{13}C NMR (75

MHz, $CDCl_3$) δ 143.6, 141.1, 139.8, 138.7, 136.2, 135.3, 134.5, 129.8, 129.1, 128.7, 128.0, 127.8, 127.5, 127.0, 116.9, 74.0, 73.8, 23.1; IR (KBr) ν (cm^{-1}) 3075, 3054, 3022, 2966, 2942, 2899, 2826, 1654, 1633, 1598, 1561, 1497, 1464, 1442, 1388, 1367, 1356, 1259, 1144, 1061, 1024; MS (EI): *m/z* (%) 312 (M^+, 65.83), 267 (100). The following signals are discernible for **6ah**: ^1H NMR (300 MHz, $CDCl_3$) δ 5.40-5.36 (m, 2 H, OCH_2), 4.93-4.91 (m, 1 H, one proton of OCH_2).

This mixture may further be purified by recrystallization to afford the pure product **5ah**: m.p. 87.2-89.2 °C ($CHCl_3$); Anal. Calcd. for $C_{23}H_{20}O$ (%): C 88.43, H 6.45; Found: C 88.20, H 6.51.

2. Synthesis of 5-butyl-7-phenyl-4-(propen-2-yl)-1,3-dihydroisobenzofuran (**5ai**). (zyc-2-28)

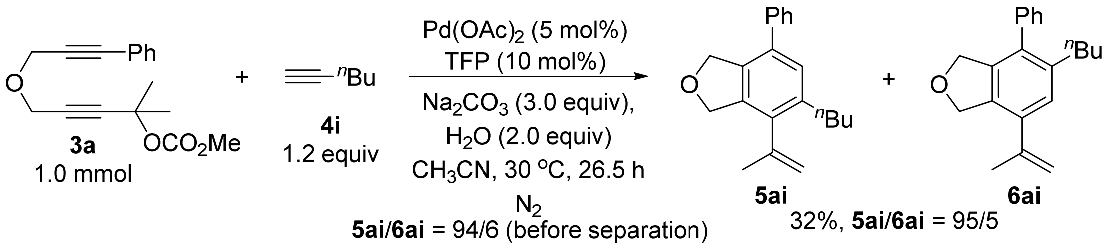

Following **Typical Procedure I**, the reaction of **3a** (285.9 mg, 1.0 mmol), **4i** (99.1 mg, 1.2 mmol), $Pd(OAc)_2$ (11.7 mg, 0.05 mmol), TFP (24.3 mg, 0.1 mmol), Na_2CO_3 (319.0 mg, 3.0 mmol), and H_2O (35.7 mg, 2.0 mmol) in CH_3CN (10 mL) afforded **5ai**/**6ai** (98.4 mg, purity = 95%, 32%, **5ai**/**6ai** = 95/5 as determined by ^1H NMR of the isolated product) (The crude product was purified by column chromatography on silica gel (petroleum ether (60-90 °C)/ethyl acetate = 100/1) to afford impure **5ai**/**6ai** (163.4 mg), which was further purified by column

chromatography on silica gel (petroleum ether (60-90 °C)/ethyl acetate = 100/1)) (**5ai**/**6ai** = 94/6 as determined by ^{1}H NMR analysis of the crude product using mesitylene (46 μL) as the internal standard).

5ai/**6ai** (95/5): liquid; ^{1}H NMR (300 MHz, $CDCl_3$) δ 7.48-7.27 (m, 5 H, ArH), 7.20 (s, 1 H, ArH), 5.28-5.23 (m, 1 H, one proton of =CH_2), 5.23-5.18 (m, 2 H, OCH_2), 5.10-5.03 (m, 2 H, OCH_2), 4.92-4.85 (m, 1 H, one proton of =CH_2), 2.63 (t, *J* = 8.0 Hz, 2 H, CH_2), 2.02 (t, *J* = 1.2 Hz, 3 H, CH_3), 1.64-1.52 (m, 2 H, CH_2), 1.47-1.31 (m, 2 H, CH_2), 0.93 (t, *J* = 7.4 Hz, 3 H, CH_3); ^{13}C NMR (75 MHz, $CDCl_3$) δ 143.1, 140.2, 139.6, 137.8, 136.1, 134.20, 134.18, 128.6, 128.5, 127.7, 127.2, 115.5, 73.9, 73.3, 34.3, 32.0, 23.8, 22.8, 14.0; IR (neat) ν (cm^{-1}) 3076, 3028, 2956, 2925, 2858, 1640, 1601, 1569, 1500, 1475, 1446, 1373, 1363, 1338, 1287, 1136, 1103, 1089, 1058, 1032; MS (EI): *m/z* (%) 292 (M^+, 100); HRMS calcd. for $C_{21}H_{24}O$ [M^+]: 292.1827, Found: 292.1826. The following signals are discernible for **6ai**: ^{1}H NMR (300 MHz, $CDCl_3$) δ 7.12 (s, 1 H, ArH), 4.84-4.79 (m, 1 H, one proton of =CH_2), 2.47 (t, *J* = 7.0 Hz, 2 H, CH_2), 2.16-2.13 (m, 3 H, CH_3), 0.77 (t, *J* = 7.2 Hz, 3 H, CH_3).

3. Synthesis of (4,6-diphenyl-7-(propen-2-yl)-1,3-dihydroisobenzofuran-5-yl)meth -anol (**5aj**). (zyc-2-35)

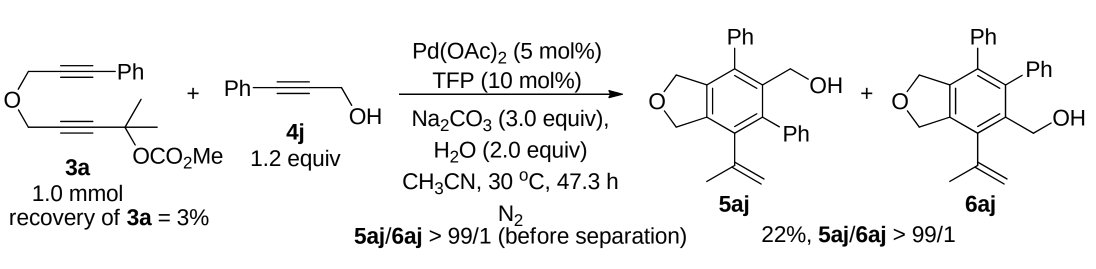

Following **Typical Procedure I**, the reaction of **3a** (286.5 mg, 1.0 mmol), **4j**

(159.4 mg, 1.2 mmol), $Pd(OAc)_2$ (11.9 mg, 0.05 mmol), TFP (23.4 mg, 0.1 mmol), Na_2CO_3 (318.7 mg, 3.0 mmol), and H_2O (36.1 mg, 2.0 mmol) in CH_3CN (10 mL) afforded **5aj** (75.4 mg, 22%, **5aj**/**6aj** > 99/1 as determined by ^1H NMR of the isolated product) (eluent: petroleum ether (60-90 °C)/ethyl acetate = 10/1) (**5aj**/**6aj** > 99/1 as determined by ^1H NMR of the crude product using mesitylene (46 μL) as the internal standard, recovery of **3a** = 3%).

5aj: solid; m.p. 146.6-148.5 °C (DCM/hexane); ^1H NMR (300 MHz, $CDCl_3$) δ 7.53-7.28 (m, 10 H, ArH), 5.18-5.12 (m, 2 H, OCH_2), 5.08-5.02 (m, 1 H, one proton of $=CH_2$), 4.97-4.91 (m, 2 H, OCH_2), 4.88-4.81 (m, 1 H, one proton of $=CH_2$), 4.22 (d, J = 6.0 Hz, 2 H, OCH_2), 1.58 (t, J = 1.1 Hz, 3 H, CH_3), 1.32 (t, J = 6.0 Hz, 1 H, OH); ^{13}C NMR (75 MHz, $CDCl_3$) δ 143.4, 140.3, 138.9, 138.5, 137.8, 136.8, 136.5, 135.8, 135.2, 130.2, 128.8, 128.4, 127.7, 127.5, 127.1, 116.5, 74.1, 74.0, 59.5, 23.2; IR (KBr) ν (cm^{-1}) 3447, 3074, 3060, 3028, 2951, 2911, 2860, 1639, 1598, 1497, 1469, 1441, 1364, 1310, 1272, 1205, 1177, 1049, 1024, 1001; MS (EI): *m/z* (%) 342 (M^+, 19.42), 279 (100); HRMS calcd. for $C_{24}H_{22}O_2$ [M^+]: 342.1620, Found: 342.1620.

4. Synthesis of 5-methyl-4,6-diphenyl-7-(propen-2-yl)-1,3-dihydroisobenzofuran (**5ak**). (zyc-2-51, zyc-2-60)

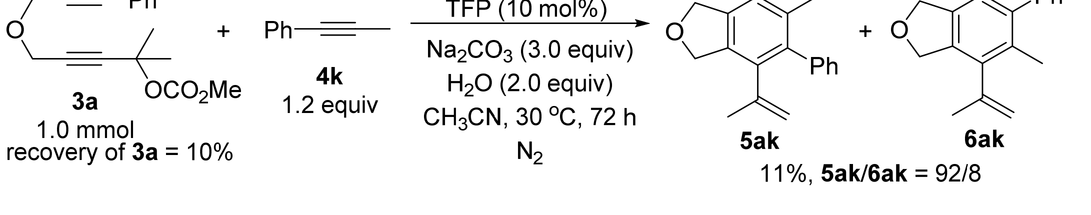

Following **Typical Procedure I**, the reaction of **3a** (286.2 mg, 1.0 mmol), **4k**

(143.4 mg, 1.2 mmol), $Pd(OAc)_2$ (11.3 mg, 0.05 mmol), TFP (23.8 mg, 0.1 mmol), Na_2CO_3 (318.3 mg, 3.0 mmol), and H_2O (36.1 mg, 2.0 mmol) in CH_3CN (10 mL) afforded **5ak/6ak** (36.0 mg, 11%, **5ak**/**6ak** = 92/8 as determined by ^{1}H NMR analysis of the isolated product) (eluent: petroleum ether (60-90 ^{o}C)/ethyl acetate = 100/1) (Recovery of **3a** = 10% as determined by ^{1}H NMR of the crude product using mesitylene (46 μL) as the internal standard).

5ak/**6ak** (92/8): solid; ^{1}H NMR (300 MHz, $CDCl_3$) δ 7.48-7.18 (m, 10 H, ArH), 5.17-5.09 (m, 2 H, OCH_2), 5.05-4.99 (m, 1 H, one proton of =CH_2), 4.95-4.89 (m, 2 H, OCH_2), 4.87-4.81 (m, 1 H, one proton of =CH_2), 1.85 (s, 3 H, CH_3), 1.57 (s, 3 H, CH_3); ^{13}C NMR (75 MHz, $CDCl_3$) δ 143.9, 140.5, 139.8, 139.7, 137.3, 136.2, 134.7, 134.2, 133.3, 130.1, 128.7, 128.5, 127.8, 127.2, 126.7, 116.2, 74.3, 74.1, 23.3, 18.4; IR (KBr) ν (cm^{-1}) 3075, 3045, 3019, 2959, 2899, 2850, 1493, 1439, 1371, 1358, 1330, 1289, 1261, 1220, 1177, 1056, 1027; GC-MS (GC condition: injector: 280 ^{o}C; column: DB-5, temperature: 60 ^{o}C (2 min), 20 ^{o}C/min to 280 ^{o}C, 280 ^{o}C (30 min)) (EI) *m/z* (%) for **5ak**: T_R 5.6 min: 326 (M^+, 100), 311 ($(M-CH_3)^+$, 29.97); for **6ak**: T_R 5.4 min: 326 (M^+, 100), 311 ($(M-CH_3)^+$, 18.14); HRMS calcd. for $C_{24}H_{22}O$ [M^+]: 326.1671, Found: 326.1672. The following signals are discernible for **6ak**: ^{1}H NMR (300 MHz, $CDCl_3$) δ 7.19-7.06 (m, 6 H, ArH), 7.03-6.96 (m, 4 H, ArH), 5.30-5.25 (m, 1 H, one proton of =CH_2), 4.98-4.95 (m, 2 H, OCH_2), 2.05 (s, 6 H, CH_3 × 2).

This mixture may further be purified by recrystallization to afford the pure product **5ak**: m.p. 146.8-148.3 ^{o}C (DCM/*n*-hexane).

5. The reaction of **3a** and **4l** under the standard conditions. (zyc-2-138)

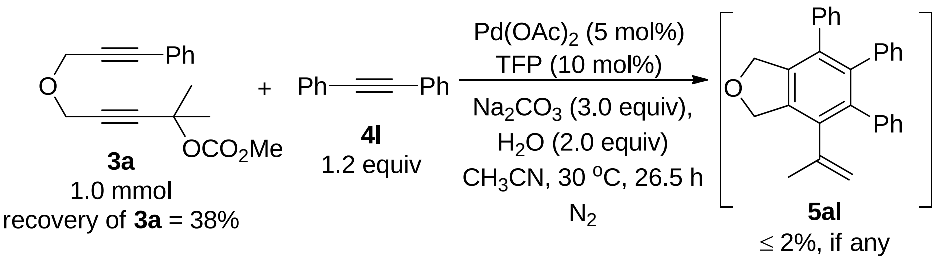

Following **Typical Procedure I**, the reaction of **3a** (286.1 mg, 1.0 mmol), **4l** (213.8 mg, 1.2 mmol), $Pd(OAc)_2$ (11.1 mg, 0.05 mmol), TFP (23.8 mg, 0.1 mmol), Na_2CO_3 (317.9 mg, 3.0 mmol), and H_2O (35.9 mg, 2.0 mmol) in CH_3CN (10 mL) afforded 7.3 mg of a complicated mixture (**5al,** ≤ 2%, if any) with 38% recovery of **3a** as determined by ^1H NMR analysis of the crude residue using mesitylene (46 μL) as the internal standard.

6. The reaction of **3a-OH** and **4a** under the standard conditions. (zyc-3-31)

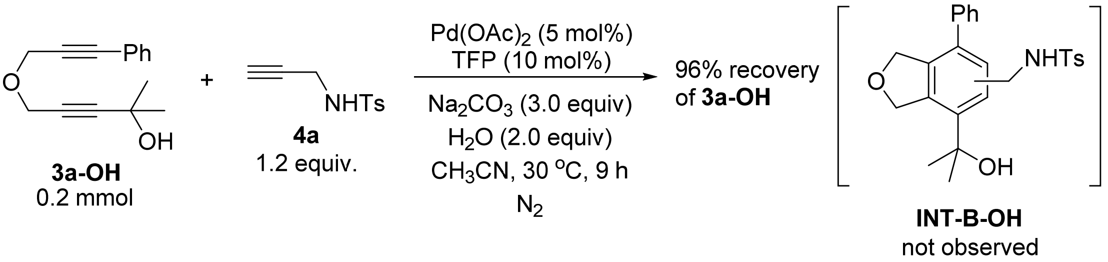

Following **Typical Procedure I**, the reaction of **3a-OH** (45.5 mg, 0.2 mmol), **4a** (50.3 mg, 0.24 mmol), $Pd(OAc)_2$ (2.2 mg, 0.01 mmol), TFP (4.7 mg, 0.02 mmol), Na_2CO_3 (63.7 mg, 0.6 mmol), and H_2O (7.2 μL, d = 1.0 g/mL, 7.2 mg, 0.4 mmol) in CH_3CN (2 mL) afforded 96% recovery of **3a-OH** as determined by ^1H NMR analysis of the crude residue using mesitylene (9.2 μL) as the internal standard.

7. Synthesis of starting material **3a-d$_6$**. (zyc-2-74)

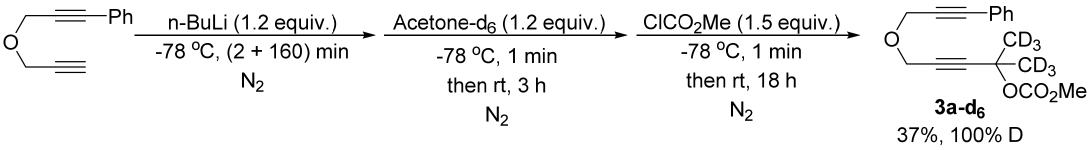

To a solution of 3-(propargyloxy)-phenyl-1-propyne (942.7 mg, 5.54 mmol) in THF (13 mL) were added dropwise *n*-BuLi (2.5 M in hexane, 2.64 mL, 6.6 mmol) at -78 ^{o}C within 2 minutes under N_2 atmosphere. After lithiation for 160 minutes at -78 ^{o}C, acetone-d$_6$ (0.5 mL, d = 0.852 g/mL, 426.0 mg, 6.64 mmol, 99.8% D) was added dropwise with an addition funnel at -78 ^{o}C within 1 minute. After the cooling bath was removed, the reaction mixture was warmed up to room temperature and stirred for 3 h. Then methyl chloroformate (0.64 mL, d = 1.22 g/mL, 780.8 mg, 8.26 mmol) was added dropwise at -78 ^{o}C within 1 minute. After the cooling bath was removed, the reaction mixture was warmed up to room temperature and stirred for 18 h. After the reaction was complete as monitored by TLC (eluent: petroleum ether/ ethyl acetate = 10/1), it was quenched with an aqueous solution of saturated NH_4Cl (10 mL). The resulting mixture was extracted with ethyl acetate (10 mL × 3) and the combined organic phase was dried over anhydrous Na_2SO_4. After filtration and evaporation of the solvent, the crude residue was purified by chromatography on silica gel (eluent: petroleum ether (60-90 ^{o}C)/ ethyl acetate = 100/1 (500 mL), and then petroleum ether/ ethyl acetate/diethyl ether = 100/1/1 (1500 mL) to 100/4/1 (800 mL)) to afford **3a-d$_6$** (594.3 mg, 37%, 100% D) as a liquid: ^{1}H NMR (300 MHz, $CDCl_3$) δ 7.49-7.41 (m, 2 H, ArH), 7.36-7.27 (m, 3 H, ArH), 4.47 (s, 2 H, OCH_2), 4.36 (s, 2 H, OCH_2), 3.76 (s, 3 H, OCH_3); ^{13}C NMR (75 MHz, $CDCl_3$) δ 153.5, 131.7, 128.5, 128.2, 122.5, 87.3,

86.7, 84.3, 80.0, 73.8, 57.2, 56.8, 54.3, 27.8 (septet); IR (neat) ν (cm^{-1}) 3054, 3022, 2955, 2898, 2852, 1755, 1599, 1490, 1442, 1383, 1351, 1271, 1233, 1194, 1119, 1083, 1013; MS (EI): *m/z* (%) 292 (M^+, 1.39), 115 (100); HRMS calcd. for $C_{17}H_{12}D_6O_4[M^+]$: 292.1582, Found: 292.1582.

8. Synthesis of *N*-((7-phenyl-4-(propen-2-yl-*d*$_5$)-1,3-dihydroisobenzofuran-5-yl)methyl)-4-methylbenzenesulfonamide (**5aa-d$_5$**). (zyc-2-76)

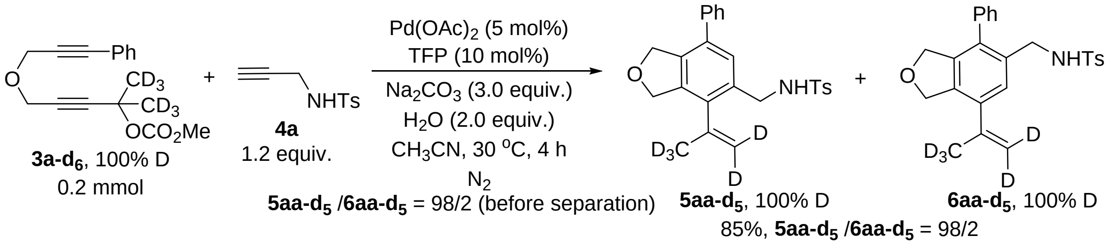

Following **Typical Procedure I**, the reaction of **3a-d$_6$** (58.6 mg, 0.2 mmol), **4a** (50.4 mg, 0.24 mmol), $Pd(OAc)_2$ (2.3 mg, 0.01 mmol), TFP (4.8 mg, 0.02 mmol), Na_2CO_3 (63.6 mg, 0.4 mmol), and H_2O (7.2 μL, d = 1.00 g/mL, 7.2 mg, 0.4 mmol) in CH_3CN (2 mL) afforded **5aa-d$_5$**/**6aa-d$_5$** (72.4 mg, 85%, 100% D, **5aa-d$_5$**/**6aa-d$_5$** = 98/2 as determined by ^{1}H NMR analysis of the isolated product) (eluent: petroleum ether (60-90 °C)/ethyl acetate = 5/1) (**5aa-d$_5$**/**6aa-d$_5$** = 98/2 as determined by ^{1}H NMR analysis of the crude product using mesitylene (9.2 μL) as the internal standard).

5aa-d$_5$/**6aa-d$_5$** (98/2): solid; ^{1}H NMR (300 MHz, $CDCl_3$) δ 7.72 (d, *J* = 8.1 Hz, 2 H, ArH), 7.42-7.10 (m, 8 H, ArH), 5.36-5.20 (t, *J* = 6.0 Hz, 1 H, NH), 5.15-5.04 (m, 2 H, OCH_2), 5.03-4.90 (m, 2 H, OCH_2), 4.14 (d, *J* = 5.7 Hz, 2 H, NCH_2), 2.36 (s, 3 H, CH_3); ^{13}C NMR (75 MHz, $CDCl_3$) δ 143.3, 141.6, 139.3, 138.1, 136.9, 136.5, 135.9, 134.6, 133.0, 129.5, 128.5, 128.4, 127.6, 127.4, 127.0, 116.0 (pentet), 73.6, 72.8, 44.1,

22.3 (septet); IR (KBr) ν (cm^{-1}) 3259, 3054, 2901, 2830, 1598, 1495, 1470, 1453, 1330, 1306, 1153, 1114, 1096, 1073, 1050; MS (EI): *m/z* (%) 424 (M^+, 0.14), 269 ($(M\text{-}Ts)^+$, 100); HRMS calcd. for $C_{25}H_{20}D_5NO_3S$ [M^+]: 424.1869, Found: 424.1870. The following signals are discernible for **6aa**: ^1H NMR (300 MHz, $CDCl_3$) δ 7.53 (d, *J* = 8.4 Hz, 2 H, ArH), 4.74-4.70 (m, 2 H, CH_2), 3.96 (d, *J* = 5.7 Hz, 2 H, NCH_2).

This mixture may further be purified by recrystallization to afford the pure product **5aa-d**$_5$: m.p. 150.4-152.0 ^oC (*n*-hexane/DCM).

Deuterium labelling experiments

1. Synthesis of starting material **D-4c**. (zyc-3-35)

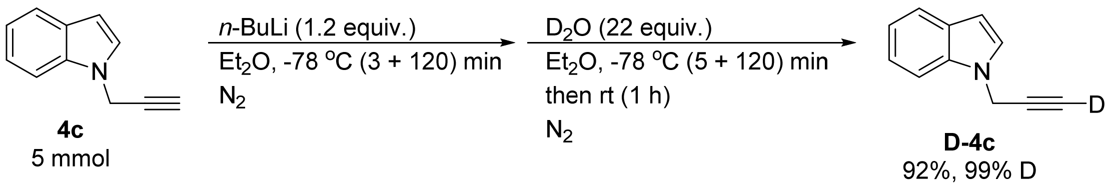

To a solution of **4c** (776.1 mg, 5.0 mmol) in Et_2O (30 mL) were added dropwise *n*-BuLi (2.5 M in hexane, 2.4 mL, 6.0 mmol) at -78 °C within 3 minutes under N_2 atmosphere. After lithiation for 120 minutes at -78 °C, D_2O (2.2 mL, d = 1.0 g/mL, 2200 mg, 110 mmol, 99.9% D) was added dropwise at -78 °C within 5 minutes. The resulting mixture was stirred for 2 hours. After the cooling bath was removed, the reaction mixture was warmed up to room temperature and stirred for 1 h. The resulting mixture was dried over anhydrous Na_2SO_4. After filtration and evaporation of the solvent, the crude residue was purified by chromatography on silica gel (eluent: petroleum ether (60-90 °C)/ ethyl acetate = 10/1) to afford **D-4c** (719.6 mg, 92%, 99% D) as a solid: m.p. 41.9-42.5 °C (*n*-hexane/Et_2O, -20 °C); ^{1}H NMR (300 MHz, $CDCl_3$) δ 7.64 (d, *J* = 7.8 Hz, 1 H, ArH), 7.40 (d, *J* = 8.1 Hz, 1 H, ArH), 7.30-7.21 (m, 1 H, ArH), 7.20 (d, *J* = 3.3 Hz, 1 H, ArH), 7.18-7.09 (m, 1 H, ArH), 6.57-6.51 (m, 1 H, ArH), 4.86 (s, 2 H, NCH_2); ^{13}C NMR (75 MHz, $CDCl_3$) δ 135.7, 128.8, 127.2, 121.8, 121.1, 119.8, 109.3, 102.0, 77.2 (t, *J* = 7.6 Hz), 73.2 (t, *J* = 38.5 Hz), 35.7; IR (neat) ν (cm^{-1}) 3055, 2589, 1986, 1612, 1514, 1484, 1463, 1397, 1363, 1335, 1315, 1259, 1189, 1012; MS (EI): *m/z* (%) 156 (M^+, 61.71), 155 (100); HRMS calcd. for $C_{11}H_8DN$ [M^+]: 156.0798, Found: 156.0795. The following signal is discernible for **4c**: 2.32 (s, 1 H, ≡CH).

2. Synthesis of **5ac**. (zyc-3-36)

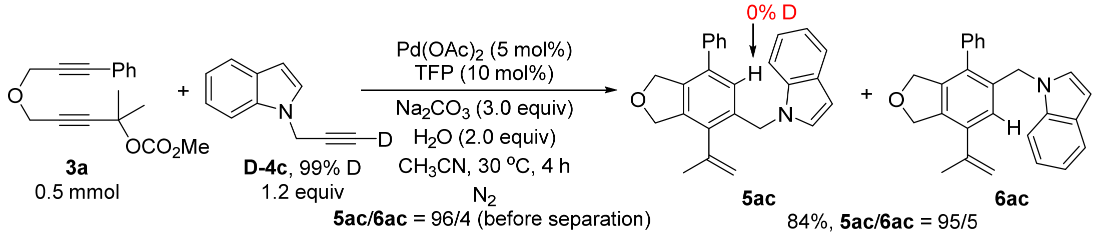

Following **Typical Procedure I**, the reaction of Na_2CO_3 (159.0 mg, 1.5 mmol), $Pd(OAc)_2$ (5.6 mg, 0.025 mmol), TFP (11.7 mg, 0.05 mmol), **D-4c** (93.8 mg, 0.6 mmol, 100% D), **3a** (143.0 mg, 0.5 mmol), and H_2O (18.1 mg, 1.0 mmol) in CH_3CN (5 mL) afforded **5ac**/**6ac** (153.4 mg, 84%, 0% D, **5ac**/**6ac** = 95/5 as determined by ^{1}H NMR analysis of the isolated product) (eluent: petroleum ether (60-90 ^{o}C)/ethyl acetate = 30/1) (**5ac**/**6ac** = 96/4 as determined by ^{1}H NMR analysis of the crude product using mesitylene (23 μL) as the internal standard).

5ac/**6ac** (95/5): solid; ^{1}H NMR (300 MHz, $CDCl_3$) δ 7.62 (d, J = 7.8 Hz, 1 H, ArH), 7.45-7.03 (m, 9 H, ArH), 6.93 (s, 1 H, ArH), 6.56-6.47 (m, 1 H, ArH), 5.37-5.28 (m, 3 H, one proton of $=CH_2$ and NCH_2), 5.23-5.17 (m, 2 H, OCH_2), 5.13-5.06 (m, 2 H, OCH_2), 5.02-4.95 (m, 1 H, one proton of $=CH_2$), 1.89 (s, 3 H, CH_3); ^{13}C NMR (75 MHz, $CDCl_3$) δ 142.1, 139.4, 138.4, 136.6, 136.3, 135.8, 134.8, 134.0, 128.6, 128.0, 127.6, 127.5, 127.3, 121.5, 120.9, 119.4, 116.3, 109.5, 101.7, 73.7, 73.0, 47.2, 23.1. The following signals are discernible for **6ac**: ^{1}H NMR (300 MHz, $CDCl_3$) δ 6.48-6.44 (m, 1 H, ArH), 5.46-5.38 (m, 3 H, one proton of $=CH_2$ and NCH_2), 5.15 (s, 2 H, OCH_2).

3. Synthesis of **D-5ac**. (zyc-3-46)

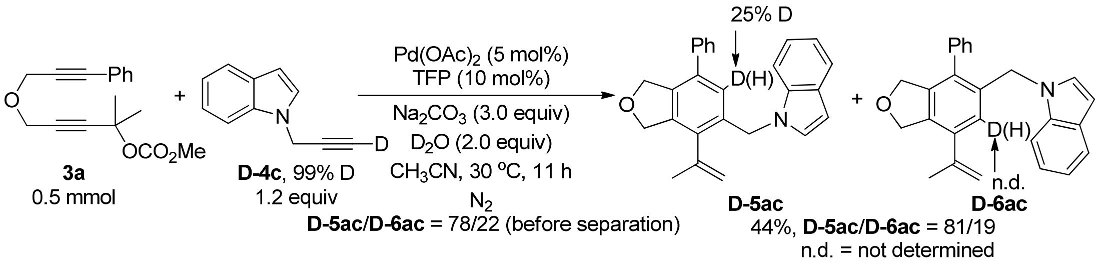

Following **Typical Procedure I**, the reaction of Na_2CO_3 (158.9 mg, 1.5 mmol), $Pd(OAc)_2$ (5.5 mg, 0.025 mmol), TFP (11.5 mg, 0.05 mmol), **D-4c** (93.7 mg, 0.6 mmol, 100% D), **3a** (143.0 mg, 0.5 mmol), and D_2O (20.1 mg, 1.0 mmol) in CH_3CN (5 mL) afforded **D-5ac**/**D-6ac** (81.1 mg, 44%, 25% D, **D-5ac**/**D-6ac** = 81/19 as determined by ^{1}H NMR analysis of the isolated product) (eluent: petroleum ether (60-90 °C)/ethyl acetate = 30/1) (**D-5ac**/**D-6ac** = 78/22 as determined by ^{1}H NMR analysis of the crude product using mesitylene (23 μL) as the internal standard).

D-5ac/**D-6ac** (81/19): solid; ^{1}H NMR (300 MHz, $CDCl_3$) δ 7.61 (d, J = 7.8 Hz, 1 H, ArH), 7.45-7.01 (m, 9 H, ArH), 6.51 (d, J = 3.0 Hz, 1 H, ArH), 5.34-5.25 (m, 3 H, one proton of $=CH_2$ and NCH_2), 5.23-5.15 (m, 2 H, OCH_2), 5.12-5.04 (m, 2 H, OCH_2), 5.01-4.93 (m, 1 H, one proton of $=CH_2$), 1.88 (s, 3 H, CH_3); IR (neat) ν (cm^{-1}) 3056, 2912, 2852, 1640, 1611, 1512, 1475, 1462, 1362, 1317, 1191, 1060; MS (EI): *m/z* (%) 366 ($(M(D))^+$, 34.62), 365 ($(M(H))^+$, 67.29), 350 (100); HRMS calcd. for $C_{26}H_{22}DNO$ [M^+]: 366.1842, Found: 366.1836. The following signals are discernible for **D-6ac**: ^{1}H NMR (300 MHz, $CDCl_3$) δ 6.47-6.43 (m, 1 H, ArH), 5.45-5.39 (m, 2 H, NCH_2), 5.14 (s, 2 H, OCH_2). The following signal is discernible for **5ac**: 6.93 (s, 1 H, ArH).

4. Synthesis of **D-4c'**. (zyc-3-48)

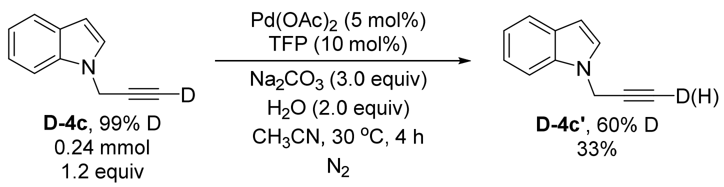

Following **Typical Procedure I**, the reaction of Na_2CO_3 (63.6 mg, 0.6 mmol), $Pd(OAc)_2$ (2.2 mg, 0.01 mmol), TFP (4.6 mg, 0.02 mmol), **D-4c** (37.6 mg, 0.24 mmol), and H_2O (7.2 μL, d = 1.0 g/mL, 7.2 mg, 0.4 mmol) in CH_3CN (2 mL) afforded **D-4c'** (12.5 mg, 33%, 60% D) (eluent: petroleum ether (60-90 °C)/ethyl acetate = 20/1) as a solid: ^{1}H NMR (400 MHz, $CDCl_3$) δ 7.63 (d, J = 7.6 Hz, 1 H, ArH), 7.40 (d, J = 8.4 Hz, 1 H, ArH), 7.28-7.21 (m, 1 H, ArH), 7.19 (d, J = 3.2 Hz, 1 H, ArH), 7.17-7.09 (m, 1 H, ArH), 6.53 (d, J = 3.2 Hz, 1 H, ArH), 4.88-4.82 (m, 2 H, NCH_2). The following signal is discernible for **4c**: 2.38 (t, J = 2.0 Hz, 1 H, ≡CH).

5. Synthesis of **D-5ac**. (zyc-3-41)

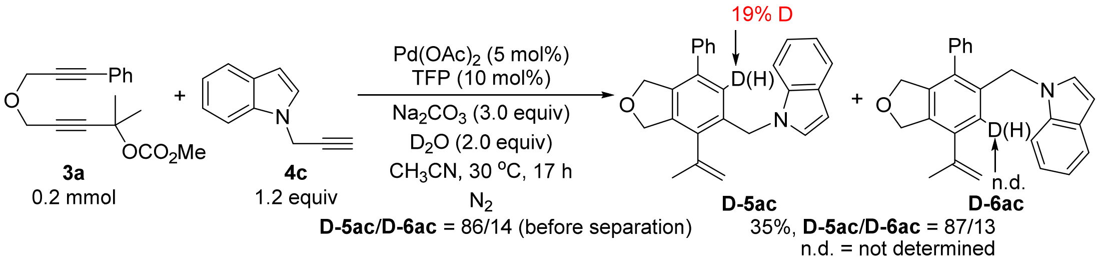

Following **Typical Procedure I**, the reaction of Na_2CO_3 (63.6 mg, 0.6 mmol), $Pd(OAc)_2$ (2.2 mg, 0.01 mmol), TFP (4.6 mg, 0.02 mmol), **4c** (37.2 mg, 0.24 mmol), **3a** (57.4 mg, 0.2 mmol), and D_2O (8.0 μL, d = 1.0 g/mL, 8.0 mg, 0.4 mmol) in CH_3CN (2 mL) afforded **D-5ac**/**D-6ac** (25.9 mg, 35%, 19% D, **D-5ac**/**D-6ac** = 87/13 as determined by ^{1}H NMR analysis of the isolated product) (eluent: petroleum ether (60-90 °C)/ethyl acetate = 30/1) (**D-5ac**/**D-6ac** = 86/14 as determined by ^{1}H NMR

analysis of the crude product using mesitylene (9.2 μL) as the internal standard).

D-5ac/**D-6ac** (87/13): solid; ^{1}H NMR (300 MHz, $CDCl_3$) δ 7.63 (d, *J* = 7.8 Hz, 1 H, ArH), 7.48-7.04 (m, 9 H, ArH), 6.52 (d, *J* = 3.0 Hz, 1 H, ArH), 5.38-5.28 (m, 3 H, one proton of =CH_2 and NCH_2), 5.24-5.17 (m, 2 H, OCH_2), 5.13-5.07 (m, 2 H, OCH_2), 5.02-4.95 (m, 1 H, one proton of =CH_2), 1.90 (s, 3 H, CH_3). The following signals are discernible for **D-6ac**: ^{1}H NMR (300 MHz, $CDCl_3$) δ 6.48-6.44 (m, 1 H, ArH), 5.46-5.42 (m, 2 H, NCH_2), 5.15 (s, 2 H, OCH_2). The following signal is discernible for **5ac**: 6.93 (s, 1 H, ArH).

Synthetic applications

1. Synthesis of 10-methyl-4-phenyl-7-(*p*-tosyl)-1,3,6,8-tetrahydro-1*H*-isobenzofuro-[5,4-*c*]azepine (**8aa**).[5,6] (zyc-1-148, zyc-1-164)

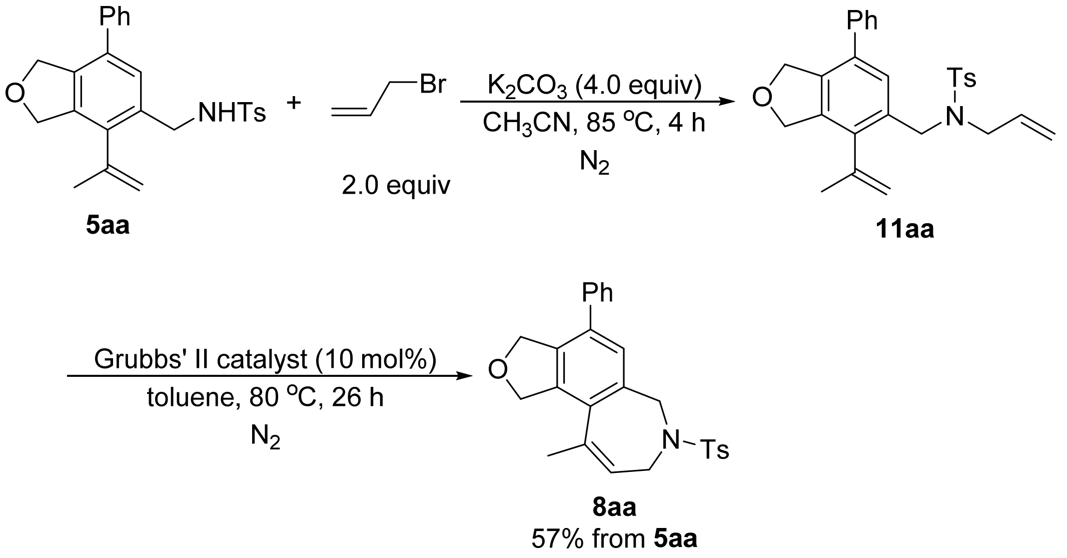

To a flame-dried Schlenk tube were added K_2CO_3 (429.6 mg, 3.12 mmol), **5aa** (327.1 mg, 0.78 mmol), and allyl bromide (188.6 mg, 1.56 mmol)/CH_3CN (2 mL) sequentially under N_2 atmosphere. Then the Schlenk tube was equipped with an internal condenser pipe and the resulting mixture was stirred with an oil bath pre-heated at 85 °C. The reaction was complete after being stirred for 4 hours as monitored by TLC. The reaction mixture was filtrated through a short column of silica gel eluted with ethyl acetate (5 mL × 3). After evaporation, the residue was purified by chromatography on silica gel to afford **11aa** (296.1 mg) (eluent: petroleum ether (60-90 °C)/ethyl acetate/DCM = 20/1/1)： solid; ^{1}H NMR (300 MHz, $CDCl_3$) δ 7.75 (d, *J* = 8.4 Hz, 2 H, ArH), 7.45-7.25 (m, 8 H, ArH), 5.63-5.45 (m, 1 H, =CH), 5.30-5.10 (m, 3 H, one proton of =CH_2 and OCH_2), 5.07-4.90 (m, 4 H, OCH_2 and =CH_2), 4.83 (s, 1 H, one proton of =CH_2), 4.44 (s, 2 H, NCH_2), 3.82 (d, *J* = 6.6 Hz, 2

H, NCH_2), 2.39 (s, 3 H, CH_3), 1.95 (s, 3 H, CH_3); ^{13}C NMR (75 MHz, $CDCl_3$) δ 143.2, 141.8, 139.5, 138.1, 137.3, 136.1, 135.7, 134.5, 132.7, 132.4, 129.7, 128.5, 127.6, 127.4, 127.2, 127.1, 119.1, 116.3, 73.7, 72.9, 49.9, 47.0, 23.3, 21.4. which was used directly in the next step without further characterization.

To a flame-dried Schlenk tube were added Grubbs' II catalyst (17.1 mg, 0.02 mmol), **11aa** (94.9 mg, 0.21 mmol), and toluene (10 mL) sequentially under N_2 atmosphere. Then the Schlenk tube was equipped with an internal condenser pipe and the resulting mixture was stirred in an oil bath pre-heated at 80 ^oC. The reaction was complete after being stirred for 26 hours as monitored by TLC (eluent: petroleum ether (60-90 ^oC)/ethyl acetate/DCM = 20/1/1). The resulting mixture was filtrated through a short column of silica gel eluted with ethyl acetate (10 mL × 3). After evaporation, the residue was purified by chromatography on silica gel to afford **8aa** (61.1 mg, 57% from **5aa**) (eluent: petroleum ether (60-90 ^oC)/ethyl acetate/DCM = 20/1/1 to 10/1/1).

8aa: solid; m.p. 162.6-164.6 ^oC (DCM/hexane); ^1H NMR (300 MHz, $CDCl_3$) δ 7.73 (d, J = 8.4 Hz, 2 H, ArH), 7.49-7.34 (m, 3 H, ArH), 7.33-7.18 (m, 4 H, ArH), 6.95 (s, 1 H, ArH), 5.86 (tq, J_1 = 7.4 Hz, J_2 = 1.4 Hz, 1 H, =CH), 5.11 (s, 4 H, OCH_2 × 2), 4.22 (s, 2 H, NCH_2), 3.52 (brs, 2 H, NCH_2), 2.37 (s, 3 H, CH_3), 2.00 (d, J = 1.2 Hz, 3 H, CH_3); ^{13}C NMR (75 MHz, $CDCl_3$) δ 143.3, 141.0, 139.1, 137.9, 137.4, 136.2, 135.2, 133.5, 132.7, 129.6, 129.3, 128.6, 127.65, 127.56, 127.4, 122.3, 73.3, 72.8, 49.1, 42.9, 21.3, 21.2; IR (neat) ν (cm^{-1}) 3054, 3028, 2963, 2917, 2857, 1631, 1597, 1493, 1457, 1446, 1400, 1378, 1362, 1325, 1307, 1292, 1164, 1112, 1089, 1072, 1059;

MS (EI): m/z (%) 431 (M^+, 11.94), 276 ($(M\text{-}Ts)^+$, 100); HRMS calcd. for $C_{26}H_{25}NO_3S$ [M^+]: 431.1555, Found: 431.1558.

2. Synthesis of *trans*-2-iodo-4'-phenyl-7'-(p-tosyl)-1',3',6'-trihydrospiro[cyclopentane-1,8'-[7*H*]-furo[3,4-e]isoindole] (**9ba**).[7] (wwt-3-51)

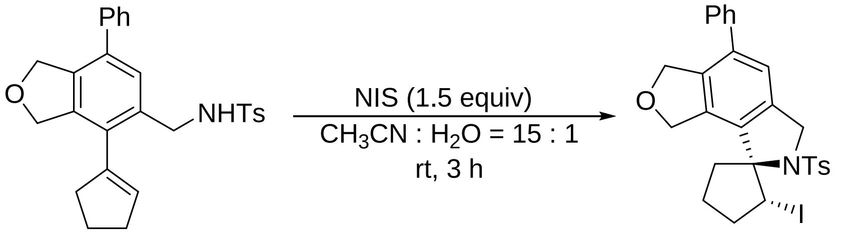

5ba **9ba** 55%

To a dry Schlenk tube were added **5ba** (89.1 mg, 0.2 mmol), CH_3CN (2.0 mL), water (0.14 mL), and NIS (67.8 mg, 0.3 mmol) sequentially. The reaction was complete after being stirred at room temperature for 3 hours as monitored by TLC. It was then quenched with an aqueous solution of saturated $Na_2S_2O_3$ (5 mL). The resulting mixture was extracted with ethyl ether (10 mL × 3), and the combined organic phase was dried over anhydrous Na_2SO_4. After filtration and evaporation of the solvent, the crude residue was purified by chromatography on silica gel to afford **9ba** (62.3 mg, 55%) (eluent: petroleum ether (60-90 °C)/ethyl acetate = 15/1).

9ba: solid; m.p. 135.0-137.2 °C (DCM/hexane); ^1H NMR (300 MHz, $CDCl_3$) δ 7.82 (d, J = 8.1 Hz, 2 H, ArH), 7.50-7.28 (m, 7 H, ArH), 7.12 (s, 1 H, ArH), 5.33 (dd, J_1 = 12.9 Hz, J_2 = 7.2 Hz, 1 H, CH), 5.27-5.14 (m, 3 H, OCH_2 and one proton of OCH_2), 5.07 (d, J = 12.3 Hz, 1 H, one proton of CH_2), 4.64 (d, J= 12.9 Hz, 1 H, one proton of NCH_2), 4.59 (d, J = 12.9 Hz, 1 H, one proton of NCH_2), 2.95-2.75 (m, 1 H,

one proton of CH_2), 2.60-2.45 (m, 1 H, one proton of CH_2), 2.43 (s, 3 H, CH_3), 2.41-2.24 (m, 2 H, CH_2), 2.24-2.05 (m, 1 H, one proton of CH_2), 1.98-1.80 (m, 1 H, one proton of CH_2); ^{13}C NMR (75 MHz, $CDCl_3$) δ 143.7, 140.3, 139.4, 138.3, 137.3, 136.1, 134.9, 133.9, 129.8, 128.7, 127.8, 127.7, 127.2, 121.5, 83.4, 73.0, 72.5, 53.3, 39.1, 36.8, 35.3, 26.3, 21.5; IR (KBr) ν (cm^{-1}) 2952, 2926, 2852, 1654, 1317, 1598, 1560, 1466, 1450, 1345, 1161, 1130, 1096, 1065; MS (EI): *m/z* (%) 289 ($(M\text{-}Ts\text{-}I)^+$, 1.79), 258 (100); Anal. Calcd. for $C_{27}H_{26}INO_3S$ (%): C 56.75, H 4.59, N 2.45, Found: C 56.99, H 4.89, N 2.12.

3. Synthesis of 7-phenyl-4-(propen-2-yl)-1,3-dihydroisobenzofuran-5-carbaldehyde (**10ab**).[8] (zyc-2-34)

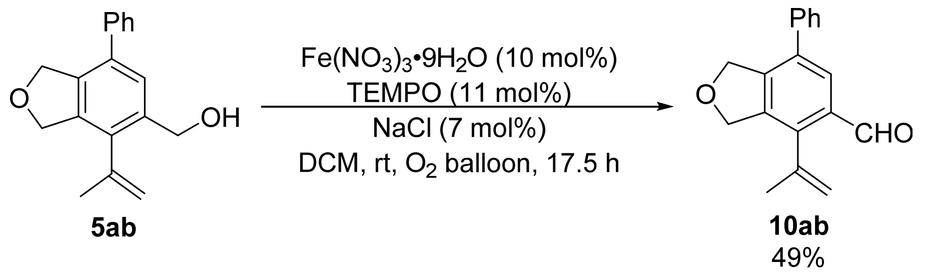

To a dry Schlenk tube were added $Fe(NO_3)_3$•$9H_2O$ (16.8 mg, 0.04 mmol), TEMPO (6.9 mg, 0.044 mmol), NaCl (1.6 mg, 0.028 mmol), DCM (2.0 mL), and **5ab** (106.3 mg, 0.4 mmol)/DCM (0.5 mL) sequentially. After being equipped with an O_2 balloon, the reaction was stirred at room temperature for 17.5 hours as monitored by TLC. The resulting reaction mixture was then filtrated through a short column of silica gel eluted with ethyl acetate (10 mL × 3). After evaporation, the residue was purified by chromatography on silica gel to afford **10ab** (51.2 mg, 49%) (eluent: petroleum ether (60-90 $^{\circ}C$)/ethyl acetate= 20/1).

10ab: solid; m.p. 83.0-83.7 °C (*n*-hexane/DCM); ^{1}H NMR (300 MHz, $CDCl_3$) δ 10.22 (s, 1 H, CHO), 7.93 (s, 1 H, ArH), 7.52-7.36 (m, 5 H, ArH), 5.52-5.44 (m, 1 H, one proton of $=CH_2$), 5.29-5.23 (m, 2 H, OCH_2), 5.19-5.13 (m, 2 H, OCH_2), 5.07-4.98 (m, 1 H, one proton of $=CH_2$), 2.13 (t, J = 1.2 Hz, 3 H, CH_3); ^{13}C NMR (75 MHz, $CDCl_3$) δ 191.3, 143.3, 140.1, 140.0, 139.0, 138.5, 135.2, 133.2, 128.8, 127.9, 127.8, 127.7, 118.3, 73.8, 72.6, 24.4; IR (KBr) ν (cm^{-1}) 3079, 2965, 2915, 2869, 2837, 2754, 1685, 1630, 1596, 1560, 1502, 1468, 1450, 1393, 1384, 1332, 1313, 1294, 1211, 1178, 1135, 1083, 1057, 1030, 1009; MS (EI): *m/z* (%) 264 (M^+, 100); Anal. Calcd. for $C_{18}H_{16}O_2$ (%): C 81.79, H 6.10, Found: C 81.82, H 6.10.

References

[1] X. Huang, W. Wu, C. Fu, Y. Yu, S. Ma, *Chem. Eur. J.* 2015, **21**, 15540.

[2] S. Song, C. Fu, X. Huang, S. Ma, *Adv. Synth. Catal.* 2018, **360**, 1019.

[3] N. Haider, J. Kaferbock, *Tetrahedron*, 2004, **60**, 6495.

[4] N. Haider, T. Kabicher, J. Kaferbock, A. Plenk, *Molecules*, 2007, **12**, 1900.

[5] X. Dong, R. Sang, Q. Wang, X. Tang, M. Shi, *Chem. Eur. J.* 2013, **19**, 16910.

[6] B. Bradshaw, P. Evans, J. Fletcher, A. Lee, P. Mwashimba, D. Oehlrich, E. Thomas, R. Davies, B. Allen, K. Broadley, A. Hamrounic, C. Escargueil, *Org. Biomol. Chem.* 2008, **6**, 2138.

[7] Y. Morino, I. Hidaka, Y. Oderaotoshi, M. Komatsu and S. Minakata, *Tetrahedron*, 2006, **62**, 12247.

[8] S. Ma, J. Liu, S. Li, B. Chen, J. Cheng, J. Kuang, Y. Liu, B. Wan, Y. Wang, J. Ye, Q. Yu, W. Yuan, S. Yu, *Adv. Synth. Catal.*, 2011, **353**, 1005.

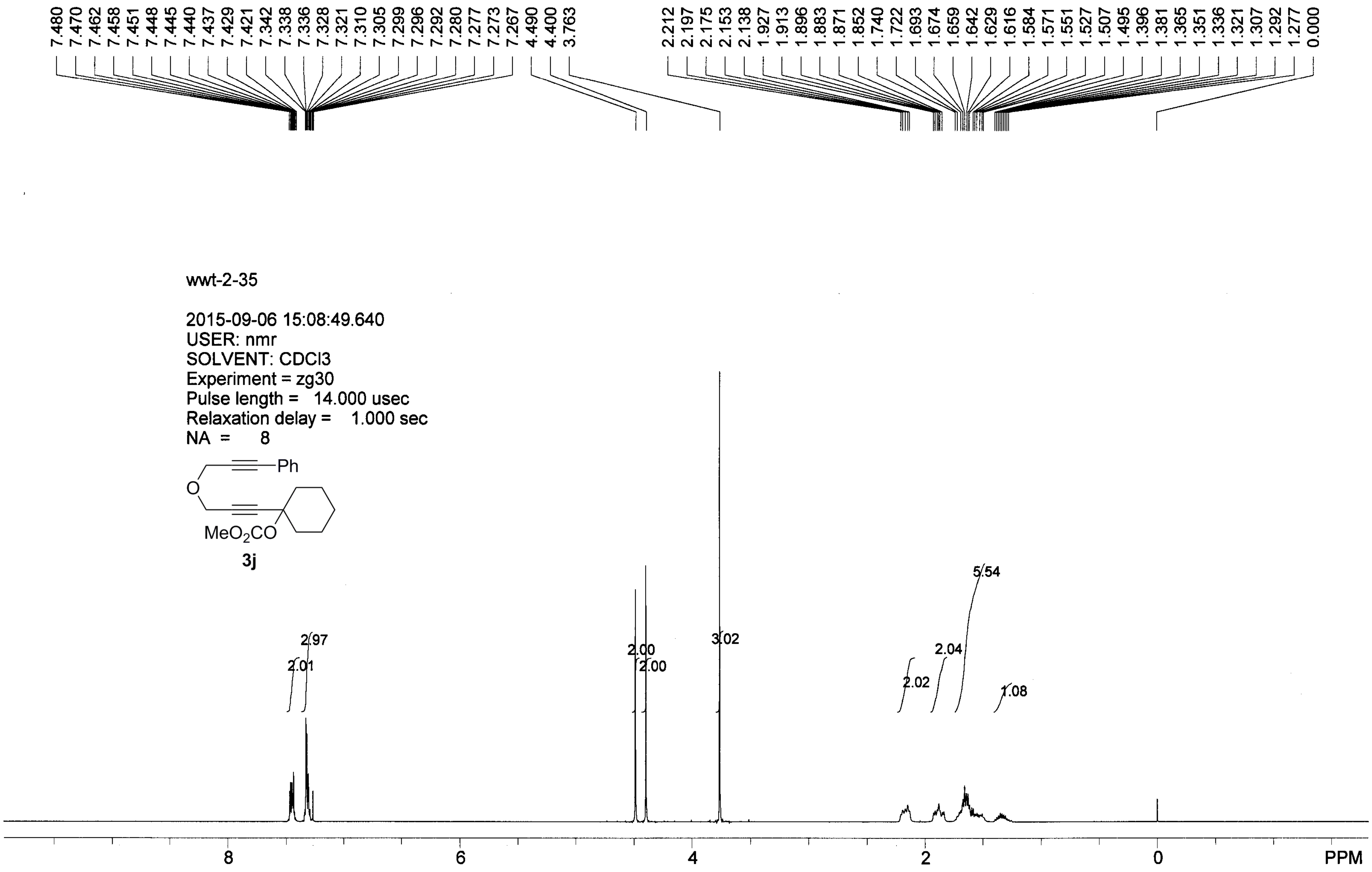
7.480
7.470
7.462
7.458
7.451
7.448
7.445
7.440
7.437
7.429
7.421
7.342
7.338
7.336
7.328
7.321
7.310
7.305
7.299
7.296
7.292
7.280
7.277
7.273
7.267
4.490
4.400
3.763
2.212
2.197
2.175
2.153
2.138
1.927
1.913
1.896
1.883
1.871
1.852
1.740
1.722
1.693
1.674
1.659
1.642
1.629
1.616
1.584
1.571
1.551
1.527
1.507
1.495
1.396
1.381
1.365
1.351
1.336
1.321
1.307
1.292
1.277
0.000
wwt-2-35
2015-09-06 15:08:49.640
USER: nmr
SOLVENT: CDCl3
Experiment = zg30
Pulse length = 14.000 usec
Relaxation delay = 1.000 sec
NA = 8
Ph
O
MeO2CO
3j
2.97
2.01
2.00
2.00
3.02
2.04
2.02
5.54
1.08
8
6
4
2
0
PPM

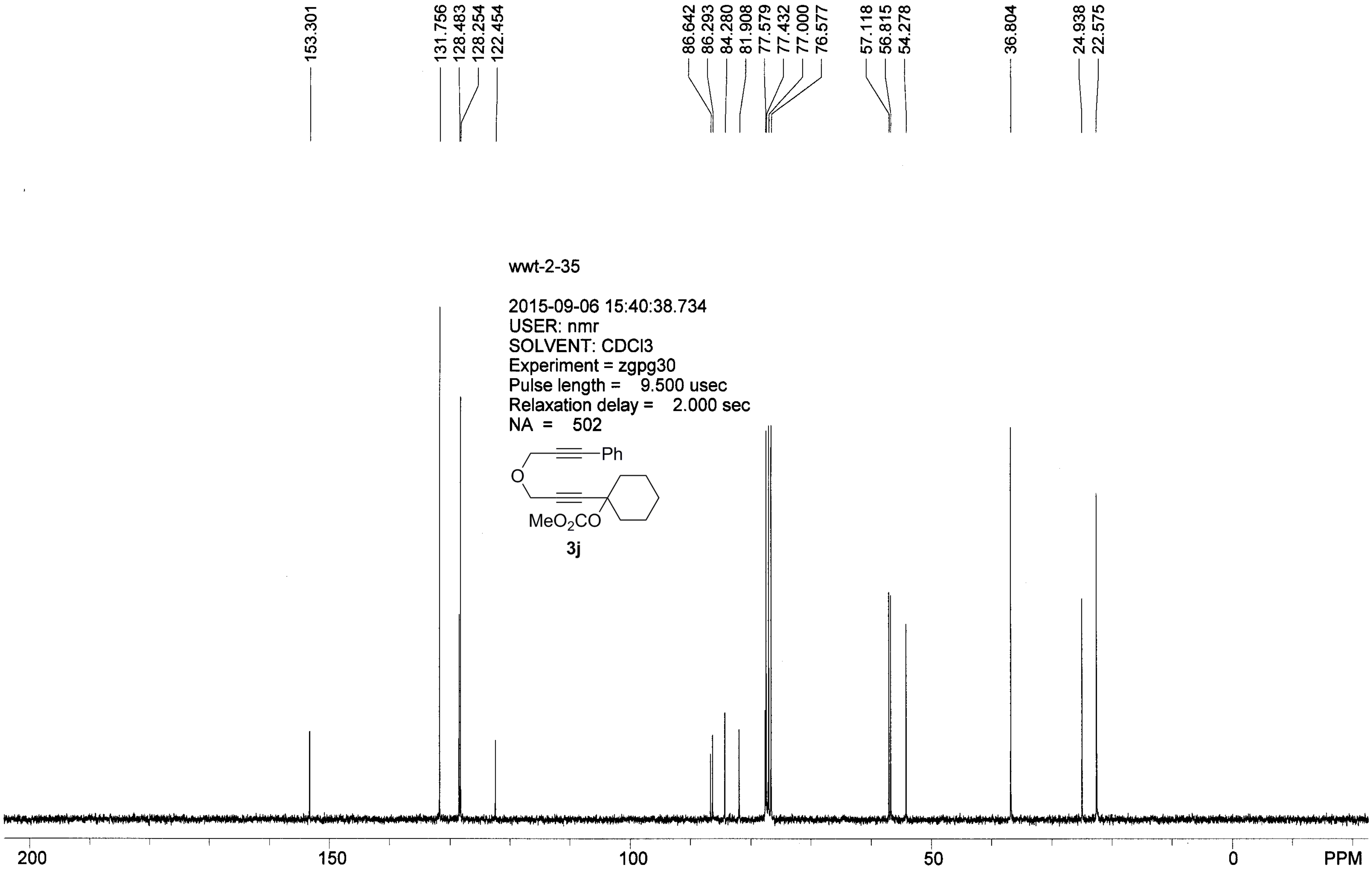
153.301
131.756
128.483
128.254
122.454
86.642
86.293
84.280
81.908
77.579
77.432
77.000
76.577
57.118
56.815
54.278
36.804
24.938
22.575
wwt-2-35
2015-09-06 15:40:38.734
USER: nmr
SOLVENT: CDCl3
Experiment = zgpg30
Pulse length = 9.500 usec
Relaxation delay = 2.000 sec
NA = 502
Ph
O
MeO2CO
3j
200
150
100
50
0
PPM

2018-11-21 14:02:45.312
zyc-3-26
SOLVENT: CDCl3
Experiment = zg30
Pulse length = 14.000 usec
Relaxation delay = 1.000 sec
NA = 8
F1 = 300.130005 MHz
F2 = 1.000000 MHz

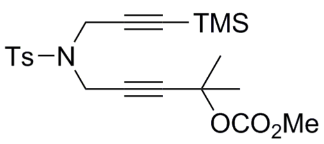

3l

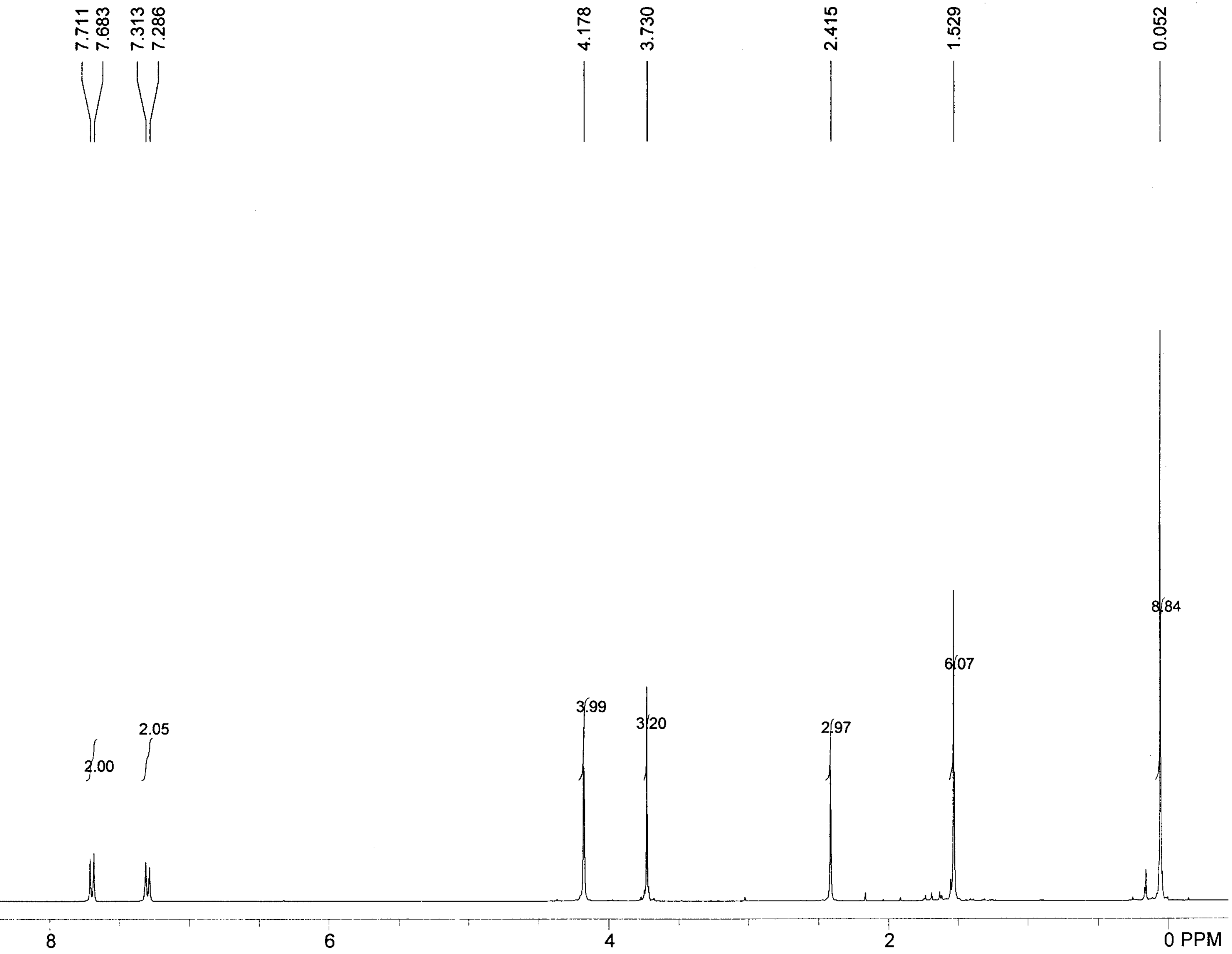

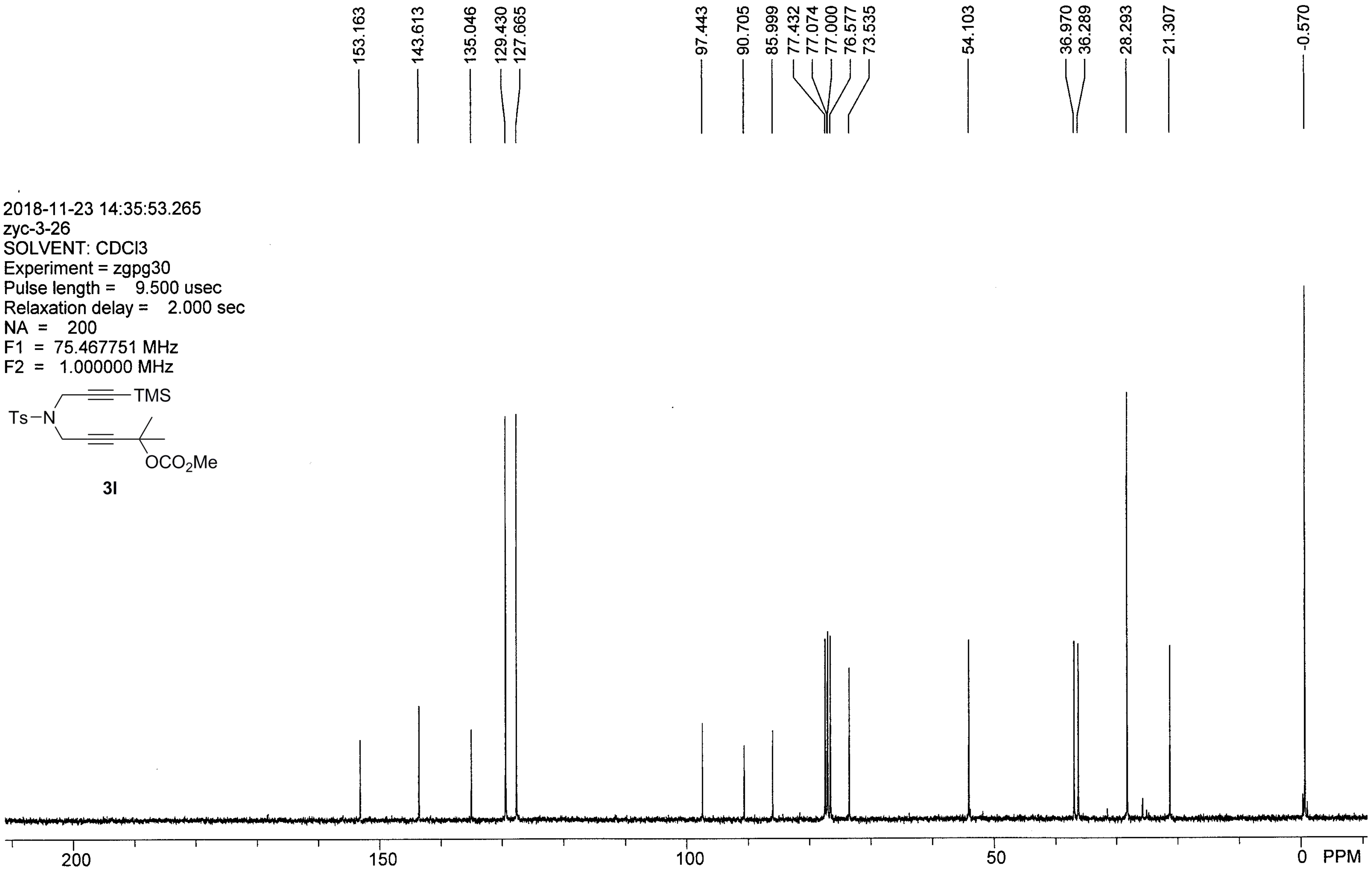
153.163
143.613
135.046
129.430
127.665
97.443
90.705
85.999
77.432
77.074
77.000
76.577
73.535
54.103
36.970
36.289
28.293
21.307
-0.570
2018-11-23 14:35:53.265
zyc-3-26
SOLVENT: CDCl3
Experiment = zgpg30
Pulse length = 9.500 usec
Relaxation delay = 2.000 sec
NA = 200
F1 = 75.467751 MHz
F2 = 1.000000 MHz
TMS
Ts–N
OCO2Me
3l
200
150
100
50
0
PPM

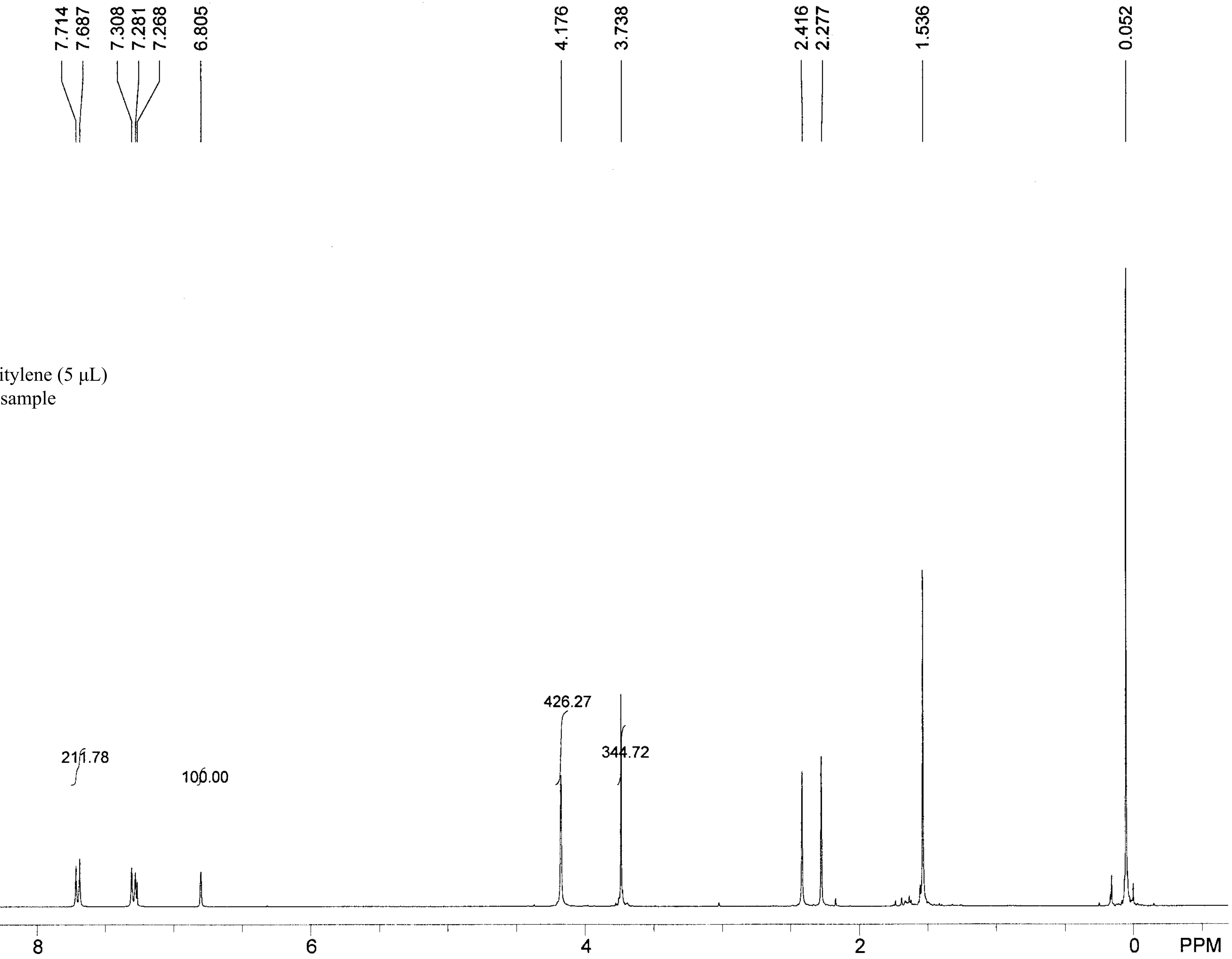

2018-11-21 20:09:26.125
zyc-3-26purity
SOLVENT: CDCl3
Experiment = zg30
Pulse length = 14.000 usec
Relaxation delay = 1.000 sec
NA = 8
F1 = 300.130005 MHz
F2 = 1.000000 MHz

Purity (96%) is determined by mesitylene (5 μL) as the internal standard in 52.2 mg sample

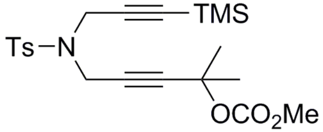

3l

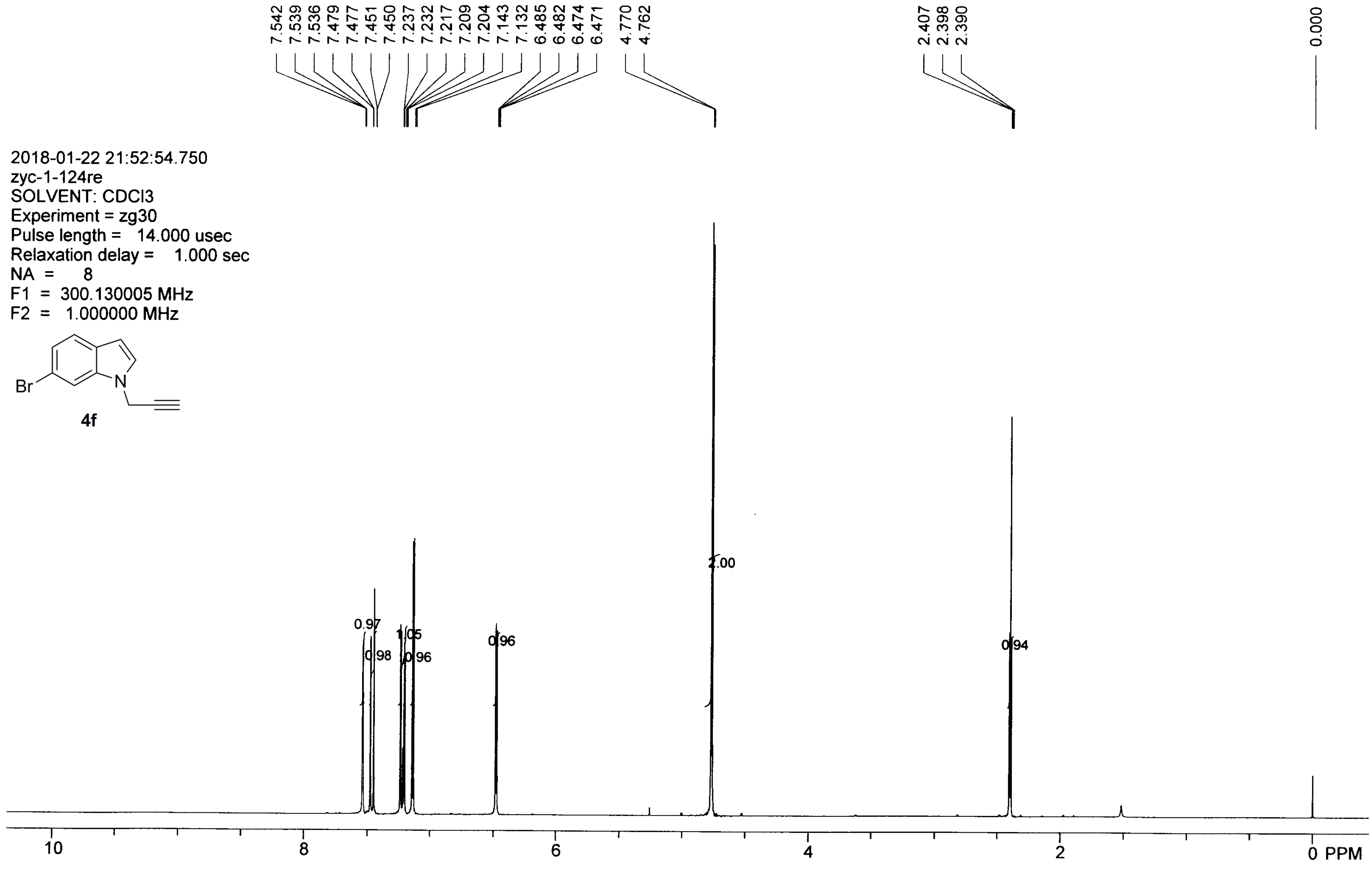
7.542
7.539
7.536
7.479
7.477
7.451
7.450
7.237
7.232
7.217
7.209
7.204
7.143
7.132
6.485
6.482
6.474
6.471
4.770
4.762
2.407
2.398
2.390
0.000
2018-01-22 21:52:54.750
zyc-1-124re
SOLVENT: CDCl3
Experiment = zg30
Pulse length = 14.000 usec
Relaxation delay = 1.000 sec
NA = 8
F1 = 300.130005 MHz
F2 = 1.000000 MHz
Br
N
4f
0.97
0.98
1.05
0.96
0.96
2.00
0.94
10
8
6
4
2
0 PPM

2018-01-22 22:08:11.734
zyc-1-124reC
SOLVENT: CDCl3
Experiment = zgpg30
Pulse length = 9.500 usec
Relaxation delay = 2.000 sec
NA = 229
F1 = 75.467751 MHz
F2 = 1.000000 MHz

4f

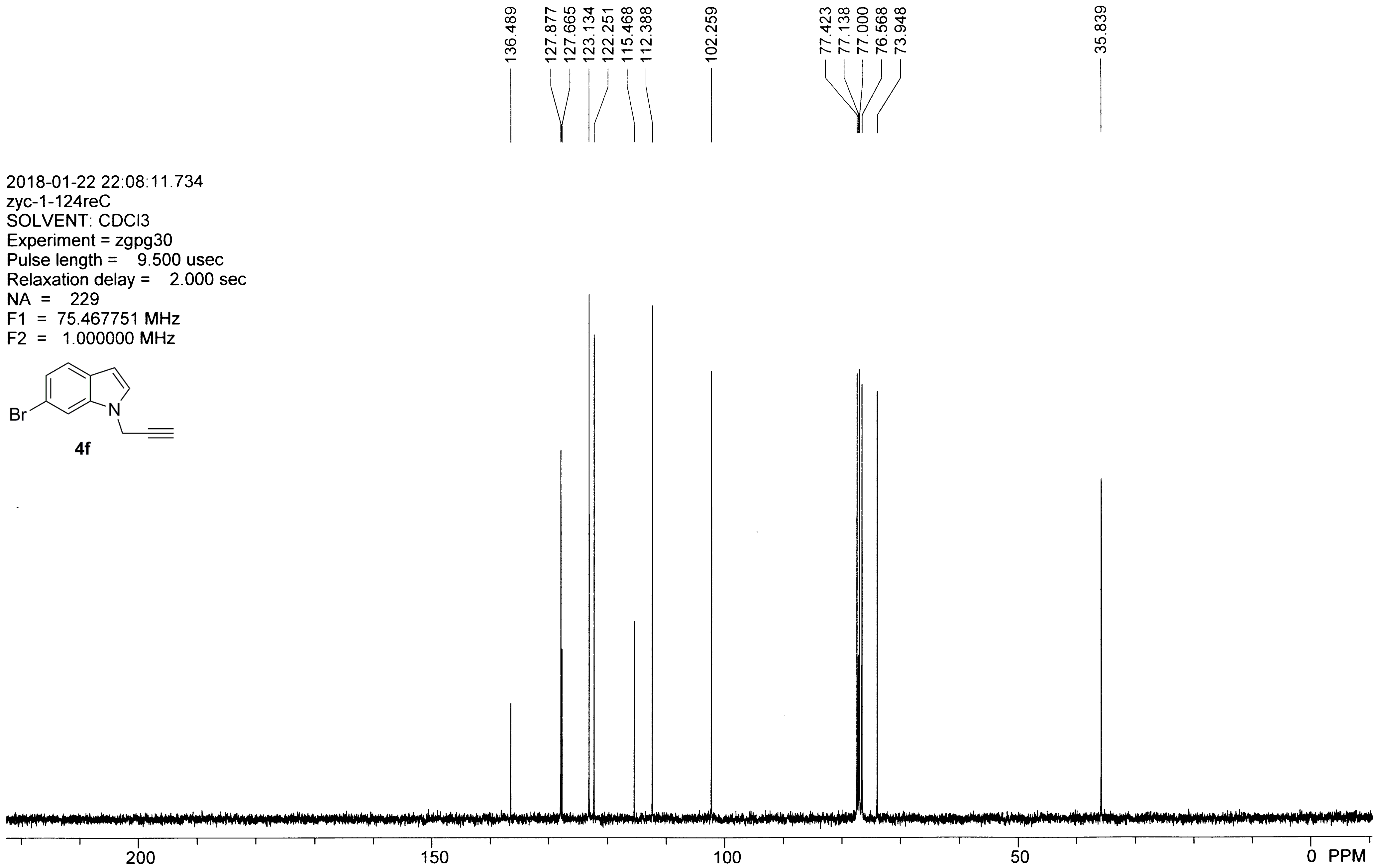

2015-06-20 14:51:37.812
wwt-1-193-4H
SOLVENT: CDCl3
Experiment = zg30
Pulse length = 14.000 usec
Relaxation delay = 1.000 sec
NA = 8
F1 = 300.130005 MHz
F2 = 1.000000 MHz

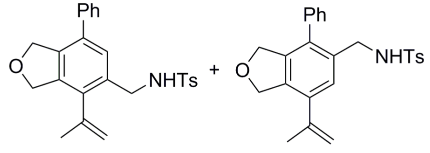

5aa **6aa**

5aa/6aa = 97/3

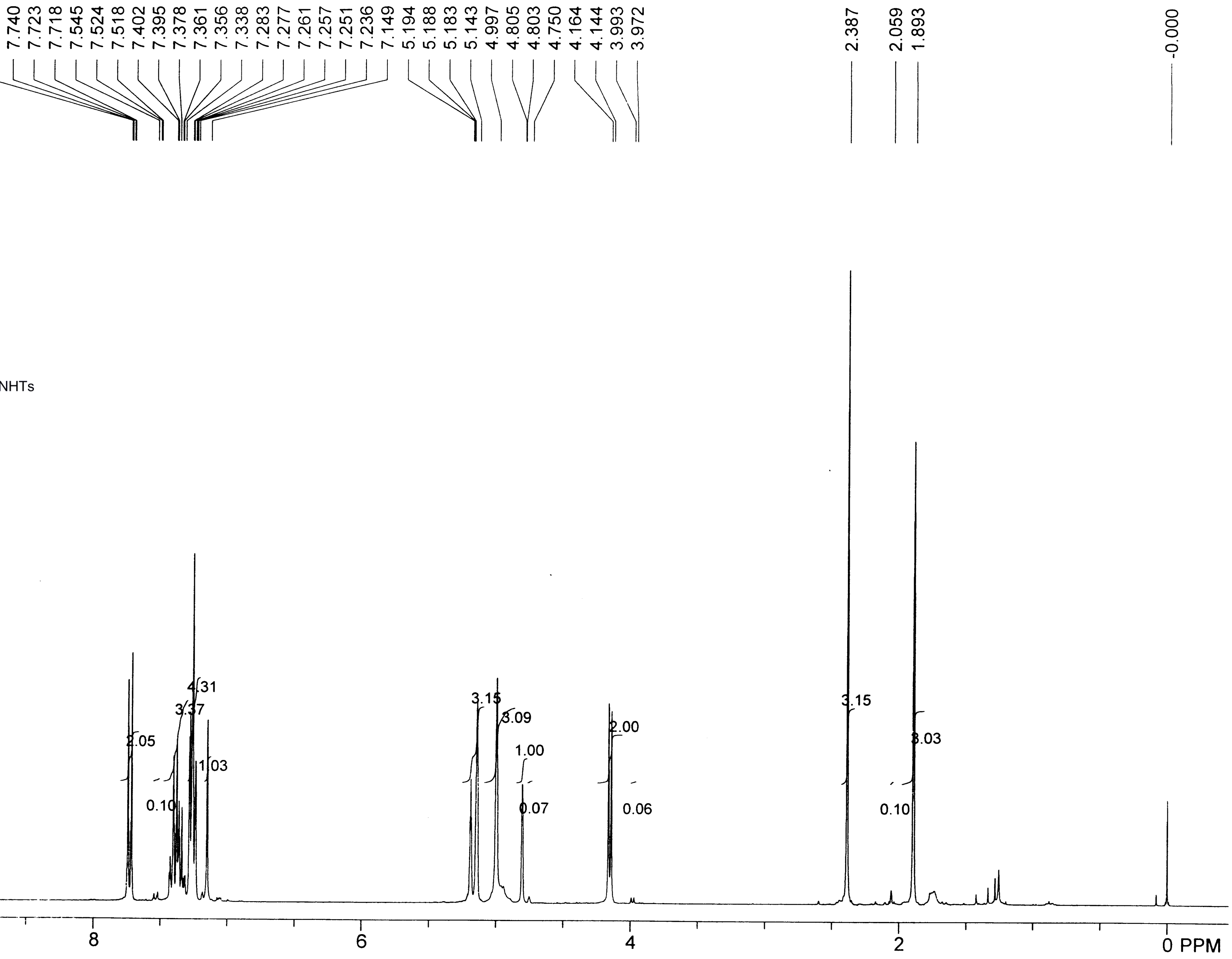

2015-06-20 15:11:42.734
wwt-1-193-4C
SOLVENT: CDCl3
Experiment = zgpg30
Pulse length = 9.500 usec
Relaxation delay = 2.000 sec
NA = 305
F1 = 75.467751 MHz
F2 = 1.000000 MHz

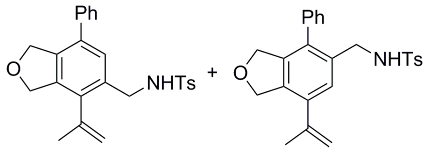

5aa **6aa**

5aa/**6aa** = 97/3

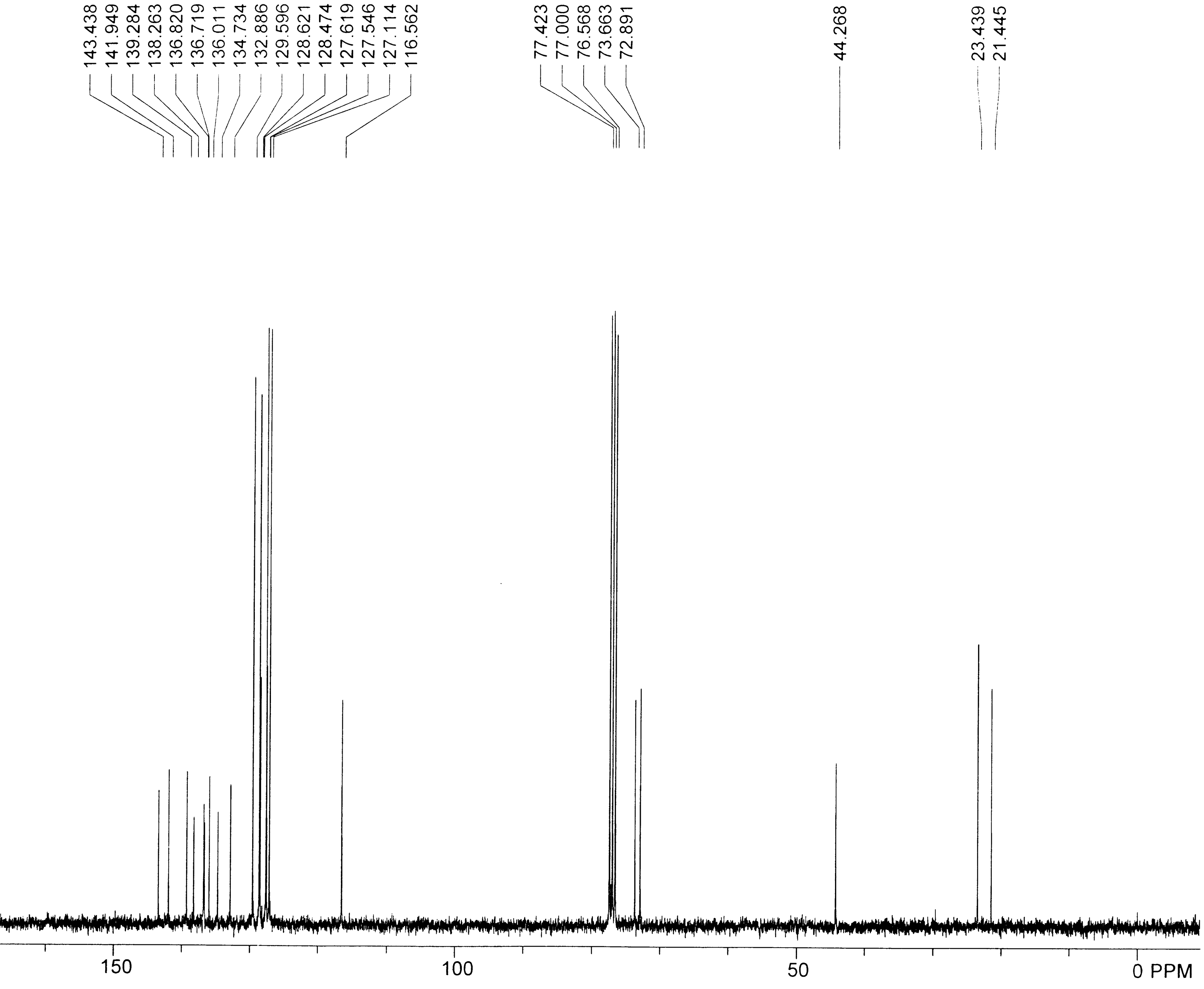

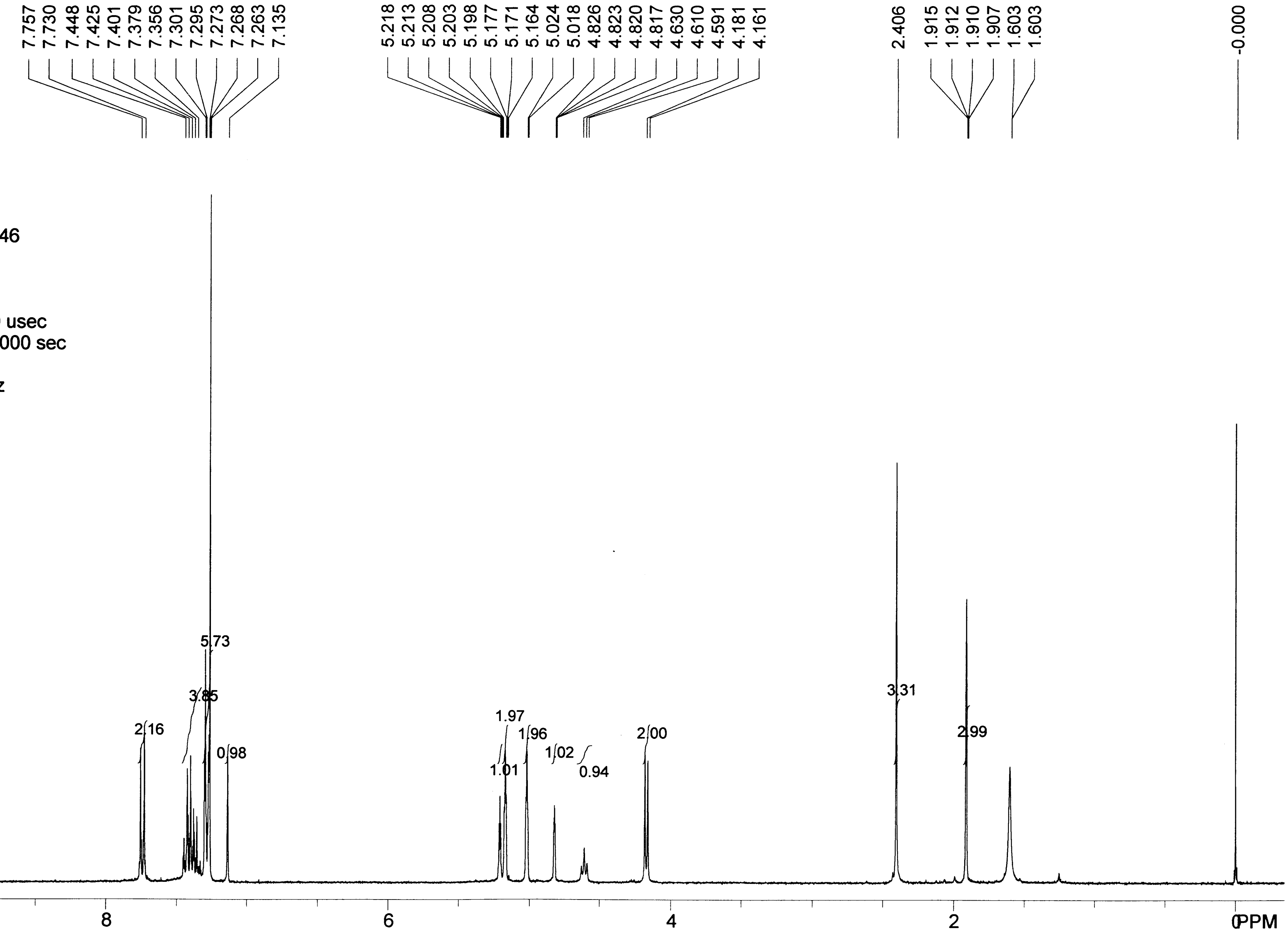
7.757
7.730
7.448
7.425
7.401
7.379
7.356
7.301
7.295
7.273
7.268
7.263
7.135
5.218
5.213
5.208
5.203
5.198
5.177
5.171
5.164
5.024
5.018
4.826
4.823
4.820
4.817
4.630
4.610
4.591
4.181
4.161
2.406
1.915
1.912
1.910
1.907
1.603
1.603
-0.000
2015-07-14 14:34:17.046
wwt-1-193-4r
SOLVENT: CDCl3
Experiment = zg30
Pulse length = 14.000 usec
Relaxation delay = 1.000 sec
NA = 8
F1 = 300.130005 MHz
F2 = 1.000000 MHz
2.16
3.85
5.73
0.98
1.97
1.01
1.96
1.02
0.94
2.00
3.31
2.99
10
8
6
4
2
0
PPM

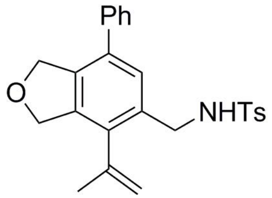
Ph
O
NHTs

5aa

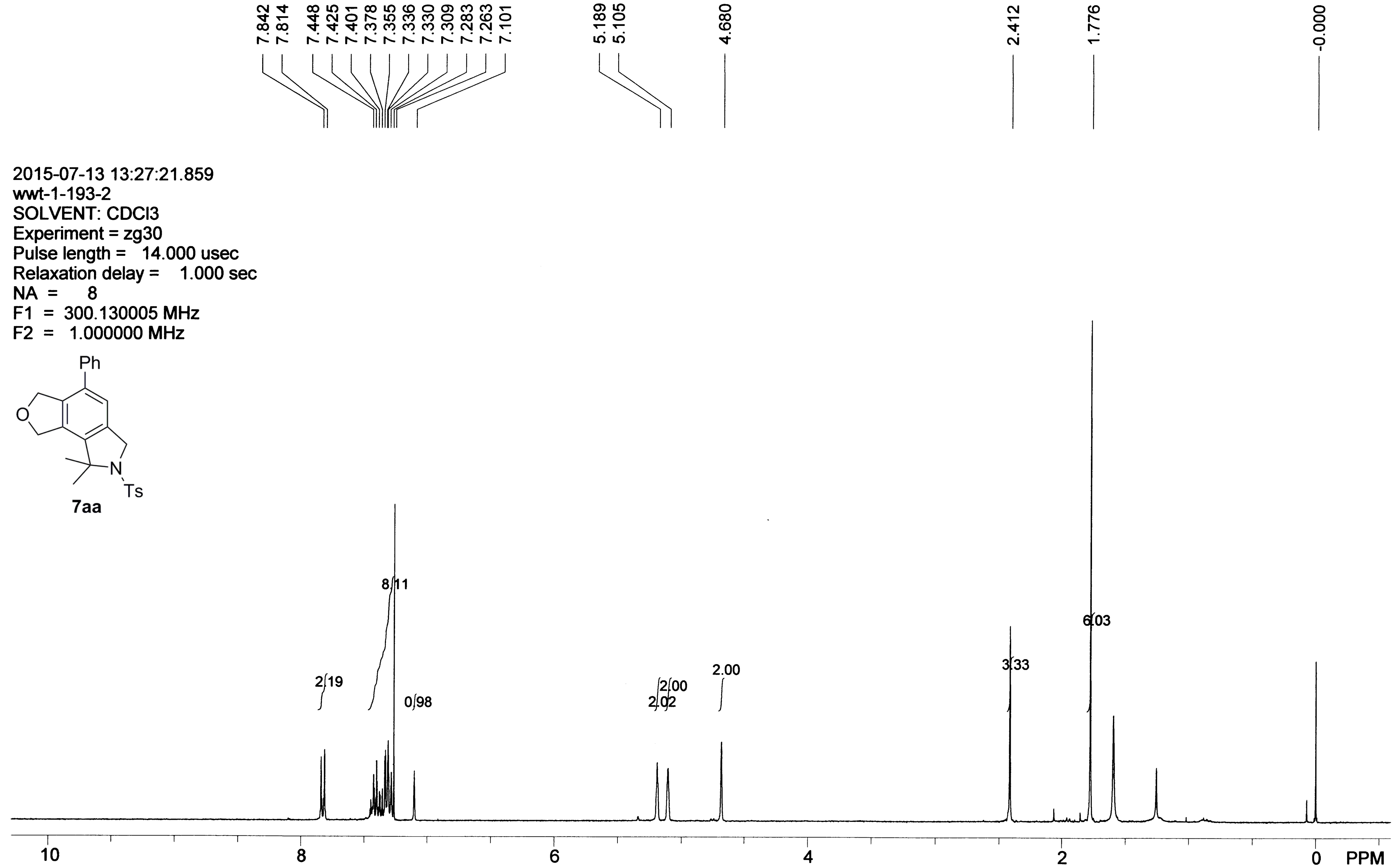

7.842
7.814
7.448
7.425
7.401
7.378
7.355
7.336
7.330
7.309
7.283
7.263
7.101
5.189
5.105
4.680
2.412
1.776
-0.000
2015-07-13 13:27:21.859
wwt-1-193-2
SOLVENT: CDCl3
Experiment = zg30
Pulse length = 14.000 usec
Relaxation delay = 1.000 sec
NA = 8
F1 = 300.130005 MHz
F2 = 1.000000 MHz
Ph
O
N
Ts
7aa
2.19
8.11
0.98
2.00
2.02
2.00
3.33
6.03
10
8
6
4
2
0
PPM

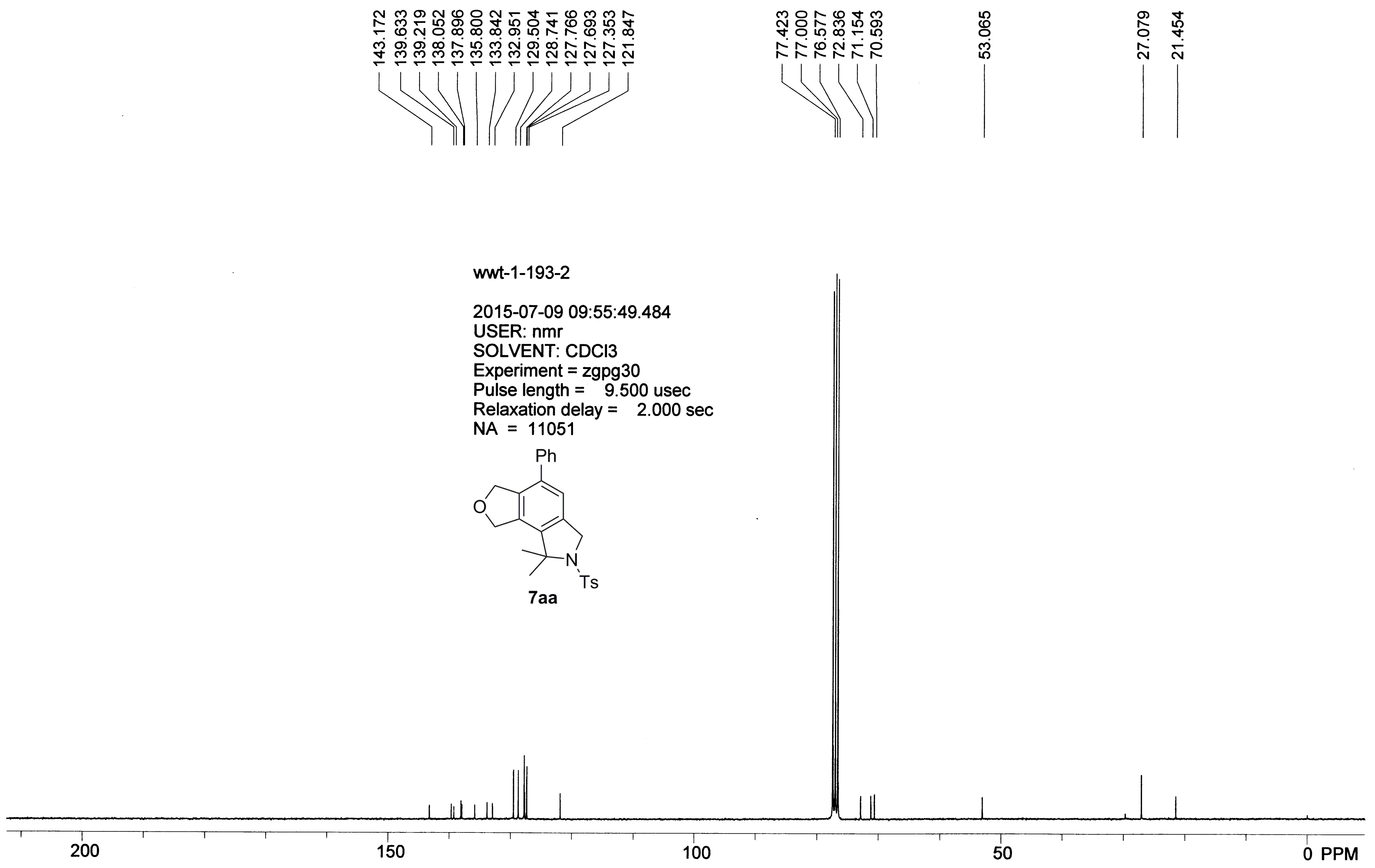
143.172
139.633
139.219
138.052
137.896
135.800
133.842
132.951
129.504
128.741
127.766
127.693
127.353
121.847
77.423
77.000
76.577
72.836
71.154
70.593
53.065
27.079
21.454
wwt-1-193-2
2015-07-09 09:55:49.484
USER: nmr
SOLVENT: CDCl3
Experiment = zgpg30
Pulse length = 9.500 usec
Relaxation delay = 2.000 sec
NA = 11051
Ph
O
N
Ts
7aa
200
150
100
50
0 PPM

2018-03-26 19:34:58.734
zyc-1-166-2
SOLVENT: CDCl3
Experiment = zg30
Pulse length = 14.000 usec
Relaxation delay = 1.000 sec
NA = 8
F1 = 300.130005 MHz
F2 = 1.000000 MHz

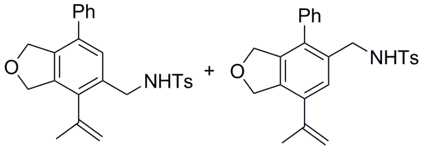

5aa **6aa**

5aa/**6aa** = 97/3

Gram Scale

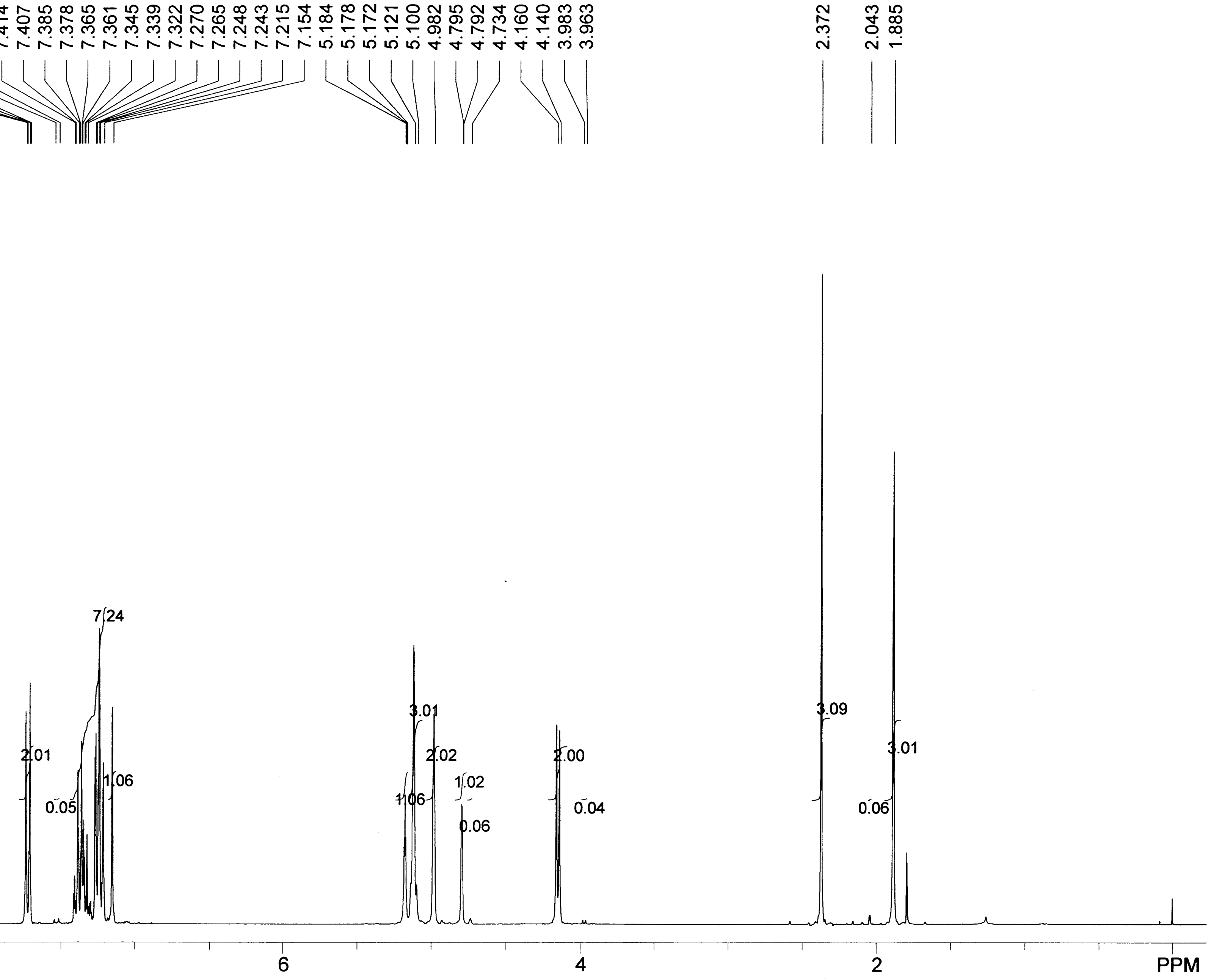

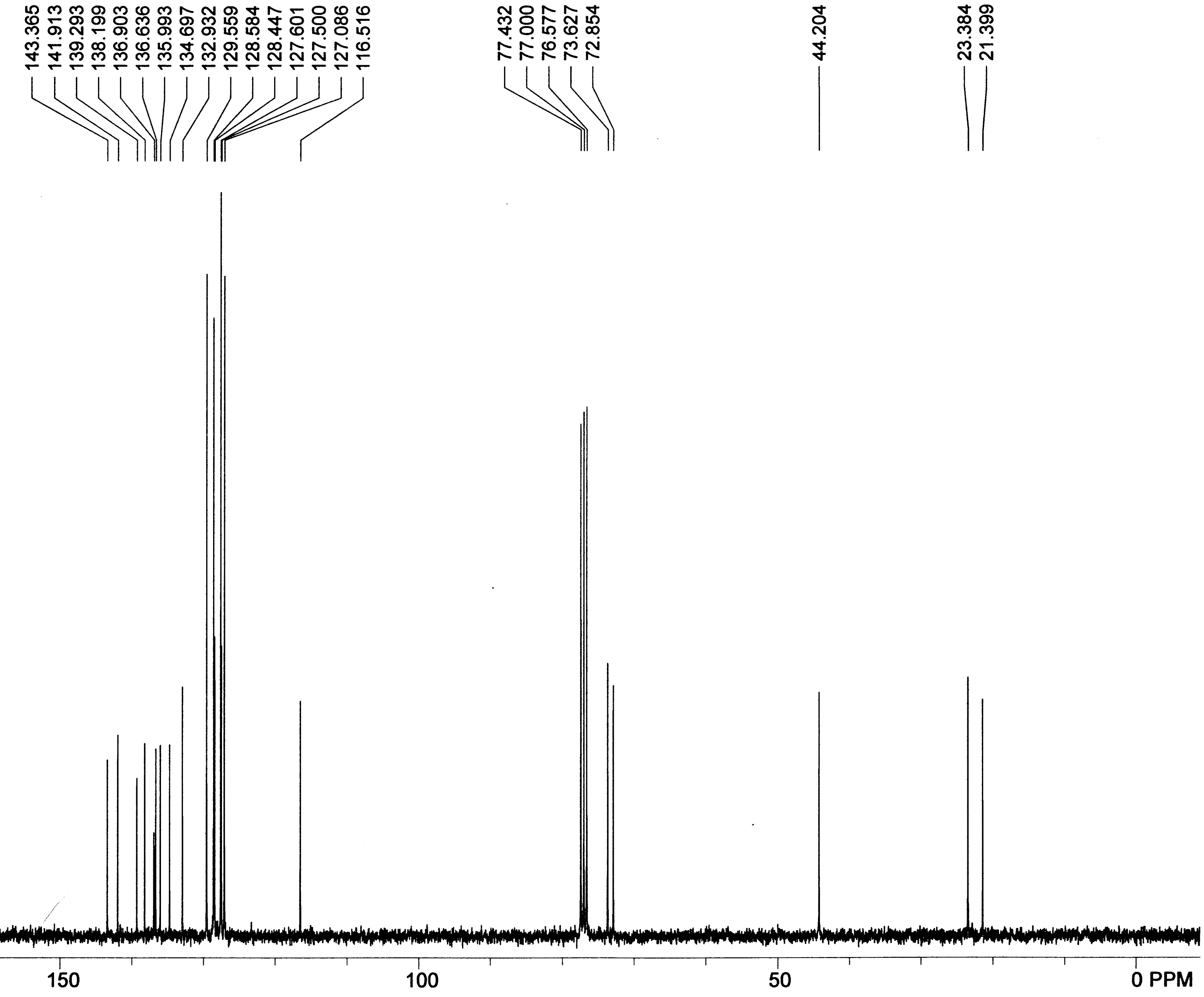

2018-03-26 20:14:33.859
zyc-1-166-2
SOLVENT: CDCl3
Experiment = zgpg30
Pulse length = 9.500 usec
Relaxation delay = 2.000 sec
NA = 401
F1 = 75.467751 MHz
F2 = 1.000000 MHz

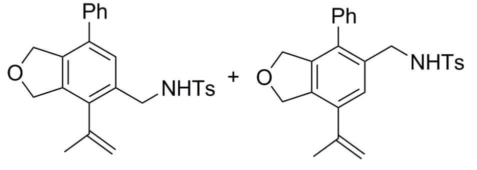

5aa **6aa**

5aa/6aa = 97/3

Gram Scale

2018-03-29 21:26:19.250
zyc-1-166-1
SOLVENT: CDCl3
Experiment = zg30
Pulse length = 14.000 usec
Relaxation delay = 1.000 sec
NA = 8
F1 = 300.130005 MHz
F2 = 1.000000 MHz

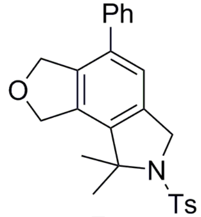

7aa
Gram Scale

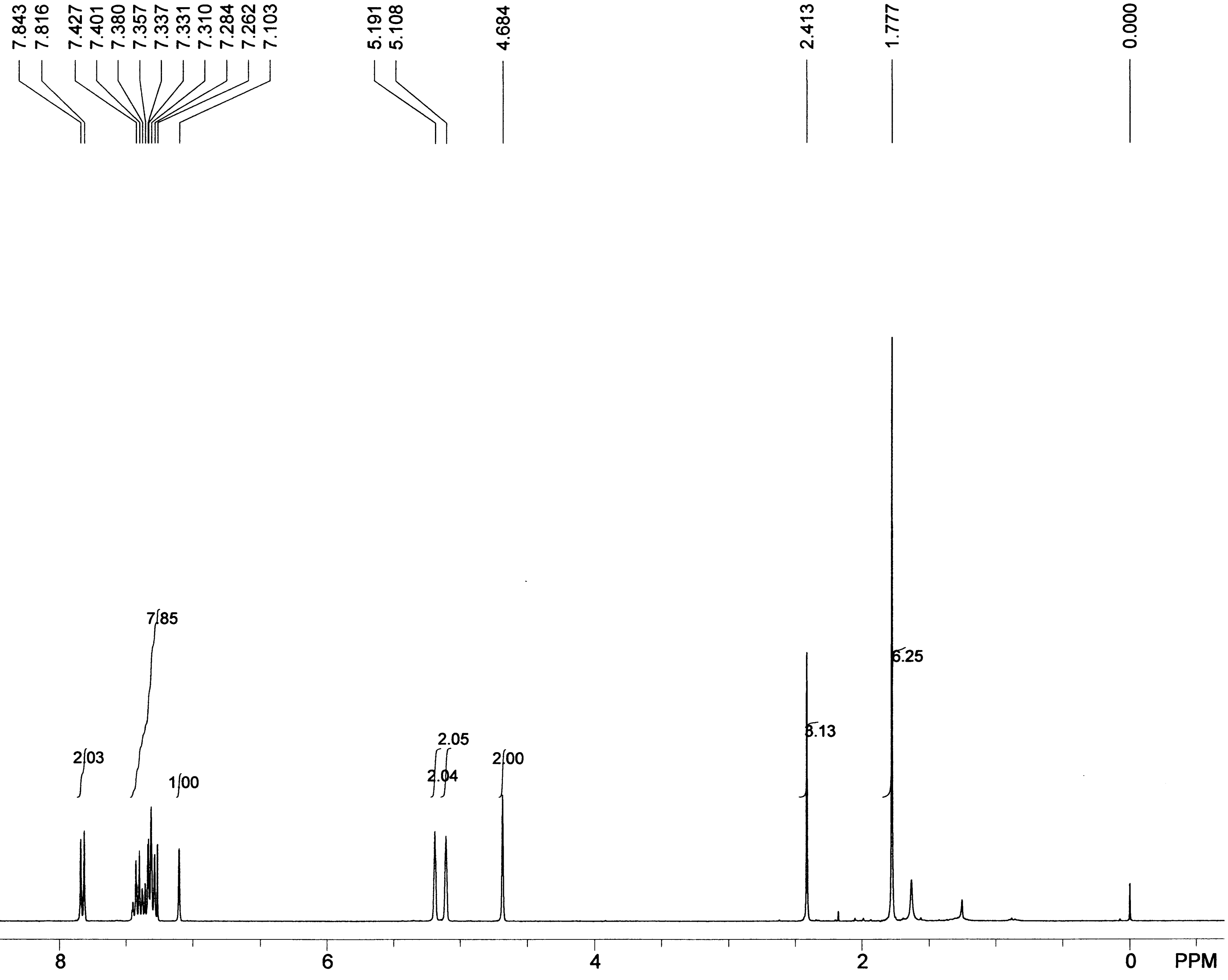

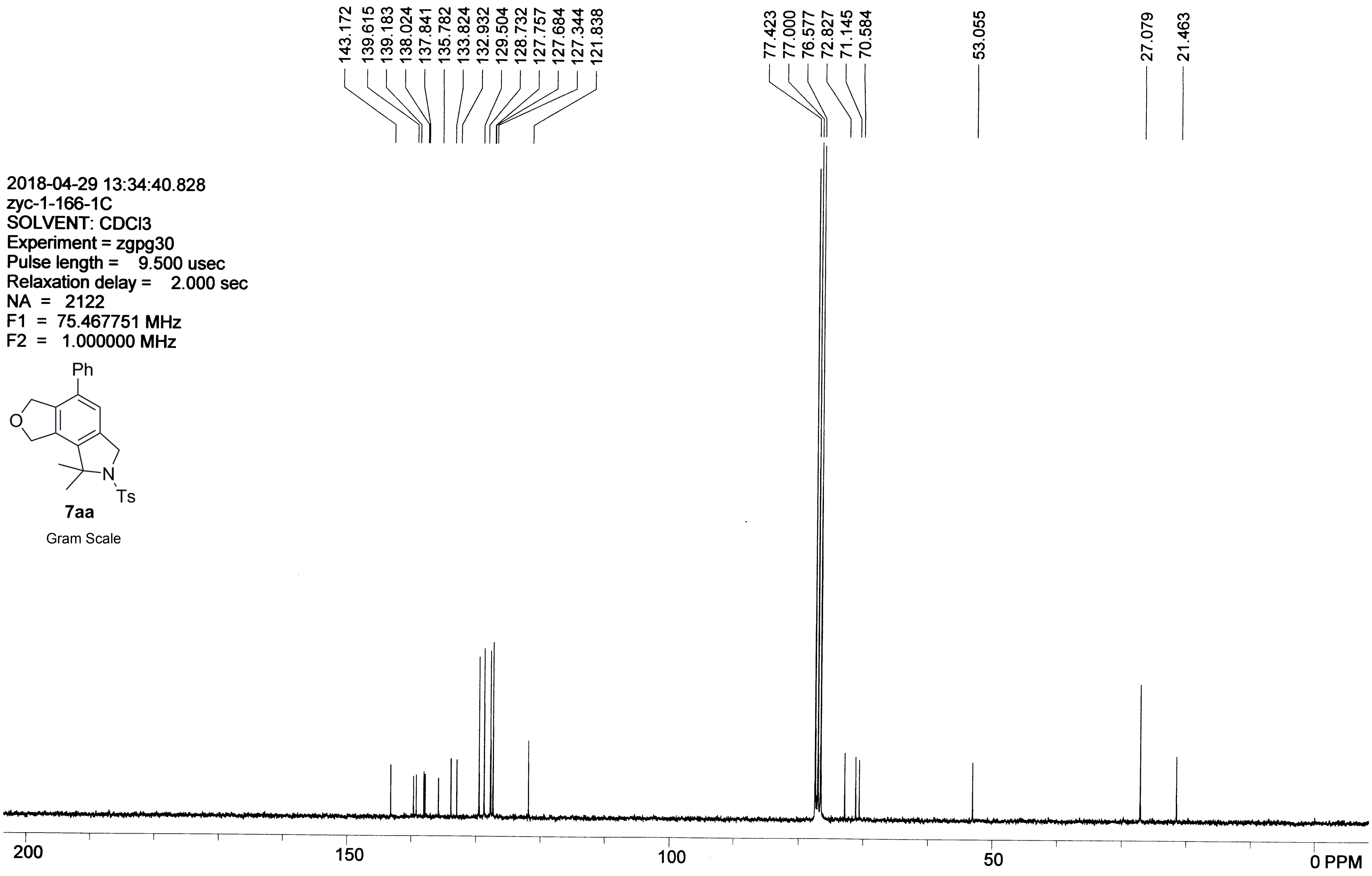

7aa

Gram Scale

2015-06-18 22:46:05.796
wwt-1-189-3H
SOLVENT: CDCl3
Experiment = zg30
Pulse length = 14.000 usec
Relaxation delay = 1.000 sec
NA = 8
F1 = 300.130005 MHz
F2 = 1.000000 MHz

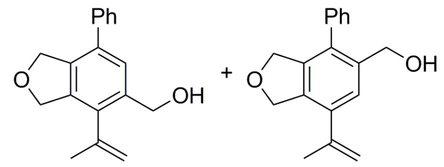

5ab **6ab**

5ab/6ab = 97/3

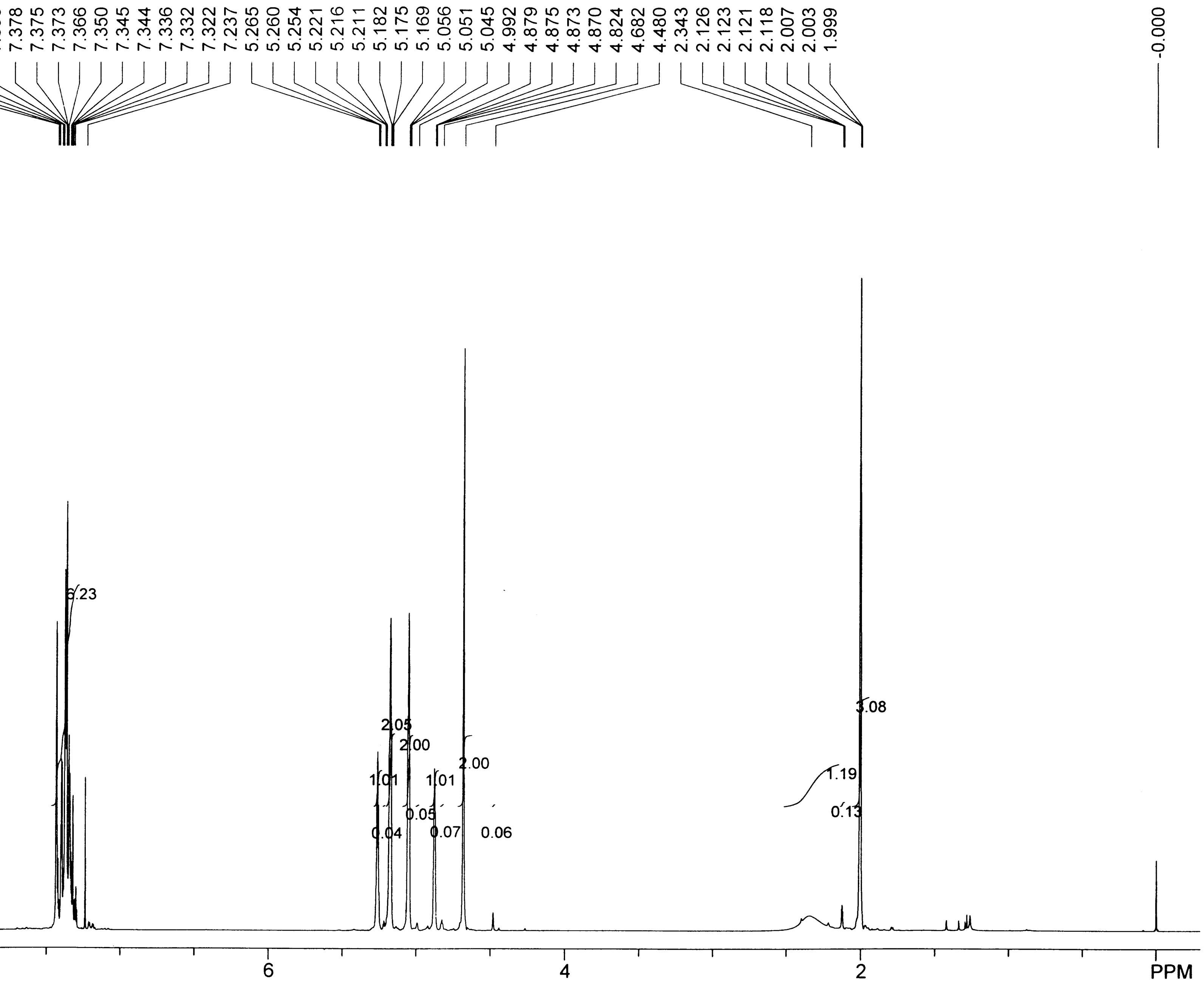

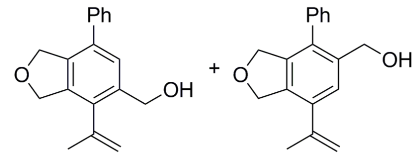

5ab 6ab

5ab/6ab = 97/3

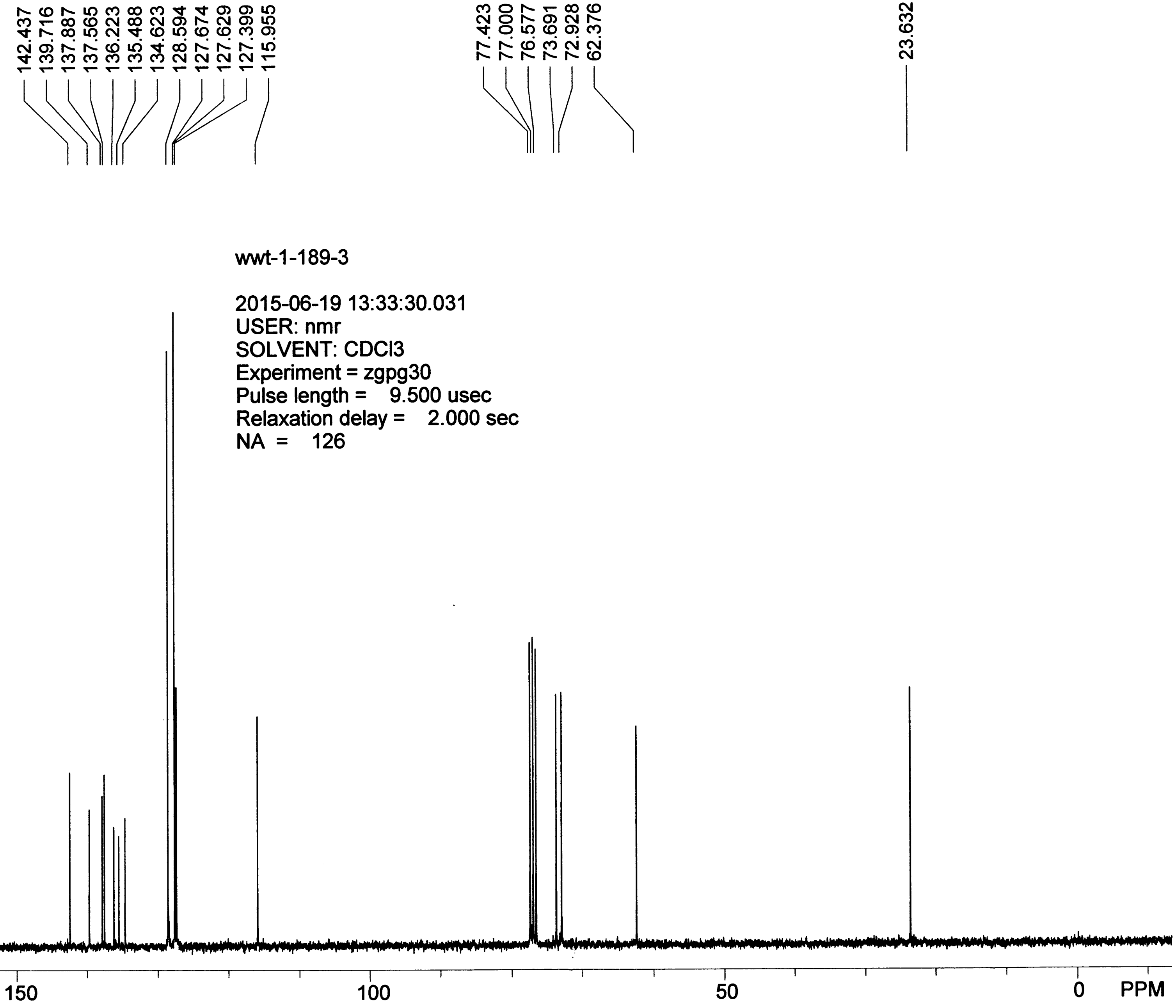

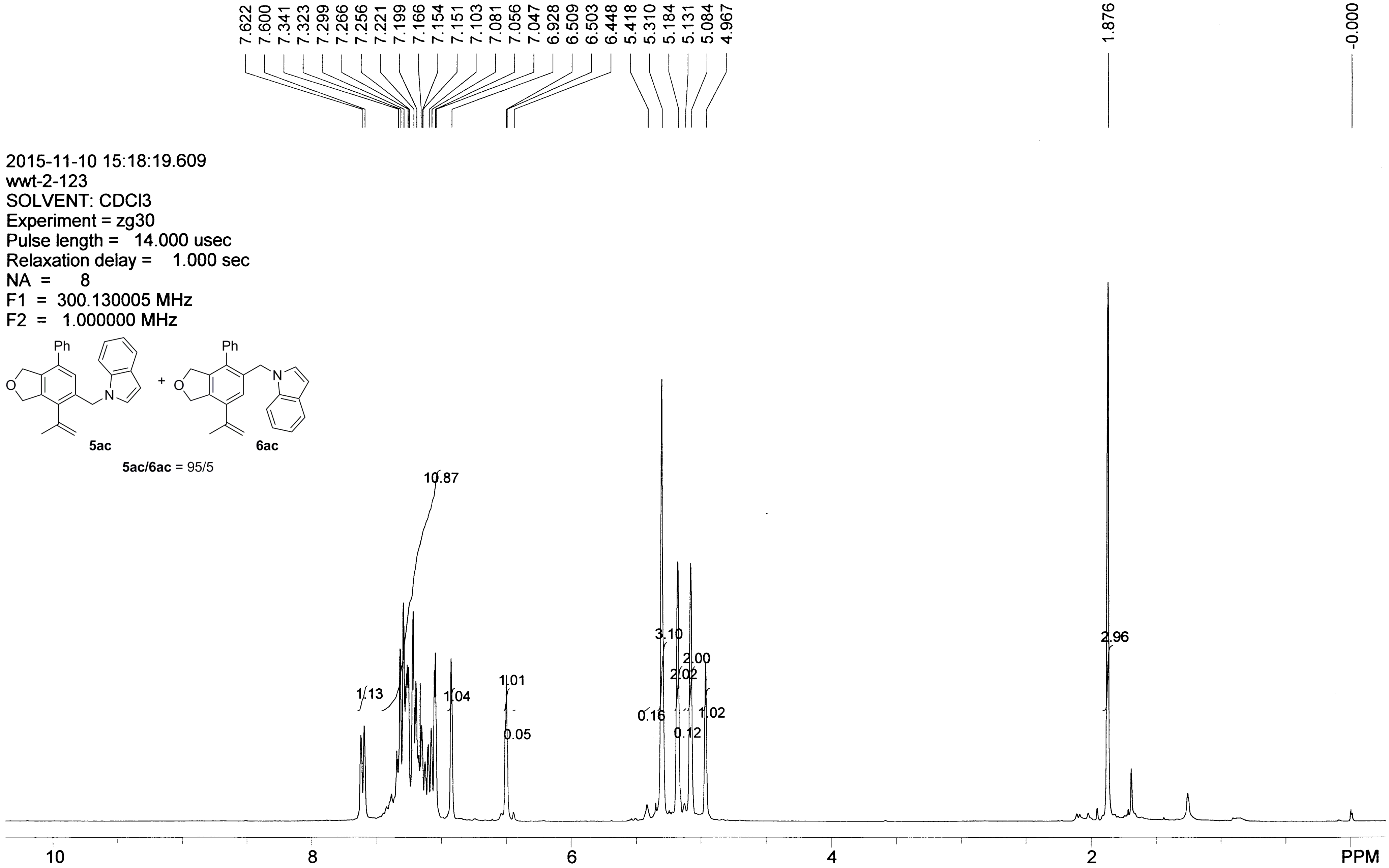
2015-11-10 15:18:19.609
wwt-2-123
SOLVENT: CDCl3
Experiment = zg30
Pulse length = 14.000 usec
Relaxation delay = 1.000 sec
NA = 8
F1 = 300.130005 MHz
F2 = 1.000000 MHz
Ph
Ph
5ac
6ac
5ac/6ac = 95/5
7.622 7.600 7.341 7.323 7.299 7.266 7.256 7.221 7.199 7.166 7.154 7.151 7.103 7.081 7.056 7.047 6.928 6.509 6.503 6.448 5.418 5.310 5.184 5.131 5.084 4.967
1.876
-0.000
10.87
1.13
1.04
1.01
0.05
3.10
2.00
2.02
0.16
1.02
0.12
2.96
10
8
6
4
2
PPM

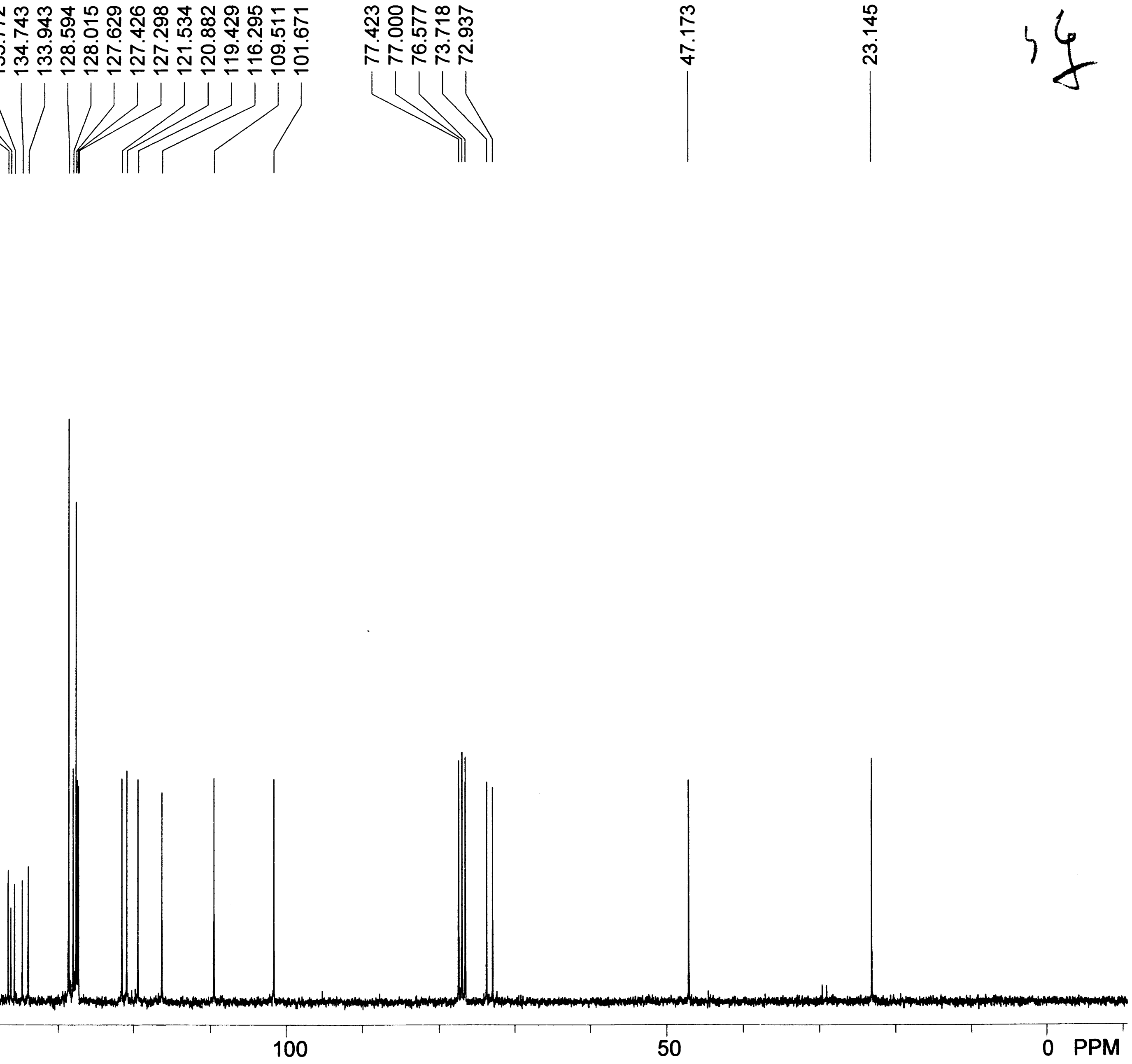

wwt-2-123

2015-11-10 15:31:56.109
USER: nmr
SOLVENT: CDCl3
Experiment = zgpg30
Pulse length = 9.500 usec
Relaxation delay = 2.000 sec
NA = 191

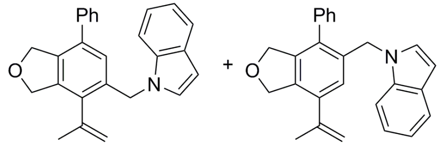

5ac **6ac**

5ac/6ac = 95/5

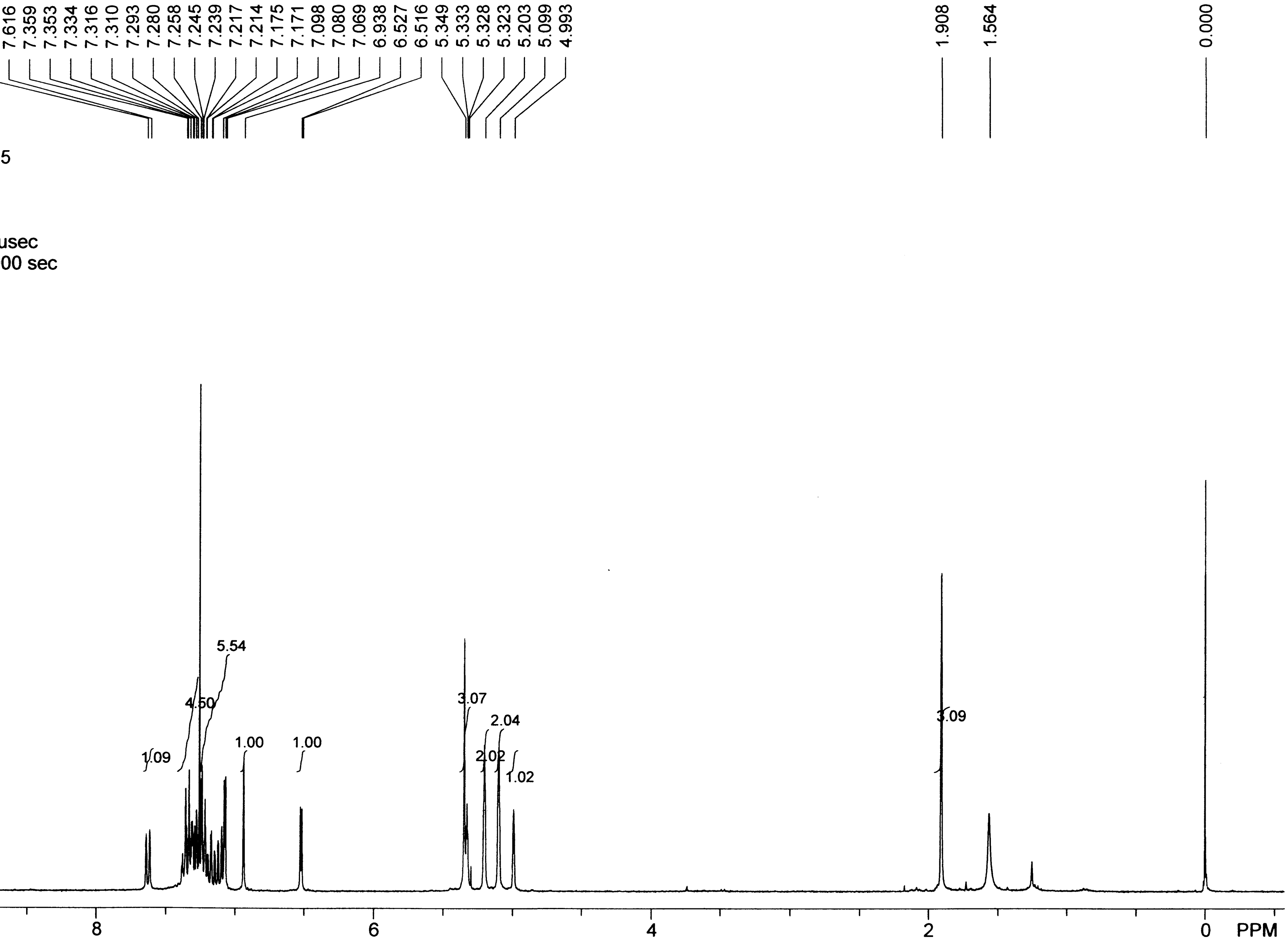
7.641
7.616
7.359
7.353
7.334
7.316
7.310
7.293
7.280
7.258
7.245
7.239
7.217
7.214
7.175
7.171
7.098
7.080
7.069
6.938
6.527
6.516
5.349
5.333
5.328
5.323
5.203
5.099
4.993
1.908
1.564
0.000
2017-10-25 11:55:31.125
zyc-1-68re
SOLVENT: CDCl3
Experiment = zg30
Pulse length = 14.000 usec
Relaxation delay = 1.000 sec
NA = 40
F1 = 300.130005 MHz
F2 = 1.000000 MHz
5.54
4.50
1.09
1.00
1.00
3.07
2.04
2.02
1.02
3.09
10
8
6
4
2
0
PPM

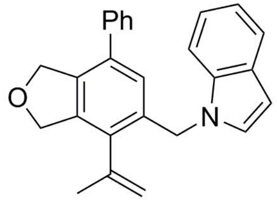
Ph
O
N

5ac

2017-12-11 16:02:16.890
zyc-1-102H
SOLVENT: CDCl3
Experiment = zg30
Pulse length = 14.000 usec
Relaxation delay = 1.000 sec
NA = 8
Solvent = CDCl3

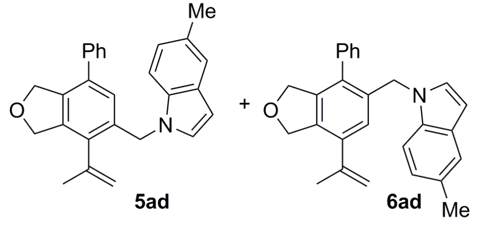

5ad + **6ad**

5ad/6ad = 95/5

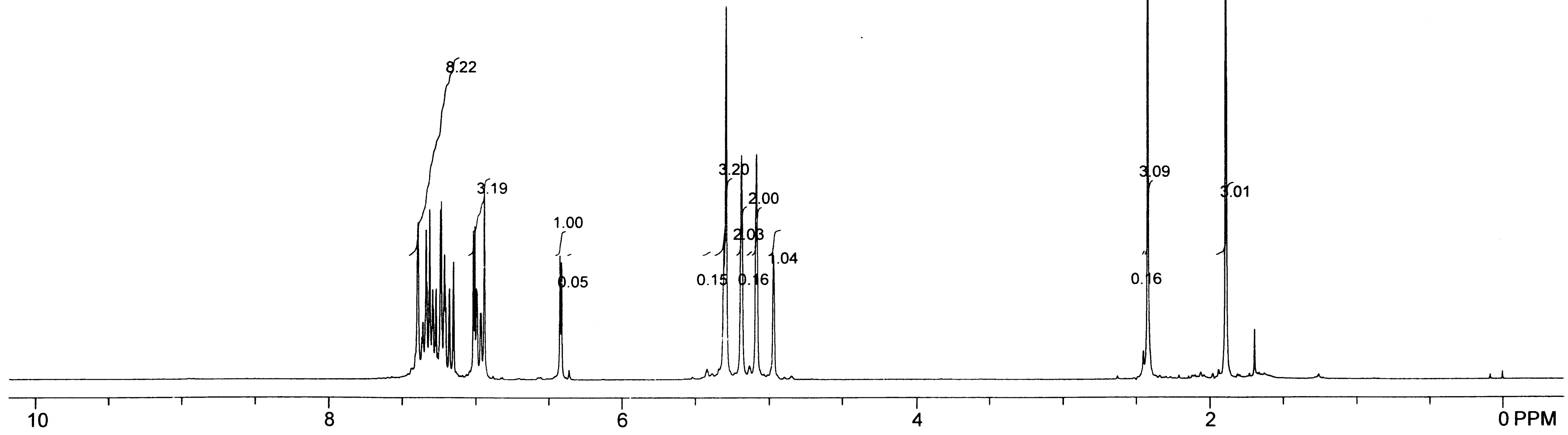

2017-12-11 16:17:09.843
zyc-1-102C
SOLVENT: CDCl3
Experiment = zgpg30
Pulse length = 9.500 usec
Relaxation delay = 2.000 sec
NA = 210
F1 = 75.467751 MHz
F2 = 1.000000 MHz

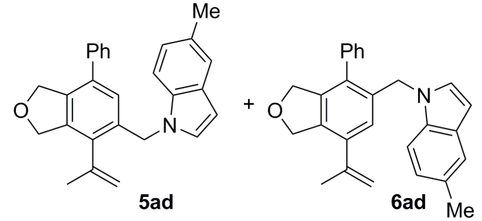

5ad **6ad**

5ad/6ad = 95/5

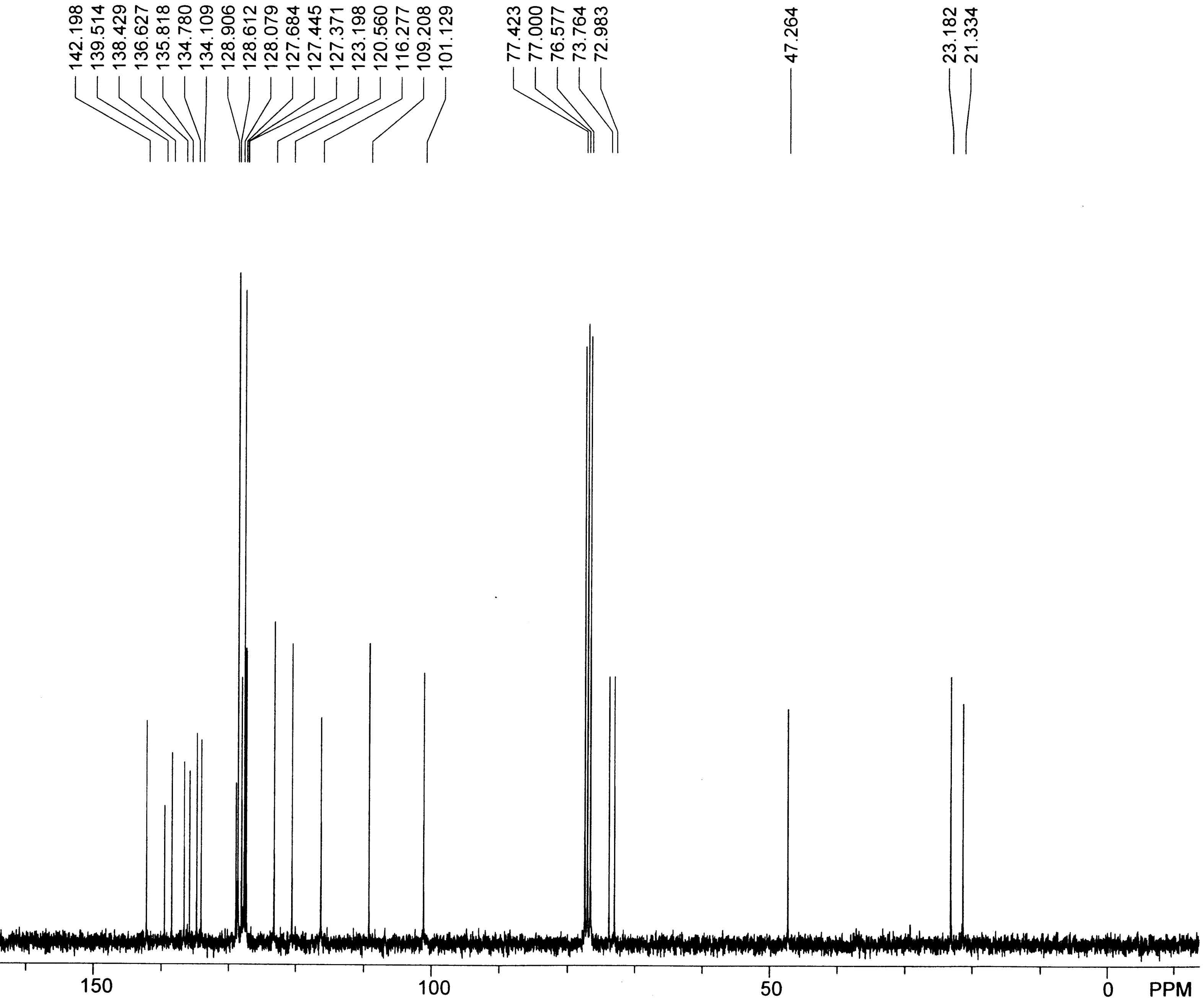

2018-01-25 11:18:11.218
zyc-1-102RE
SOLVENT: CDCl3
Experiment = zg30
Pulse length = 14.000 usec
Relaxation delay = 1.000 sec
NA = 8
F1 = 300.130005 MHz
F2 = 1.000000 MHz

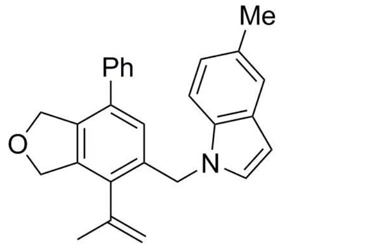

5ad

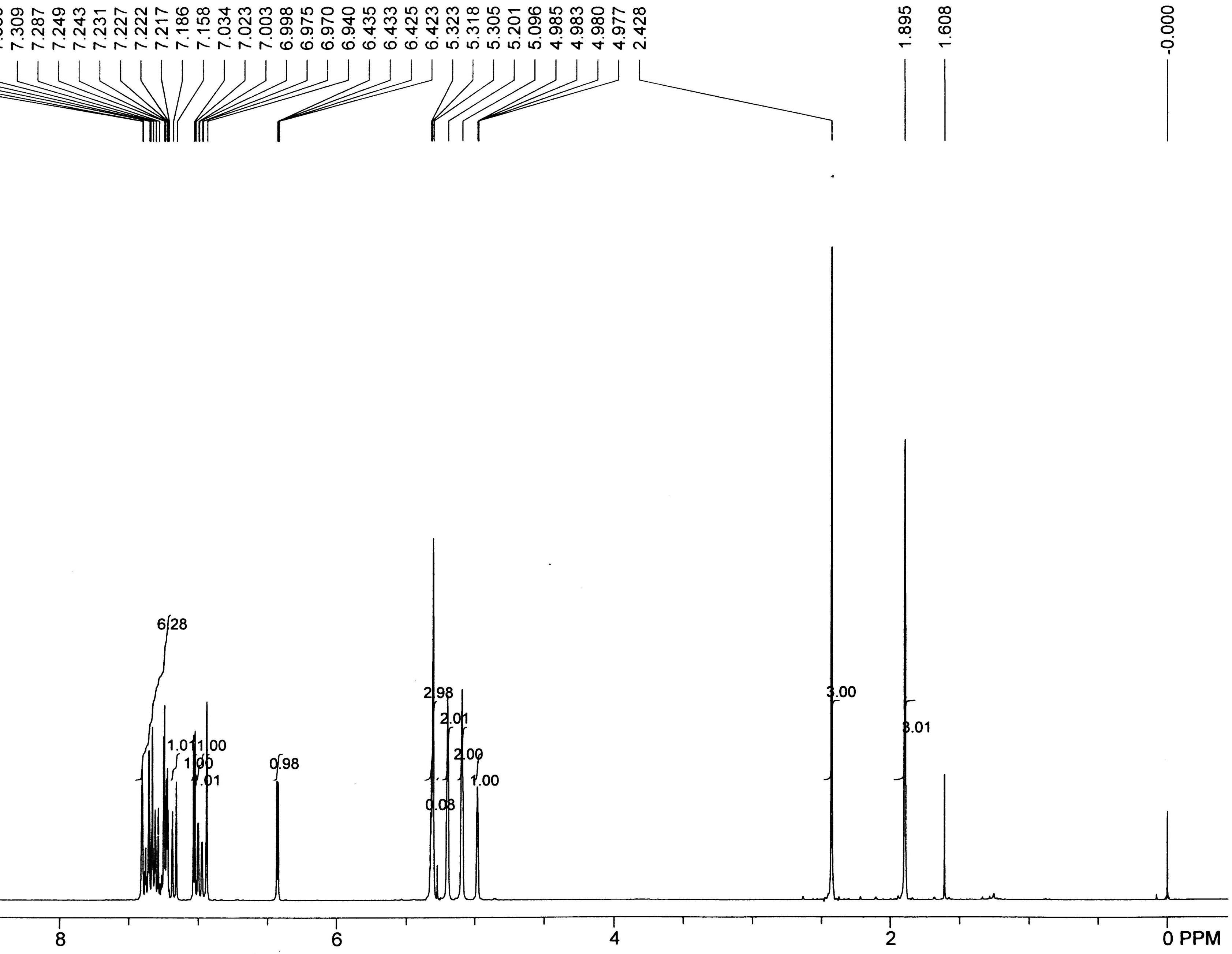

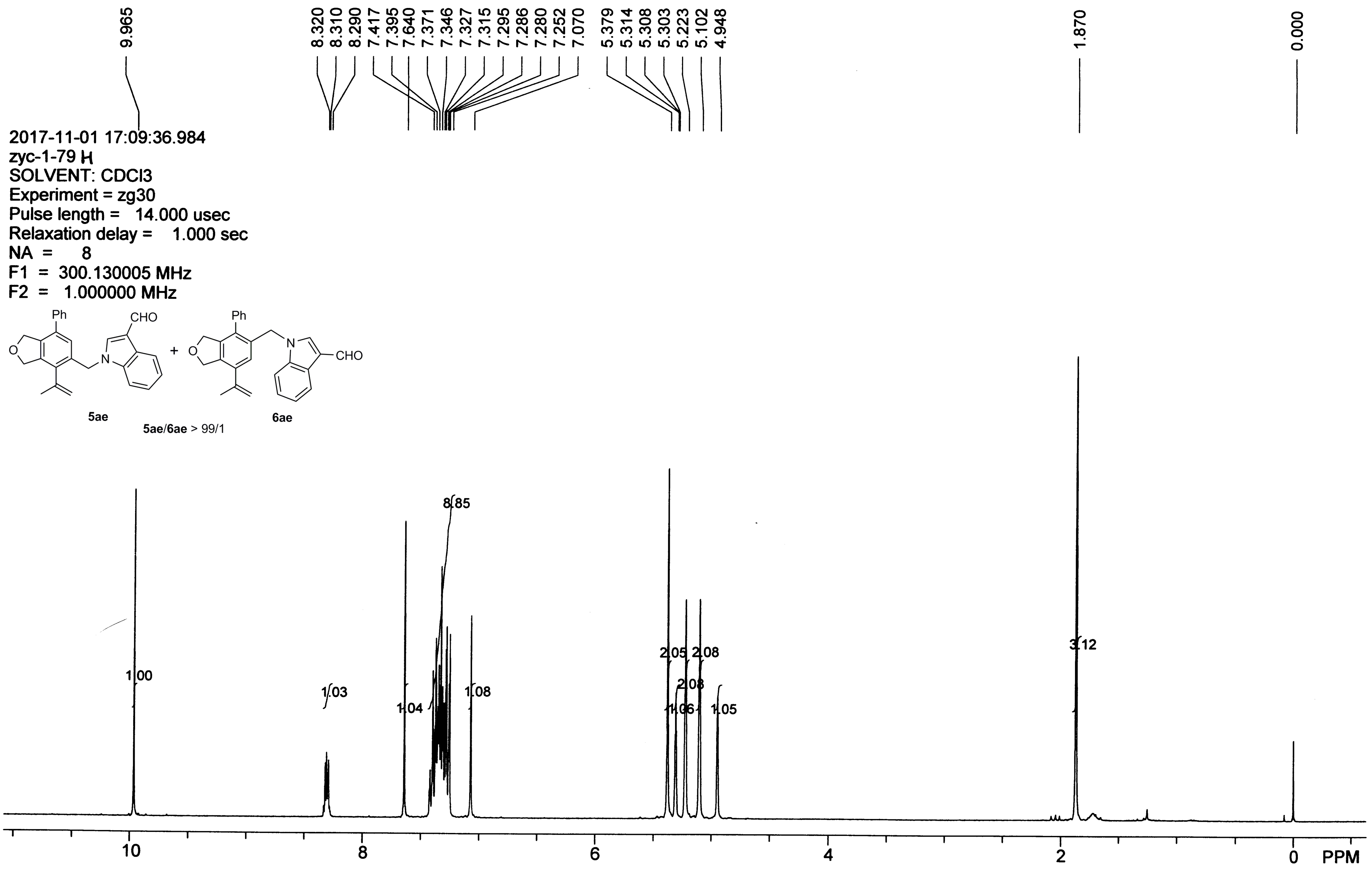

9.965
8.320
8.310
8.290
7.417
7.395
7.640
7.371
7.346
7.327
7.315
7.295
7.286
7.280
7.252
7.070
5.379
5.314
5.308
5.303
5.223
5.102
4.948
1.870
0.000
2017-11-01 17:09:36.984
zyc-1-79 H
SOLVENT: CDCl3
Experiment = zg30
Pulse length = 14.000 usec
Relaxation delay = 1.000 sec
NA = 8
F1 = 300.130005 MHz
F2 = 1.000000 MHz
Ph
CHO
5ae
6ae
5ae/6ae > 99/1
1.00
1.03
1.04
8.85
1.08
2.05
2.08
2.08
1.06
1.05
3.12
10
8
6
4
2
0
PPM

2017-11-01 19:44:12.125
zyc-1-79C
SOLVENT: CDCl3
Experiment = zgpg30
Pulse length = 9.500 usec
Relaxation delay = 2.000 sec
NA = 1092
F1 = 75.467751 MHz
F2 = 1.000000 MHz

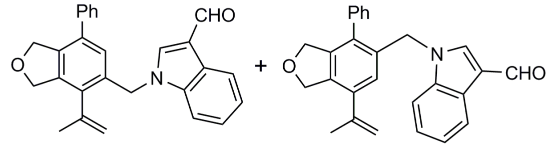

5ae **6ae**

5ae/6ae > 99/1

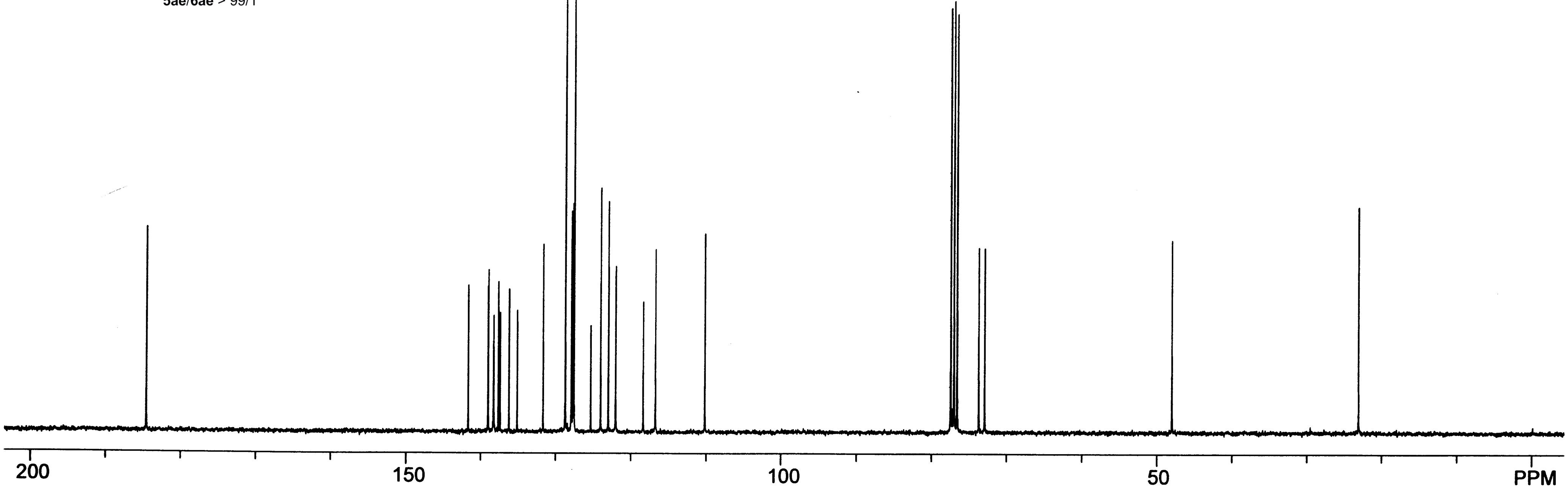

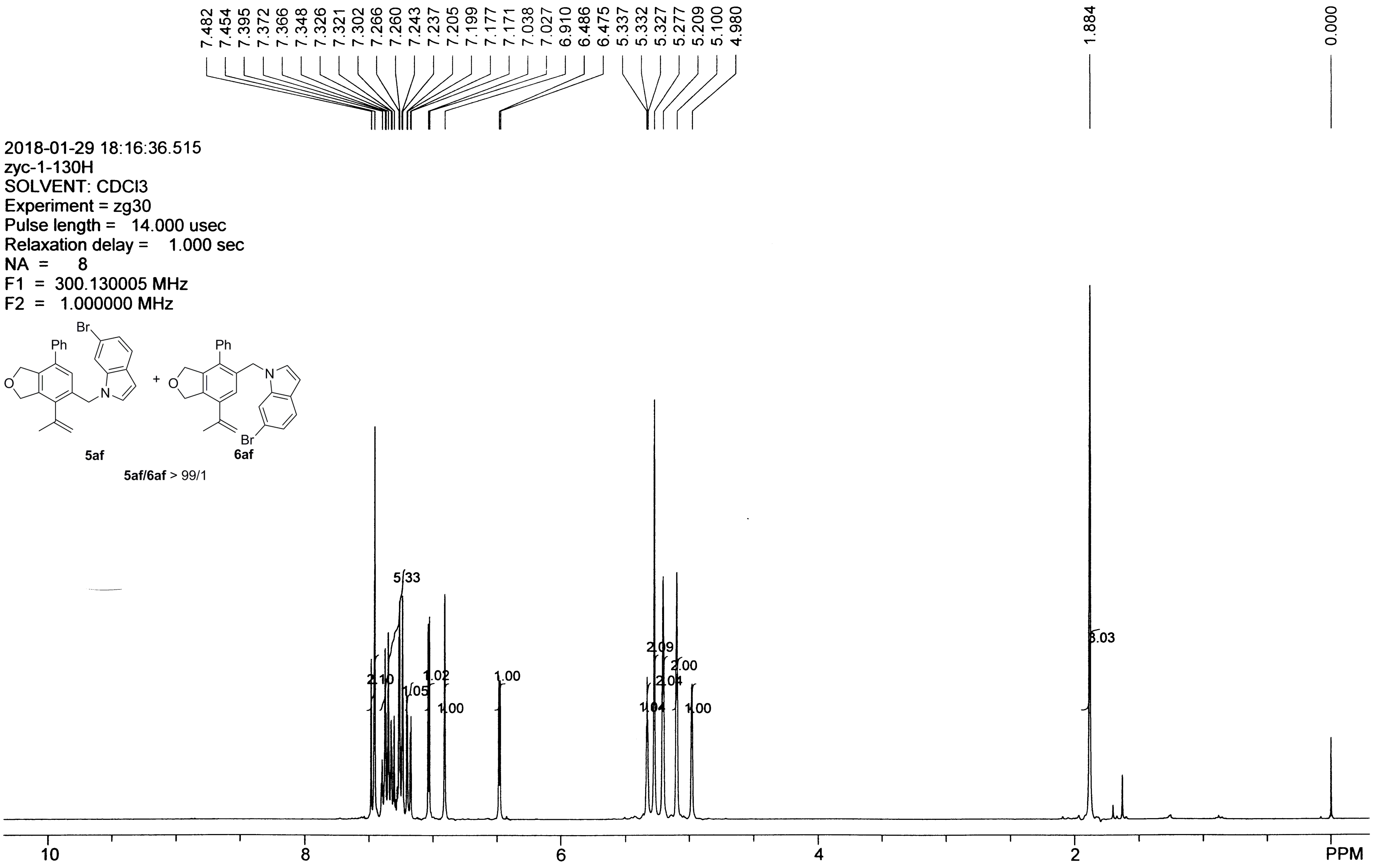
7.482
7.454
7.395
7.372
7.366
7.348
7.326
7.321
7.302
7.266
7.260
7.243
7.237
7.205
7.199
7.177
7.171
7.038
7.027
6.910
6.486
6.475
5.337
5.332
5.327
5.277
5.209
5.100
4.980
1.884
0.000
2018-01-29 18:16:36.515
zyc-1-130H
SOLVENT: CDCl3
Experiment = zg30
Pulse length = 14.000 usec
Relaxation delay = 1.000 sec
NA = 8
F1 = 300.130005 MHz
F2 = 1.000000 MHz
Ph
Br
N
O
+
5af
6af
5af/6af > 99/1
5.33
2.10
1.05
1.02
1.00
1.00
2.09
2.00
2.04
1.04
1.00
3.03
10
8
6
4
2
PPM

2018-01-29 18:41:36.671
zyc-1-130C
SOLVENT: CDCl3
Experiment = zgpg30
Pulse length = 9.500 usec
Relaxation delay = 2.000 sec
NA = 383
F1 = 75.467751 MHz
F2 = 1.000000 MHz

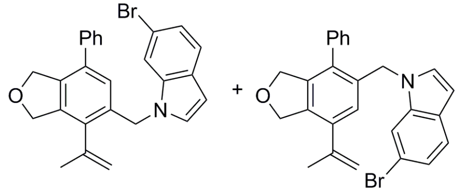

5af **6af**

5af/6af > 99/1

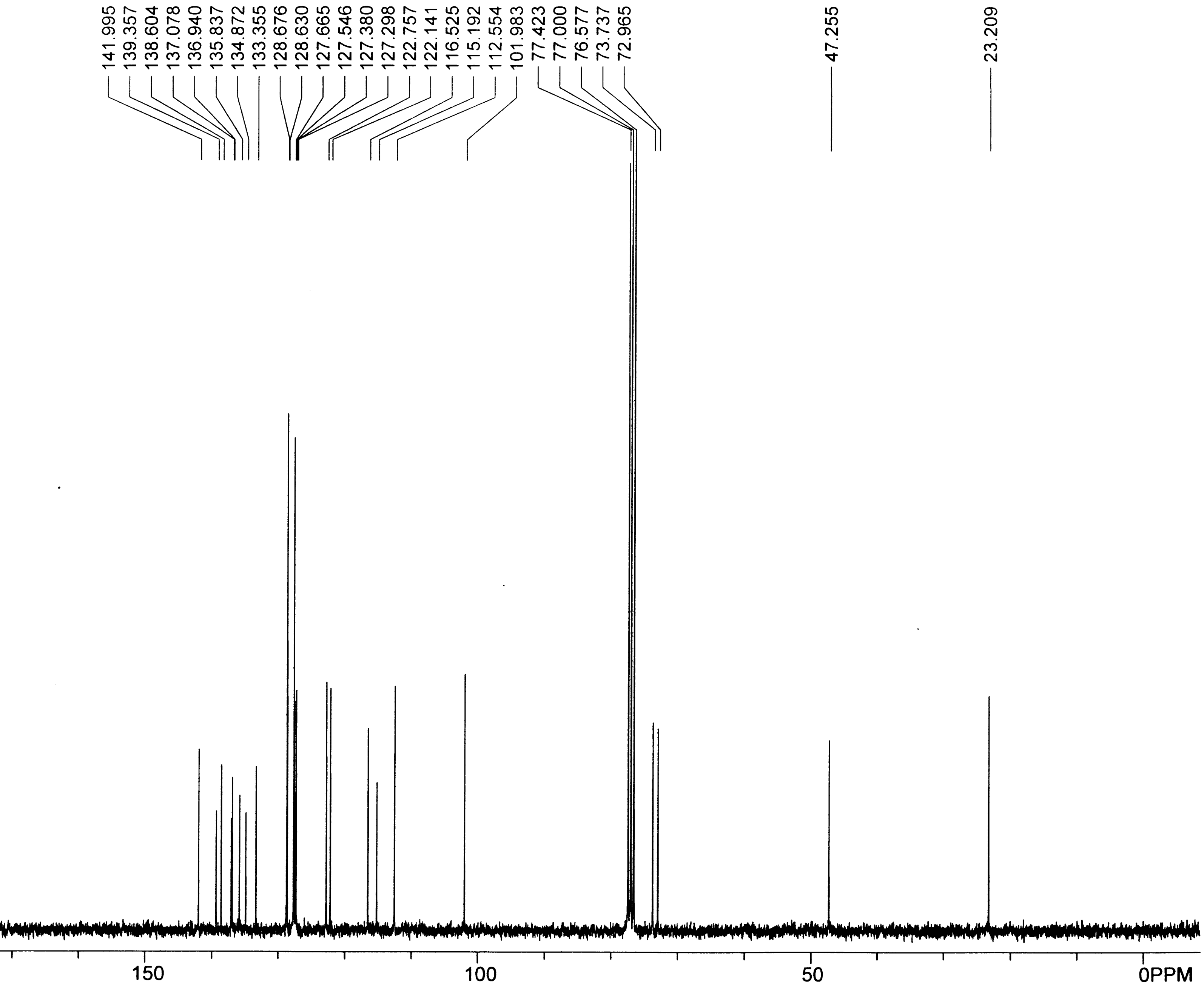

2015-11-10 15:36:28.781
wwt-2-130H
SOLVENT: CDCl3
Experiment = zg30
Pulse length = 14.000 usec
Relaxation delay = 1.000 sec
NA = 8
F1 = 300.130005 MHz
F2 = 1.000000 MHz

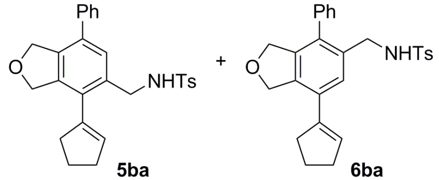

5ba 6ba

5ba/6ba = 98/2

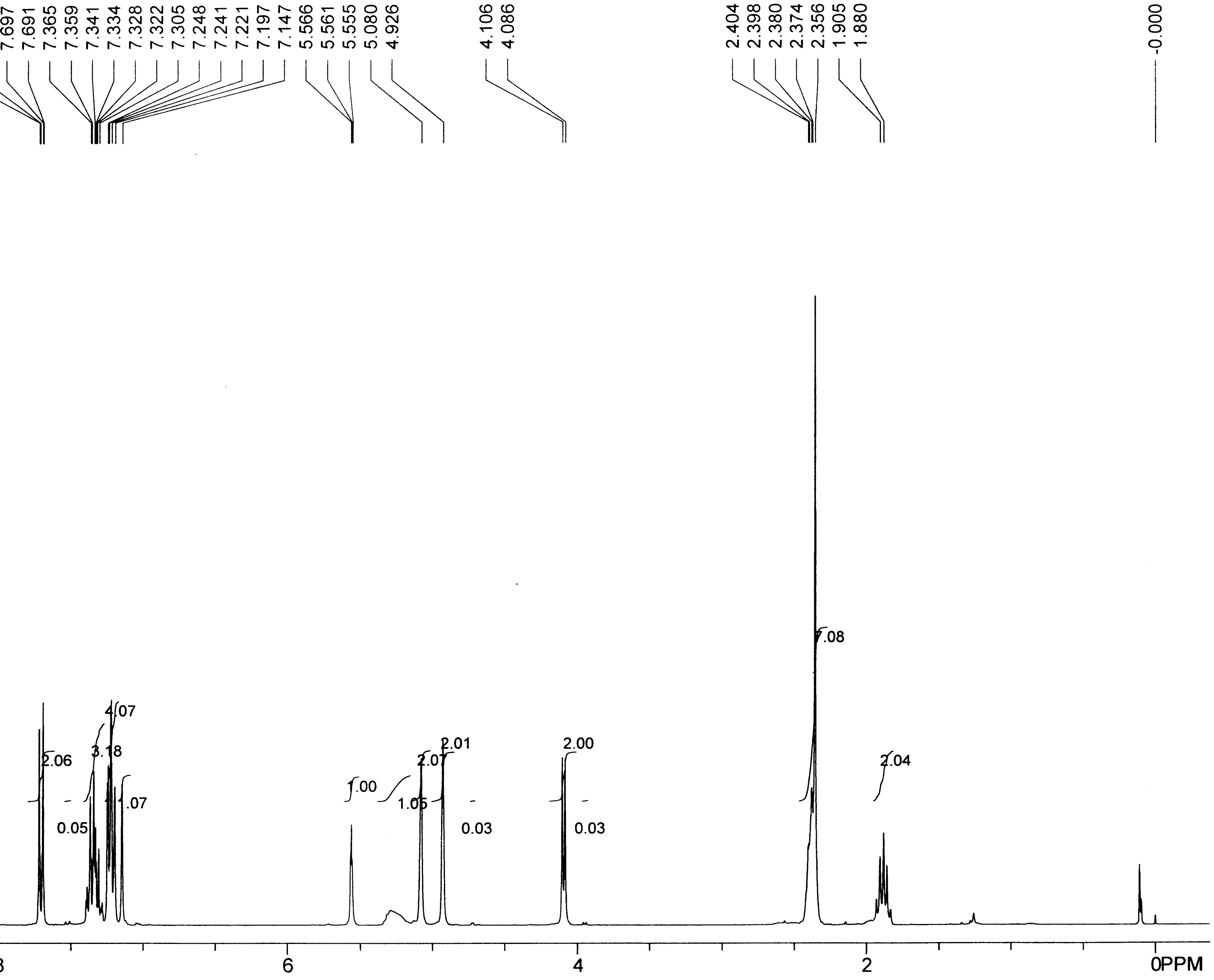

2015-11-10 15:14:08.968
wwt-2-130C
SOLVENT: CDCl3
Experiment = zgpg30
Pulse length = 9.500 usec
Relaxation delay = 2.000 sec
NA = 129
F1 = 75.467751 MHz
F2 = 1.000000 MHz

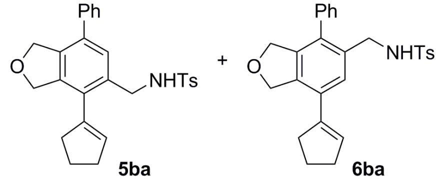

5ba **6ba**

5ba/6ba = 98/2

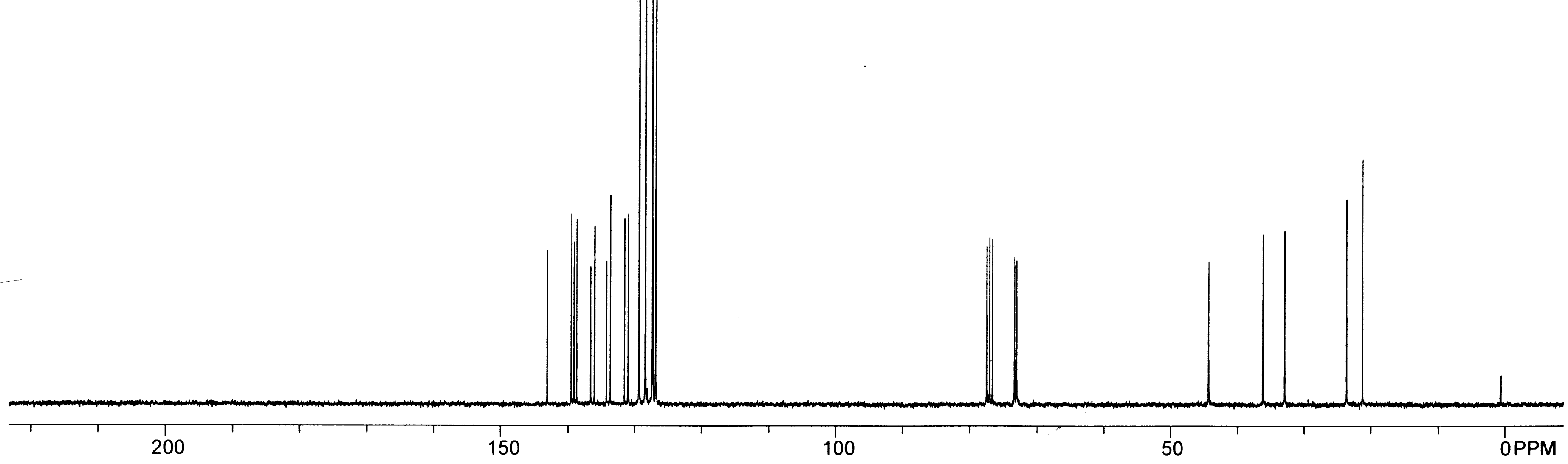

2015-11-11 21:45:40.765
wwt-2-130re
SOLVENT: CDCl3
Experiment = zg30
Pulse length = 14.000 usec
Relaxation delay = 1.000 sec
NA = 8
F1 = 300.130005 MHz
F2 = 1.000000 MHz

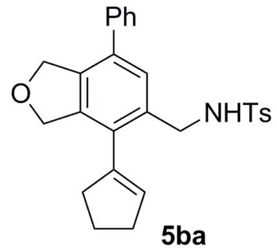

5ba

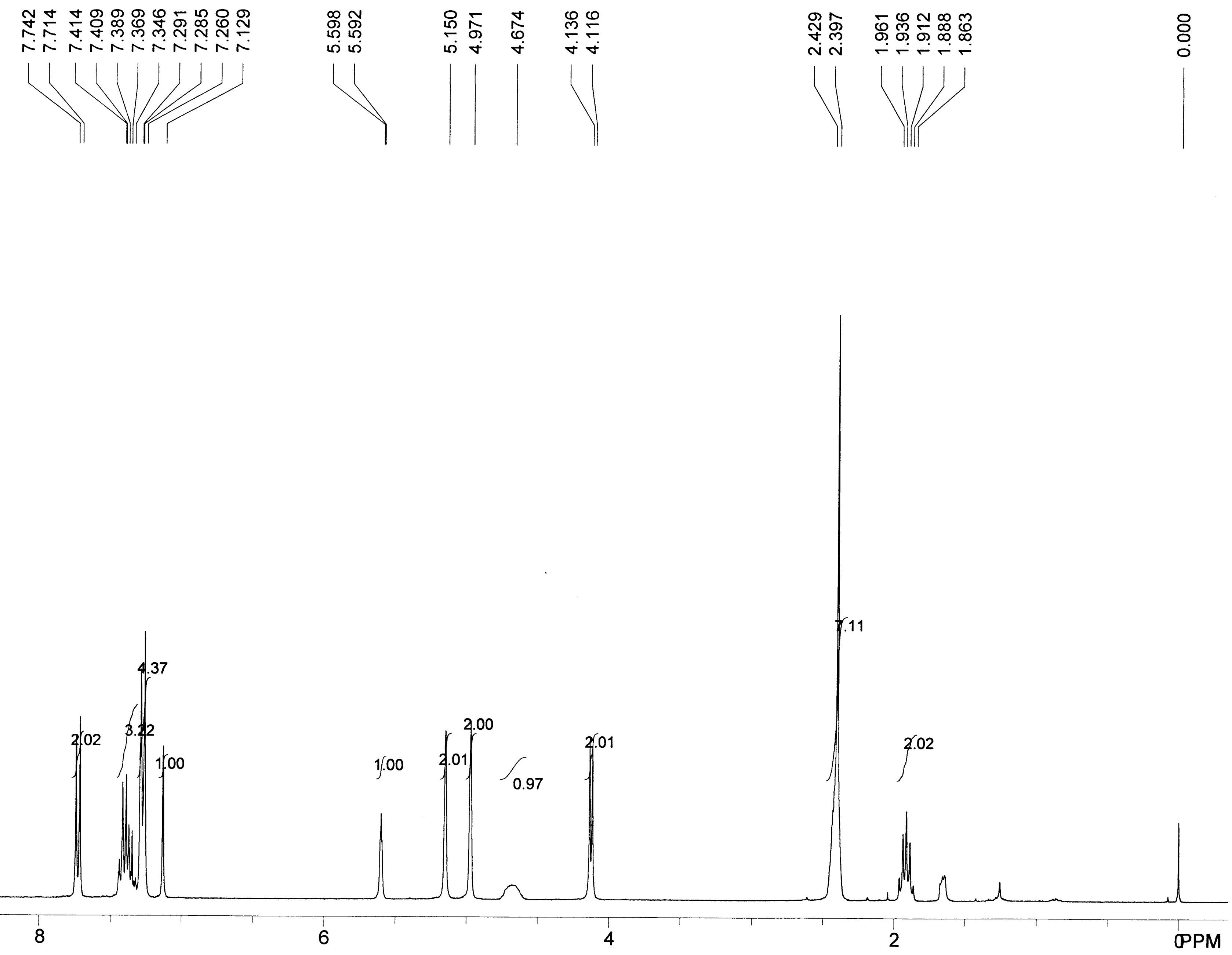

2018-11-26 11:20:24.718
zyc-3-38H
SOLVENT: CDCl3
Experiment = zg30
Pulse length = 14.000 usec
Relaxation delay = 1.000 sec
NA = 8
F1 = 300.130005 MHz
F2 = 1.000000 MHz

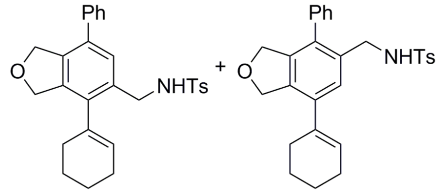

5ja **6ja**

5ja/6ja = 90/10

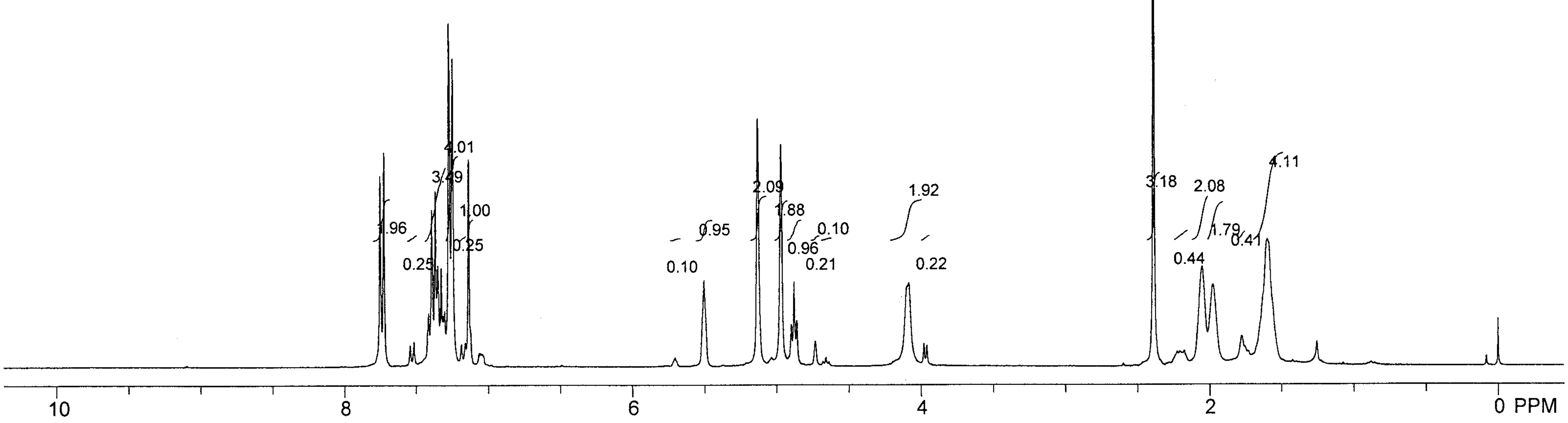

2018-11-26 12:50:56.453
zyc-3-38C
SOLVENT: CDCl3
Experiment = zgpg30
Pulse length = 9.500 usec
Relaxation delay = 2.000 sec
NA = 1520
F1 = 75.467751 MHz
F2 = 1.000000 MHz

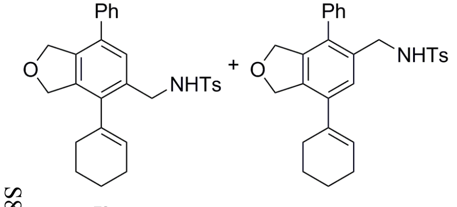

5ja **6ja**

5ja/6ja = 90/10

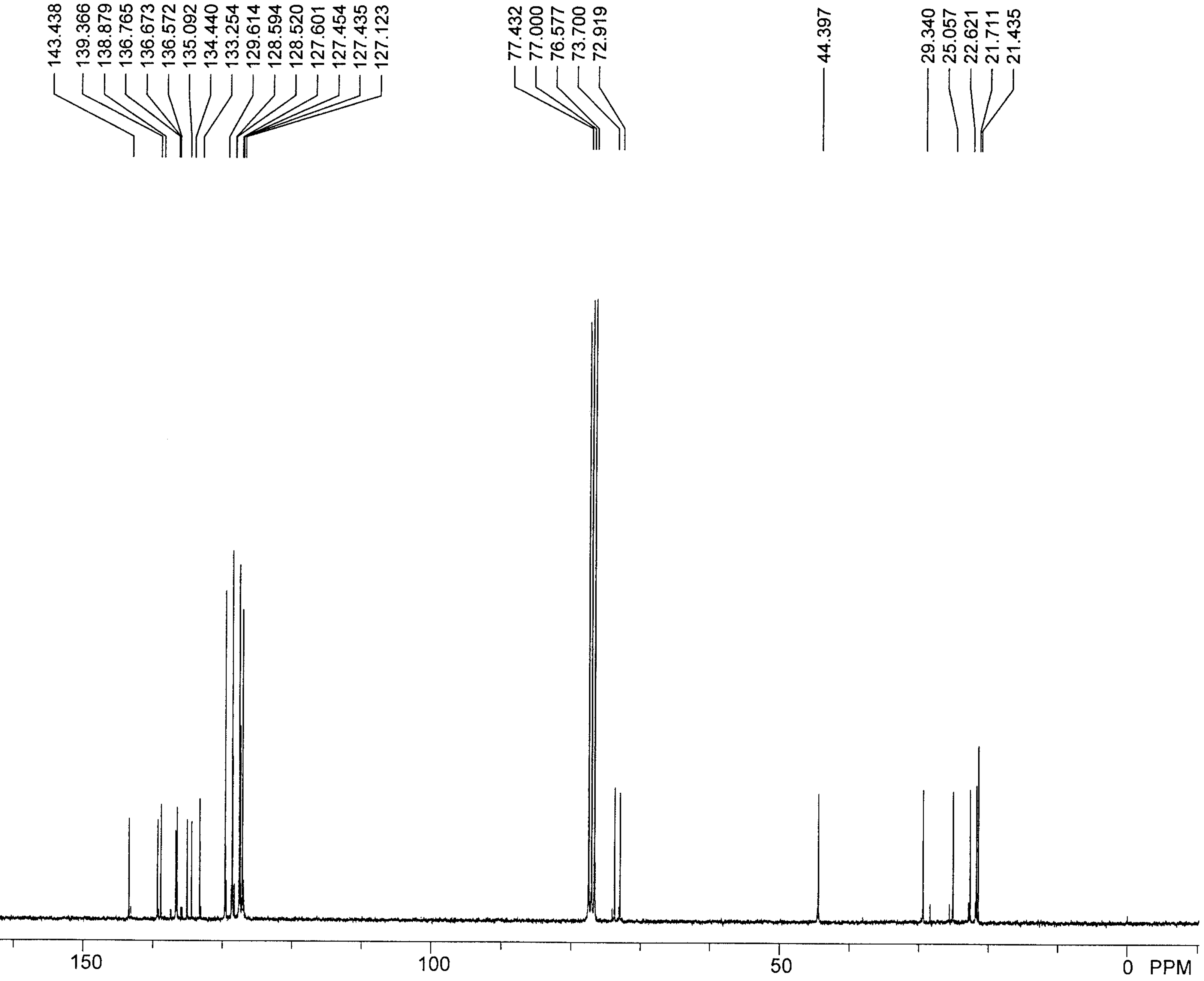

2018-09-18 22:27:02.932
cfsy-zy-138
SOLVENT: CDCl3
Experiment = zg30
Pulse length = 10.000 usec
Relaxation delay = 5.000 sec
NA = 4
F1 = 400.130005 MHz
F2 = 1.000000 MHz

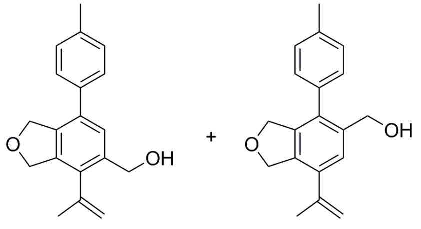

5cb + **6cb**

5cb/6cb ≥ 95/5

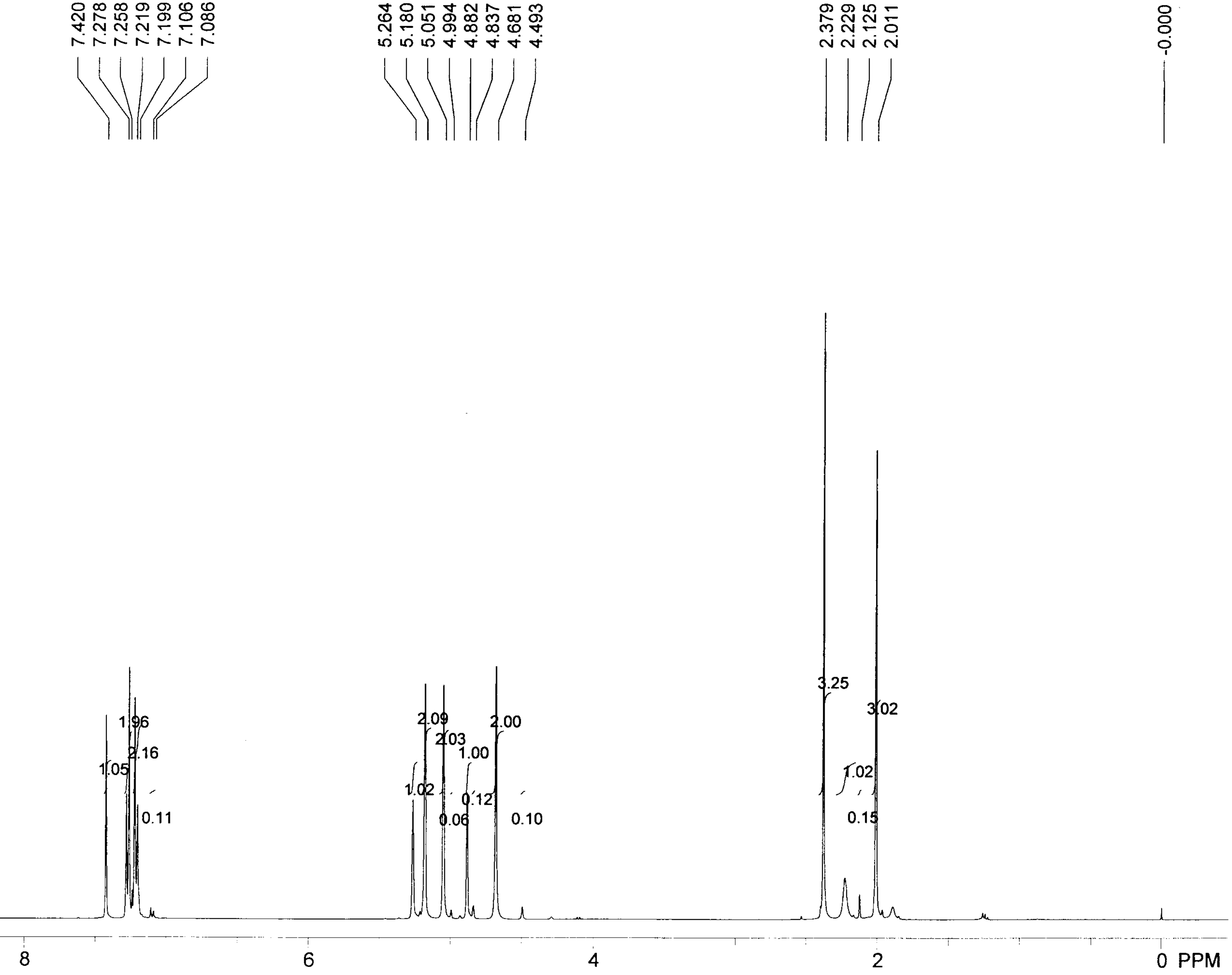

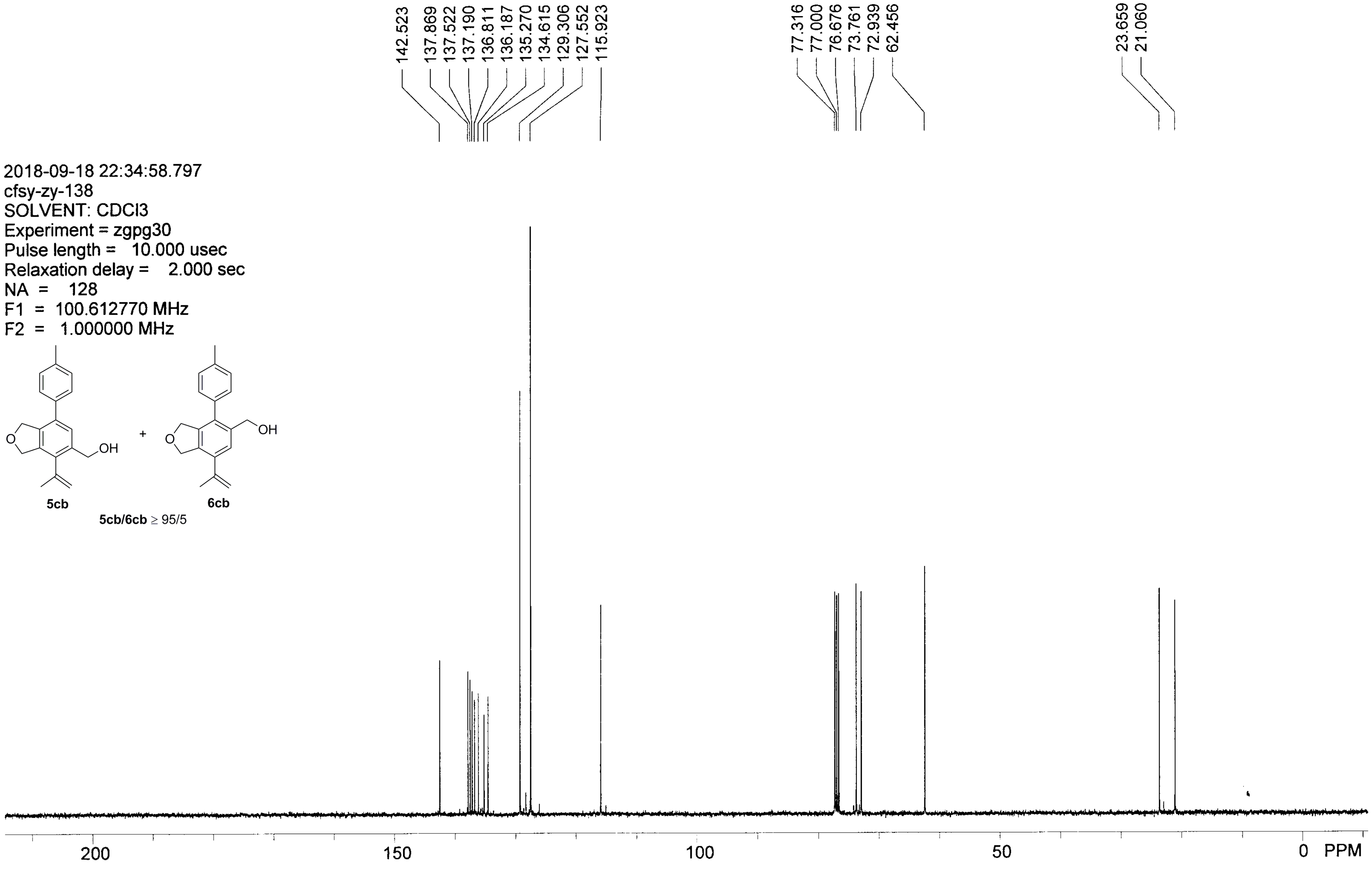
142.523
137.869
137.522
137.190
136.811
136.187
135.270
134.615
129.306
127.552
115.923
77.316
77.000
76.676
73.761
72.939
62.456
23.659
21.060
2018-09-18 22:34:58.797
cfsy-zy-138
SOLVENT: CDCl3
Experiment = zgpg30
Pulse length = 10.000 usec
Relaxation delay = 2.000 sec
NA = 128
F1 = 100.612770 MHz
F2 = 1.000000 MHz
OH
+
OH
5cb
6cb
5cb/6cb ≥ 95/5
200
150
100
50
0 PPM

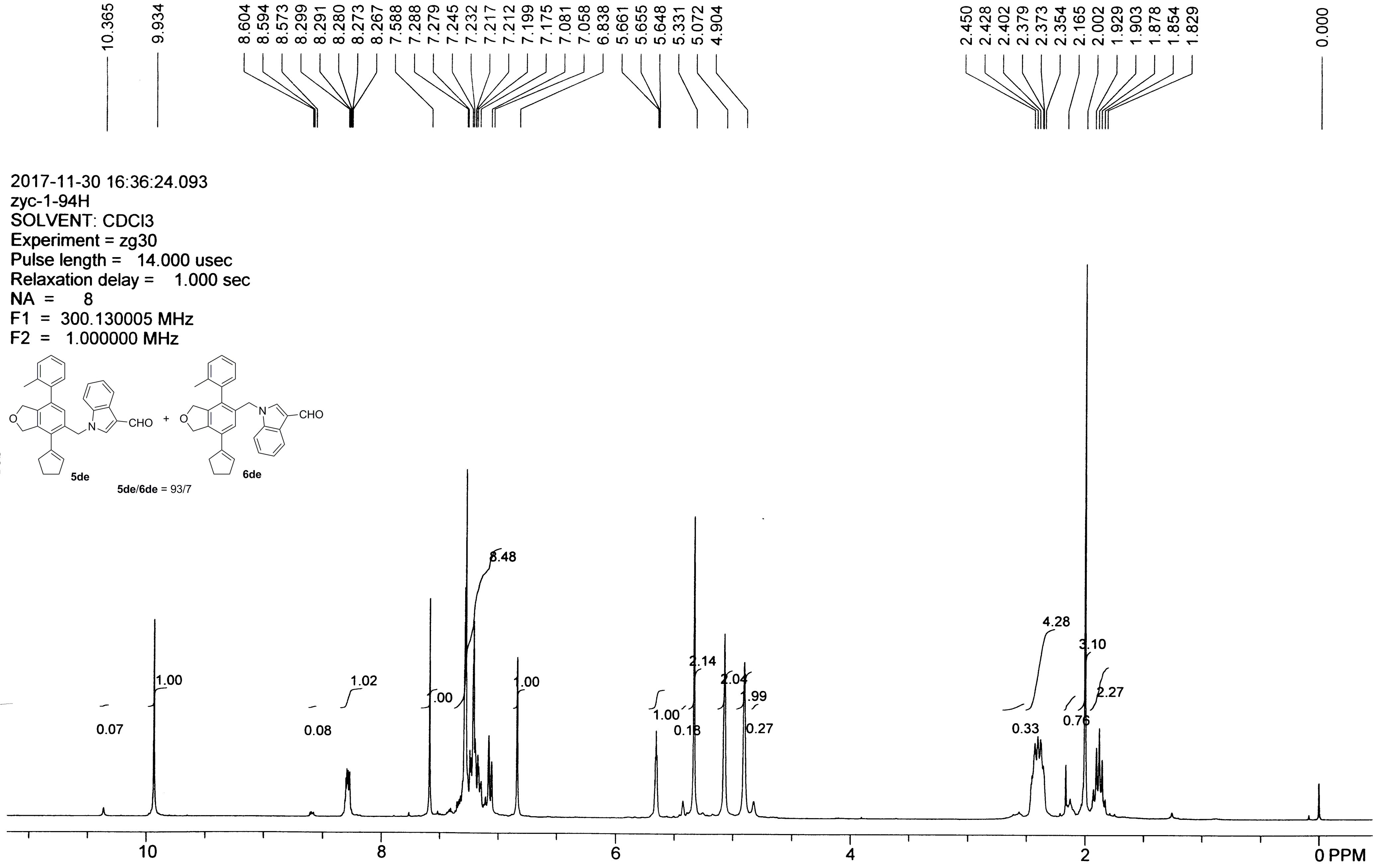

2017-11-30 16:36:24.093
zyc-1-94H
SOLVENT: CDCl3
Experiment = zg30
Pulse length = 14.000 usec
Relaxation delay = 1.000 sec
NA = 8
F1 = 300.130005 MHz
F2 = 1.000000 MHz

5de **6de**

5de/6de = 93/7

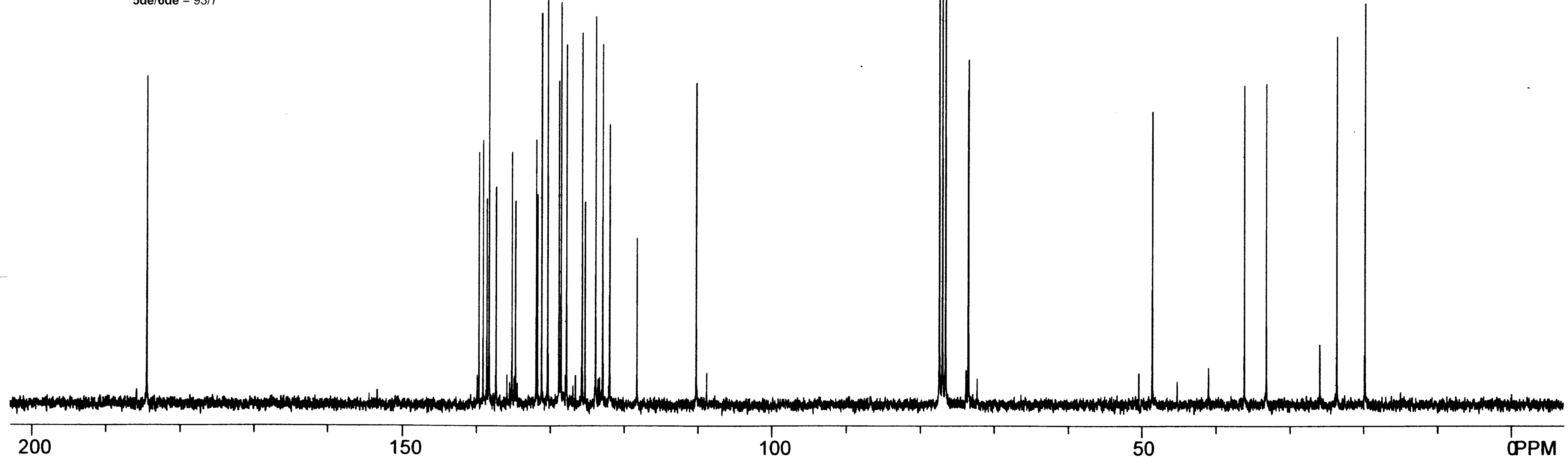

2017-11-30 20:30:04.687
zyc-1-94C
SOLVENT: CDCl3
Experiment = zgpg30
Pulse length = 9.500 usec
Relaxation delay = 2.000 sec
NA = 580
F1 = 75.467751 MHz
F2 = 1.000000 MHz

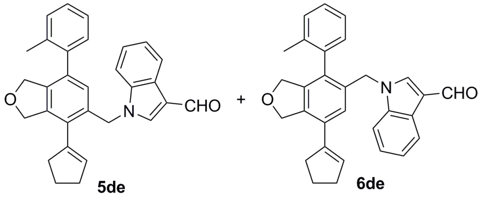

5de 6de

5de/6de = 93/7

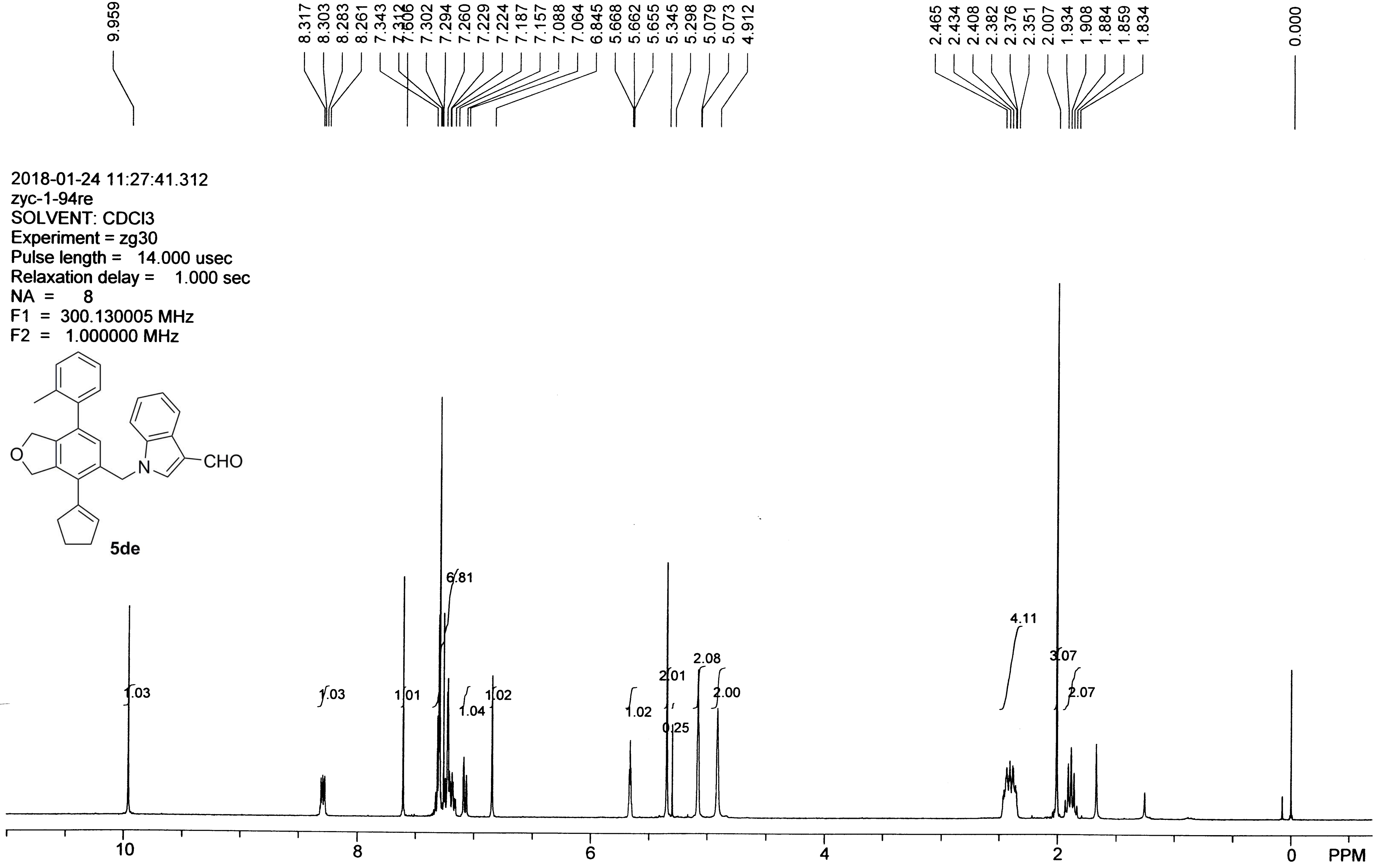
2018-01-24 11:27:41.312
zyc-1-94re
SOLVENT: CDCl3
Experiment = zg30
Pulse length = 14.000 usec
Relaxation delay = 1.000 sec
NA = 8
F1 = 300.130005 MHz
F2 = 1.000000 MHz
9.959
8.317
8.303
8.283
8.261
7.343
7.312
7.606
7.302
7.294
7.260
7.229
7.224
7.187
7.157
7.088
7.064
6.845
5.668
5.662
5.655
5.345
5.298
5.079
5.073
4.912
2.465
2.434
2.408
2.382
2.376
2.351
2.007
1.934
1.908
1.884
1.859
1.834
0.000
1.03
1.03
1.01
6.81
1.04
1.02
1.02
2.01
0.25
2.08
2.00
4.11
3.07
2.07
10
8
6
4
2
0
PPM
O
N
CHO

5de

2017-12-07 21:21:13.203
zyc-1-101H
SOLVENT: CDCl3
Experiment = zg30
Pulse length = 14.000 usec
Relaxation delay = 1.000 sec
NA = 8
F1 = 300.130005 MHz
F2 = 1.000000 MHz

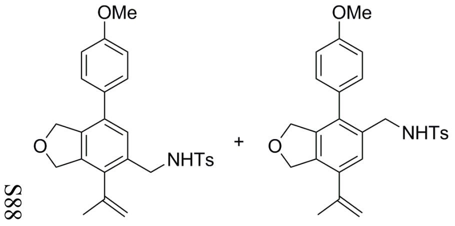

5ea **6ea**

5ea/6ea = 97/3

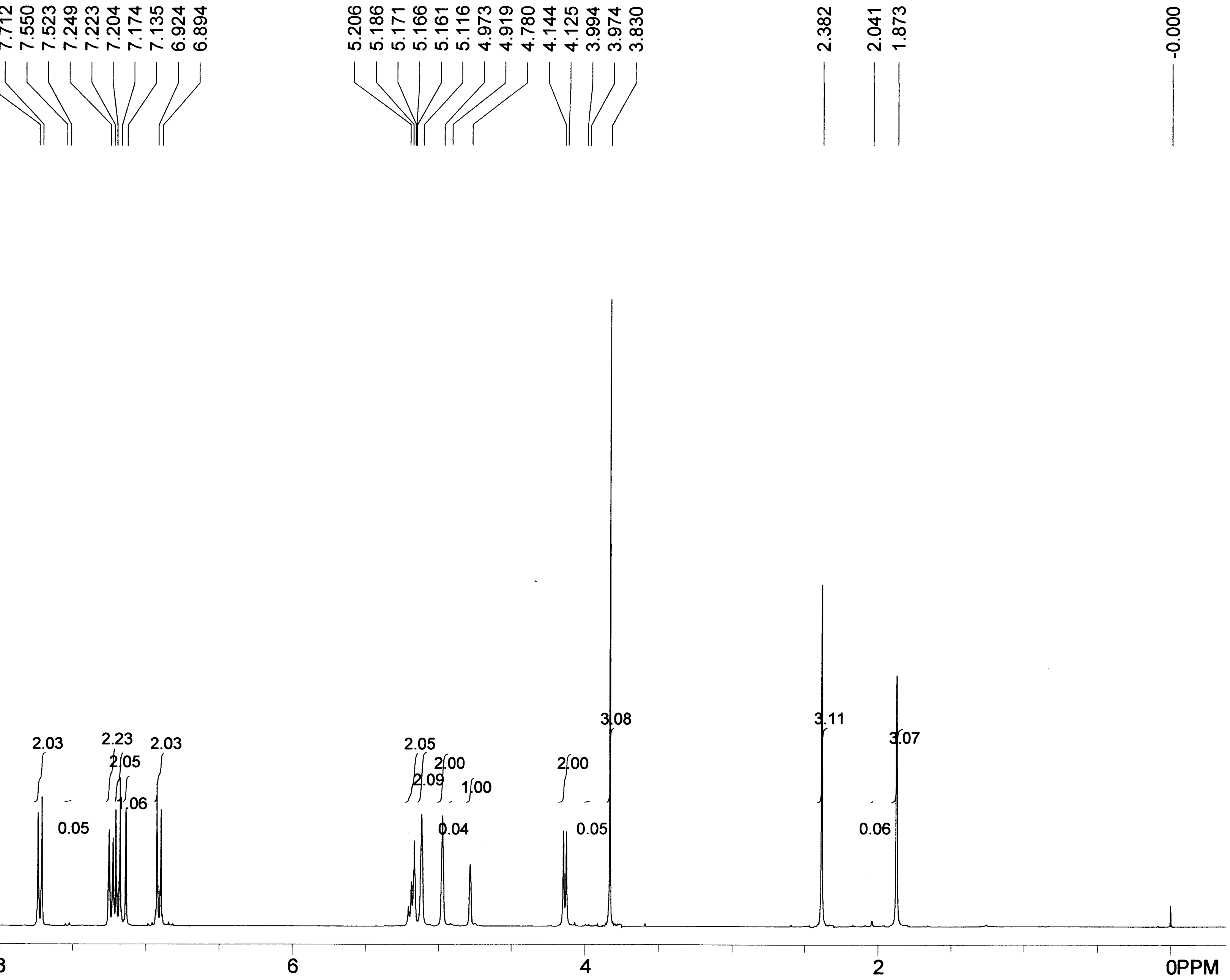

2017-12-11 17:03:28.125
zyc-1-101C
SOLVENT: CDCl3
Experiment = zgpg30
Pulse length = 9.500 usec
Relaxation delay = 2.000 sec
NA = 263
F1 = 75.467751 MHz
F2 = 1.000000 MHz

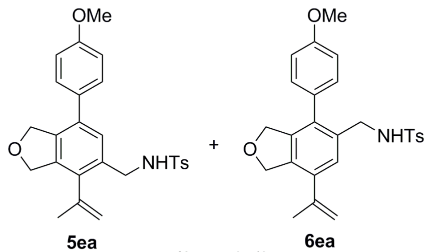

5ea 6ea
5ea/6ea = 97/3

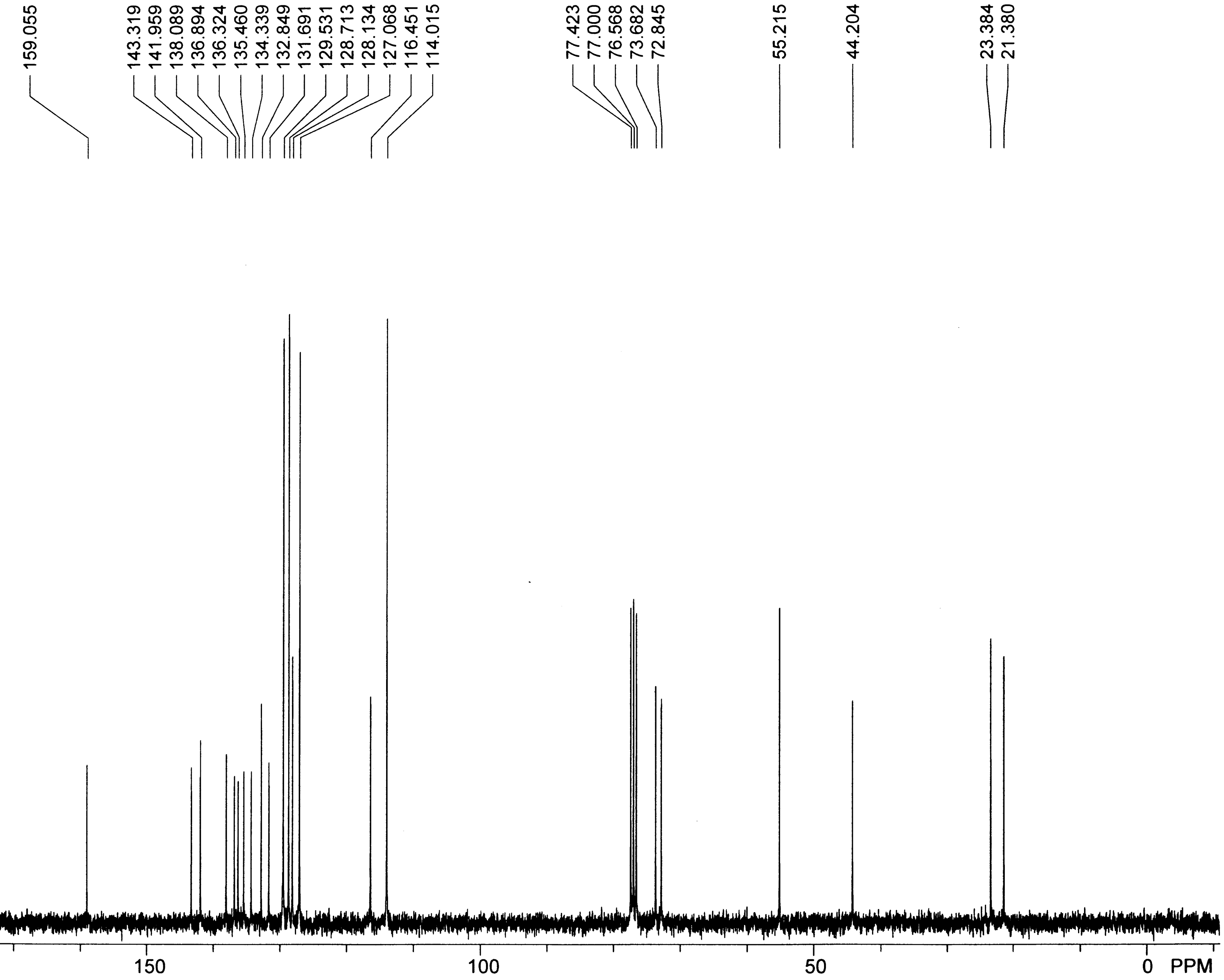

2017-12-19 21:17:22.250
zyc-1-101re
SOLVENT: CDCl3
Experiment = zg30
Pulse length = 14.000 usec
Relaxation delay = 1.000 sec
NA = 8
F1 = 300.130005 MHz
F2 = 1.000000 MHz

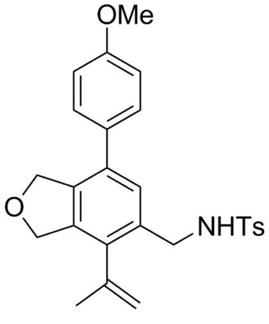

5ea

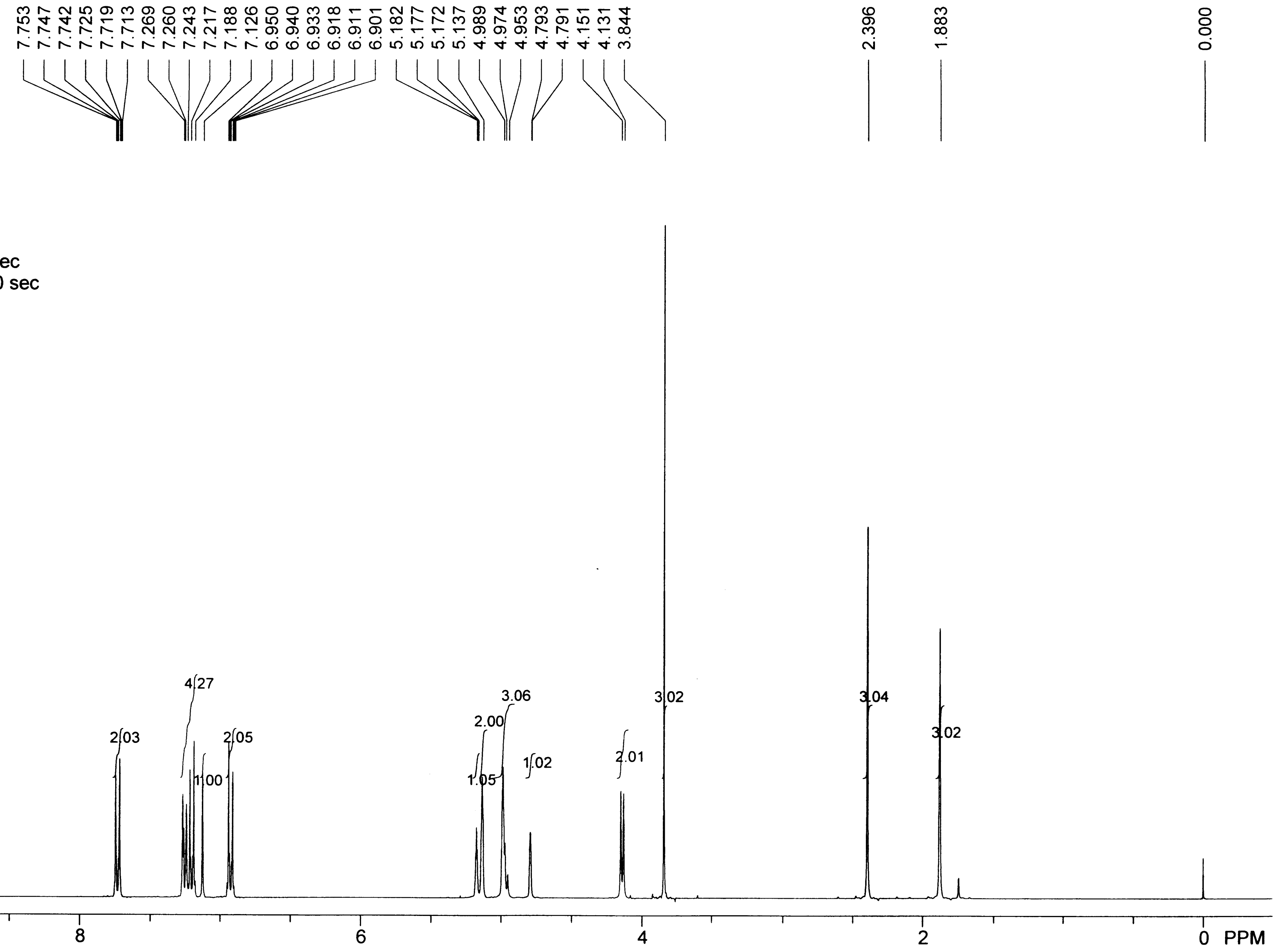

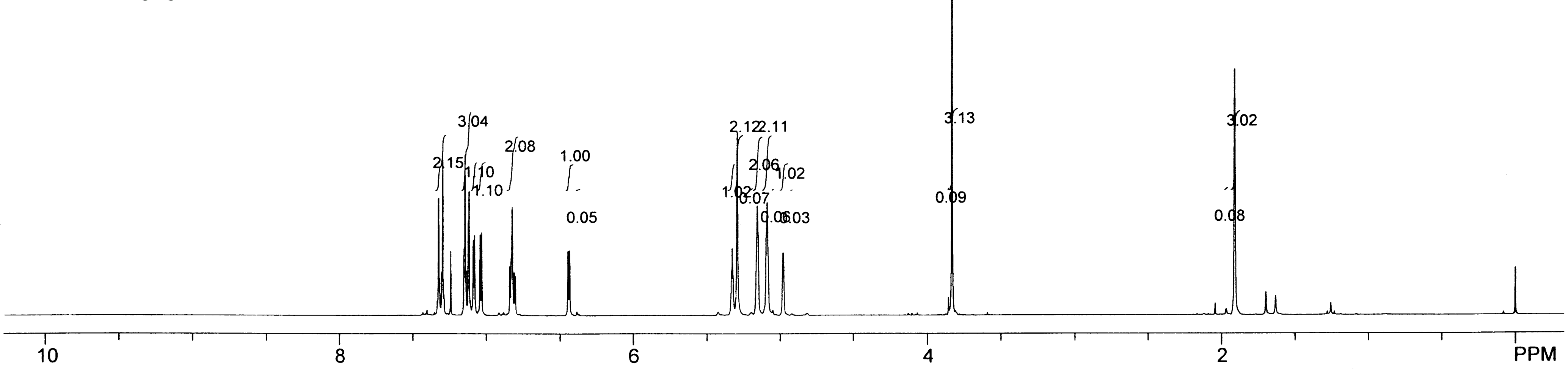

2017-11-19 21:25:23.234
zyc-1-87H
SOLVENT: CDCl3
Experiment = zg30
Pulse length = 14.000 usec
Relaxation delay = 1.000 sec
NA = 8
F1 = 300.130005 MHz
F2 = 1.000000 MHz

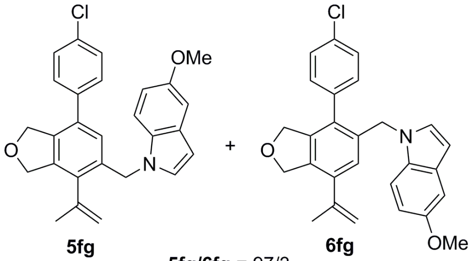

5fg **5fg/6fg** = 97/3 **6fg**

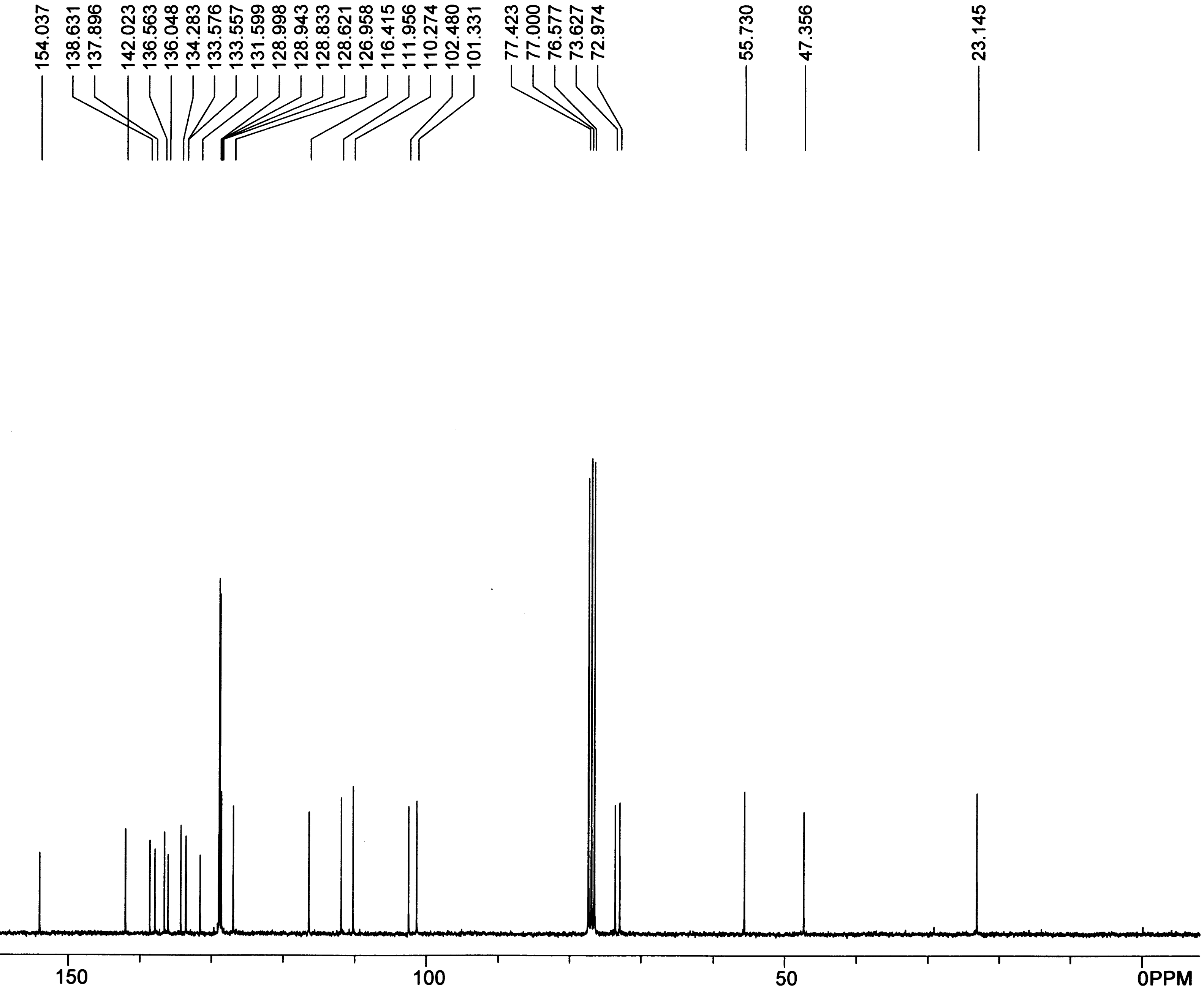

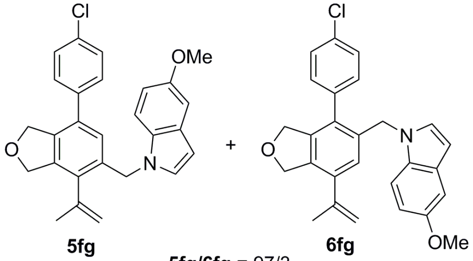

5fg + **6fg**

5fg/6fg = 97/3

2017-12-17 09:08:09.625
zyc-1-87reH
SOLVENT: CDCl3
Experiment = zg30
Pulse length = 14.000 usec
Relaxation delay = 1.000 sec
NA = 8
F1 = 300.130005 MHz
F2 = 1.000000 MHz

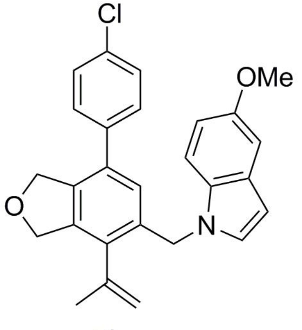

5fg

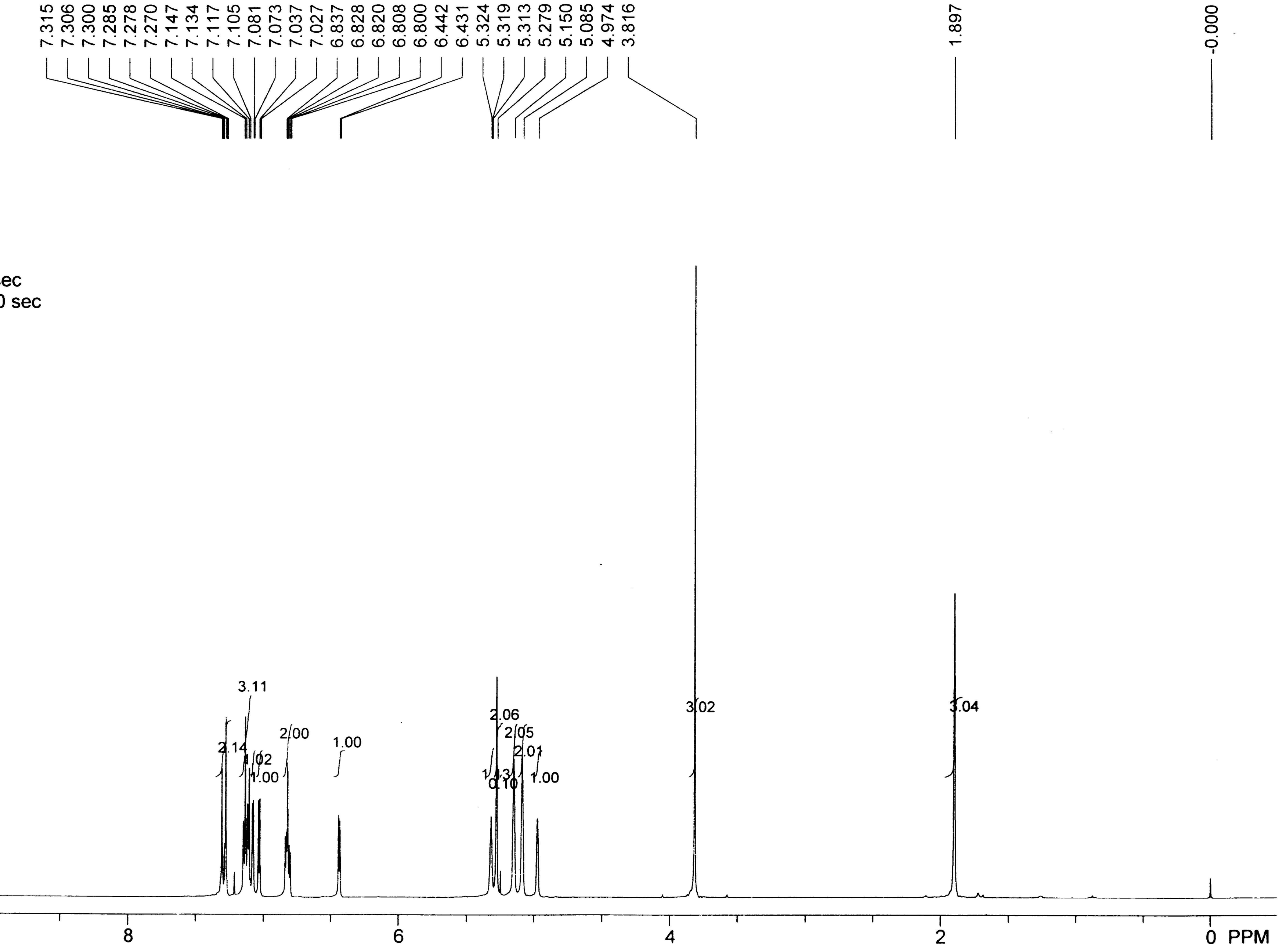

2018-01-30 21:59:35.921
zyc-1-131re
SOLVENT: CDCl3
Experiment = zg30
Pulse length = 14.000 usec
Relaxation delay = 1.000 sec
NA = 8
F1 = 300.130005 MHz
F2 = 1.000000 MHz

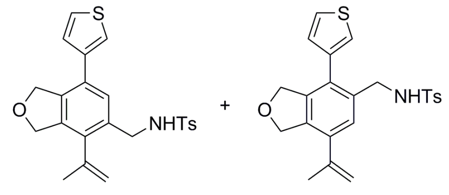

5ga + **6ga**

5ga/6ga = 96/4

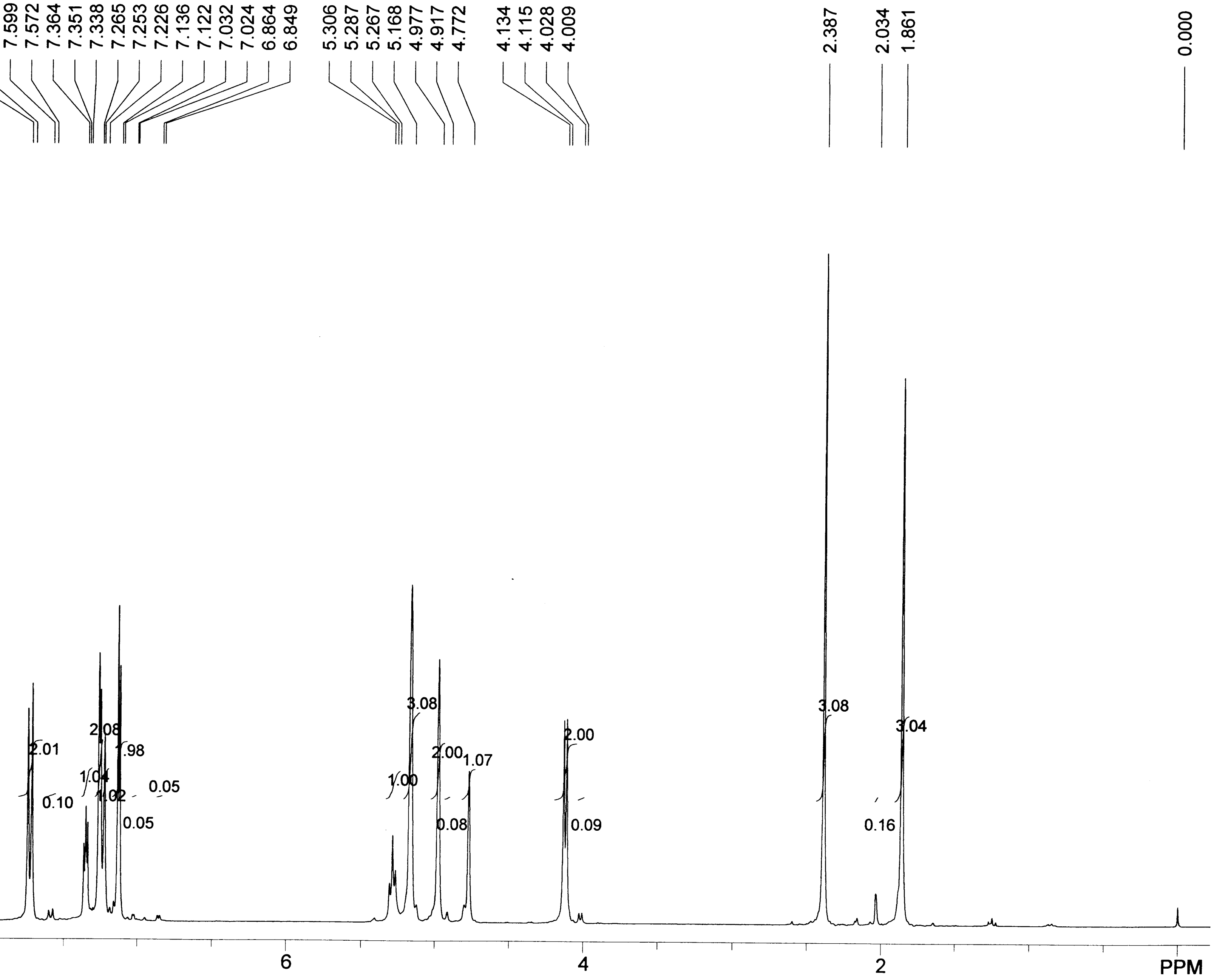

2018-01-31 09:03:21.640
zyc-1-131re
SOLVENT: CDCl3
Experiment = zgpg30
Pulse length =   9.500 usec
Relaxation delay =   2.000 sec
NA =   417
F1 = 75.467751 MHz
F2 =   1.000000 MHz

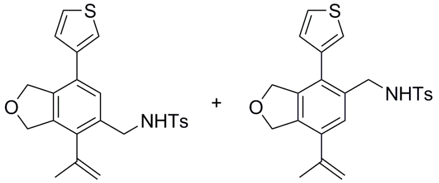

5ga **6ga**

5ga/6ga = 96/4

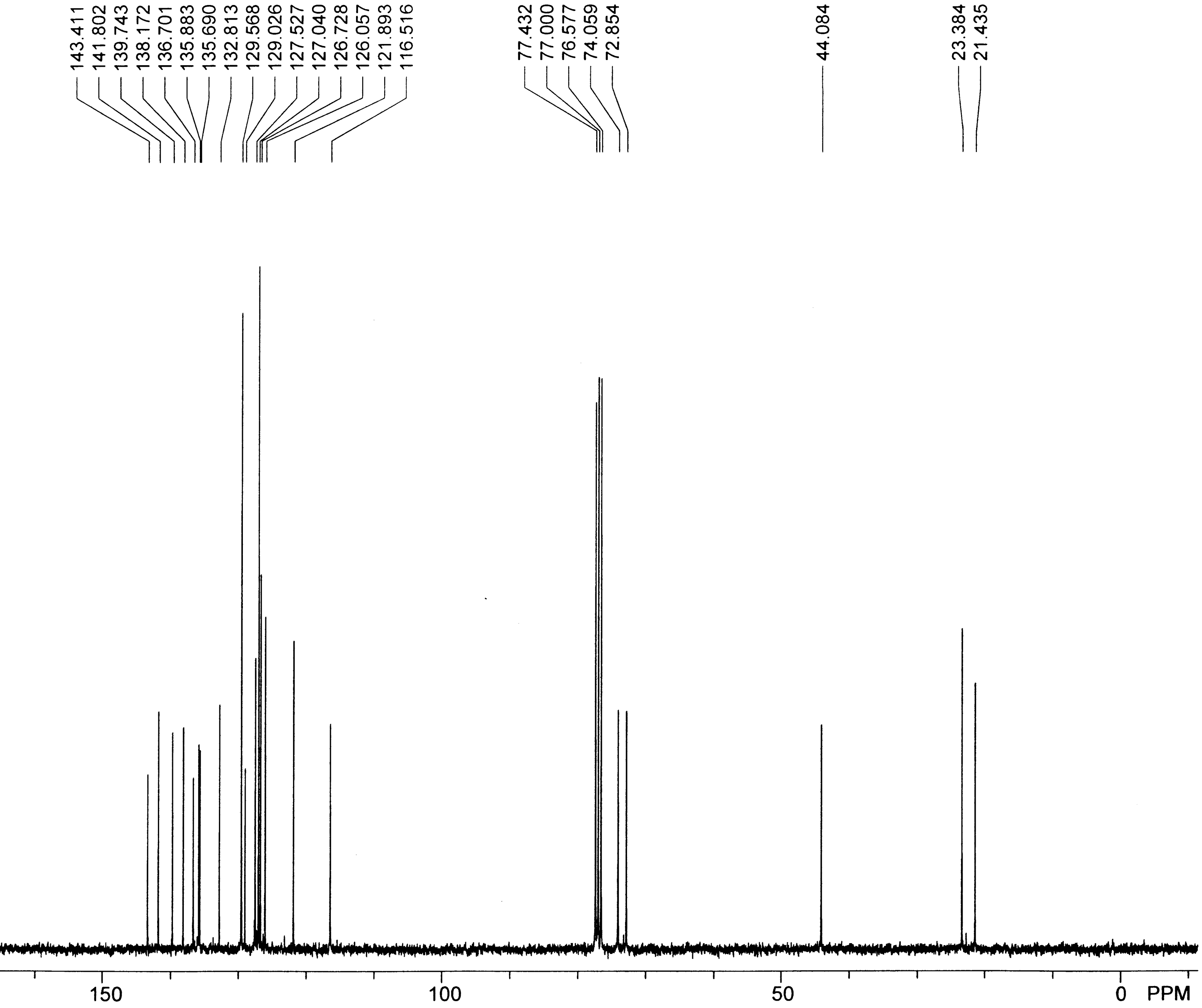

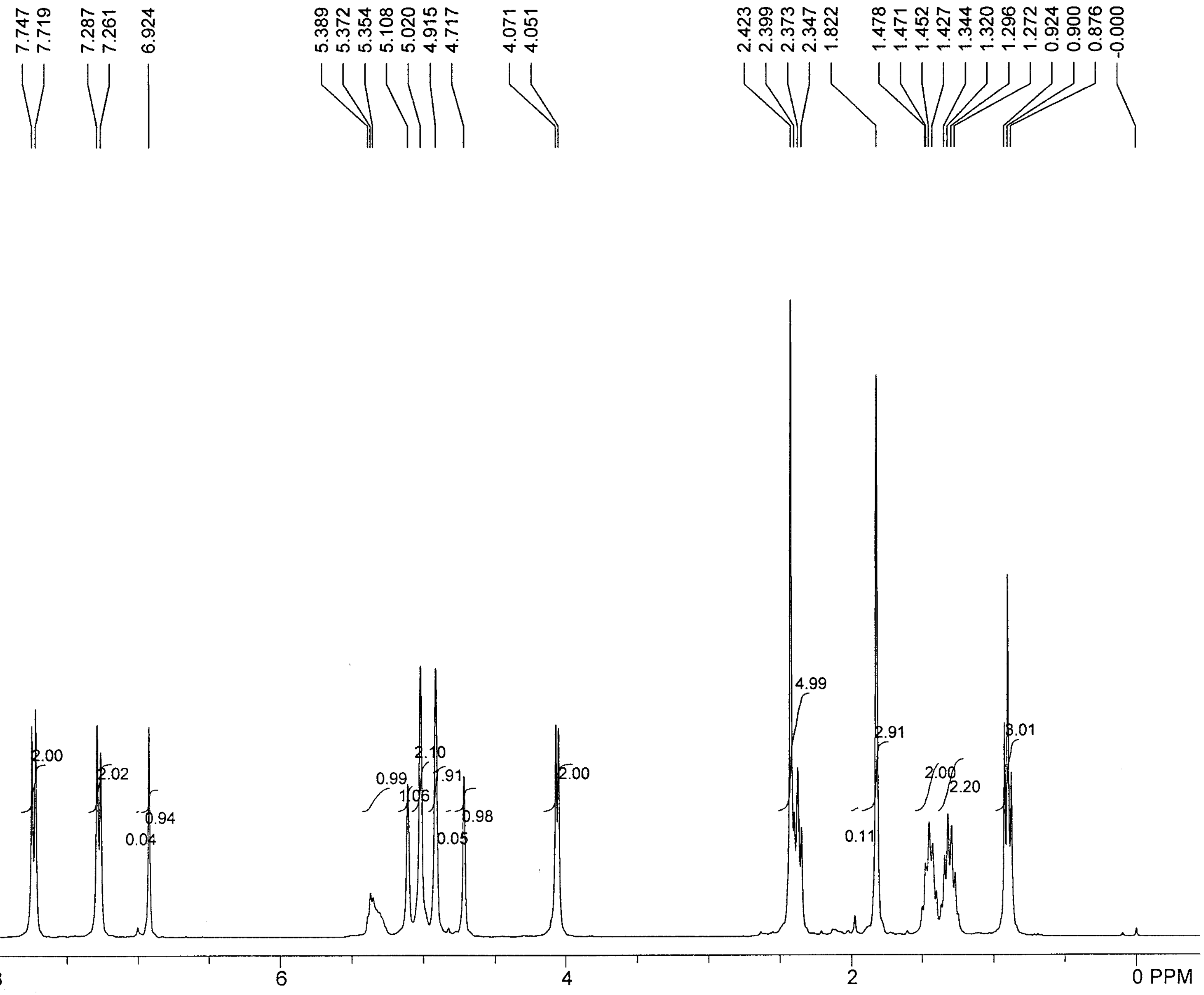

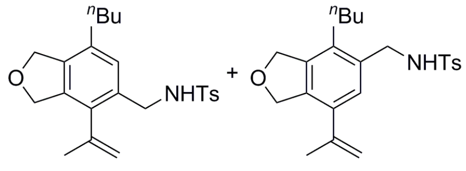

5ka **6ka**

5ka/6ka = 96/4

2018-11-23 10:21:56.359
zyc-3-32
SOLVENT: CDCl3
Experiment = zgpg30
Pulse length = 9.500 usec
Relaxation delay = 2.000 sec
NA = 360
F1 = 75.467751 MHz
F2 = 1.000000 MHz

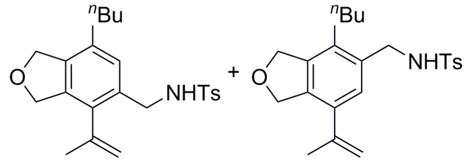

5ka **6ka**

5ka/6ka = 96/4

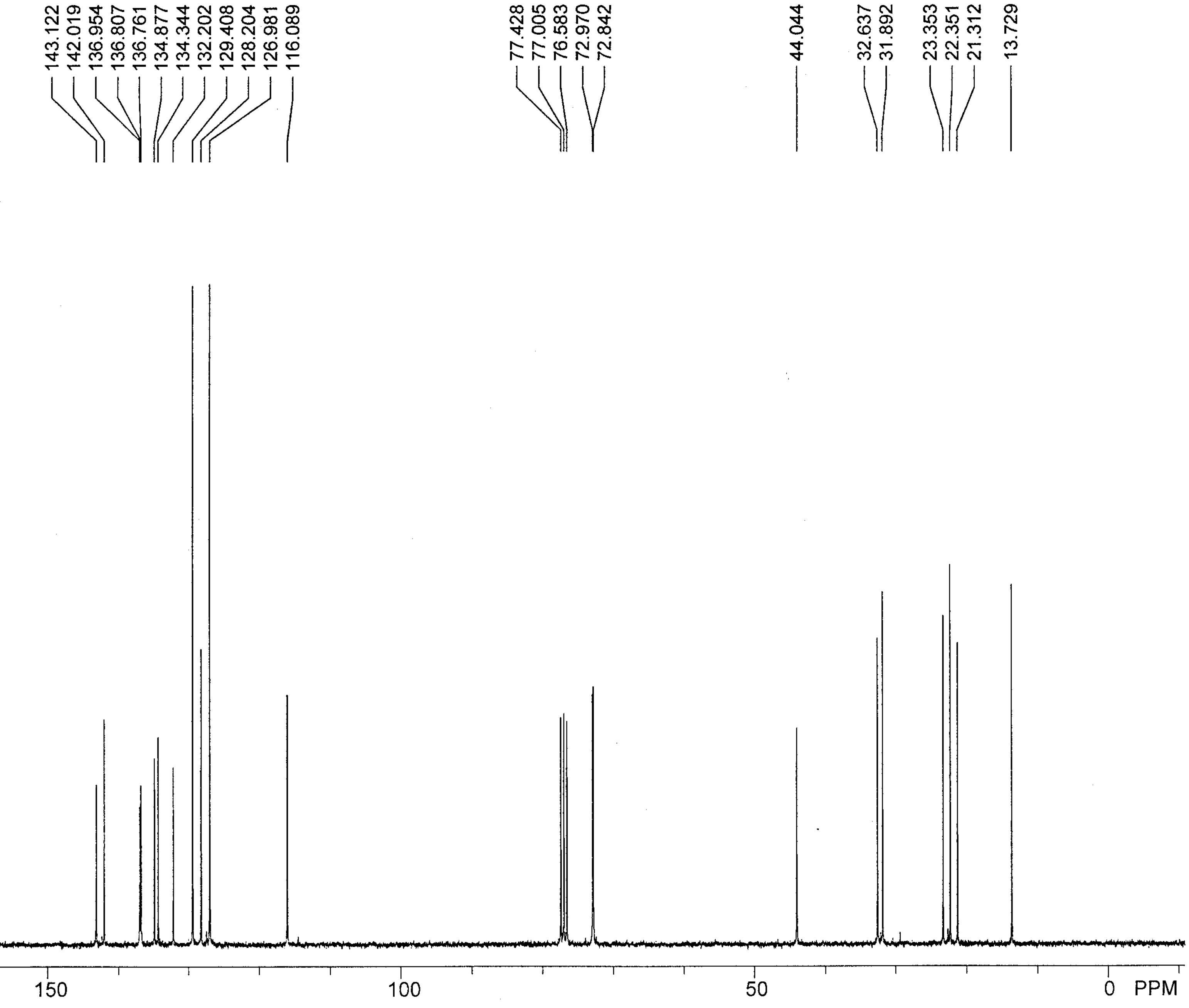

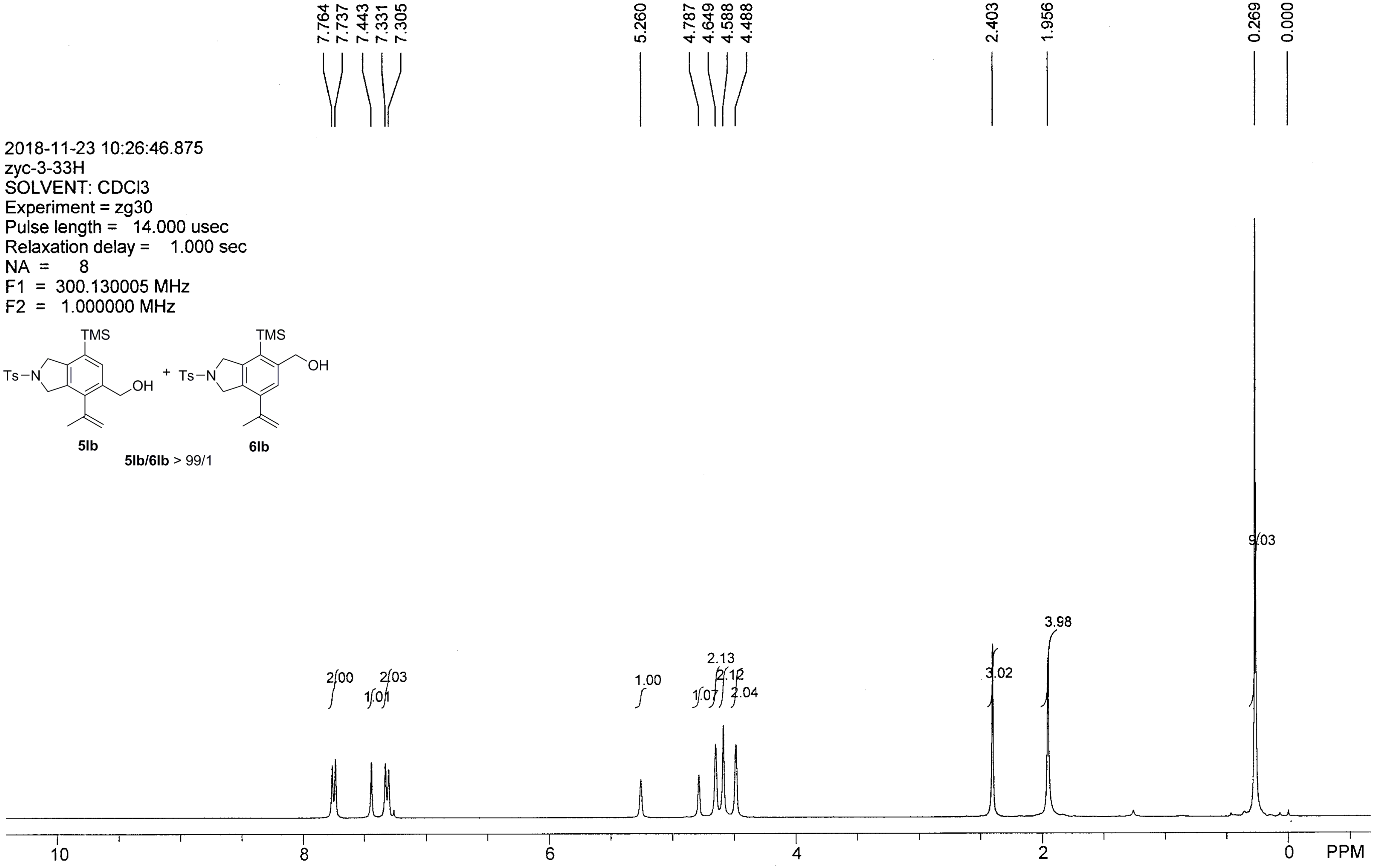
2018-11-23 10:26:46.875
zyc-3-33H
SOLVENT: CDCl3
Experiment = zg30
Pulse length = 14.000 usec
Relaxation delay = 1.000 sec
NA = 8
F1 = 300.130005 MHz
F2 = 1.000000 MHz
TMS
Ts−N
OH
+
5lb
6lb
5lb/6lb > 99/1
7.764
7.737
7.443
7.331
7.305
5.260
4.787
4.649
4.588
4.488
2.403
1.956
0.269
0.000
2.00
1.01
2.03
1.00
1.07
2.13
2.12
2.04
3.02
3.98
9.03
10
8
6
4
2
0
PPM

2018-11-23 10:40:48.890
zyc-3-33
SOLVENT: CDCl3
Experiment = zgpg30
Pulse length = 9.500 usec
Relaxation delay = 2.000 sec
NA = 221
F1 = 75.467751 MHz
F2 = 1.000000 MHz

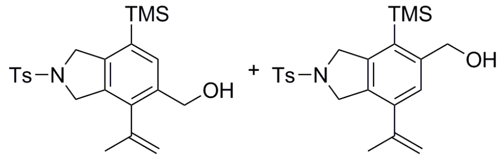

5lb **6lb**

5lb/6lb > 99/1

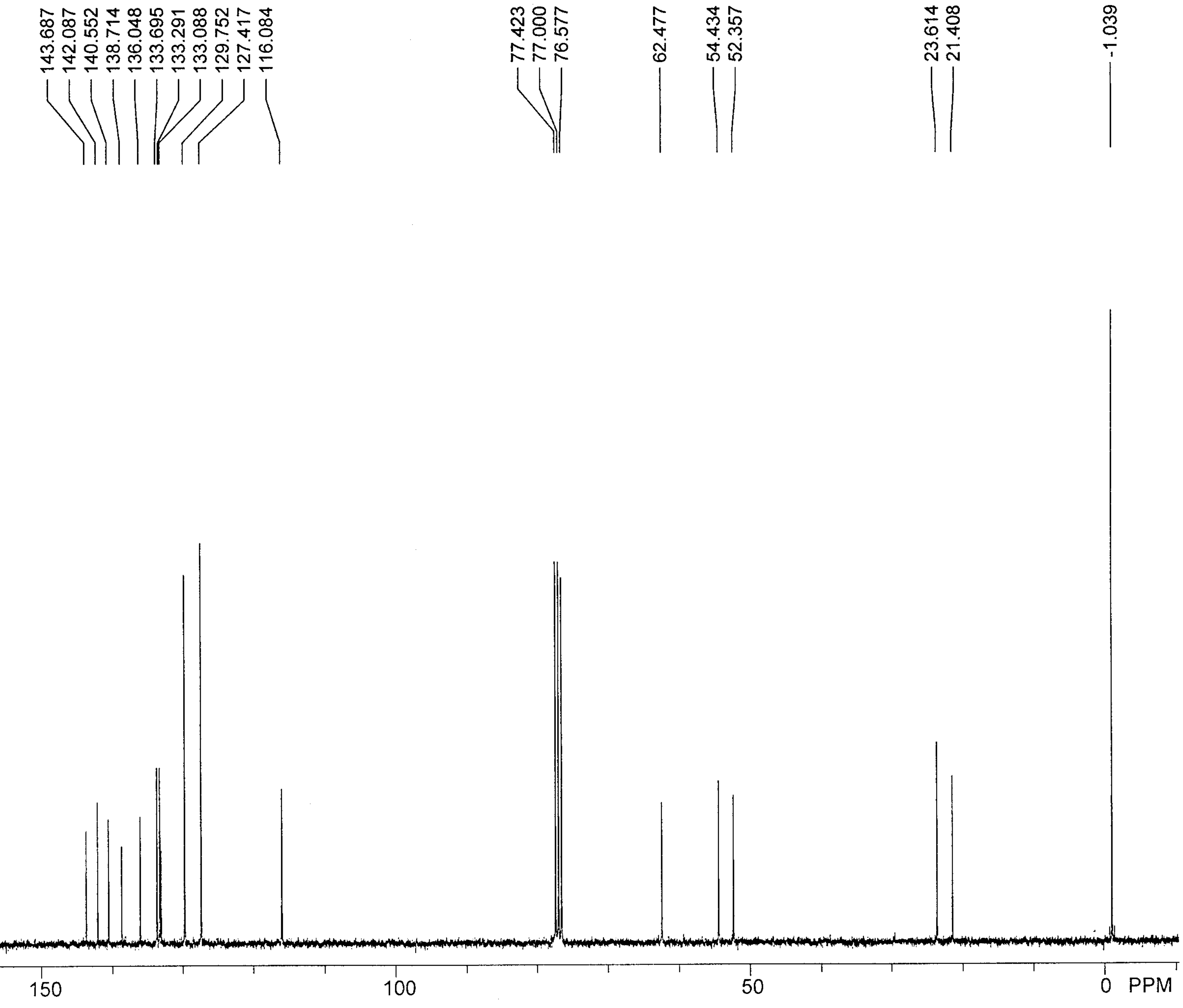

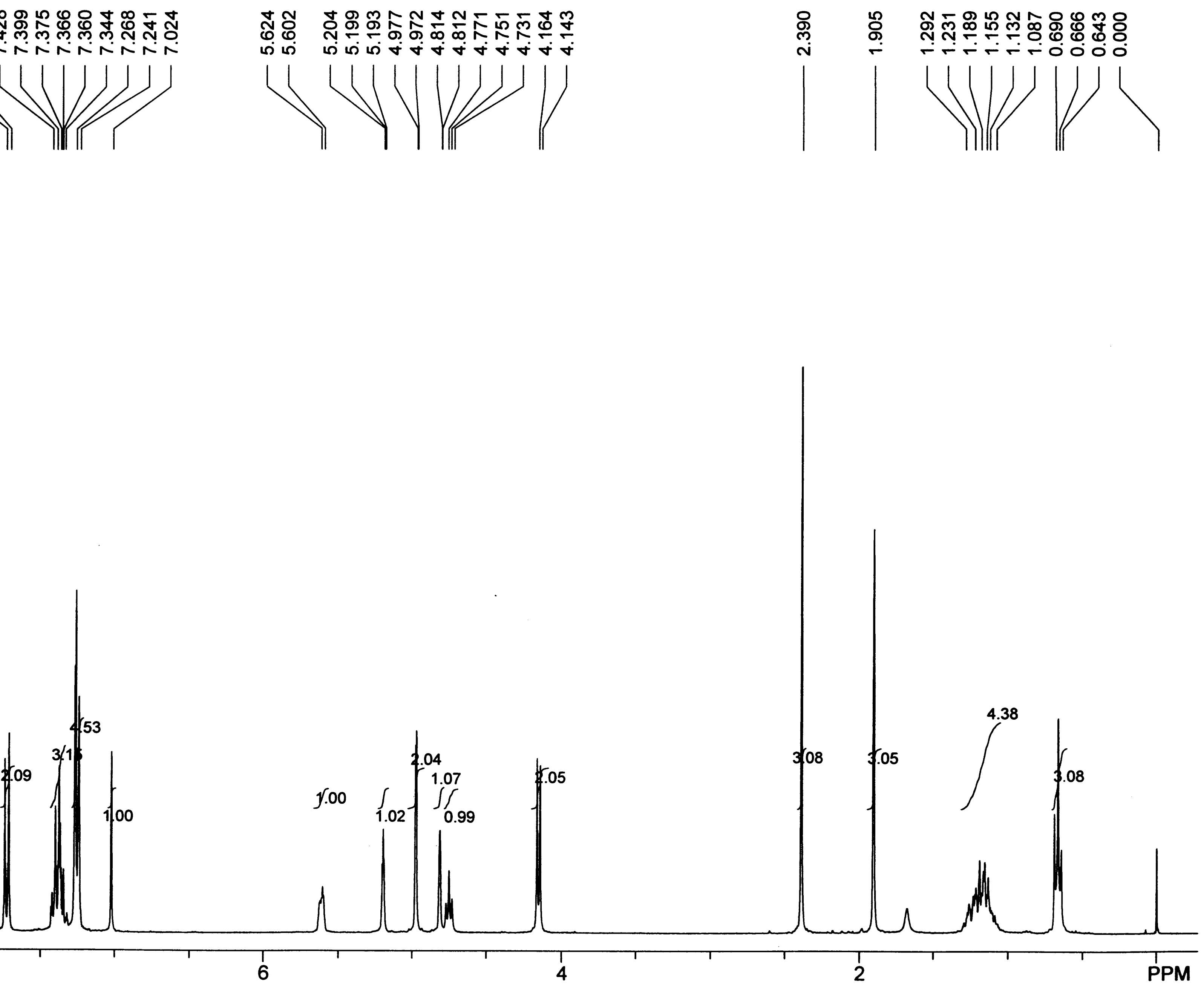

2017-10-22 13:44:42.125
zyc-1-71H
SOLVENT: CDCl3
Experiment = zg30
Pulse length = 14.000 usec
Relaxation delay = 1.000 sec
NA = 8
F1 = 300.130005 MHz
F2 = 1.000000 MHz

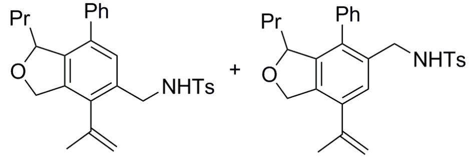

5ha **6ha**

5ha/6ha > 99/1

2017-10-22 17:19:02.953
zyc-1-71C
SOLVENT: CDCl3
Experiment = zgpg30
Pulse length = 9.500 usec
Relaxation delay = 2.000 sec
NA = 454
F1 = 75.467751 MHz
F2 = 1.000000 MHz

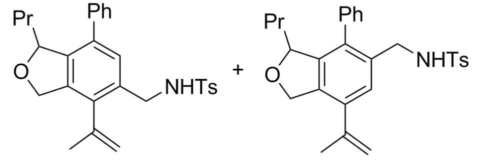

5ha **6ha**

5ha/6ha > 99/1

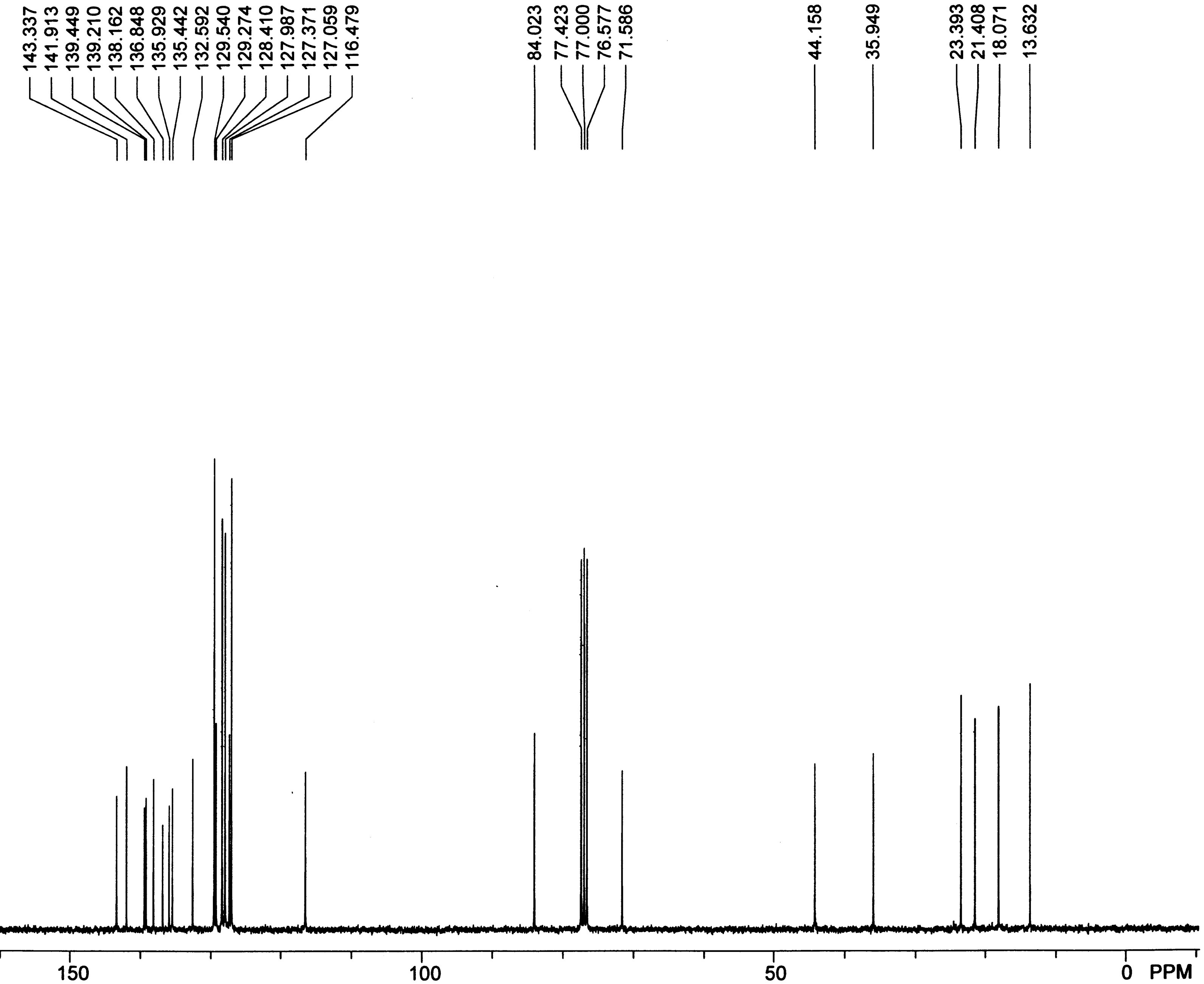

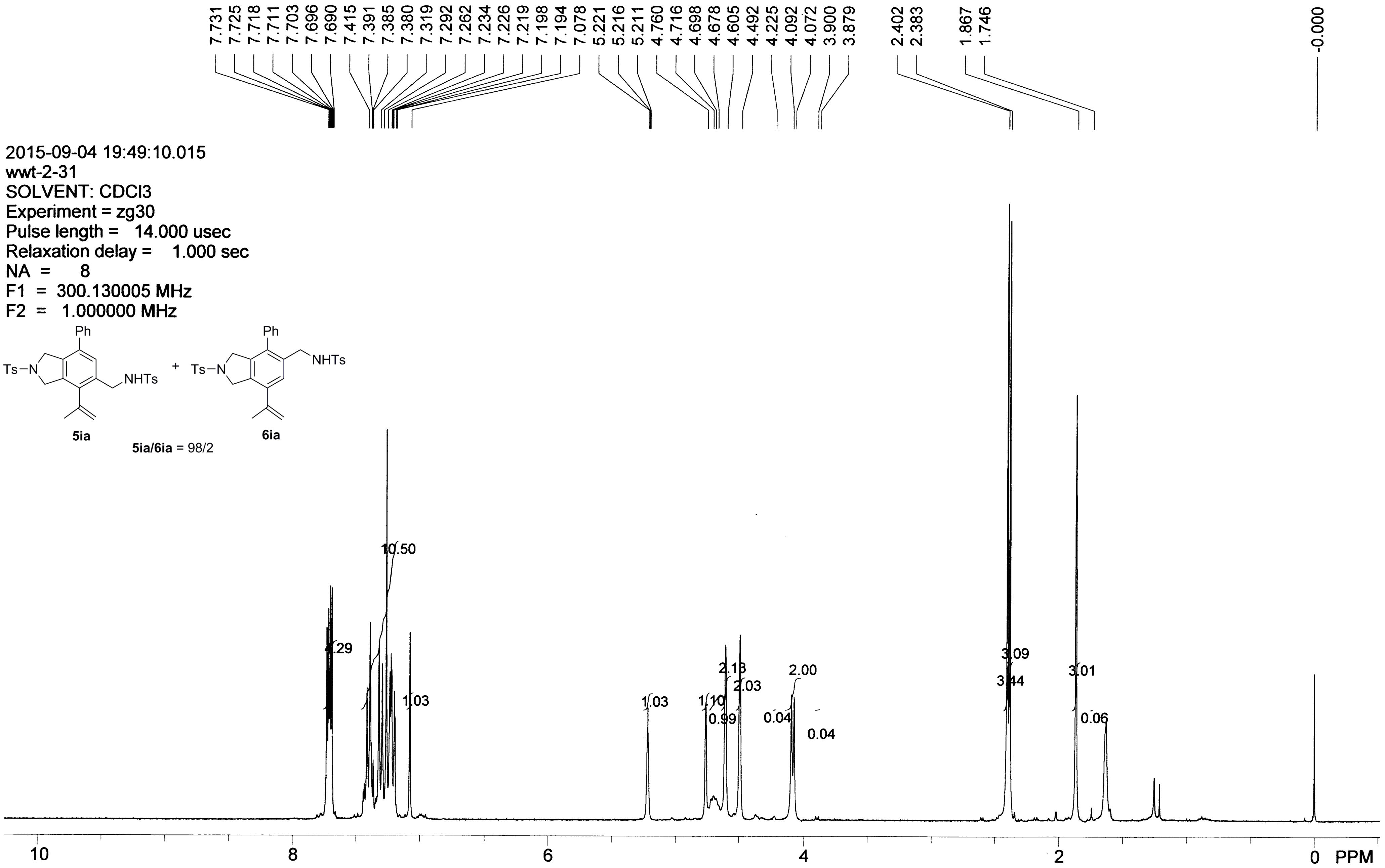
2015-09-04 19:49:10.015
wwt-2-31
SOLVENT: CDCl3
Experiment = zg30
Pulse length = 14.000 usec
Relaxation delay = 1.000 sec
NA = 8
F1 = 300.130005 MHz
F2 = 1.000000 MHz
Ph
Ts−N
NHTs
+
5ia
6ia
5ia/6ia = 98/2
7.731 7.725 7.718 7.711 7.703 7.696 7.690 7.415 7.391 7.385 7.380 7.319 7.292 7.262 7.234 7.226 7.219 7.198 7.194 7.078 5.221 5.216 5.211 4.760 4.716 4.698 4.678 4.605 4.492 4.225 4.092 4.072 3.900 3.879 2.402 2.383 1.867 1.746 -0.000
4.29
10.50
1.03
1.03
1.10
0.99
2.18
2.03
0.04
2.00
0.04
3.09
3.44
3.01
0.06
10
8
6
4
2
0 PPM

2015-09-03 15:00:16.937
wwt-2-31C
SOLVENT: CDCl3
Experiment = zgpg30
Pulse length = 9.500 usec
Relaxation delay = 2.000 sec
NA = 196
F1 = 75.467751 MHz
F2 = 1.000000 MHz

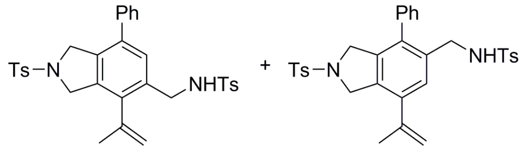

5ia **6ia**

5ia/6ia = 98/2

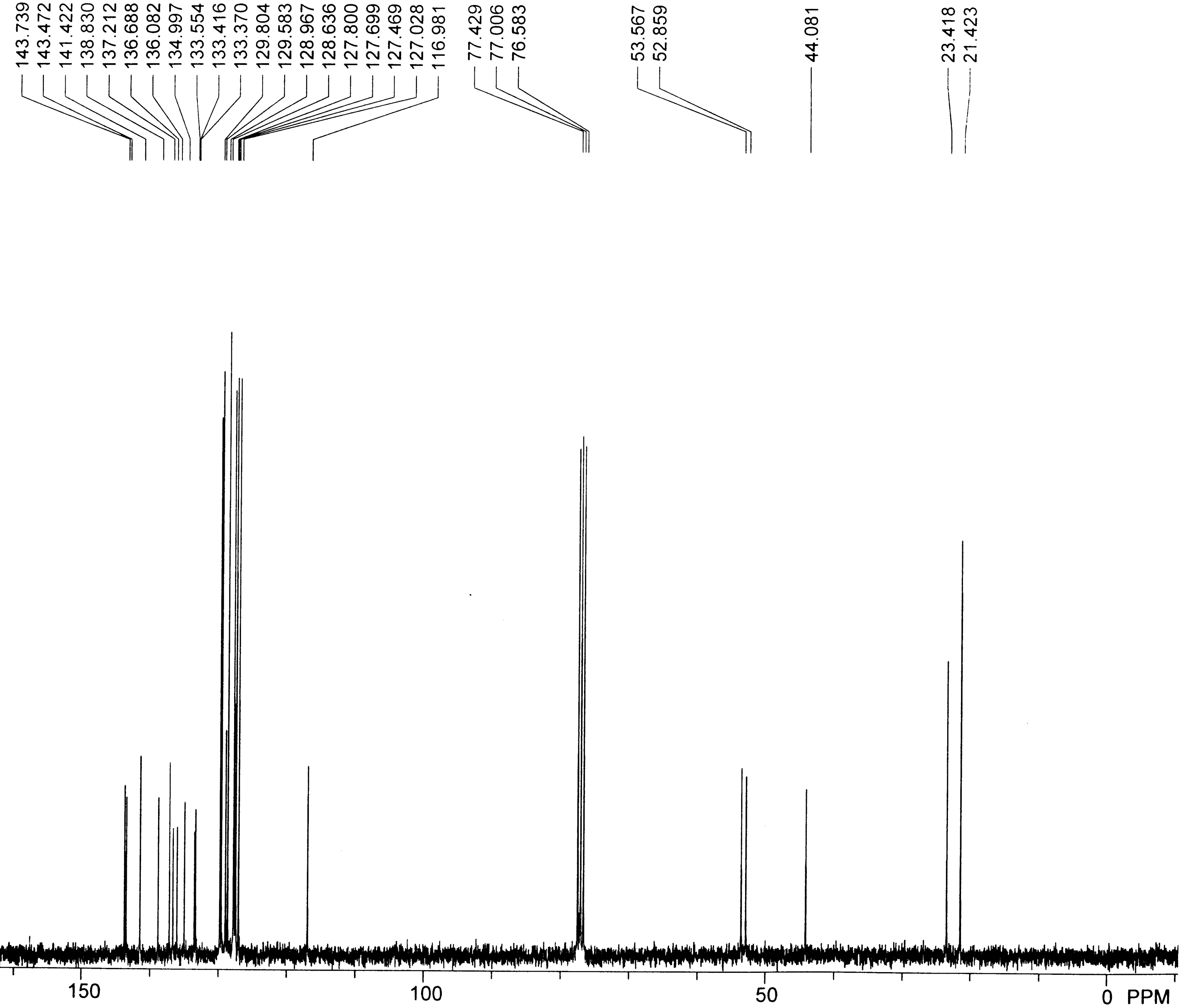

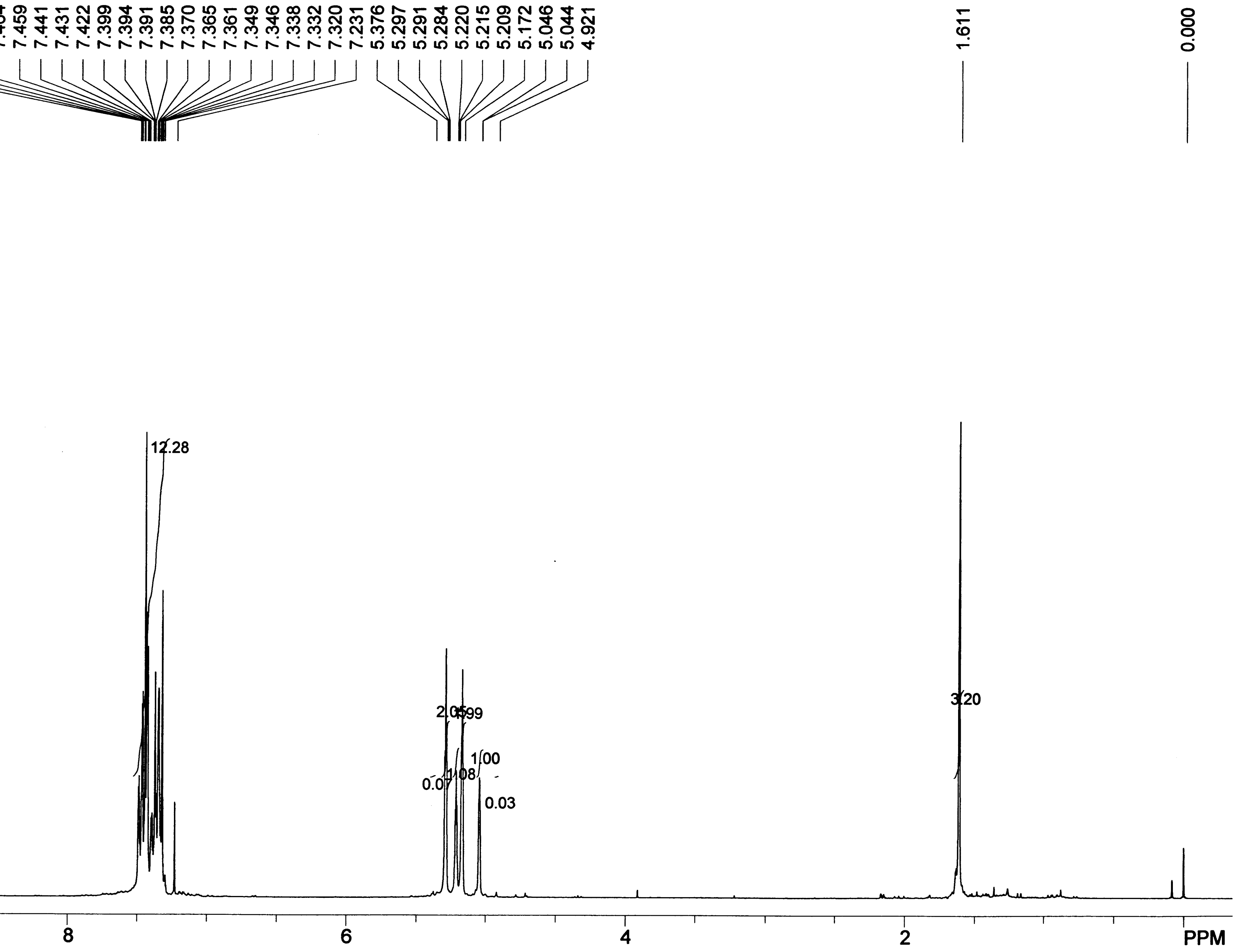

2017-12-24 09:39:48.484
zyc-1-113
SOLVENT: CDCl3
Experiment = zg30
Pulse length = 14.000 usec
Relaxation delay = 1.000 sec
NA = 8
F1 = 300.130005 MHz
F2 = 1.000000 MHz

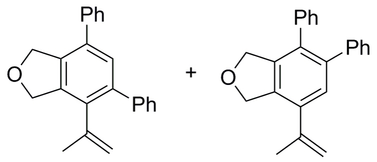

5ah **6ah**

5ah/6ah = 96/4

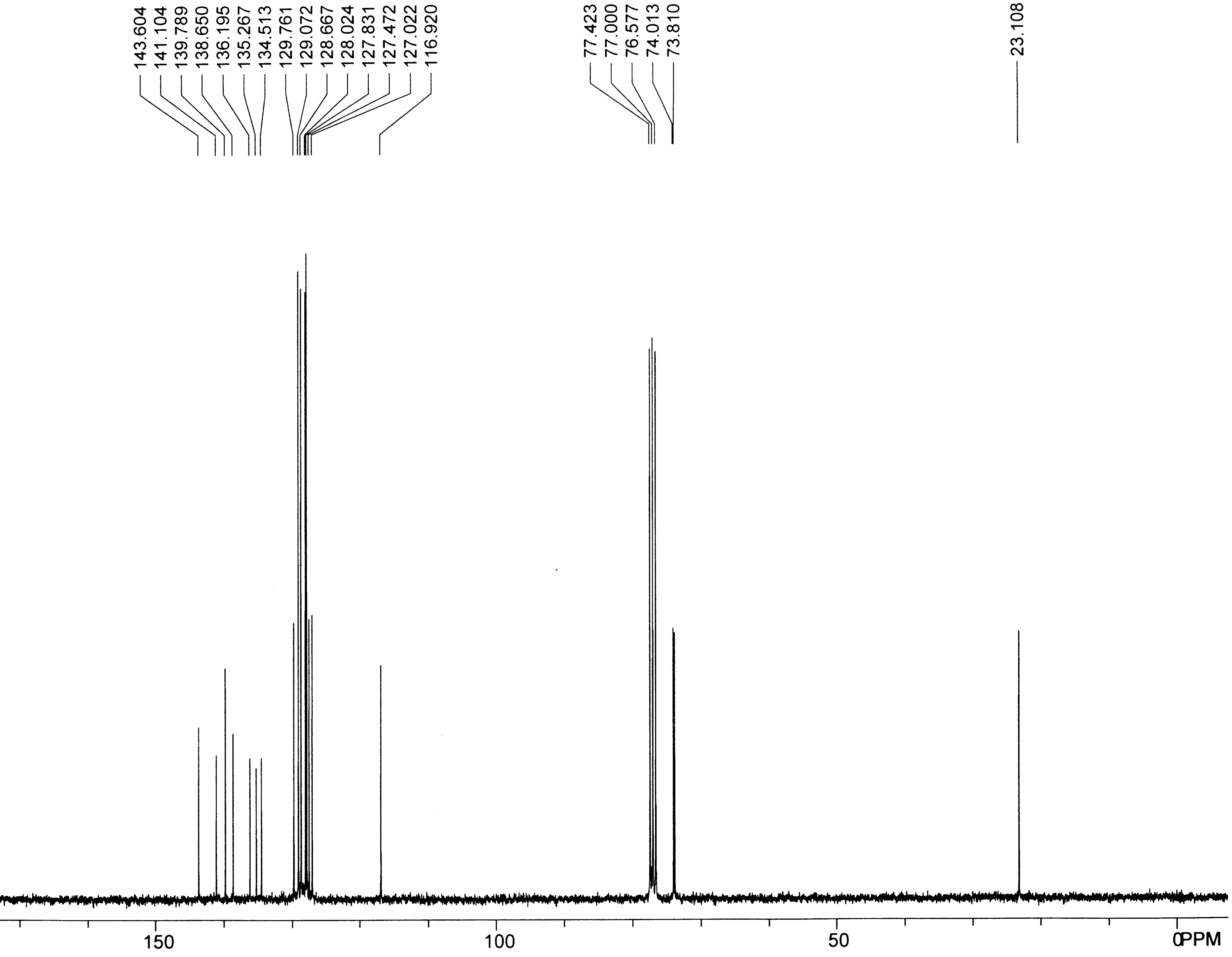

2017-12-24 12:17:19.265
zyc-1-113c
SOLVENT: CDCl3
Experiment = zgpg30
Pulse length = 9.500 usec
Relaxation delay = 2.000 sec
NA = 789
F1 = 75.467751 MHz
F2 = 1.000000 MHz

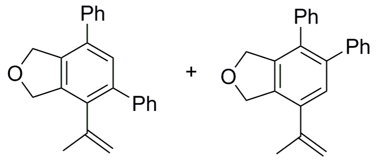

5ah **6ah**

5ah/6ah = 96/4

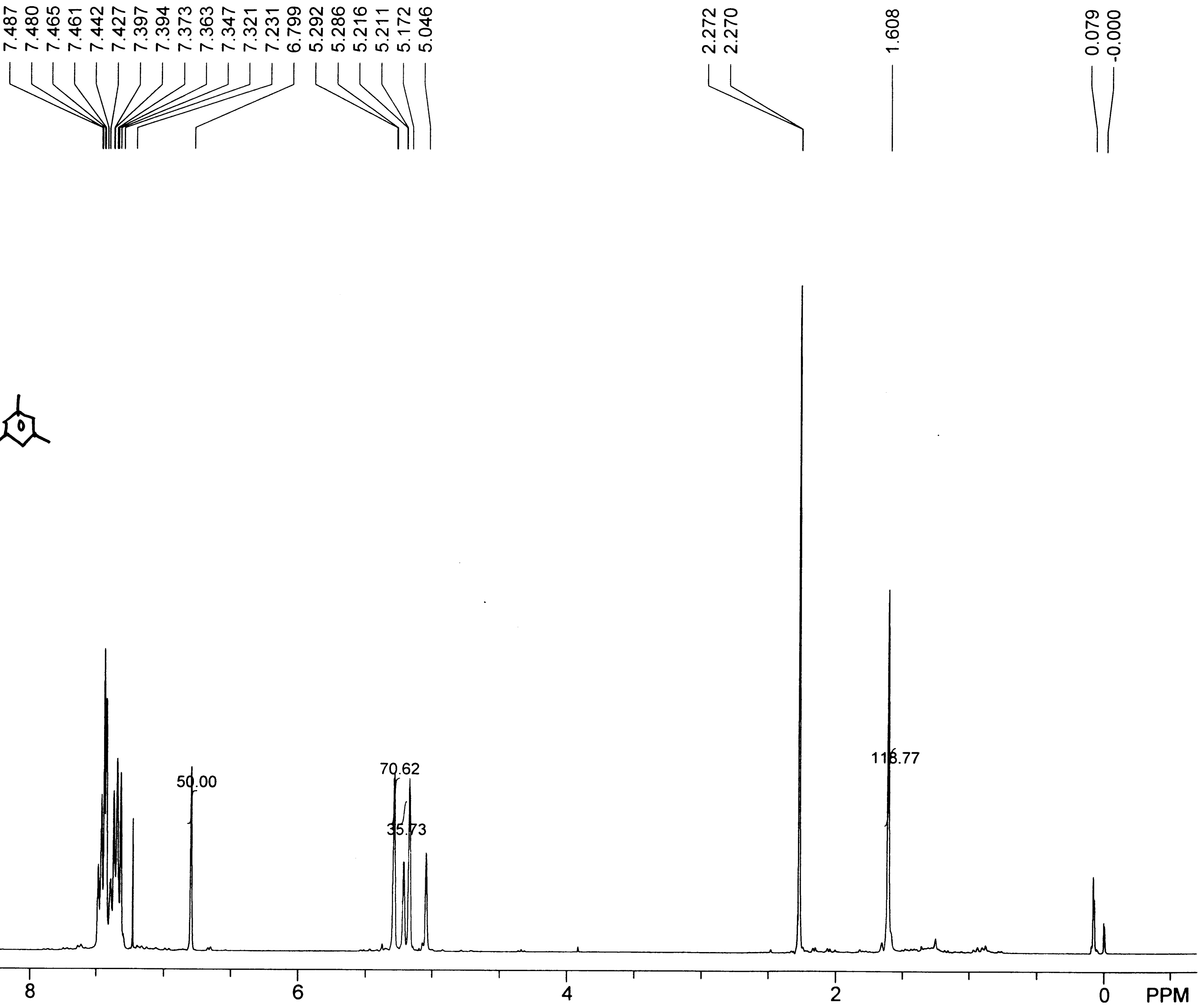

2017-12-31 21:01:18.109
zyc-1-113purity
SOLVENT: CDCl3
Experiment = zg30
Pulse length = 14.000 usec
Relaxation delay = 1.000 sec
NA = 16
F1 = 300.130005 MHz
F2 = 1.000000 MHz

119.7 mg sample added 23 μL

$$\text{purity} = \frac{35.73}{\frac{119.73}{312.41}} = 93\%$$

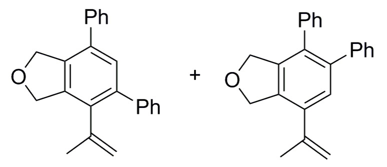

5ah 6ah

5ah/6ah = 96/4

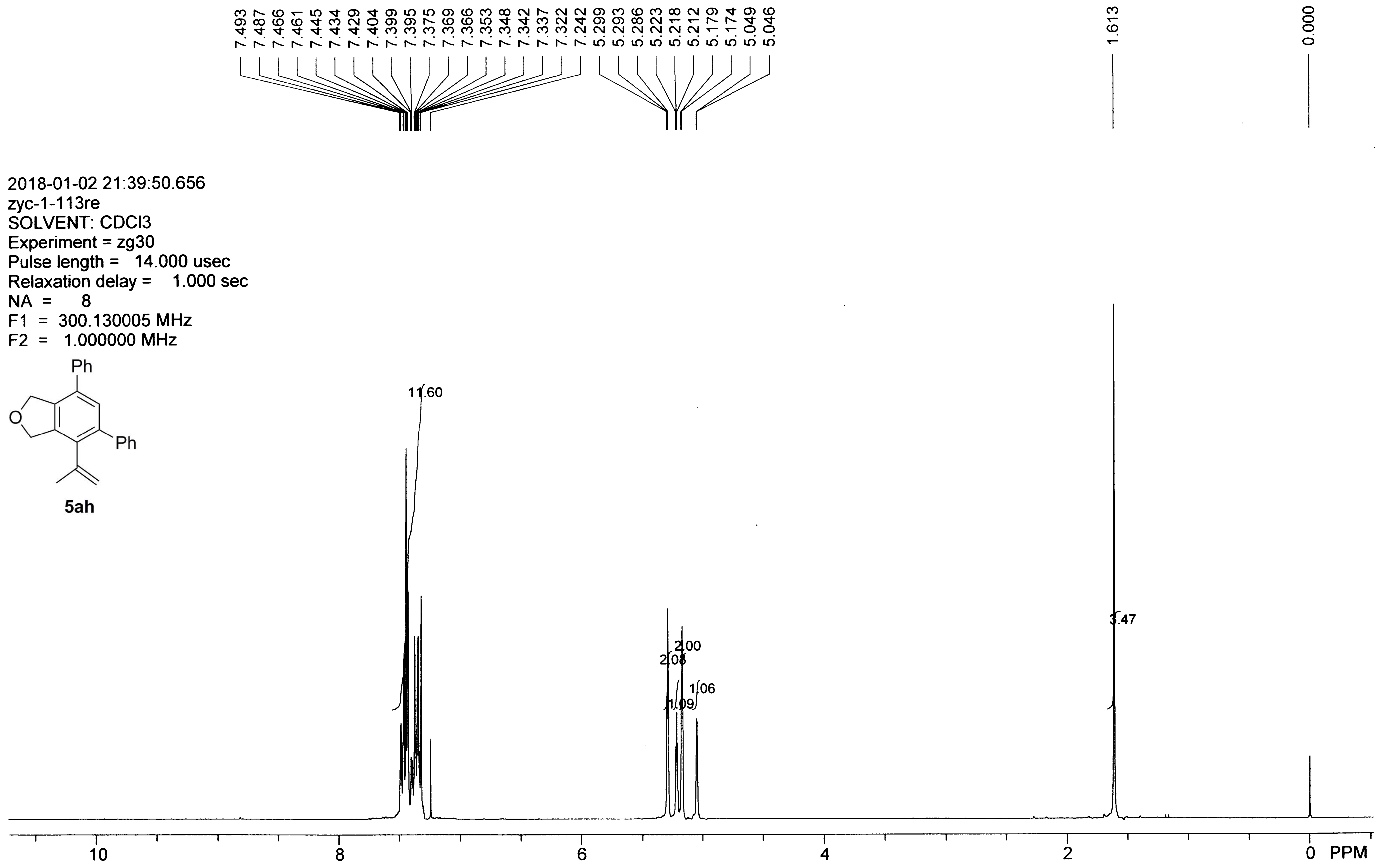
7.493
7.487
7.466
7.461
7.445
7.434
7.429
7.404
7.399
7.395
7.375
7.369
7.366
7.353
7.348
7.342
7.337
7.322
7.242
5.299
5.293
5.286
5.223
5.218
5.212
5.179
5.174
5.049
5.046
1.613
0.000
2018-01-02 21:39:50.656
zyc-1-113re
SOLVENT: CDCl3
Experiment = zg30
Pulse length = 14.000 usec
Relaxation delay = 1.000 sec
NA = 8
F1 = 300.130005 MHz
F2 = 1.000000 MHz
Ph
O
Ph
11.60
2.00
2.08
1.09
1.06
3.47
10
8
6
4
2
0 PPM

5ah

2018-05-27 10:20:37.468
zyc-2-28-2H
SOLVENT: CDCl3
Experiment = zg30
Pulse length = 14.000 usec
Relaxation delay = 1.000 sec
NA = 8
F1 = 300.130005 MHz
F2 = 1.000000 MHz

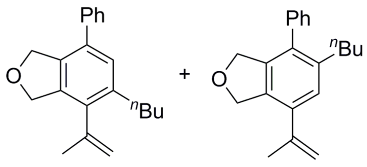

5ai **6ai**

5ai/6ai = 95/5

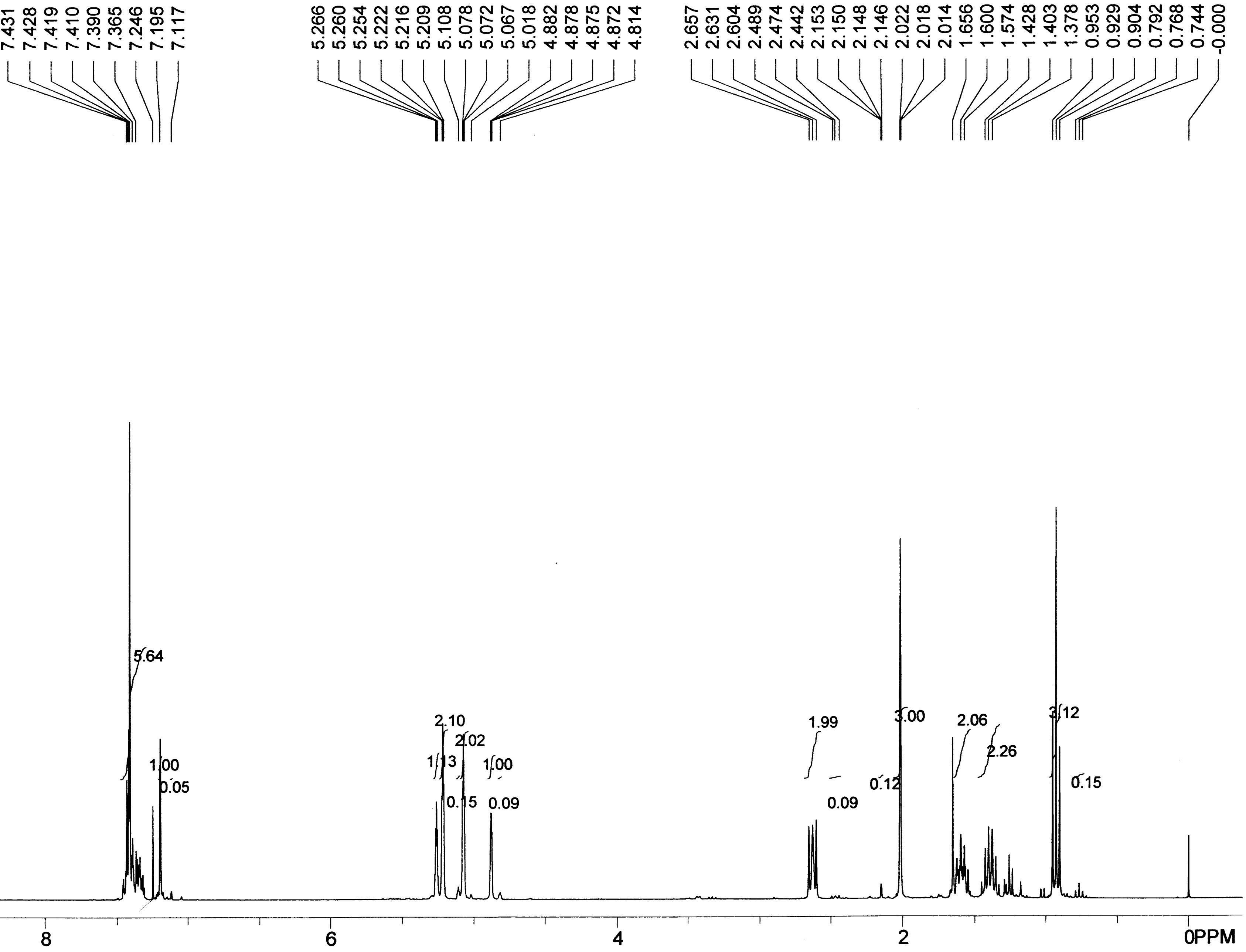

2018-05-25 21:59:06.421
zyc-2-28-2
SOLVENT: CDCl3
Experiment = zgpg30
Pulse length =   9.500 usec
Relaxation delay =   2.000 sec
NA =   102
F1 = 75.467751 MHz
F2 =   1.000000 MHz

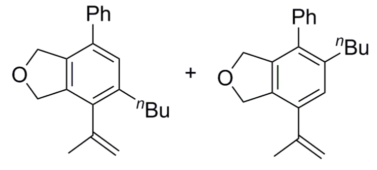

5ai **6ai**

5ai/6ai = 95/5

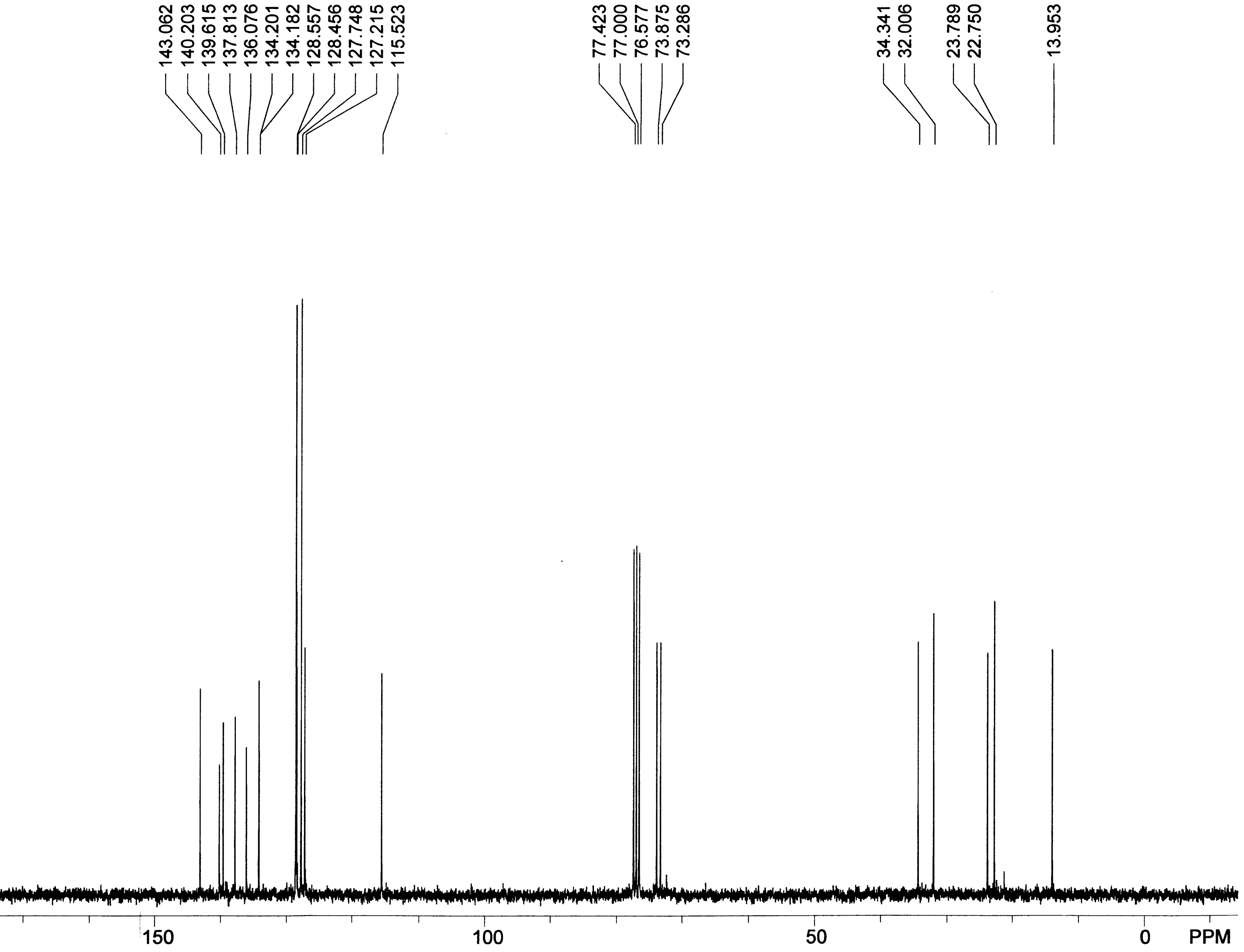

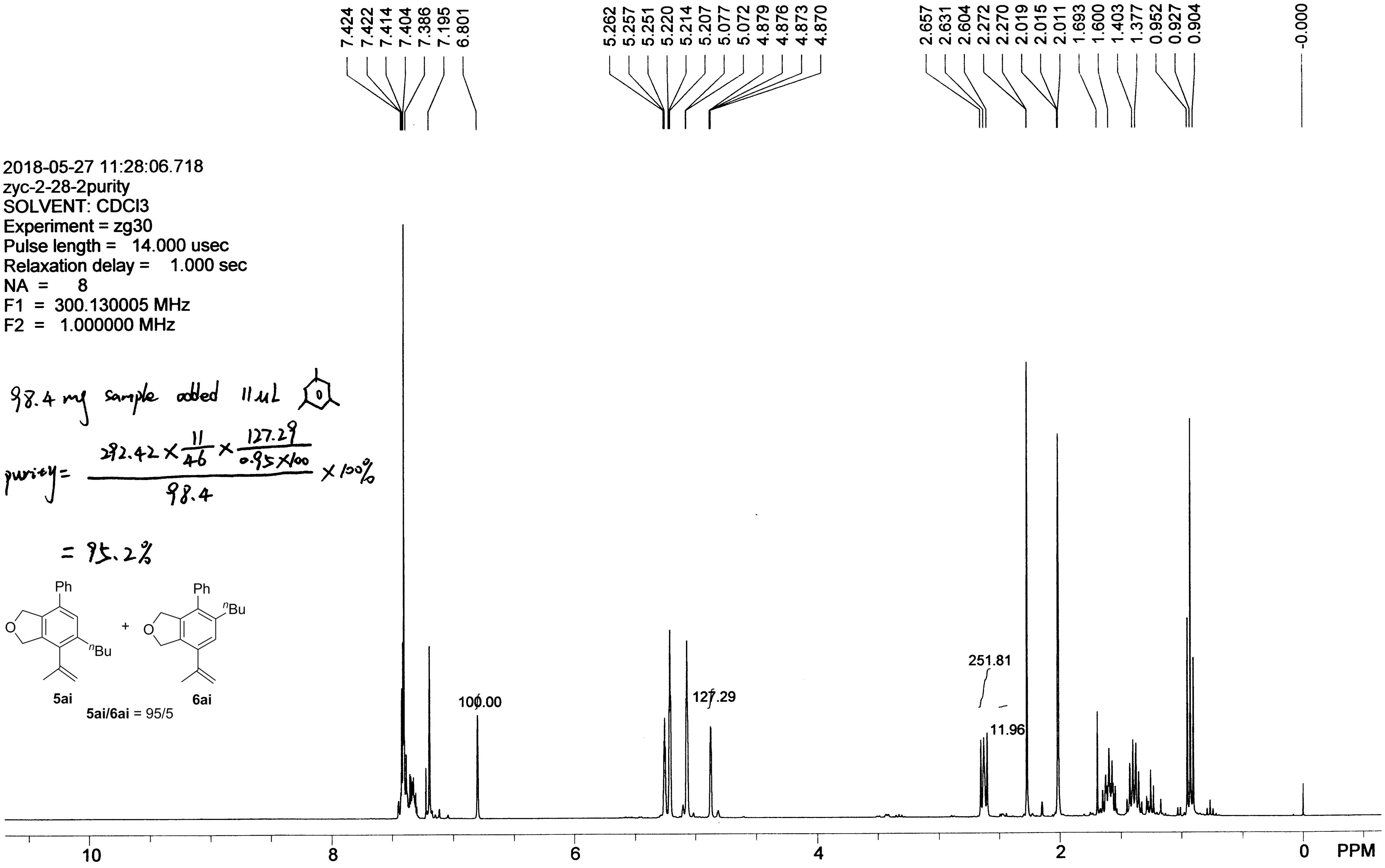
7.424
7.422
7.414
7.404
7.386
7.195
6.801
5.262
5.257
5.251
5.220
5.214
5.207
5.077
5.072
4.879
4.876
4.873
4.870
2.657
2.631
2.604
2.272
2.270
2.019
2.015
2.011
1.693
1.600
1.403
1.377
0.952
0.927
0.904
-0.000
2018-05-27 11:28:06.718
zyc-2-28-2purity
SOLVENT: CDCl3
Experiment = zg30
Pulse length = 14.000 usec
Relaxation delay = 1.000 sec
NA = 8
F1 = 300.130005 MHz
F2 = 1.000000 MHz
98.4 mg sample added 11 uL
purity = 292.42 × 11/46 × 127.29/(0.95×100) / 98.4 × 100%
= 95.2%
Ph
nBu
+
5ai
6ai
5ai/6ai = 95/5
100.00
127.29
251.81
11.96
10
8
6
4
2
0
PPM

2018-06-01 09:34:08.187
zyc-2-35
SOLVENT: CDCl3
Experiment = zg30
Pulse length = 14.000 usec
Relaxation delay = 1.000 sec
NA = 8
F1 = 300.130005 MHz
F2 = 1.000000 MHz

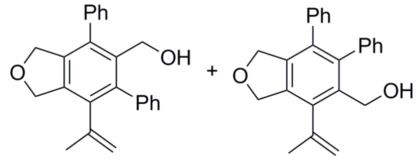

5aj **6aj**

5aj/6aj > 99/1

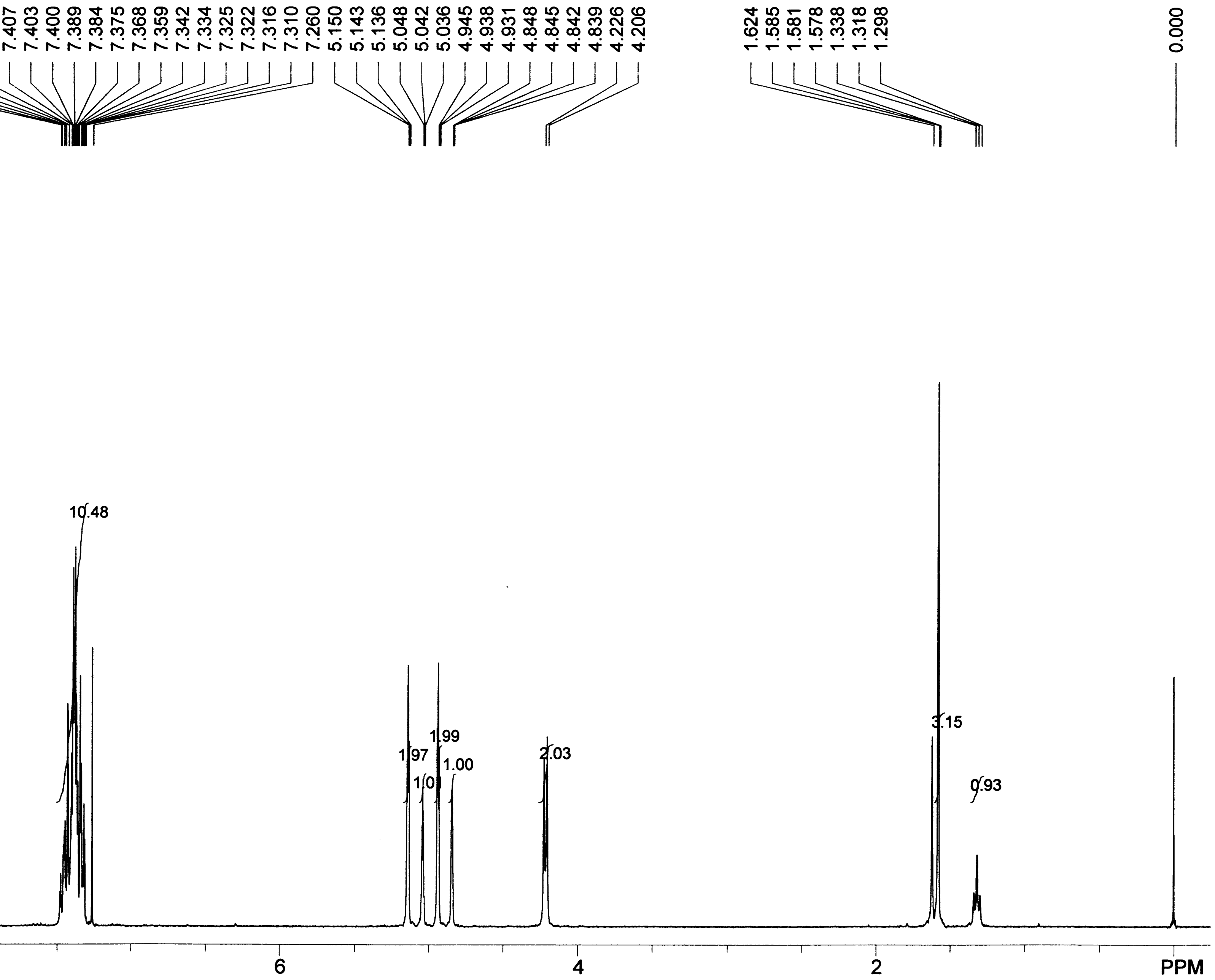

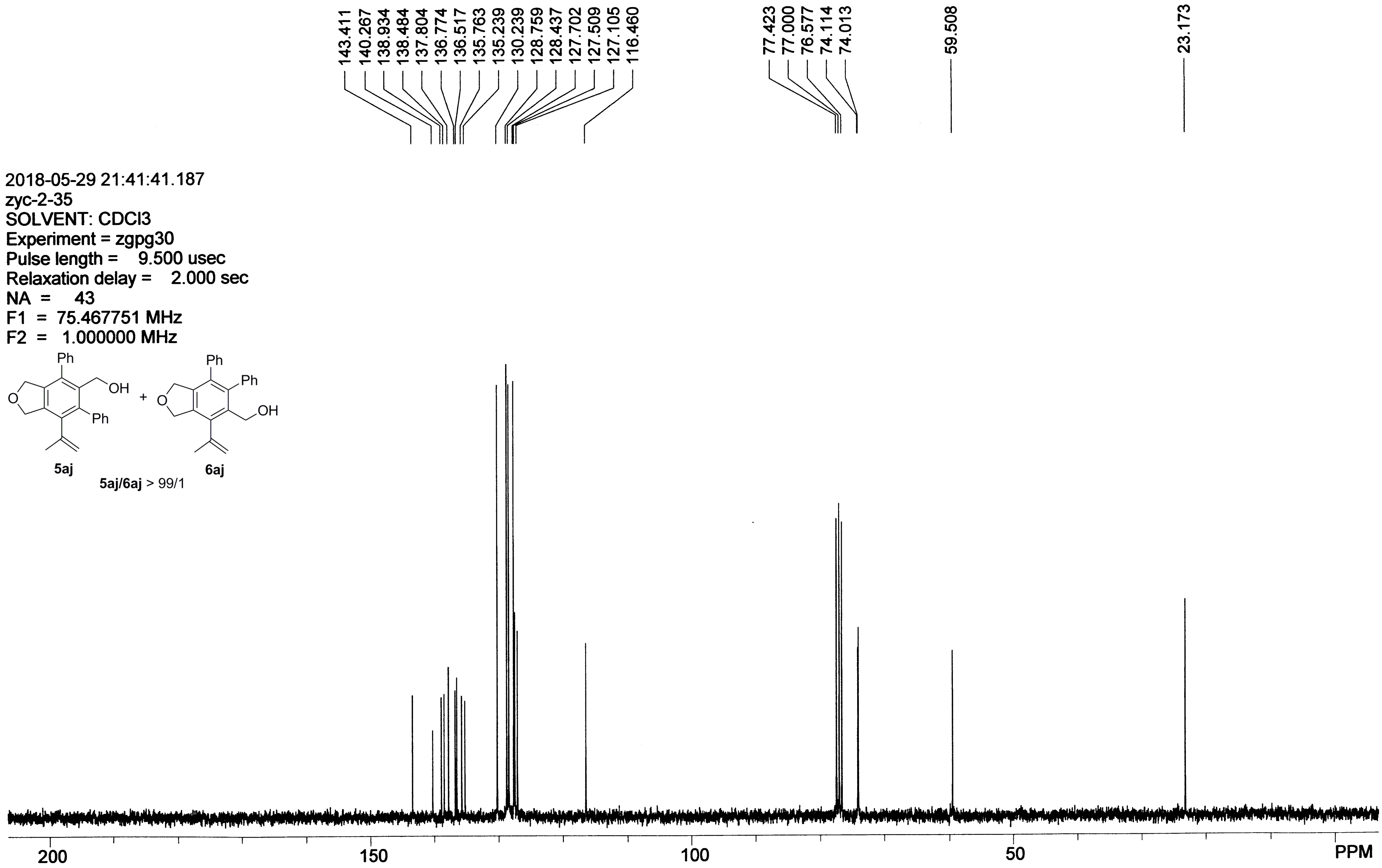
143.411
140.267
138.934
138.484
137.804
136.774
136.517
135.763
135.239
130.239
128.759
128.437
127.702
127.509
127.105
116.460
77.423
77.000
76.577
74.114
74.013
59.508
23.173
2018-05-29 21:41:41.187
zyc-2-35
SOLVENT: CDCl3
Experiment = zgpg30
Pulse length = 9.500 usec
Relaxation delay = 2.000 sec
NA = 43
F1 = 75.467751 MHz
F2 = 1.000000 MHz
Ph
OH
Ph
+
Ph
Ph
OH
5aj
6aj
5aj/6aj > 99/1
200
150
100
50
PPM

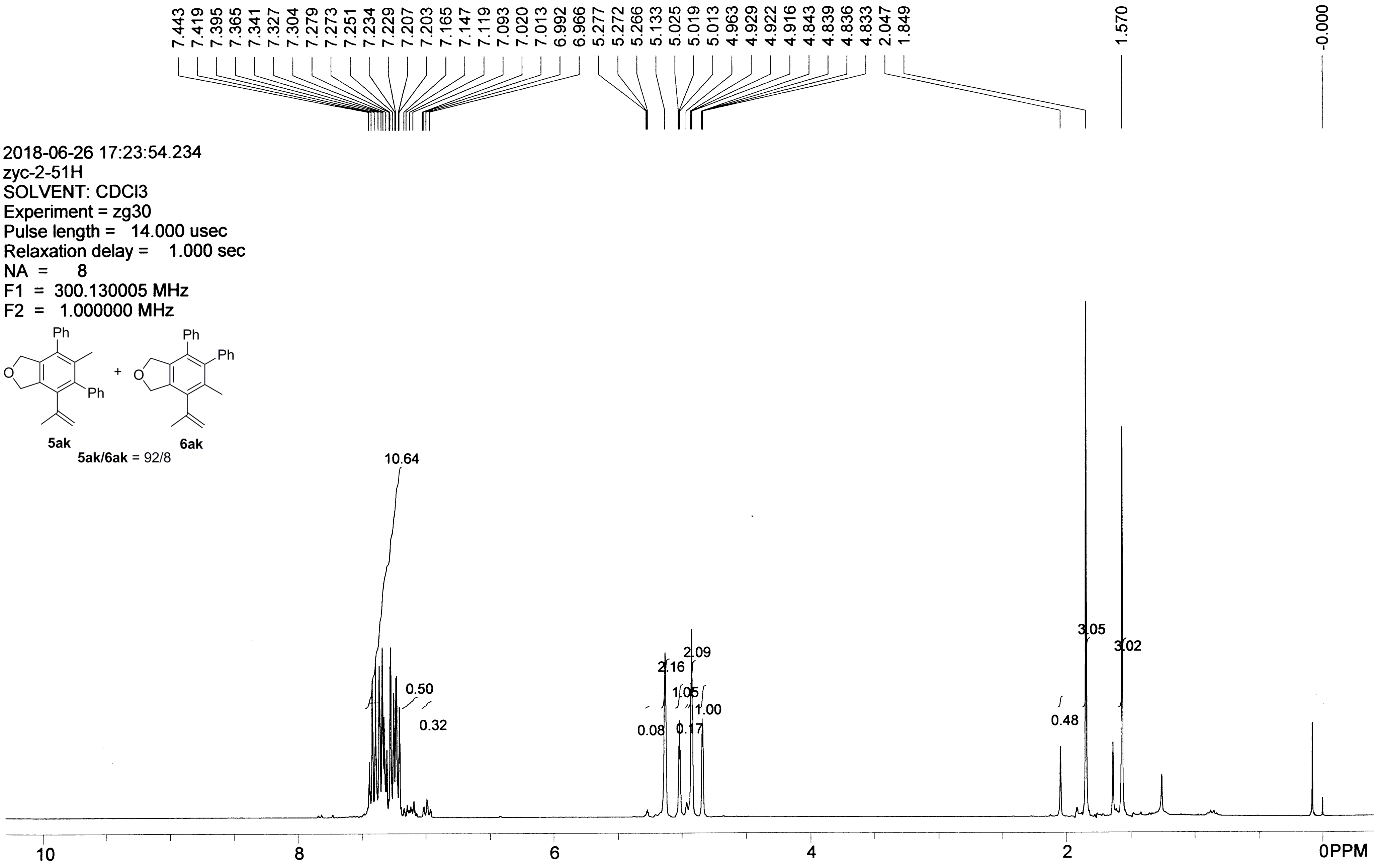

5ak **6ak**

5ak/6ak = 92/8

2018-06-26 18:56:54.484
zyc-2-51
SOLVENT: CDCl3
Experiment = zgpg30
Pulse length = 9.500 usec
Relaxation delay = 2.000 sec
NA = 1561
F1 = 75.467751 MHz
F2 = 1.000000 MHz

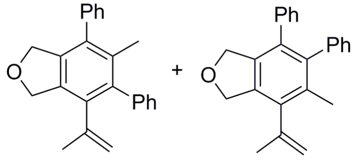

5ak **6ak**

5ak/6ak = 92/8

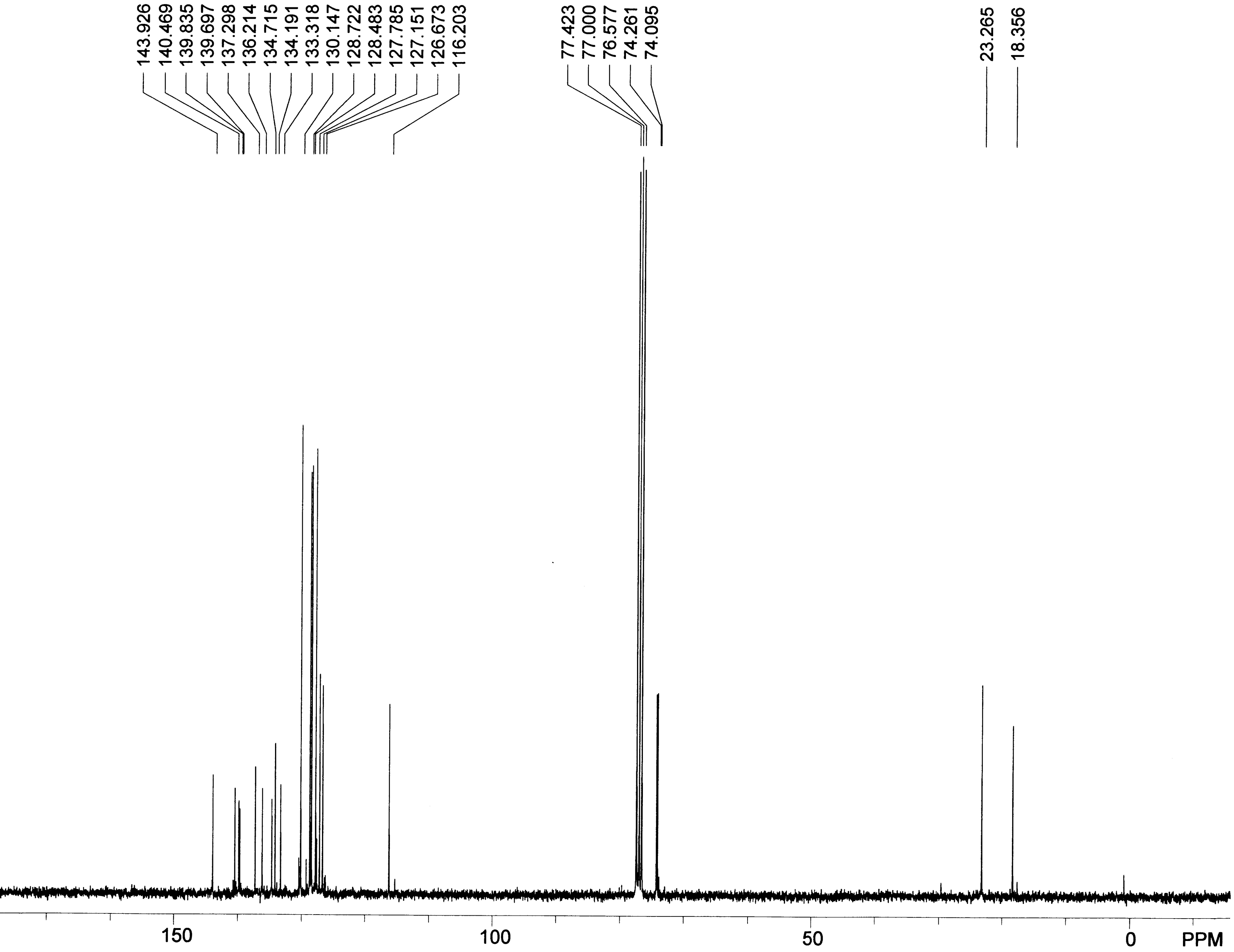

2018-07-10 16:39:25.046
zyc-2-60crystal
SOLVENT: CDCl3
Experiment = zg30
Pulse length = 14.000 usec
Relaxation delay = 1.000 sec
NA = 8
F1 = 300.130005 MHz
F2 = 1.000000 MHz

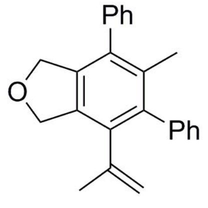

5ak

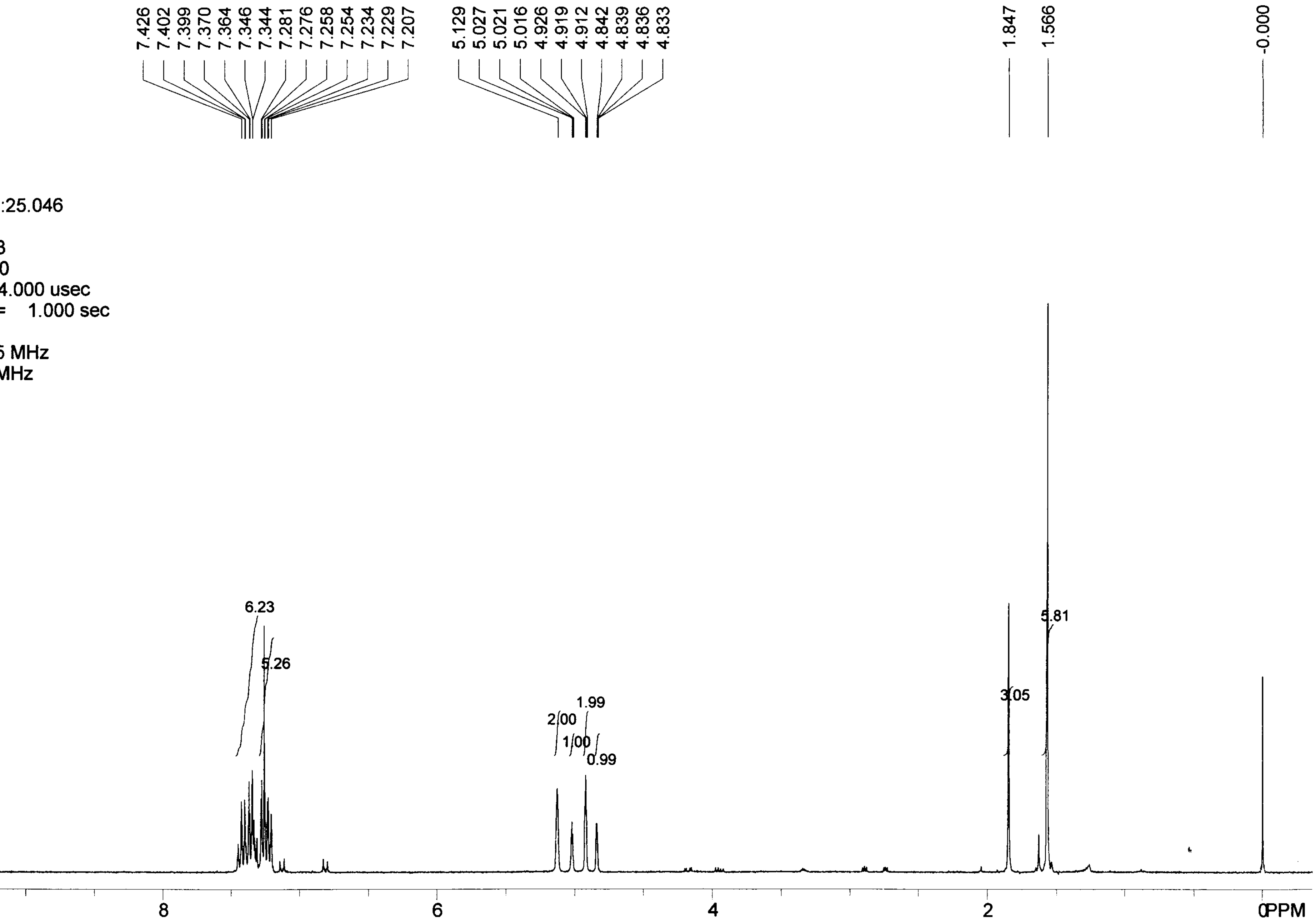

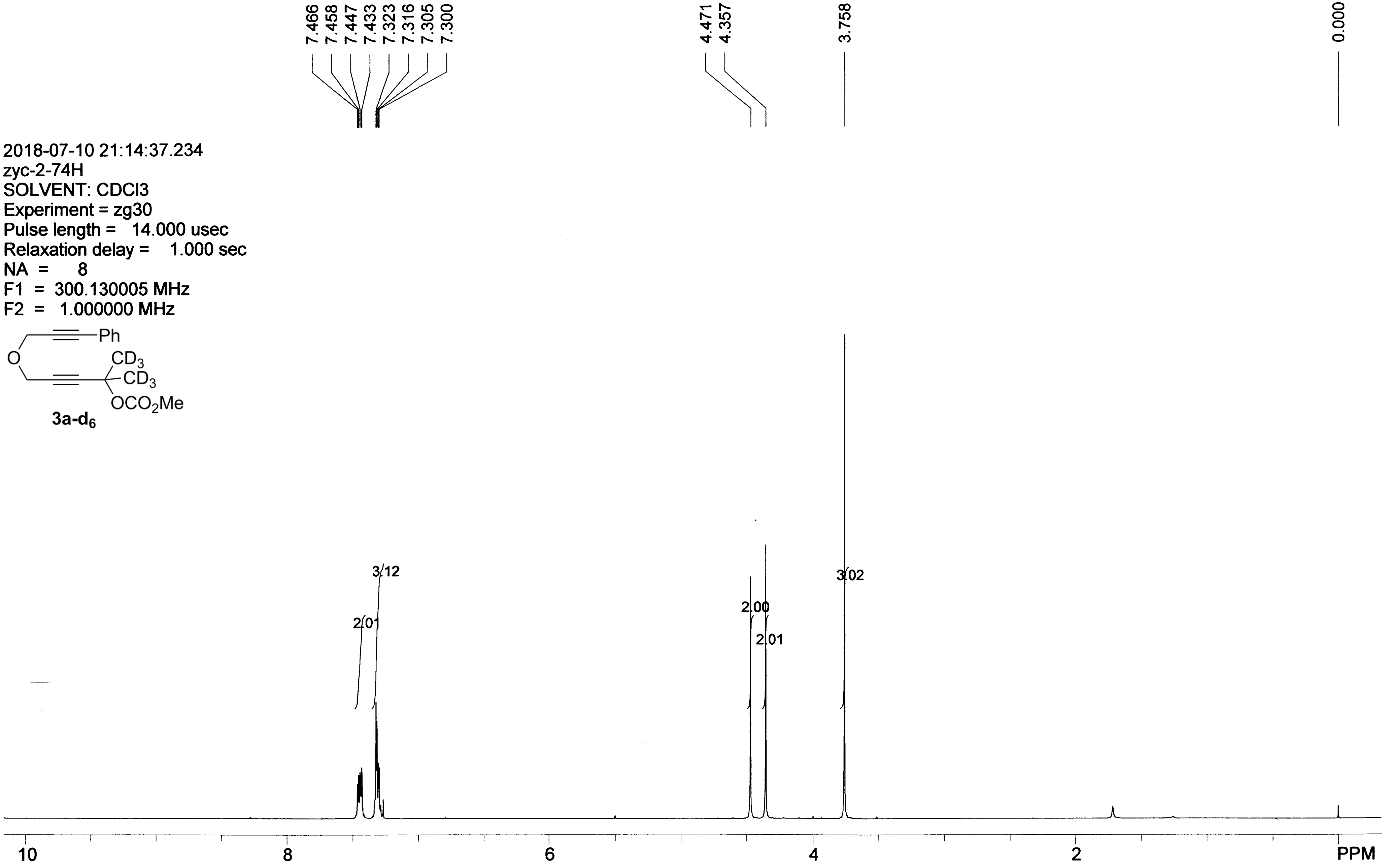
7.466
7.458
7.447
7.433
7.323
7.316
7.305
7.300
4.471
4.357
3.758
0.000
2018-07-10 21:14:37.234
zyc-2-74H
SOLVENT: CDCl3
Experiment = zg30
Pulse length = 14.000 usec
Relaxation delay = 1.000 sec
NA = 8
F1 = 300.130005 MHz
F2 = 1.000000 MHz
Ph
O
CD3
CD3
OCO2Me
3a-d6
2.01
3.12
2.00
2.01
3.02
10
8
6
4
2
PPM

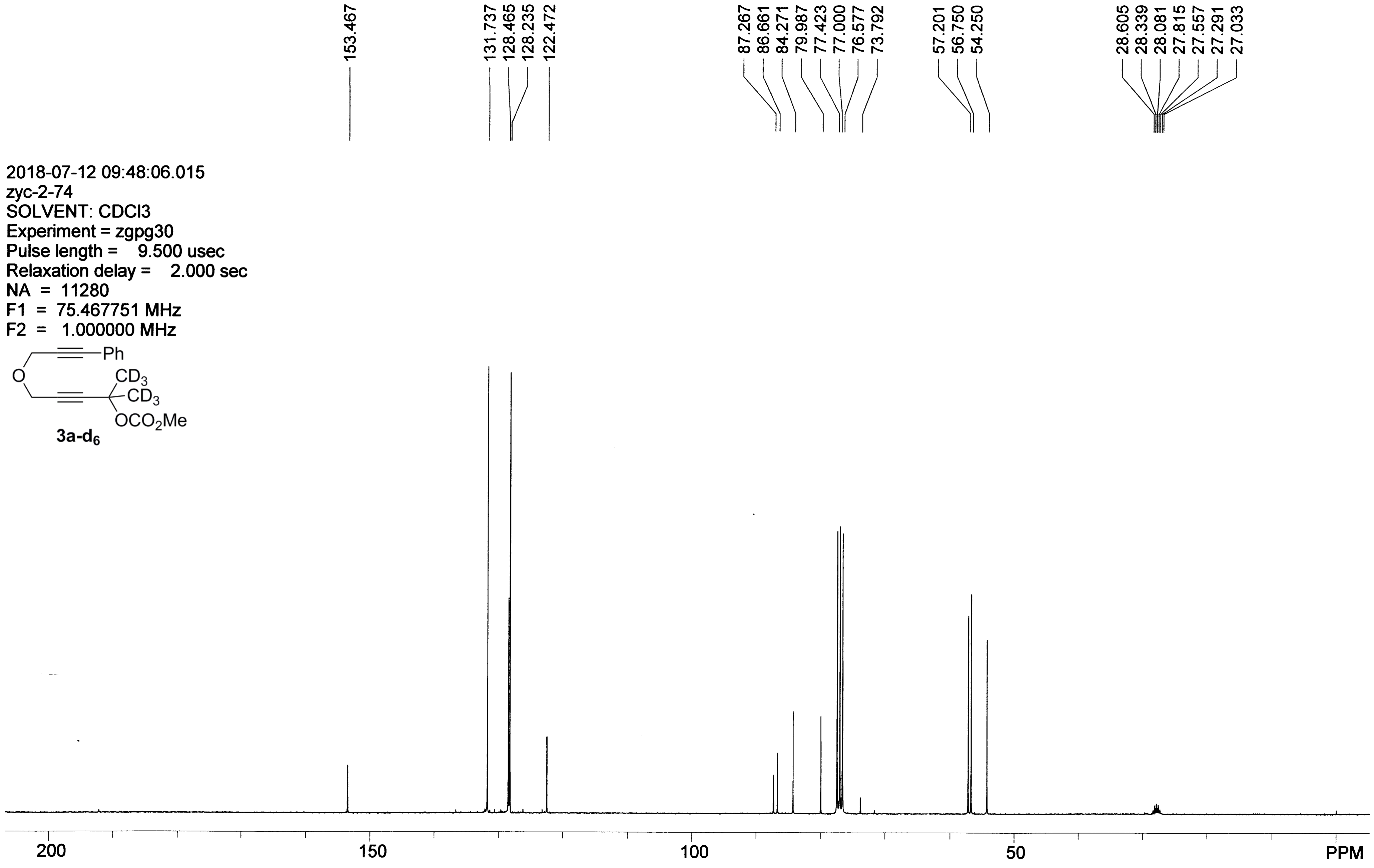

2018-07-12 09:48:06.015
zyc-2-74
SOLVENT: CDCl3
Experiment = zgpg30
Pulse length = 9.500 usec
Relaxation delay = 2.000 sec
NA = 11280
F1 = 75.467751 MHz
F2 = 1.000000 MHz

3a-d$_6$

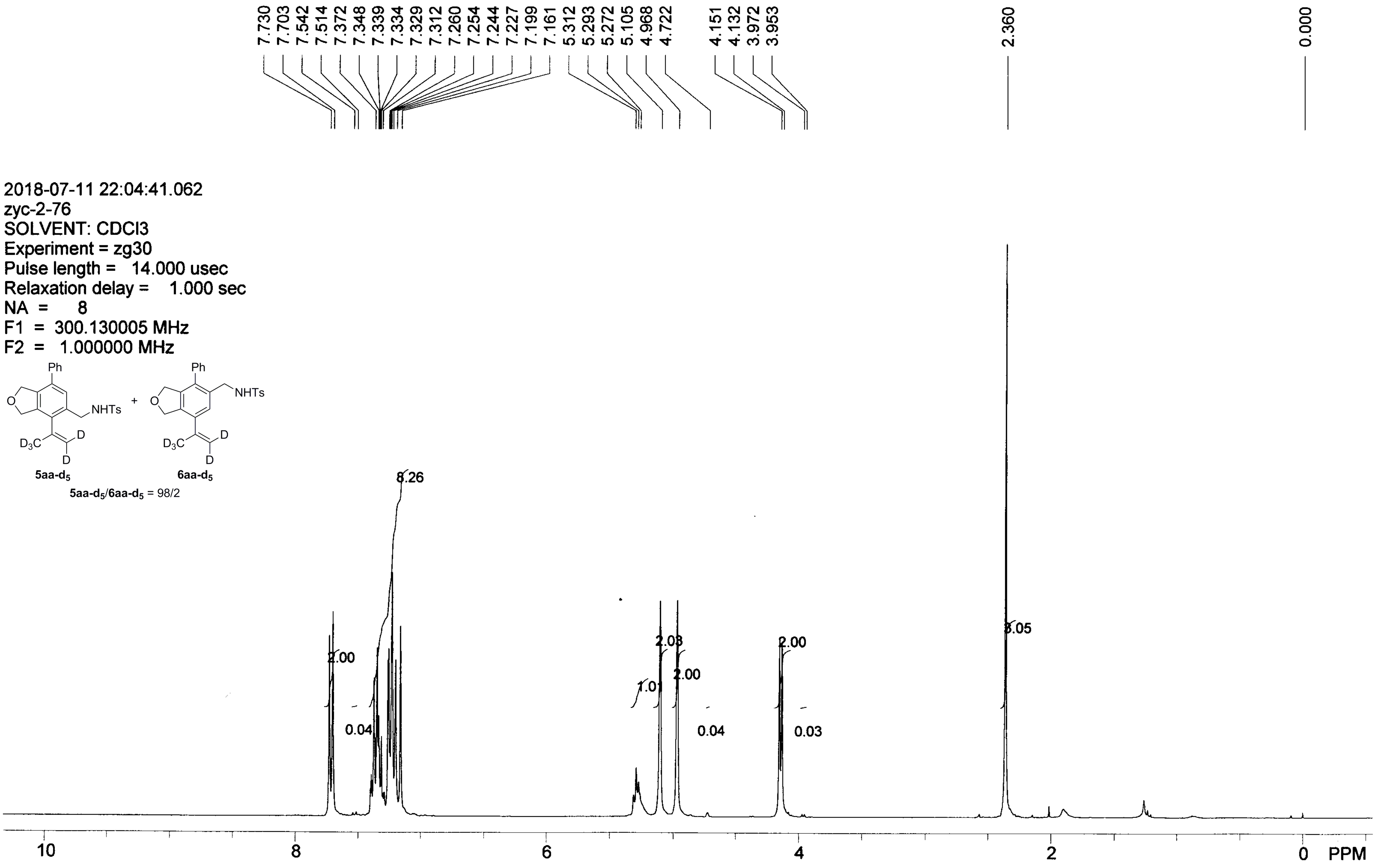
7.730
7.703
7.542
7.514
7.372
7.348
7.339
7.334
7.329
7.312
7.260
7.254
7.244
7.227
7.199
7.161
5.312
5.293
5.272
5.105
4.968
4.722
4.151
4.132
3.972
3.953
2.360
0.000
2018-07-11 22:04:41.062
zyc-2-76
SOLVENT: CDCl3
Experiment = zg30
Pulse length = 14.000 usec
Relaxation delay = 1.000 sec
NA = 8
F1 = 300.130005 MHz
F2 = 1.000000 MHz
Ph
NHTs
D
D_3C
D
5aa-d_5
6aa-d_5
5aa-d_5/6aa-d_5 = 98/2
8.26
2.00
0.04
2.03
1.01
2.00
0.04
2.00
0.03
3.05
10
8
6
4
2
0
PPM

2018-07-13 09:14:55.328
zyc-2-76C
SOLVENT: CDCl3
Experiment = zgpg30
Pulse length = 9.500 usec
Relaxation delay = 2.000 sec
NA = 11005
F1 = 75.467751 MHz
F2 = 1.000000 MHz

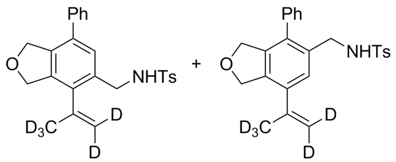

5aa-d_5 **6aa-d_5**

5aa-d_5/6aa-d_5 = 98/2

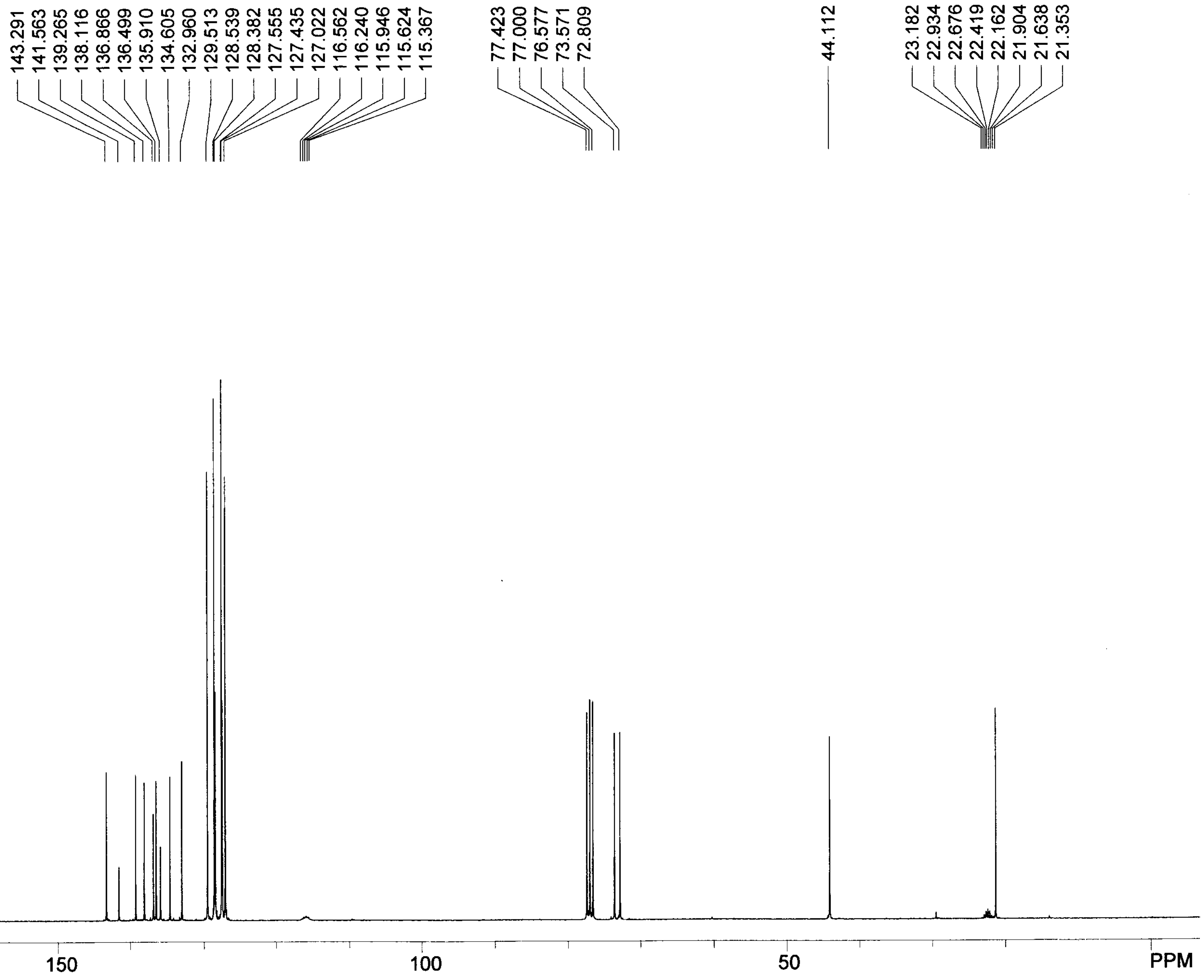

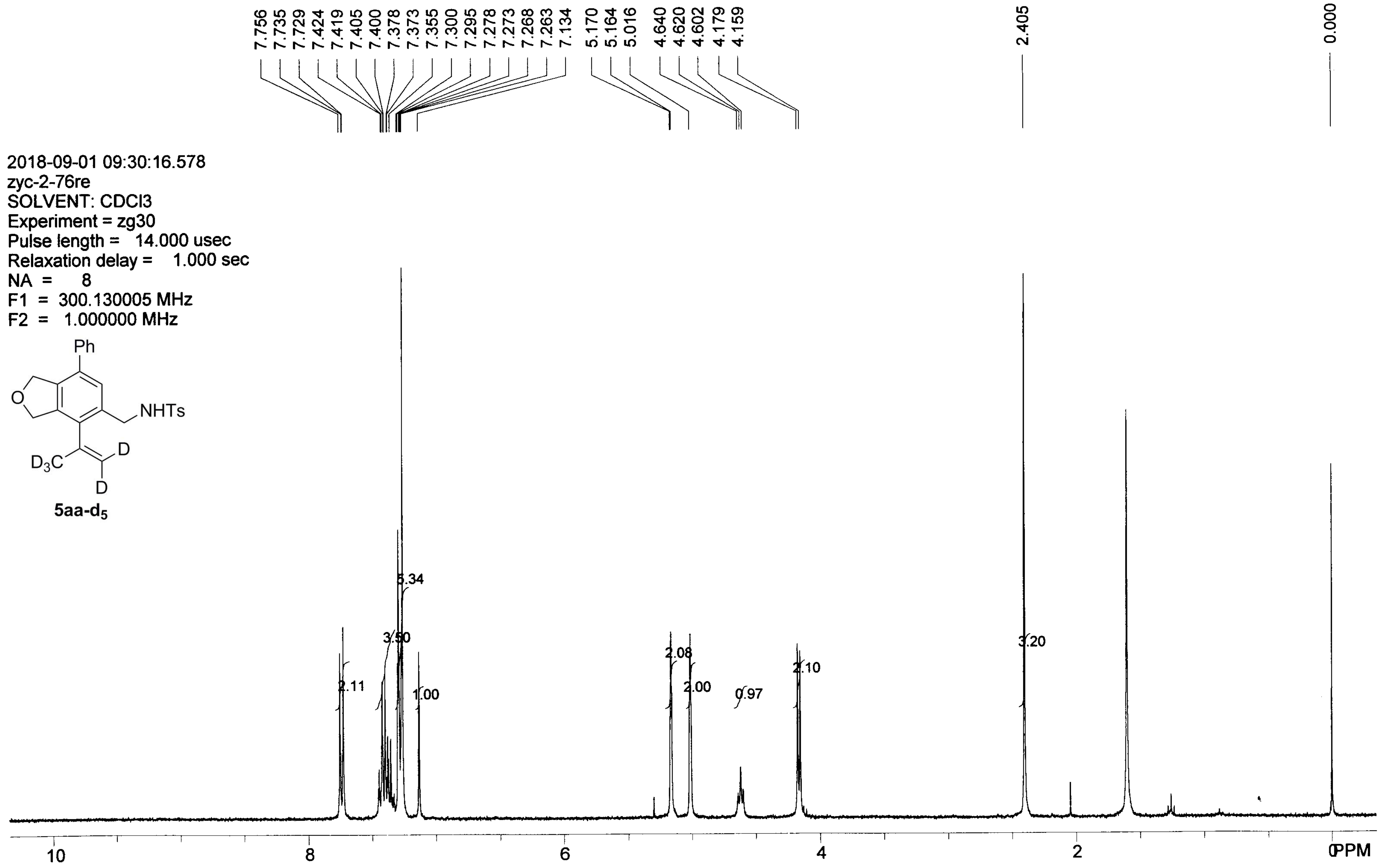

7.756
7.735
7.729
7.424
7.419
7.405
7.400
7.378
7.373
7.355
7.300
7.295
7.278
7.273
7.268
7.263
7.134
5.170
5.164
5.016
4.640
4.620
4.602
4.179
4.159
2.405
0.000
2018-09-01 09:30:16.578
zyc-2-76re
SOLVENT: CDCl3
Experiment = zg30
Pulse length = 14.000 usec
Relaxation delay = 1.000 sec
NA = 8
F1 = 300.130005 MHz
F2 = 1.000000 MHz
Ph
O
NHTs
D
D_3C
D
5aa-d_5
2.11
3.50
5.34
1.00
2.08
2.00
0.97
2.10
3.20
10
8
6
4
2
0
PPM

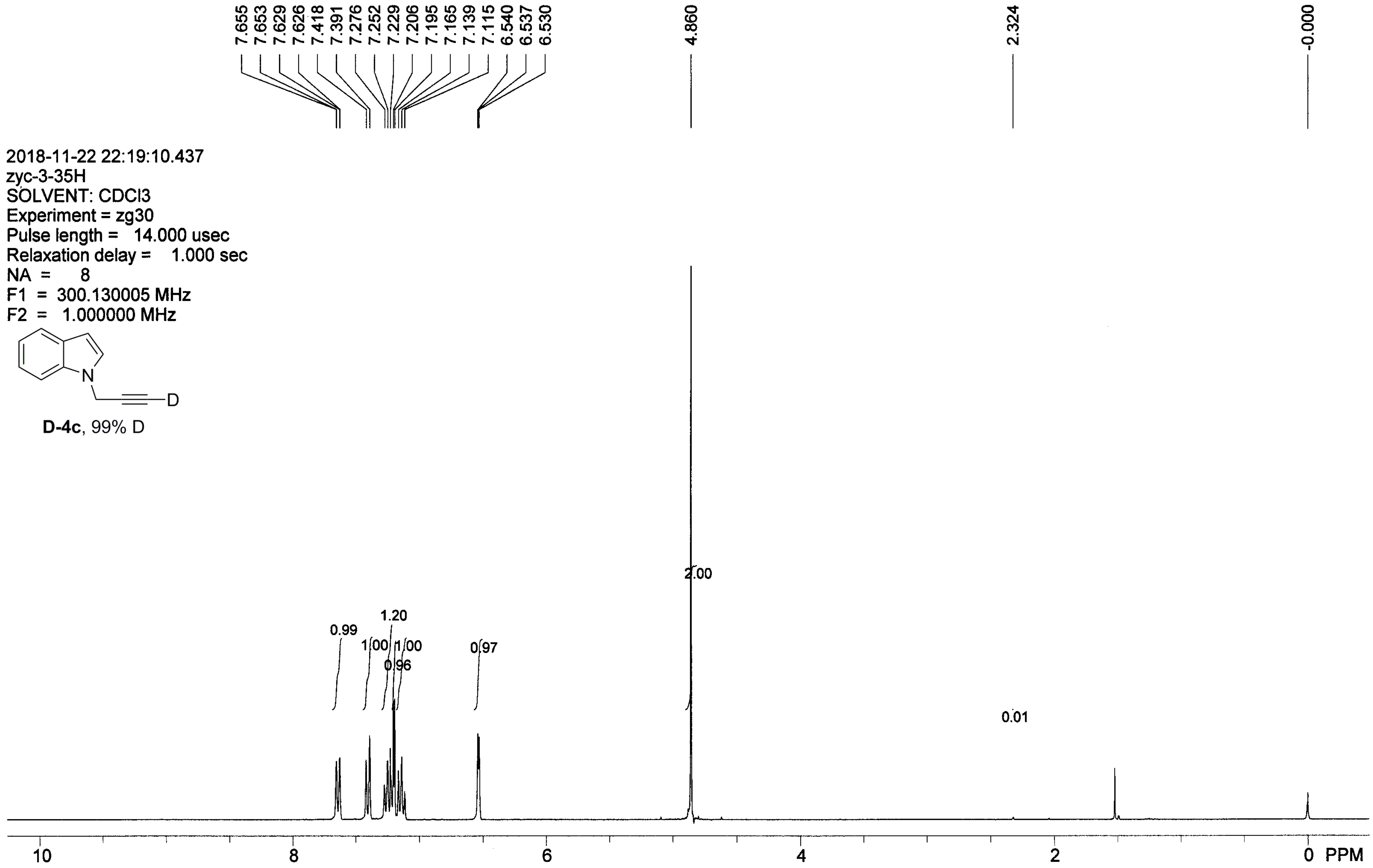
7.655
7.653
7.629
7.626
7.418
7.391
7.276
7.252
7.229
7.206
7.195
7.165
7.139
7.115
6.540
6.537
6.530
4.860
2.324
-0.000
2018-11-22 22:19:10.437
zyc-3-35H
SOLVENT: CDCl3
Experiment = zg30
Pulse length = 14.000 usec
Relaxation delay = 1.000 sec
NA = 8
F1 = 300.130005 MHz
F2 = 1.000000 MHz
N
D
0.99
1.20
1.00
1.00
0.96
0.97
2.00
0.01
10
8
6
4
2
0 PPM

D-4c, 99% D

2018-11-23 07:56:24.046
zyc-3-35
SOLVENT: CDCl3
Experiment = zgpg30
Pulse length = 9.500 usec
Relaxation delay = 2.000 sec
NA = 9867
F1 = 75.467751 MHz
F2 = 1.000000 MHz

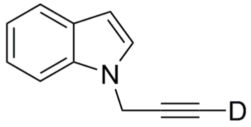

D-4c, 99% D

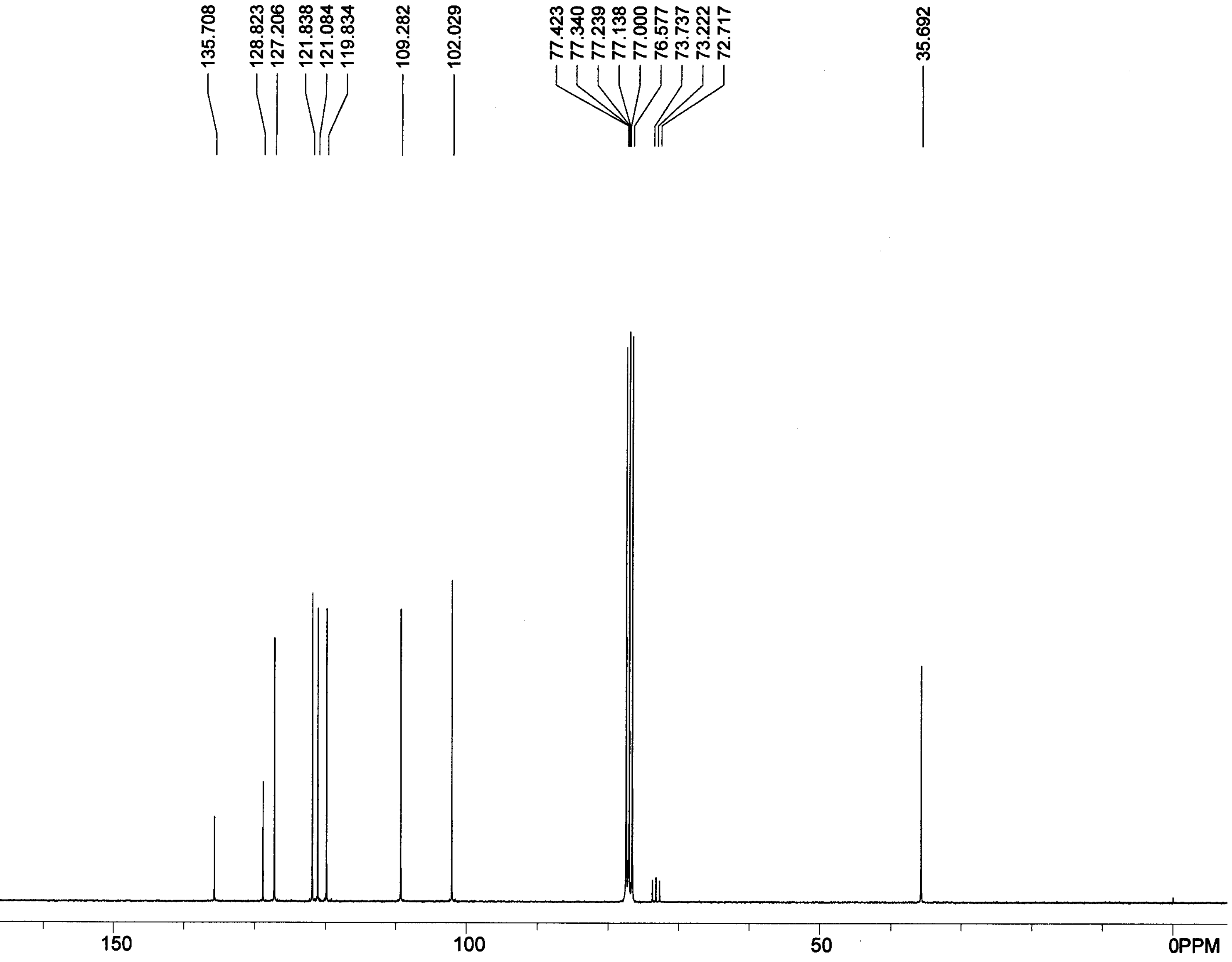

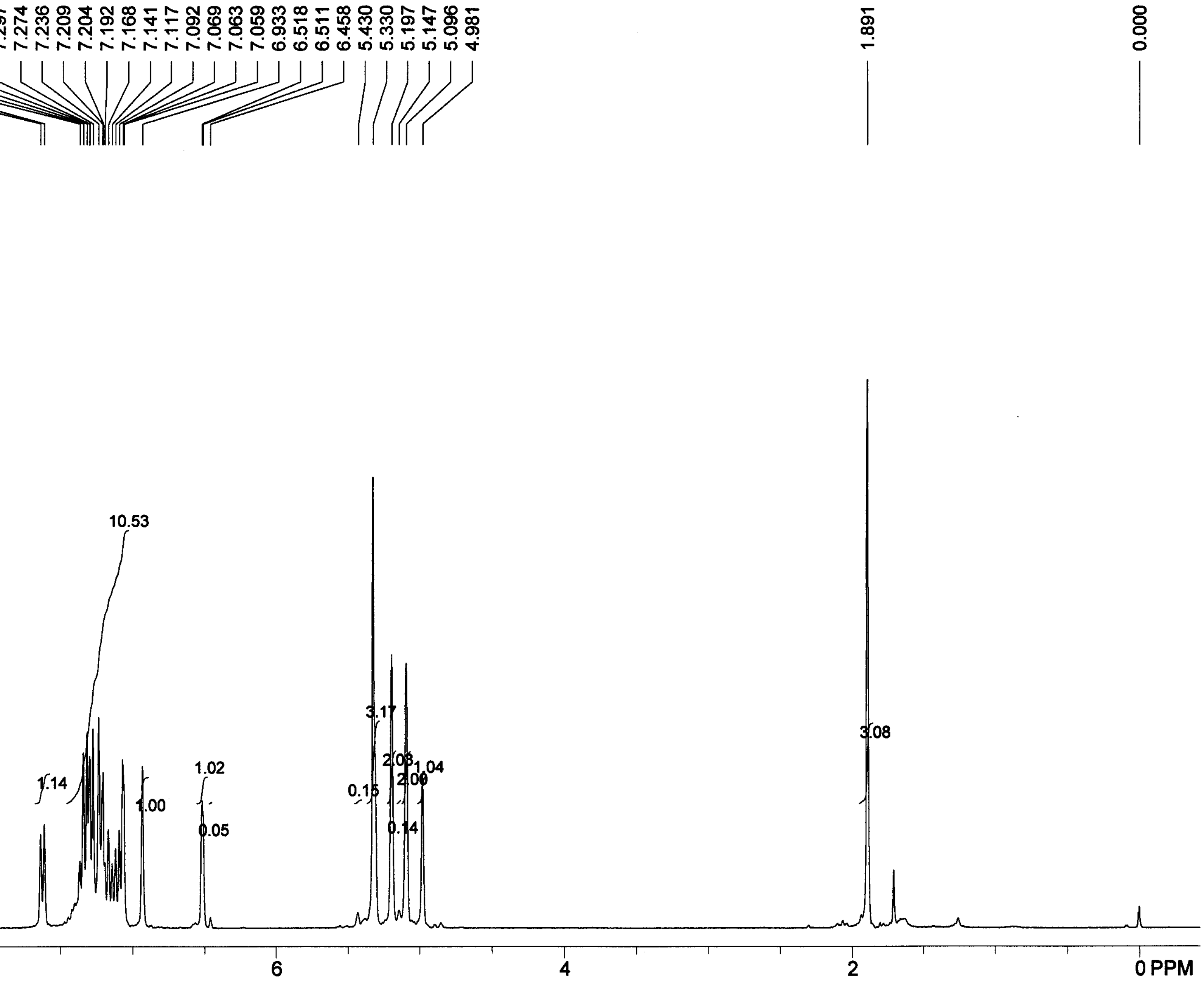

2018-11-24 13:15:56.671
zyc-3-36H
SOLVENT: CDCl3
Experiment = zg30
Pulse length = 14.000 usec
Relaxation delay = 1.000 sec
NA = 8
F1 = 300.130005 MHz
F2 = 1.000000 MHz

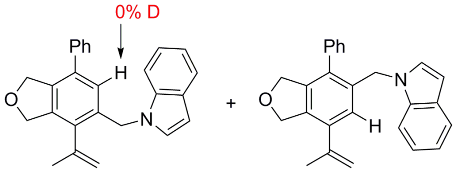

5ac 6ac

5ac/6ac = 95/5

2018-11-24 13:36:12.406
zyc-3-36
SOLVENT: CDCl3
Experiment = zgpg30
Pulse length = 9.500 usec
Relaxation delay = 2.000 sec
NA = 302
F1 = 75.467751 MHz
F2 = 1.000000 MHz

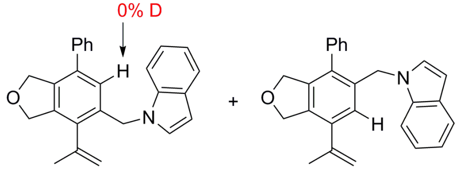

5ac 6ac

5ac/6ac = 95/5

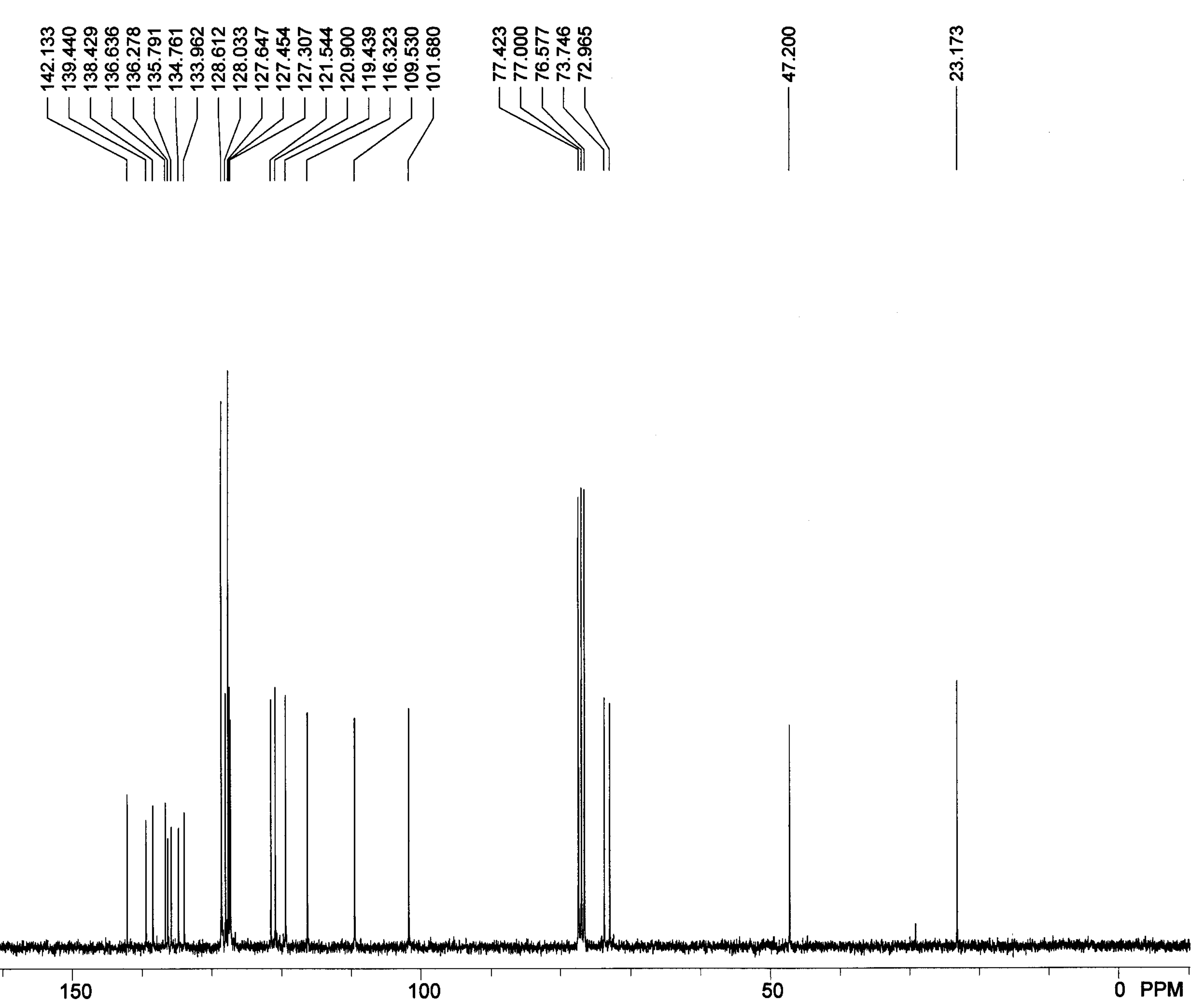

2018-11-27 22:22:54.078
zyc-3-46
SOLVENT: CDCl3
Experiment = zg30
Pulse length = 14.000 usec
Relaxation delay = 1.000 sec
NA = 8
F1 = 300.130005 MHz
F2 = 1.000000 MHz

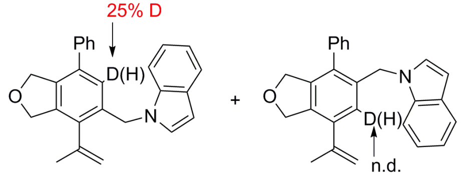

D-5ac
D-6ac
D-5ac/D-6ac = 81/19

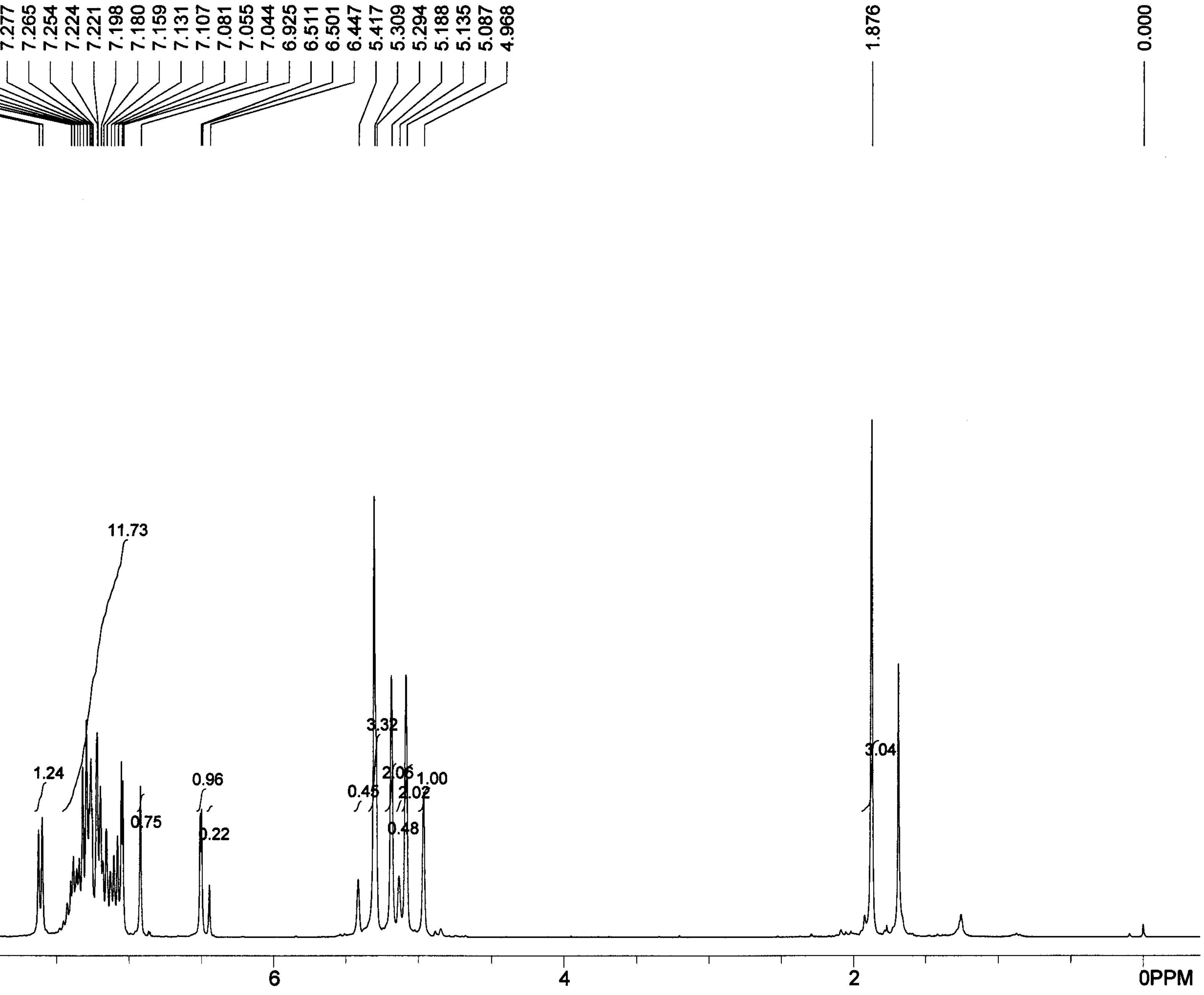

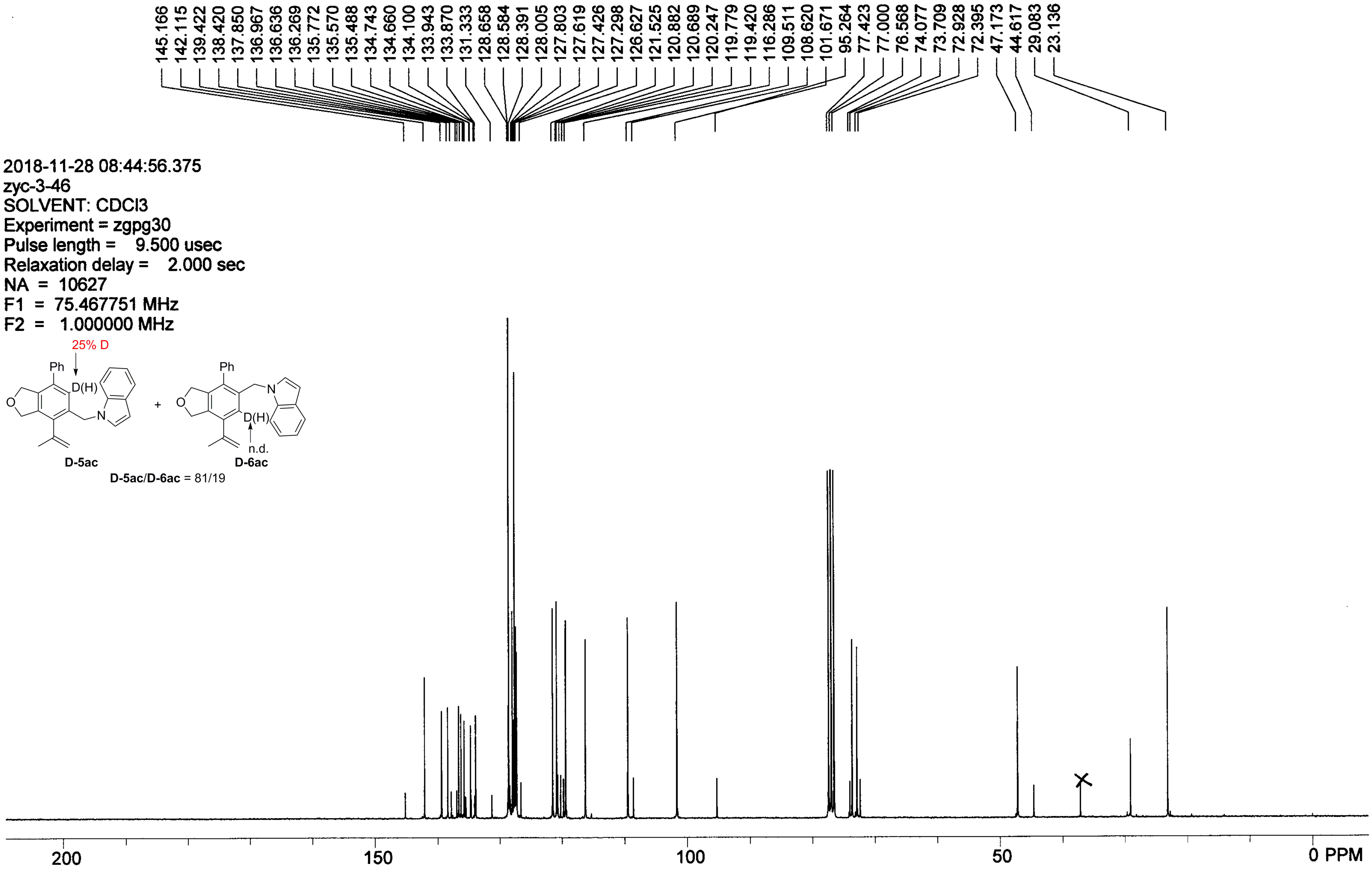
145.166
142.115
139.422
138.420
137.850
136.967
136.636
136.269
135.772
135.570
135.488
134.743
134.660
134.100
133.943
133.870
131.333
128.658
128.584
128.391
128.005
127.803
127.619
127.426
127.298
126.627
121.525
120.882
120.689
120.247
119.779
119.420
116.286
109.511
108.620
101.671
95.264
77.423
77.000
76.568
74.077
73.709
72.928
72.395
47.173
44.617
29.083
23.136
2018-11-28 08:44:56.375
zyc-3-46
SOLVENT: CDCl3
Experiment = zgpg30
Pulse length = 9.500 usec
Relaxation delay = 2.000 sec
NA = 10627
F1 = 75.467751 MHz
F2 = 1.000000 MHz
25% D
Ph
D(H)
N
O
+
n.d.
D-5ac
D-6ac
D-5ac/D-6ac = 81/19
200
150
100
50
0 PPM

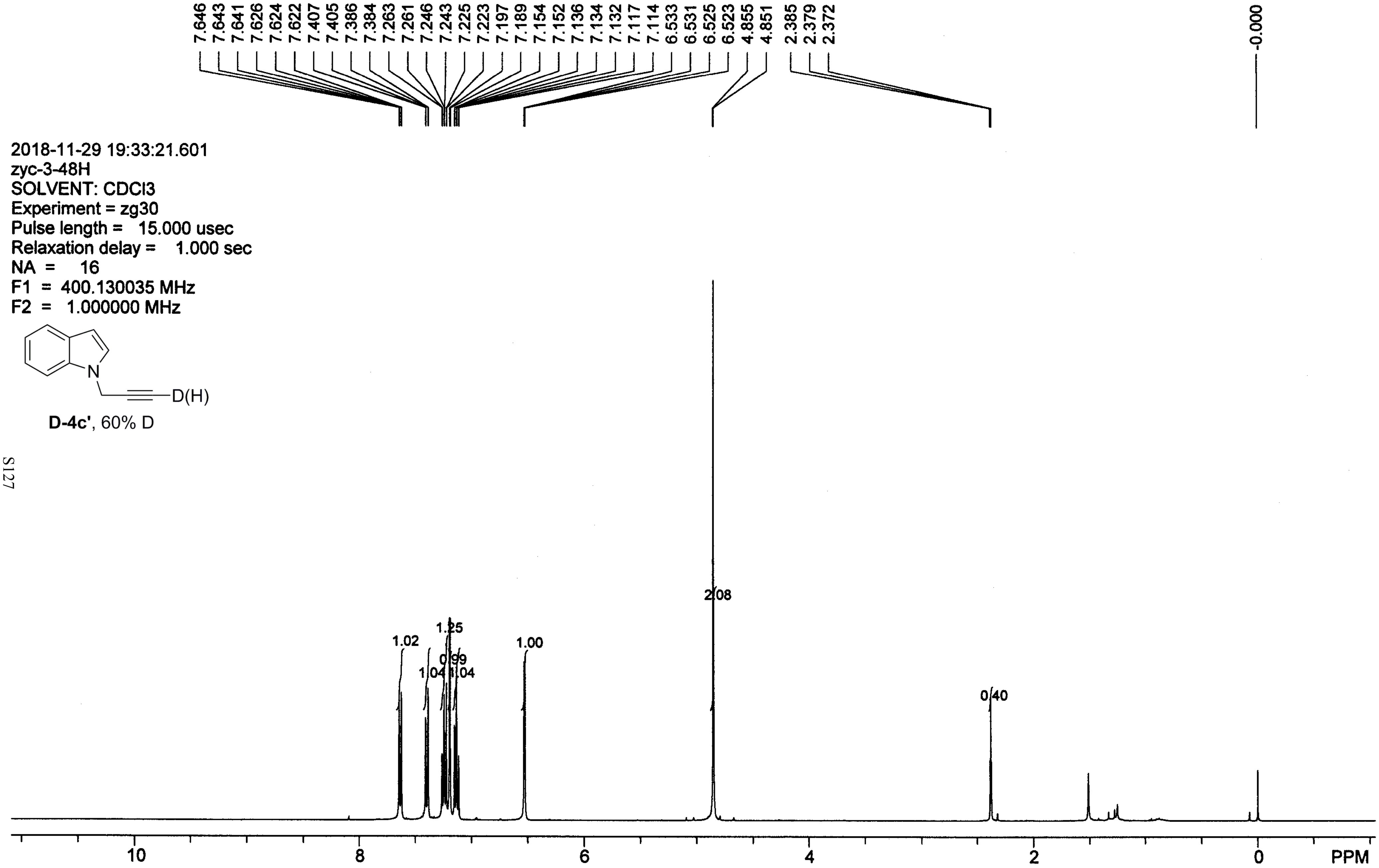

2018-11-29 19:33:21.601
zyc-3-48H
SOLVENT: CDCl3
Experiment = zg30
Pulse length = 15.000 usec
Relaxation delay = 1.000 sec
NA = 16
F1 = 400.130035 MHz
F2 = 1.000000 MHz

D-4c', 60% D

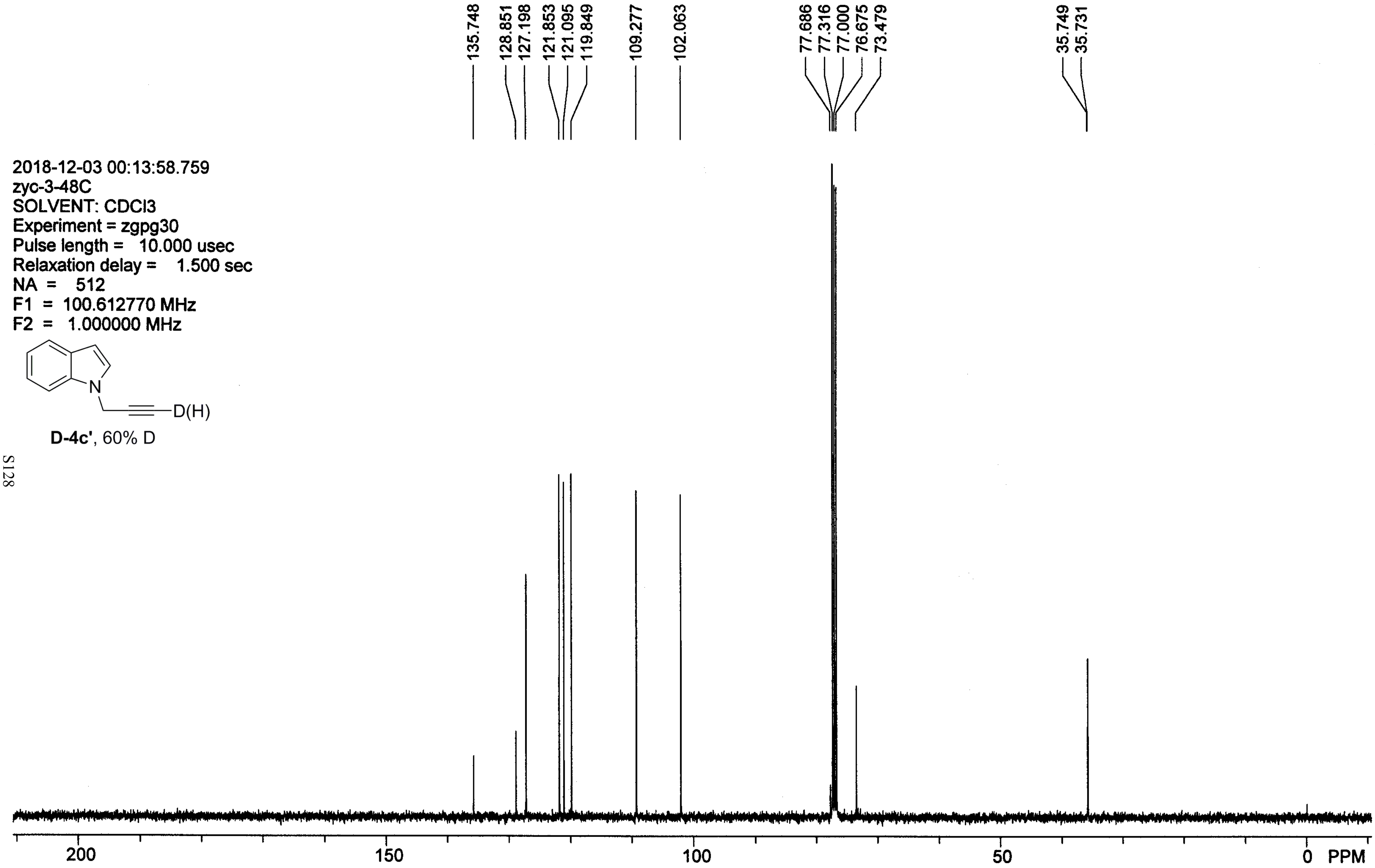
135.748
128.851
127.198
121.853
121.095
119.849
109.277
102.063
77.686
77.316
77.000
76.675
73.479
35.749
35.731
2018-12-03 00:13:58.759
zyc-3-48C
SOLVENT: CDCl3
Experiment = zgpg30
Pulse length = 10.000 usec
Relaxation delay = 1.500 sec
NA = 512
F1 = 100.612770 MHz
F2 = 1.000000 MHz
200
150
100
50
0 PPM
N
D(H)

D-4c', 60% D

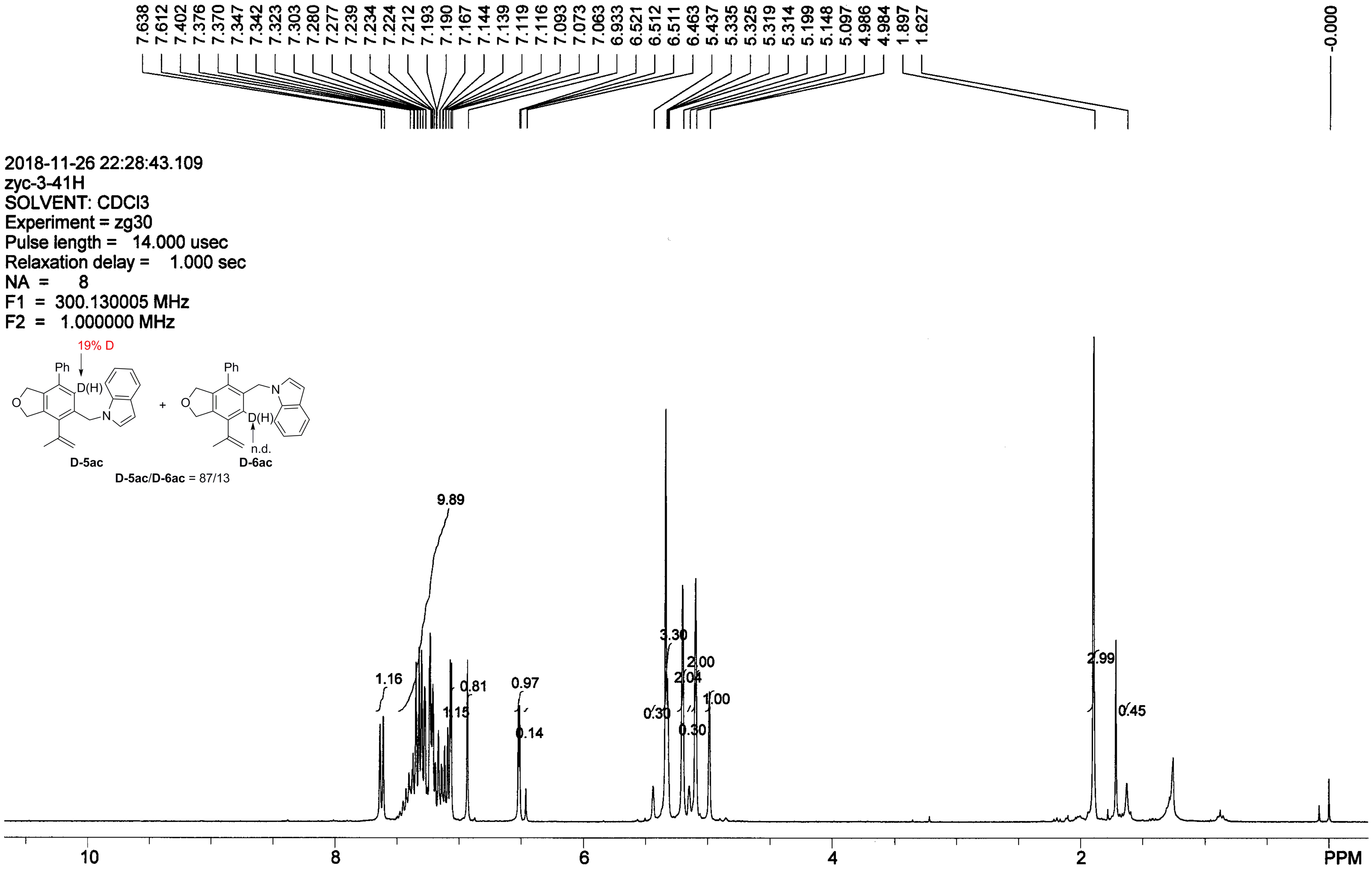

7.638
7.612
7.402
7.376
7.370
7.347
7.342
7.323
7.303
7.280
7.277
7.239
7.234
7.224
7.212
7.193
7.190
7.167
7.144
7.139
7.119
7.116
7.093
7.073
7.063
6.933
6.521
6.512
6.511
6.463
5.437
5.335
5.325
5.319
5.314
5.199
5.148
5.097
4.986
4.984
1.897
1.627
-0.000
2018-11-26 22:28:43.109
zyc-3-41H
SOLVENT: CDCl3
Experiment = zg30
Pulse length = 14.000 usec
Relaxation delay = 1.000 sec
NA = 8
F1 = 300.130005 MHz
F2 = 1.000000 MHz
19% D
Ph
D(H)
N
O
+
n.d.
D-5ac
D-6ac
D-5ac/D-6ac = 87/13
9.89
1.16
0.81
1.15
0.97
0.14
3.30
2.00
2.04
0.30
0.30
1.00
2.99
0.45
10
8
6
4
2
PPM

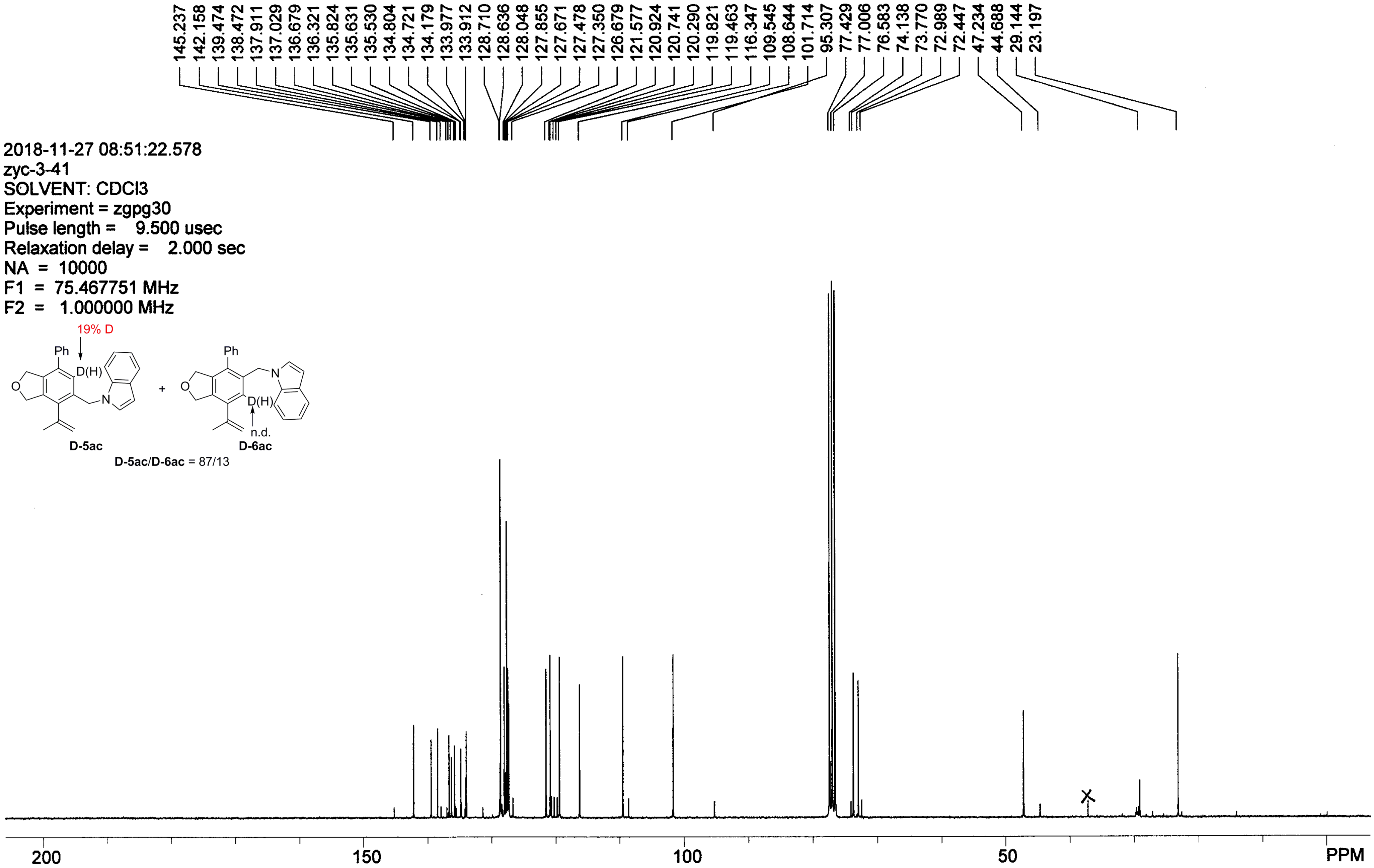

145.237
142.158
139.474
138.472
137.911
137.029
136.679
136.321
135.824
135.631
135.530
134.804
134.721
134.179
133.977
133.912
128.710
128.636
128.048
127.855
127.671
127.478
127.350
126.679
121.577
120.924
120.741
120.290
119.821
119.463
116.347
109.545
108.644
101.714
95.307
77.429
77.006
76.583
74.138
73.770
72.989
72.447
47.234
44.688
29.144
23.197
2018-11-27 08:51:22.578
zyc-3-41
SOLVENT: CDCl3
Experiment = zgpg30
Pulse length = 9.500 usec
Relaxation delay = 2.000 sec
NA = 10000
F1 = 75.467751 MHz
F2 = 1.000000 MHz
19% D
Ph
D(H)
N
O
+
n.d.
D-5ac
D-6ac
D-5ac/D-6ac = 87/13
200
150
100
50
PPM

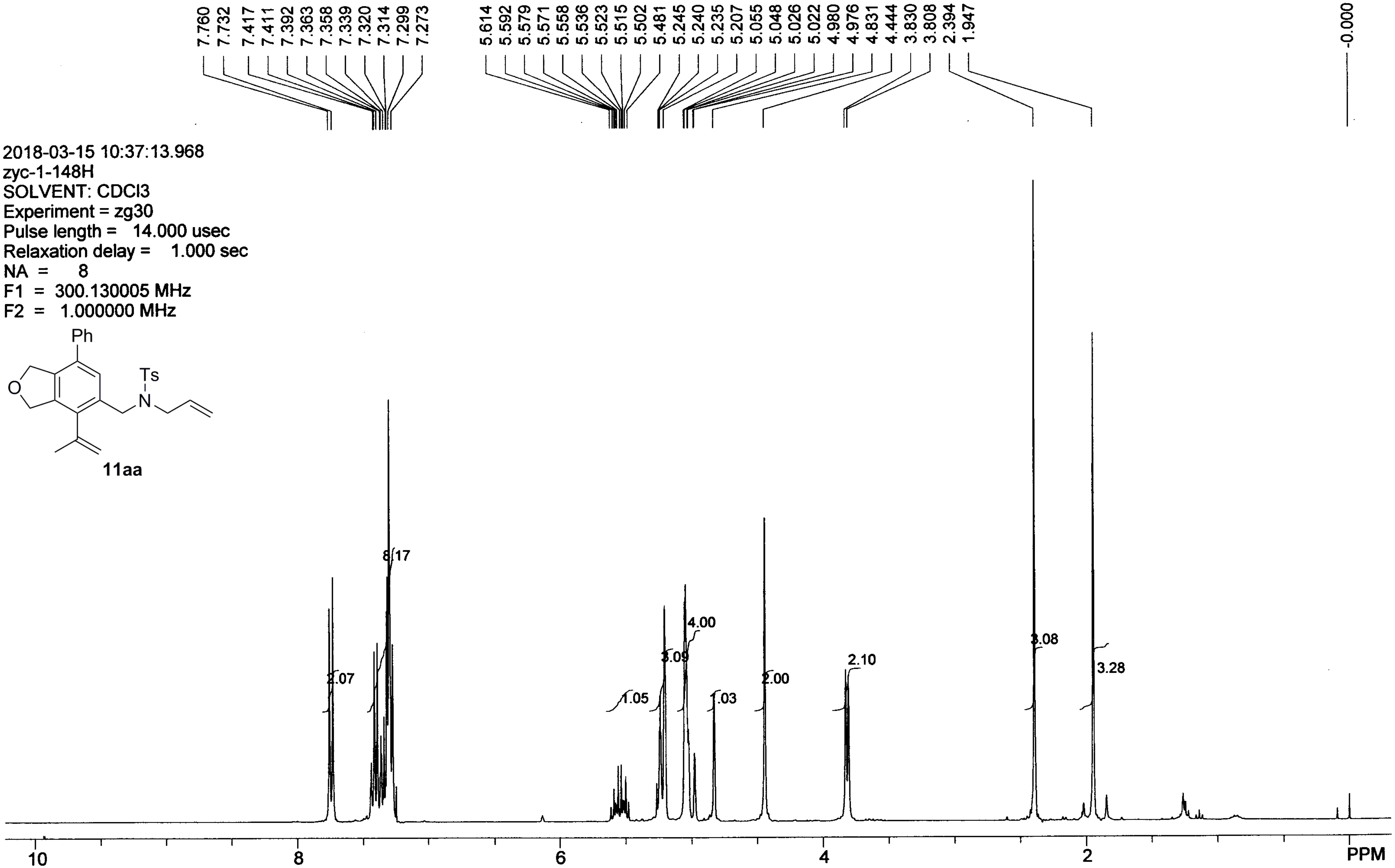

2018-03-15 10:37:13.968
zyc-1-148H
SOLVENT: CDCl3
Experiment = zg30
Pulse length = 14.000 usec
Relaxation delay = 1.000 sec
NA = 8
F1 = 300.130005 MHz
F2 = 1.000000 MHz

11aa

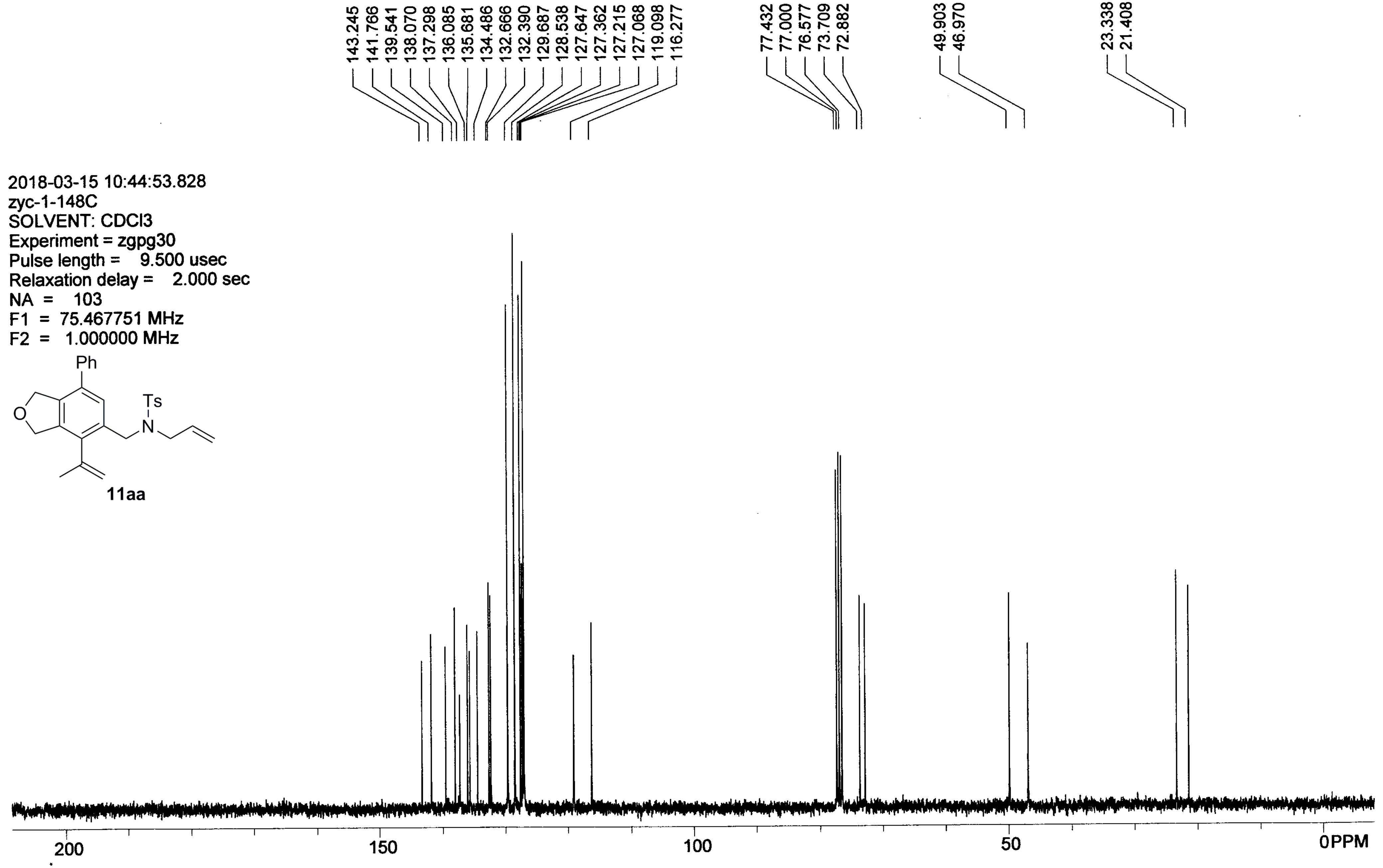
143.245
141.766
139.541
138.070
137.298
136.085
135.681
134.486
132.666
132.390
129.687
128.538
127.647
127.362
127.215
127.068
119.098
116.277
77.432
77.000
76.577
73.709
72.882
49.903
46.970
23.338
21.408
2018-03-15 10:44:53.828
zyc-1-148C
SOLVENT: CDCl3
Experiment = zgpg30
Pulse length = 9.500 usec
Relaxation delay = 2.000 sec
NA = 103
F1 = 75.467751 MHz
F2 = 1.000000 MHz
Ph
Ts
N
O
11aa
200
150
100
50
0PPM

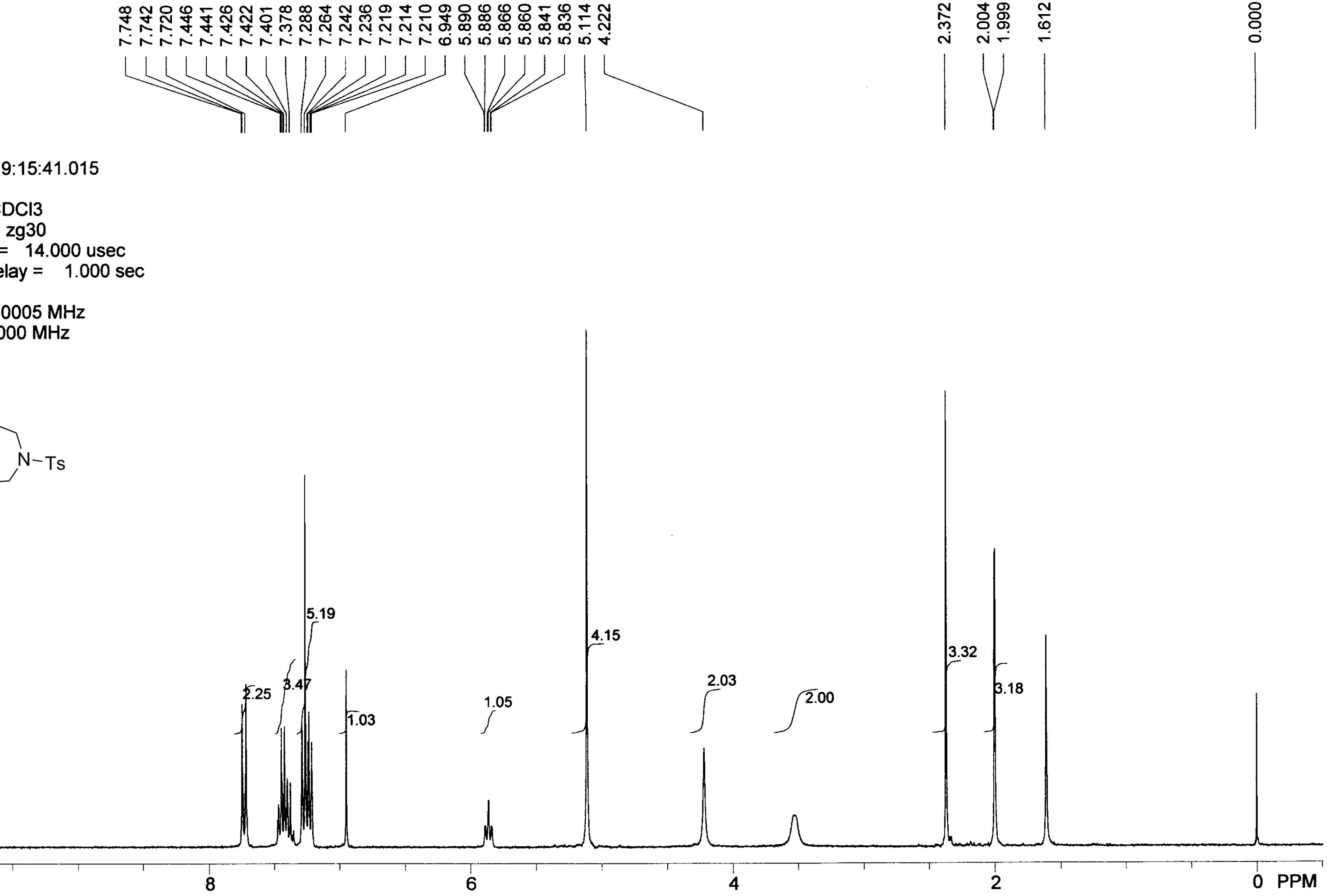

2018-03-26 19:15:41.015
zyc-1-164H
SOLVENT: CDCl3
Experiment = zg30
Pulse length = 14.000 usec
Relaxation delay = 1.000 sec
NA = 8
F1 = 300.130005 MHz
F2 = 1.000000 MHz

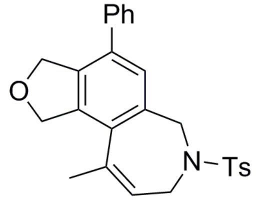

8aa

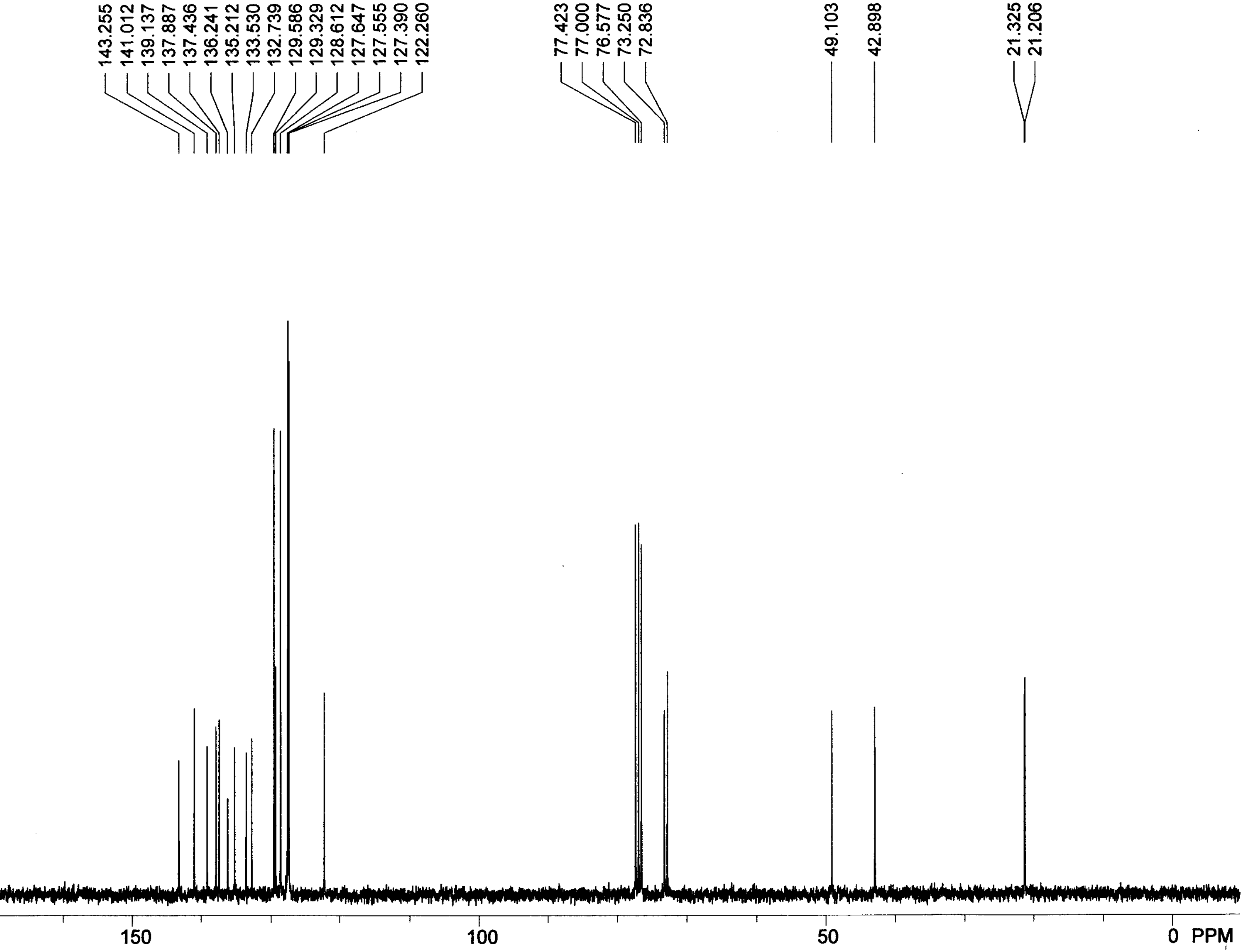
143.255
141.012
139.137
137.887
137.436
136.241
135.212
133.530
132.739
129.586
129.329
128.612
127.647
127.555
127.390
122.260
77.423
77.000
76.577
73.250
72.836
49.103
42.898
21.325
21.206
2018-03-26 19:29:01.671
zyc-1-164C
SOLVENT: CDCl3
Experiment = zgpg30
Pulse length = 9.500 usec
Relaxation delay = 2.000 sec
NA = 203
F1 = 75.467751 MHz
F2 = 1.000000 MHz
200
150
100
50
0 PPM

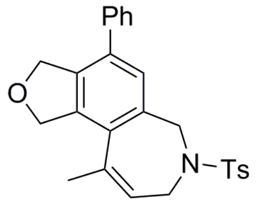
Ph
O
N
Ts

8aa

2016-03-26 16:39:23.843
wwt-3-51
SOLVENT: CDCl3
Experiment = zg30
Pulse length = 14.000 usec
Relaxation delay = 1.000 sec
NA = 8
F1 = 300.130005 MHz
F2 = 1.000000 MHz

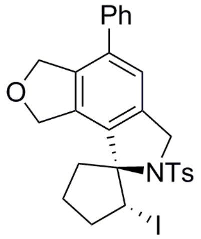

9ba

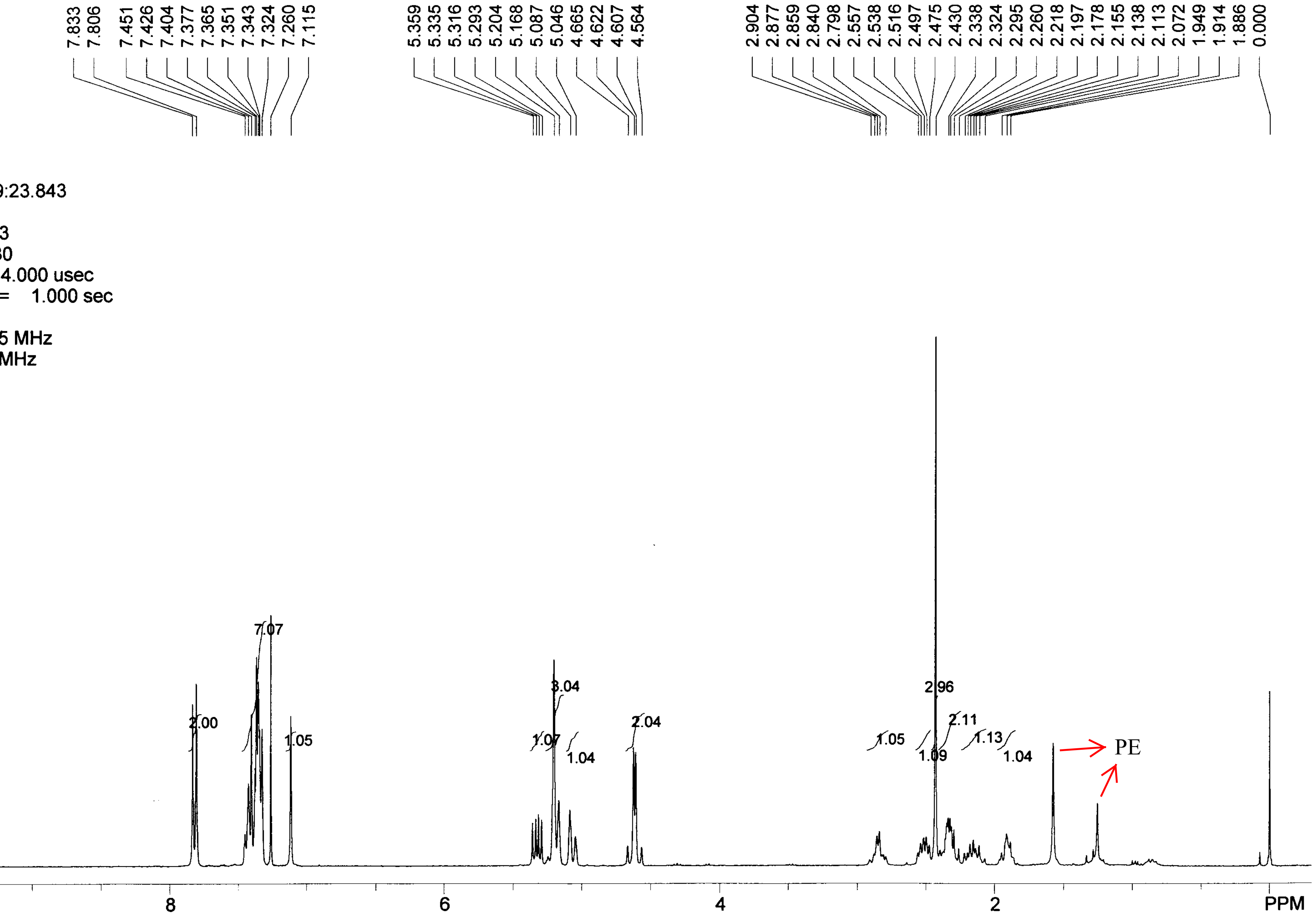

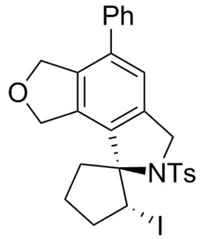
Ph
O
NTs
I

9ba

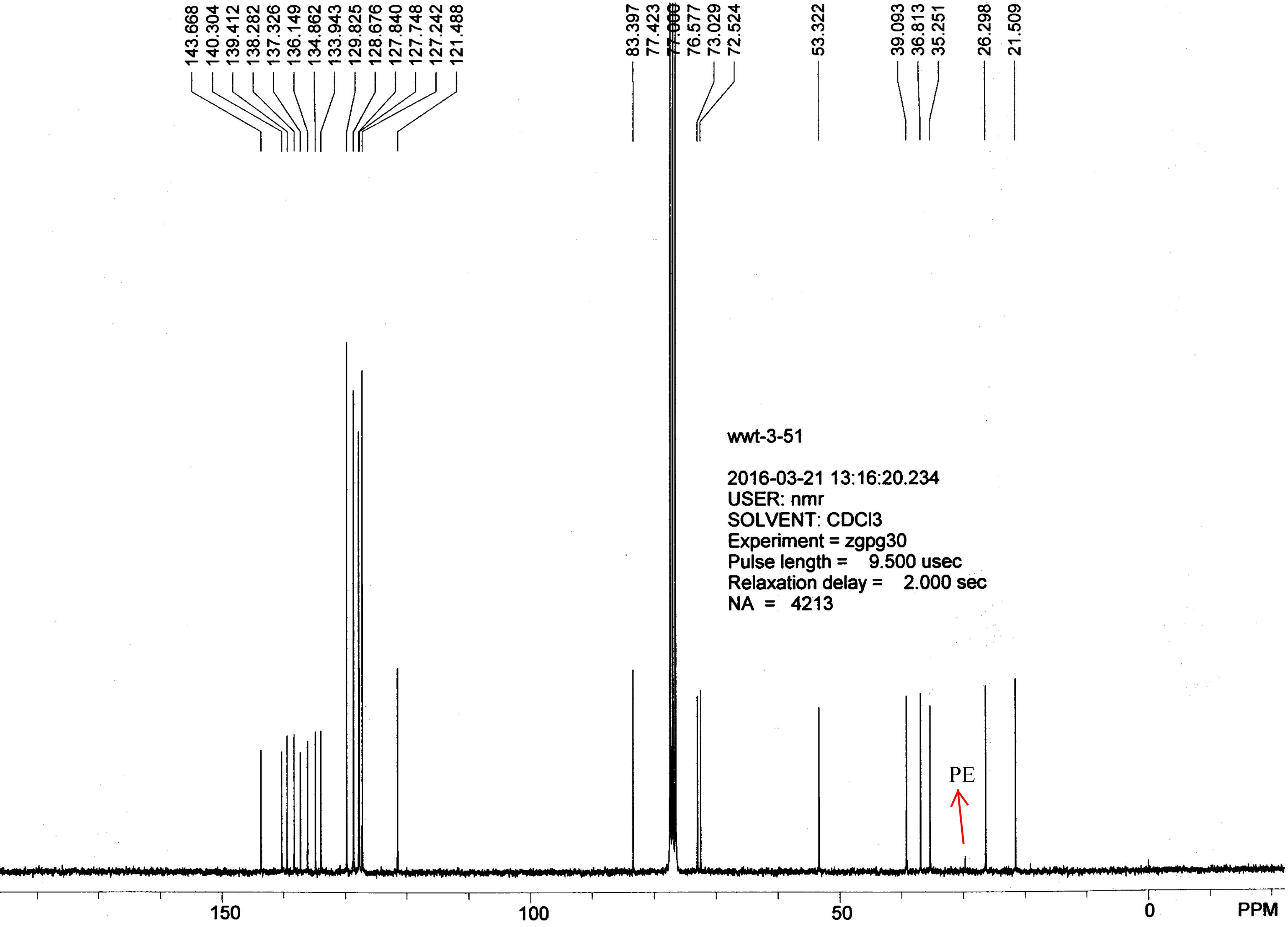
143.668
140.304
139.412
138.282
137.326
136.149
134.862
133.943
129.825
128.676
127.840
127.748
127.242
121.488
83.397
77.423
77.000
76.577
73.029
72.524
53.322
39.093
36.813
35.251
26.298
21.509
wwt-3-51
2016-03-21 13:16:20.234
USER: nmr
SOLVENT: CDCl3
Experiment = zgpg30
Pulse length = 9.500 usec
Relaxation delay = 2.000 sec
NA = 4213
PE
200
150
100
50
0
PPM

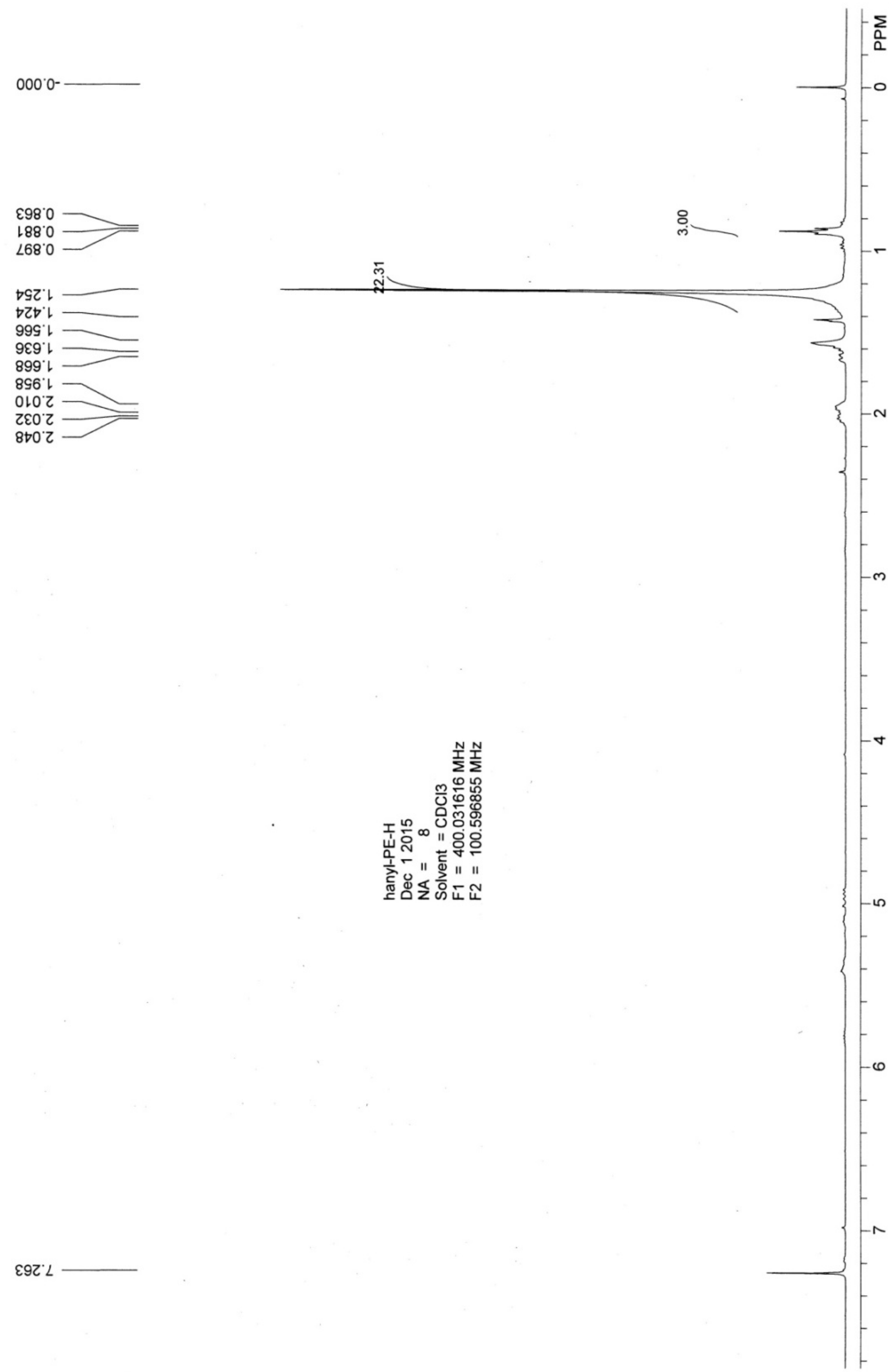
hanyl-PE-H
Dec 1 2015
NA = 8
Solvent = CDCl3
F1 = 400.031616 MHz
F2 = 100.596855 MHz
-0.000
0.863
0.881
0.897
1.254
1.424
1.566
1.636
1.668
1.958
2.010
2.032
2.048
7.263
22.31
3.00
PPM
0
1
2
3
4
5
6
7

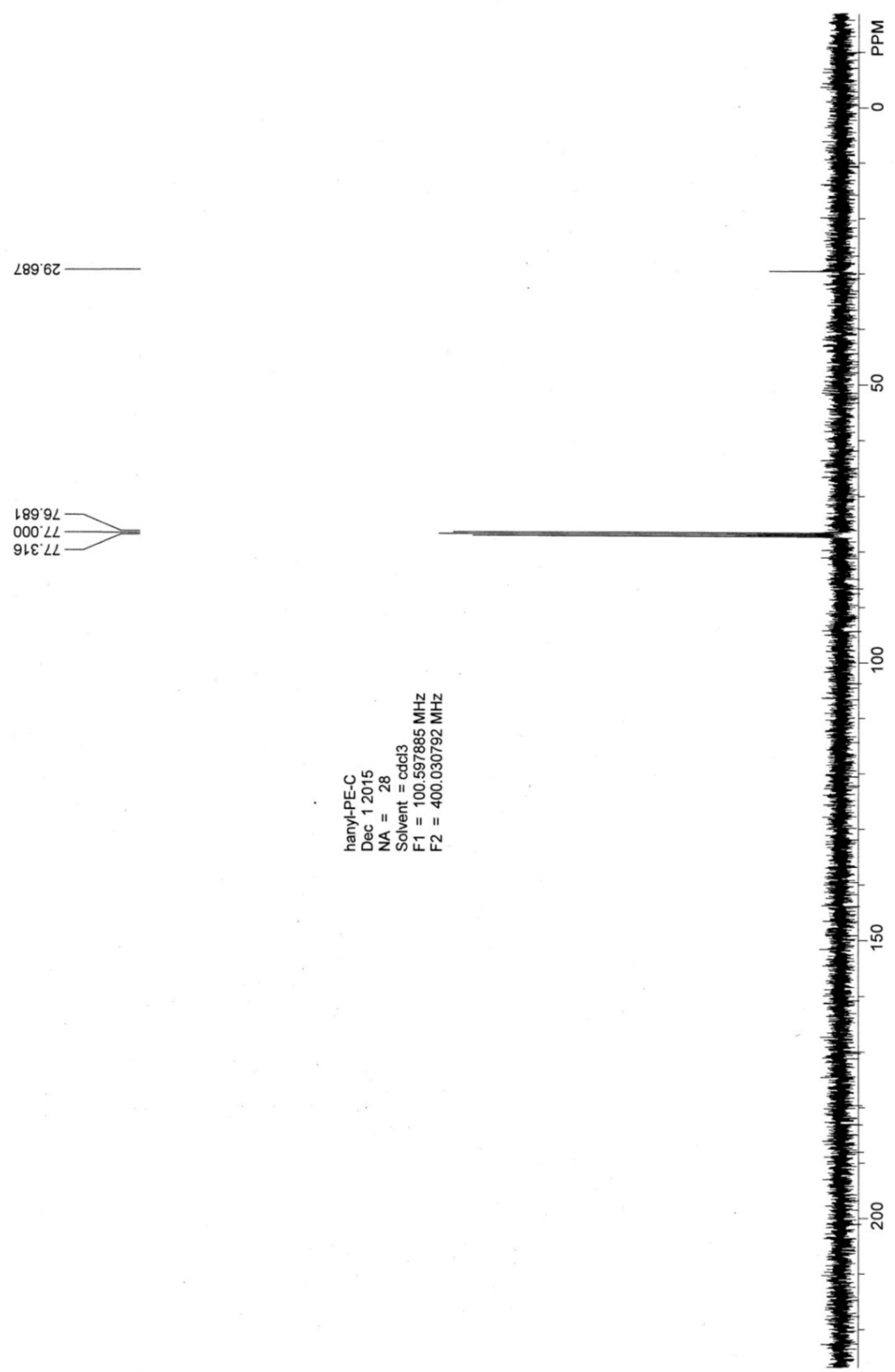
29.687
76.681
77.000
77.316
hanyl-PE-C
Dec 1 2015
NA = 28
Solvent = cdcl3
F1 = 100.597885 MHz
F2 = 400.030792 MHz
PPM
0
50
100
150
200

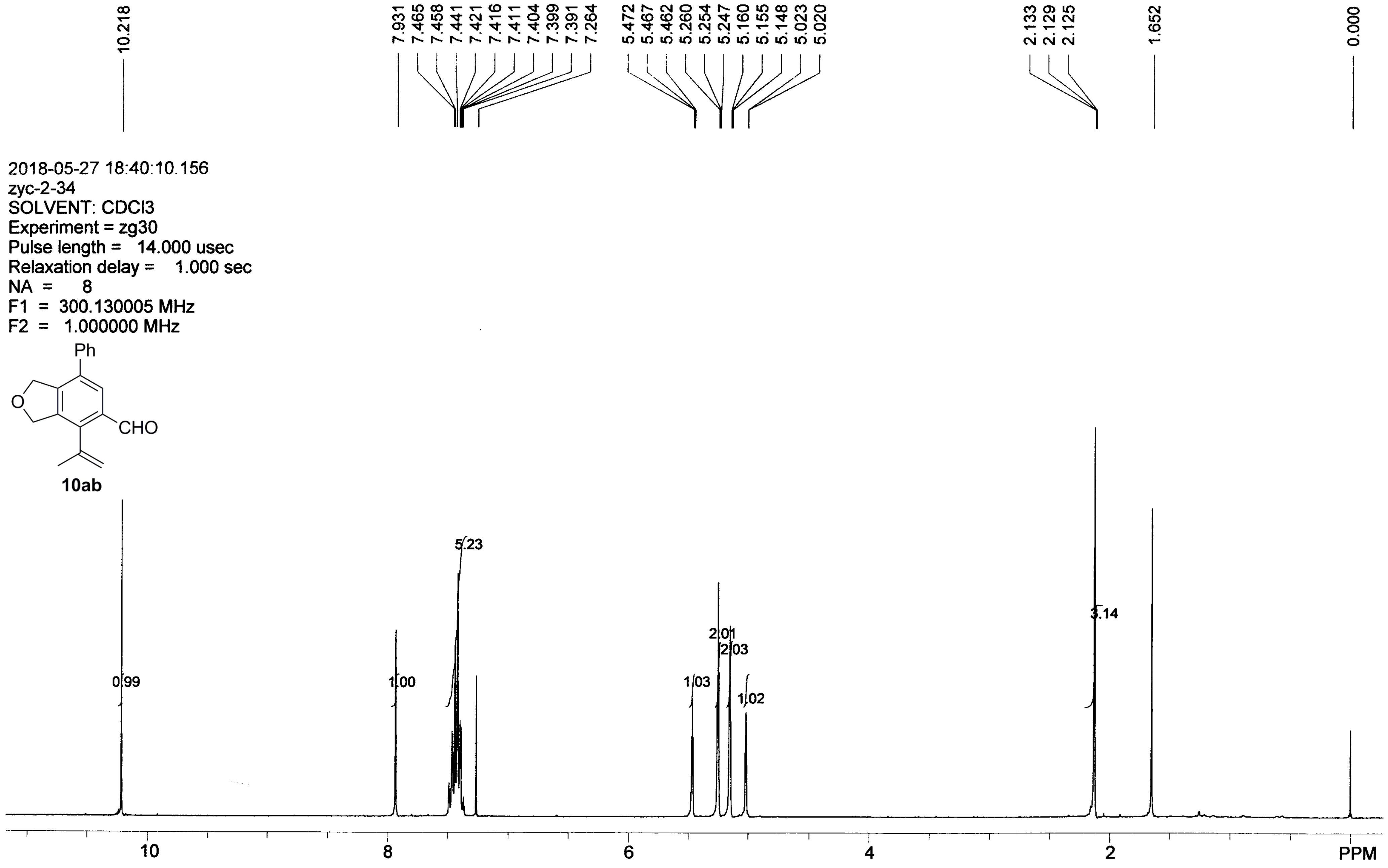
10.218
7.931
7.465
7.458
7.441
7.421
7.416
7.411
7.404
7.399
7.391
7.264
5.472
5.467
5.462
5.260
5.254
5.247
5.160
5.155
5.148
5.023
5.020
2.133
2.129
2.125
1.652
0.000
2018-05-27 18:40:10.156
zyc-2-34
SOLVENT: CDCl3
Experiment = zg30
Pulse length = 14.000 usec
Relaxation delay = 1.000 sec
NA = 8
F1 = 300.130005 MHz
F2 = 1.000000 MHz
0.99
1.00
5.23
1.03
2.01
2.03
1.02
3.14
10
8
6
4
2
PPM
Ph
O
CHO

10ab

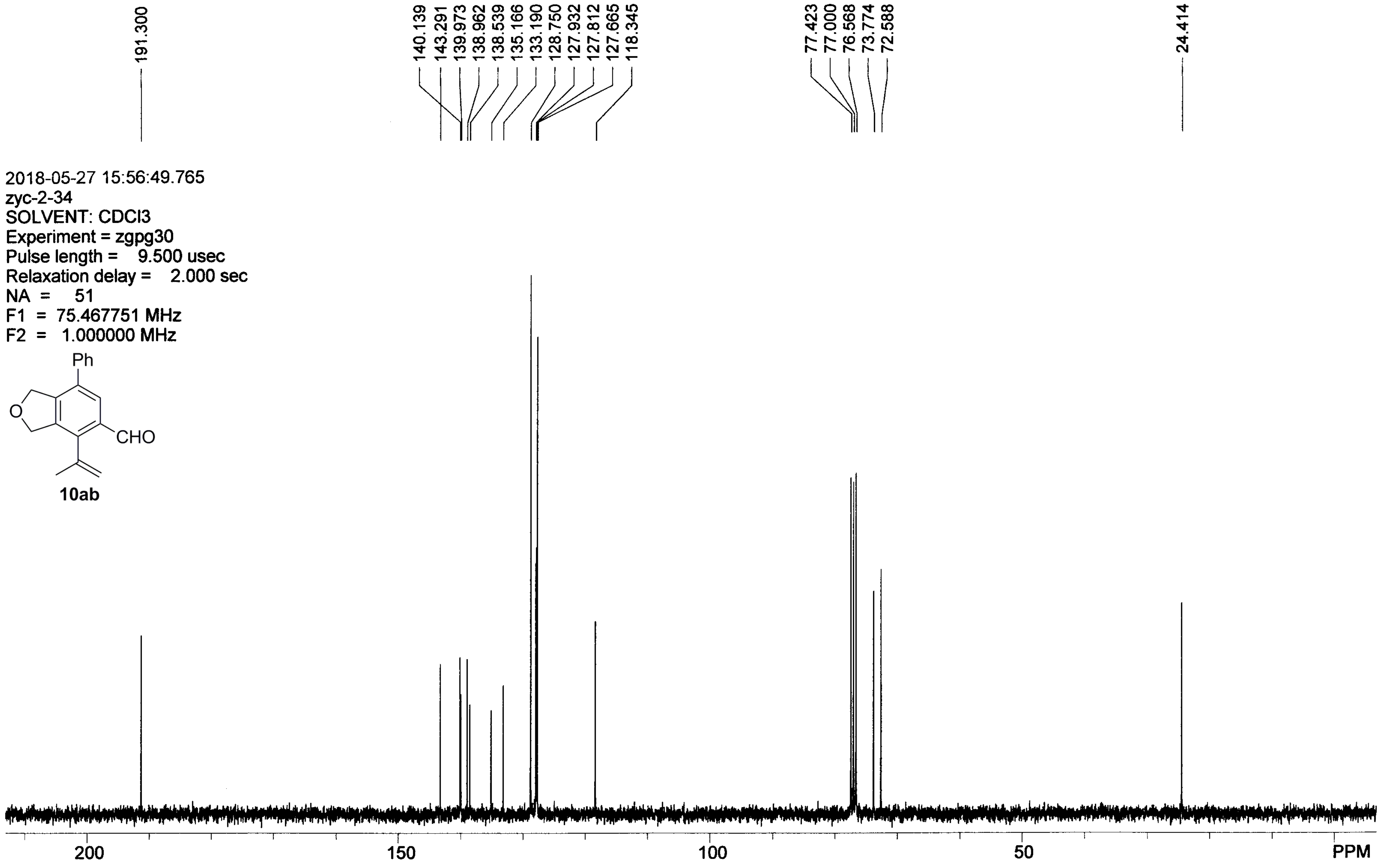
191.300
140.139
143.291
139.973
138.962
138.539
135.166
133.190
128.750
127.932
127.812
127.665
118.345
77.423
77.000
76.568
73.774
72.588
24.414
2018-05-27 15:56:49.765
zyc-2-34
SOLVENT: CDCl3
Experiment = zgpg30
Pulse length = 9.500 usec
Relaxation delay = 2.000 sec
NA = 51
F1 = 75.467751 MHz
F2 = 1.000000 MHz
Ph
O
CHO
10ab
200
150
100
50
PPM
